# Singlet, Doublet, and Triplet Emissions of Diarylamine‐Modified Bismuth Pincer Complexes

**DOI:** 10.1002/chem.202500384

**Published:** 2025-04-28

**Authors:** Marcel Geppert, Michelle Müller, Katharina J. Scherer, Jessica Henzler, Rainer F. Winter

**Affiliations:** ^1^ Fachbereich Chemie Universität Konstanz, Universitätsstraße 10 78457 Konstanz Germany

**Keywords:** bismuth, doublet emission, heavy atom effect, phosphorescence, triarylamine cations

## Abstract

We present six bismuth complexes **(*NC^R^N*)BiX_2_** (X = Cl, I) with diarylamine‐modified pincer ligands (*NC^R^N* = (4‐R‐C_6_H_4_)_2_N‐C_6_H_2_‐(CH_2_NMe_2_)_2_‐1,3; R = Me, O, NMe_2_) and report on their optoelectronic, photophysical, and electrochemical properties. The complexes exhibit intriguing photophysical behavior, with the *p*‐tolyl and *p‐*anisyl derivatives showing phosphorescence at 77 K in frozen solvent matrices and at room temperature (r.t.) in the solid state. In THF solutions at r.t., only ligand‐based fluorescence is observed with strongly reduced quantum yields compared to free proligands ***NCH^R^N***. Electrochemical studies reveal up to three reversible one‐electron oxidations. The NMe_2_‐substituted complexes display the lowest oxidation potentials and the largest number of redox waves. Radical cations **[*NCH^NMe2^N*]*^+^*** and **[(*NC^NMe2^N*)BiX_2_]*^+^*** are chemically stable and fluoresce weakly in the near‐infrared (NIR) at ca. 1200 nm.

## Graphical abstract

Reversibly oxidizable diarylamine‐modified phenyl‐bis(dimethylaminomethyl) pincer bismuth complexes and their radical cations emit via excited singlet and triplet or doublet excited states at 77 K in solvent matrices, in solution, or in the solid state.

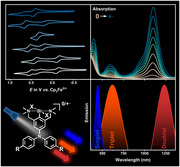

## 1 Introduction

The interplay between different electronic states—singlet, doublet,700 and triplet—is at the heart of photophysical phenomena that crucially determine the emissive properties of molecules. Excited singlet and doublet states give both rise to fluorescence emission, entailing spin‐allowed radiative decay to the respective electronic ground state. In contrast, phosphorescence emission requires spin‐forbidden intersystem crossing (ISC) from an initially generated singlet state *S*_n_ to an excited triplet state *T*_m_, and subsequent, likewise spin‐forbidden radiative decay to the singlet ground state *S*_0_.

While fluorescence emissions from excited singlet states are widely studied and utilized, emissions from excited doublet and triplet states are gaining increasing attention for advanced applications in diverse fields, such as optoelectronics and biomedicine, owing to their unique characteristics. Triplet‐state emitters are pivotal in technologies such as organic light‐emitting diodes (OLEDs)^[^ ^1^, ^2^, ^3^, ^4^ ^]^ and in dye‐sensitized solar cells (DSSCs),^[^ ^5^, ^6^, ^7^ ^]^ where they can increase the hypothetical limit of internal quantum efficiency from the 25% of pure singlet emitters to 100%.^[^ ^8^ ^]^ They also find application in photodynamic therapy (PDT)^[^ ^9^, ^10^, ^11^, ^12^ ^]^ and in bioimaging.^[^ ^13^, ^14^, ^15^, ^16^, ^17^ ^]^ Molecules with a doublet ground state were historically believed to suffer from emission quenching rather than emitting light,^[^ ^18^, ^19^ ^]^ but are now recognized as offering compelling advantages in specific domains, particularly as emitters in the near‐infrared (NIR),^[^ ^20^ ^]^ for visible light communication (VLC) systems that are critical to the evolution of the Internet of Things (IoT),^[^ ^21^, ^22^ ^]^ and in OLEDs.^[^ ^23^ ^]^ NIR emission at wavelengths of 700–1000 nm falls in the biological “window of transparency,” coinciding with the absorption minimum of biological tissue. The resulting deep tissue penetration^[^ ^24^ ^]^ renders such NIR emitters of great relevance for biomedical applications, including noninvasive imaging,^[^ ^25^ ^]^ photobiomodulation therapy (PBMT),^[^ ^26^ ^]^ and blood oximetry.^[^ ^27^ ^]^ Most of the work on doublet emitters has focused on heavily chlorinated derivatives of the triphenylmethyl (trityl) radical, like the perchlorotriphenylmethyl (PTM) and trichlorotriphenylmethyl (TTM). They also serve as the acceptor (A) component in donor‐acceptor (D‐A) dyads, in conjunction with a good electron donor (D) such as carbazole, imidazole, or a triarylamine. Such dyads often show enhanced emission properties and stability.^[^ ^18^, ^20^ ^]^

Triarylaminium radical cations are isolobal and isosteric with trityl radicals. Known primarily for their intense absorption in the visible (vis), often displaying blue to green colors,^[^ ^28^, ^29^, ^30^, ^31^, ^32^, ^33^ ^]^ and their utility as oxidizing agents (e.g., “Magic Blue” or “Magic Blue's Cousin”),^[^ ^34^ ^]^ they have only rarely been studied for their emissive properties. Following an initial, passing remark in a paper from 1994 on weak NIR fluorescence from Magic Blue and its methoxy derivative,^[^ ^35^ ^]^ more recent work has demonstrated doublet emission at 580 nm from in‐situ surface‐oxidized crystals of tris(*p*‐tolyl)amine and films made thereof.^[^ ^36^ ^]^

We recently reported on the bismuth complex **(*NC^Me^N*)BiCl_2_** with a bis(*p*‐tolyl)amine‐appended pincer ligand, which was prepared en route to complexes **(*NC^Me^N*)Bi(SAr)_2_** with pyrene‐ or coumarin‐derived mercapto ligands.^[^ ^37^ ^]^ These complexes were designed to harness the heavy‐atom effect (HAE) of the bismuth ion for promoting ISC and converting dye‐based fluorescence into phosphorescence, a property commonly exploited in complexes of the noble metals platinum, gold, iridium, osmium, or rhenium.^[^ ^38^, ^39^, ^40^, ^41^, ^42^, ^43^, ^44^, ^45^ ^]^ Bismuth offers a compelling alternative to these costly metals, owing to its affordable pricing, the significantly lower toxicity of most of its complexes, larger natural abundance, and having the highest spin‐orbit coupling (SOC) constant among all nonradioactive elements.^[^ ^46^, ^47^, ^48^, ^49^, ^50^ ^]^ In spite of these advantages, phosphorescent bismuth complexes remain relatively scarce compared to complexes of gold or the platinum group metals.^[^ ^37^, ^51^, ^52^, ^53^, ^54^, ^55^, ^56^, ^57^, ^58^, ^59^, ^60^, ^61^, ^62^, ^63^, ^64^, ^65^, ^66^, ^67^, ^68^, ^69^, ^70^, ^71^ ^]^ Critical challenges are the relatively weaker Bi‐ligand bonds and the lesser propensity of the Bi^3+^ ion to engage in charge‐transfer excitations, i.e., for providing an efficient pathway for ISC according to El‐Sayeds rule,^[^ ^72^, ^73^, ^74^, ^75^ ^]^ which is both due to the lack of d‐orbital contributions to M‐L bonding.

**Scheme 1:**
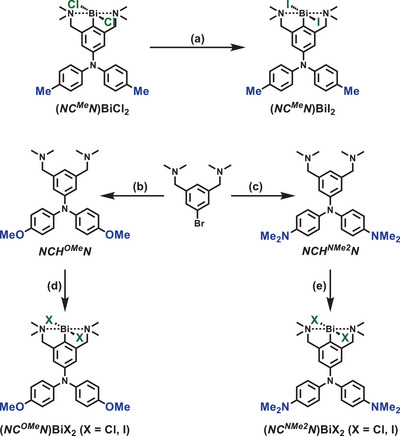
Synthesis of complexes **(*NC^R^N*)BiX_2_** (R = Me, MeO, NMe_2_; X = Cl, I): (a) KI, CH_2_Cl_2_/EtOH/H_2_O, 24 hours, r.t.; (b) bis(*p*‐anisyl)amine, P*^t^* Bu_3_, NaO*^t^* Bu, [Pd_2_(dba)_3_], toluene, 3 days, reflux; (c) bis(*p*‐dimethylaminophenyl)amine, P*^t^* Bu_3_, NaO*^t^* Bu, [Pd_2_(dba)_3_], toluene, 3 days, reflux; (d) (i) *n*‐BuLi, *n*‐hexane, 24 hours, reflux; (ii) BiX_3_ (X = Cl, I), Et_2_O, 1 hour, −78 °C, then r.t., 24 hours; (e) (i) *n*‐BuLi, *n‐*hexane, 24 hours, reflux; (ii) BiX_3_ (X = Cl, I), Et_2_O, 1 hour, −78 °C, then r.t., 24 hours.

In the present study, we report on six bismuth complexes **(*NC^R^N*)BiX_2_** (R = Me, MeO, NMe_2_; X = Cl, I; see Scheme 1) with varied *para*‐substituents on the appended diarylamine donor and their photophysical and electrochemical properties. Depending on the conditions and the oxidation state (neutral: frozen matrices of 2‐MeTHF, toluene, and EtOH at 77 K, THF solution, or in the solid state at r.t.; radical cations in CH_2_Cl_2_ solution at r.t.), these compounds were found to emit via excited singlet, doublet, or triplet states. This work therefore highlights the potential of bismuth‐based complexes as versatile and tunable platforms for photophysical applications. They hence contribute to fill the gap between phosphorescent complexes of the noble metals and more sustainable alternatives.

## 2 Results and Discussion

### 2.1 Synthesis and NMR Spectra

**(*NC^Me^N*)BiI_2_** was synthesized in 80% yield from the known dichlorido congener^[^ ^37^ ^]^ by halide exchange with excess KI in a two‐layer solvent system of H_2_O/EtOH/CH_2_Cl_2_, in a manner analogous to the synthesis of parent **(*NCN*)BiI_2_**.^[^ ^76^ ^]^ Complexes **(*NC^OMe^N*)BiX_2_** and **(*NC^NMe2^N*)BiX_2_** (X = Cl, I) were synthesized in two steps. buchwald−hartwig coupling of 1‐bromo‐3,5‐bis(dimethylaminomethyl)benzene and bis(*p*‐anisyl)‐ or bis(*p*‐dimethylaminophenyl)amine gave the proligands ***NCH^OMe^N*** and ***NCH^NMe2^N*** in 89% and 92% yield. Deprotonation with *n*‐BuLi and subsequent reaction with BiCl_3_ or BiI_3_ then provided the target complexes **(*NC^R^N*)BiX_2_** in good (X = Cl: 73‐77%), or modest (X = I: 16‐26%) yields. ^1^H‐ and ^13^C‐NMR spectra (see Figures S1‐S2 and S7‐S14 of the Supporting Information) of the complexes resemble those of other *NCN* pincer dihalogenido bismuth complexes in the literature.^[^ ^37^, ^76^ ^]^ The new complexes are stable toward air and water.

### 2.2 Absorption Spectra and TD‐DFT Calculations

**Figure 1:**
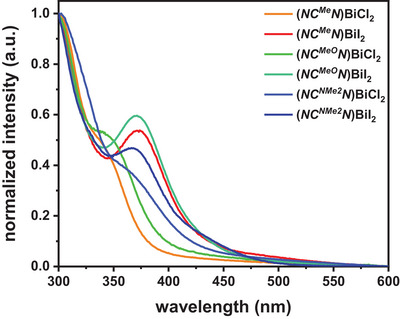
UV/vis absorption spectra of complexes **(*NC^R^N*)BiX_2_** in THF at r.t.

Insight into the electronic structures and the character of the electronic transitions of complexes **(*NC^R^N*)BiX_2_** was obtained by amalgamating the results from TD‐DFT calculations with the experimental absorption spectra shown in Figure 1. Every complex absorbs strongly in the UV region, with a main band at ca. 300 nm and a second, weaker band at 350–375 nm, tailing into the vis. The latter band is better resolved for the iodido complexes, while it appears blue‐shifted for the chlorido complexes, merging as a shoulder into the first band. Extinction coefficients *ε* (see Table 1 and Figure S30) follow the ordering **(*NC^R^N*)BiI_2_** > **(*NC^R^N*)BiCl_2_** > ***NCH^R^N*** and are in the range of 7700‐23 000 Lmol^−1^ cm^−1^.

**Table 1:** Absorption and photoluminescence data for proligands ***NCH^R^N*** and complexes **(*NC^R^N*)BiX_2_** (F = fluorescence, P = phosphorescence).

	*λ*_max_ (nm) [*ε*_λ_] (×10^−3^∙M^−1^ cm^−1^)	*λ*_em_ [nm]	*λ*_exc_ [nm]	*τ*_F_ [ns]^[d]^	*τ*_P_ [µs]^[d]^	*ϕ*_em_
***NCH^Me^N***	301 (7.7) 352 (2.3)	^[a]^F: 421 ^[b]^F: 380 494	314 345 359	2.4 [93%], 16 [7%] 4.6 [71%], 23 [29%] 3.3 [36%], 8.1 [64%]	‐ ‐ ‐	79% 1.5% ‐
**(*NC^Me^N*)BiCl_2_**	300 (13) 343 (5.9)	^[a]^F: 445 ^[a]^P: 585 ^[b]^F: 379 506 ^[c]^P: 634	304, 353 304, 353 300 316 ‐	2.3 [81%], 9.7 [19%] ‐ 1.9 [47%], 6.3 [53%] 2.4 [62%], 8.7 [38%] ‐	‐ 5.6 [58%], 20 [42%] ‐ ‐ 0.14 [59%], 0.96 [41%]	28% 47% <0.1% 1.3%
**(*NC^Me^N*)BiI_2_**	300 (23) 373 (12)	^[a]^F: 446 ^[a]^P: 612 ^[b]^F: 381 505 ^[c]^P: 653	316 316, 365 300 317 ‐	2.4 [80%], 9.6 [20%] ‐ 2.2 [28%], 6.5 [72%] 2.8 [45%], 9.9 [55%] ‐	‐ 12 [36%], 21 [64%] ‐ ‐ 0.61 [72%], 2.0 [28%]	2% 29% <0.1% <0.1%
***NCH^OMe^N***	302 (11) 352 (1.2)	^[a]^F: 428 ^[b]^F: 386	310 304, 354	2.4 [78%], 5.4 [22%] 5.1 [100%]	‐ ‐	83% 5.6%
**(*NC^OMe^N*)BiCl_2_**	300 (19) 345 (9.7)	^[a]^F: 447 ^[a]^P: 611 ^[b]^F: 389 ^[c]^P: 612	304, 353 300, 353 305, 350 ‐	3.1 [67%], 9.6 [33%] ‐ 0.55 [6%], 4.9 [94%] ‐	‐ 12 [87%], 31 [13%] ‐ 0.29 [44%], 1.0 [56%]	3% 47% <0.1% 0.9%
**(*NC^OMe^N*)BiI_2_**	300 (21) 371 (13)	^[a]^F: 450 ^[a]^P: 655 ^[b]^F: 393 ^[c]^P: 710	309 309, 362 308, 347 ‐	1.9 [39%], 4.4 [61%] ‐ 0.85 [5%], 4.9 [95%] ‐	‐ 6.4 [42%], 15 [58%] ‐ 0.59 [70%], 1.8 [30%]	0.3% 14% <0.1% 0.2%
***NCH^NMe2^N***	309 (14) 370 (1.2)	^[a]^F: 450 ^[b]^F: 422	326 311	2.2 [73%], 3.6 [27%] 2.4 [91%], 11 [9%]	‐ ‐	73% 2.5%
**(*NC^NMe2^N*)BiCl_2_**	302 (17) 365 (6.2)	^[a]^F: 466 ^[b]^F: 429	319 310, 368	2.5 [57%], 9.2 [43%] 2.2 [82%], 9.0 [18%]	‐ ‐	1.3% <0.1%
**(*NC^NMe2^N*)BiI_2_**	304 (22) 367 (10)	^[a]^F: 477 ^[b]^F: 431	318 318	2.2 [60%], 7.7 [40%] 2.3 [53%], 9.0 [47%]	‐ ‐	0.3% <0.1%

^[a]^ in 2‐MeTHF at 77 K. The reported quantum yields correspond to *λ*_exc_ = 350 nm.

^[b]^ in THF at r.t.

^[c]^ in the solid state at r.t.

^[d]^ relative contributions in [%].

Our TD‐DFT calculations (Figures S35 to S40) indicate that both absorption bands have ligand‐to‐metal charge‐transfer (LMCT) character and involve the transfer of electron density from the electron‐rich triarylamine donor to the acceptor MOs LUMO and LUMO+1. The latter correspond to antibonding combinations between the empty p‐orbitals of the Bi^3+^ ion and p‐orbitals of the halogenido ligands or with nitrogen lone‐pairs at the *NC^R^N* ^−^ pincer ligand. For complexes **(*NC^R^N*)BiX_2_** (X = Cl, I) with OMe or NMe_2_ donor substituents at the peripheral amine‐bonded phenyl rings, the latter are augmented by contributions from π→π* excitations that are confined within the pincer ligand. In the iodido complexes, additional I^−^→Bi^3+^ (5p) LMCT and I^−^→pincer ligand charge transfer (LL´CT) contributions are noted as well.

### 2.3 Photoluminescence Properties

**Figure 2:**
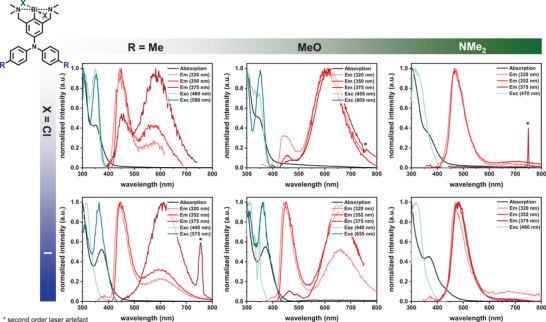
Normalized PL spectra of 10 µM solutions of complexes **(*NC^R^N*)BiX_2_** at 77 K in 2‐MeTHF. Absorption spectra are shown as black, emission spectra as red, and excitation spectra as cyan lines.

The emissive properties of the complexes **(*NC^R^N*)BiX_2_** and the corresponding proligands were investigated in a frozen 2‐MeTHF glass at 77 K, (see Figure 2) and as THF solutions as well as in the solid state at r.t. Gratifyingly, all compounds proved to be stable toward continuous laser irradiation (365 nm, 10 W), with no signs of degradation observed over 10 minutes. (see Figures S15‐S20). An exception was observed for CH_2_Cl_2_ solutions of **(*NC^NMe2^N*)BiX_2_** (X = Cl, I), where, in the presence of air, oxidation to the radical cations occurred UV irradiation (*vide infra*). In frozen 2‐MeTHF, the complexes with the ***NC^Me^N^−^*** and the ***NC^OMe^N^−^*** pincer ligand show dual emission, with weak, blue, ligand‐based fluorescence and significantly more intense orange to red phosphorescence. Phosphorescence quantum yields are 47% for the two chlorido complexes and 29% or 14% for their iodido congeners, with lifetimes in the range of 1‐30 µs. No consistent influence of the halogenido ligands on the phosphorescence lifetimes was found, while the phosphorescence quantum yields decrease from X = Cl^−^ to X = I^−^. This contrasts with the recent discovery of luminescent β‐diketiminate aluminum complexes LAlX_2_, where the phosphorescence quantum yield in the crystalline state increased in the order Cl^−^ < Br^−^ < I^−^.^[^ ^77^ ^]^

As a result of the slightly varied characters of the underlying excitations, the intensity ratio between the phosphorescence and fluorescence emissions depends on the excitation wavelength *λ*_exc_. Specifically, phosphorescence becomes more and more prevalent as λ_exc_ is increased, i.e., as LMCT excitations contribute more to the absorption envelope. In contrast, complexes (***NC^NMe2^N*)BiX_2_** with the strong NMe_2_‐donor substituents and the corresponding free proligand solely emit via fluorescence, with only a hint of a very weak phosphorescence for (***NC^NMe2^N*)BiI_2_**. The rather drastic reduction in quantum yields from 73% for the proligand to 1.3% or 0.3% for (***NC^NMe2^N*)BiX_2_** (Table 1, Figure S51) attests to the efficacy of the Bi ion to impart ISC and the presence of additional detrimental, radiationless decay paths for the complexes of this particular electron‐rich ligand. The quenching of fluorescence of organic dyes by incorporated heavy atoms is a frequent observation.^[^ ^78^, ^79^, ^80^, ^81^ ^]^

As TD‐DFT calculations suggested CT character of the relevant excitations in the absorption spectra, we also probed for solvent effects by recording PL spectra of the complexes **(*NC^R^N*)BiCl_2_** (R = Me, OMe, NMe_2_) at 77 K in the glass‐forming solvents toluene and EtOH. Absorption and emission wavelengths vary only slightly with solvent, indicating only minor differences between the relative energies of the triplet and singlet states. However, we noted a strong solvent effect on the bifurcation between the different emissions, so that the phosphorescence intensity and quantum yield decrease at the expense of fluorescence with increasing polarity, i.e., in the order toluene > 2‐MeTHF > EtOH (see Figures S52 and S53 and associated Tables). In particular, the complex **(*NC^OMe^N*)BiCl_2_** exhibits a phosphorescence quantum yield *ϕ*_P_ of 94% in toluene, which decreases to 7% in EtOH, while *ϕ*_F_ increases from 6% to 14%. **(*NC^NMe2^N*)BiCl_2_** is nonemissive in toluene but becomes fluorescent in 2‐MeTHF and EtOH, with quantum yields increasing from 1.3% to 10% (see Figure S54).

In THF solution at r.t., all six complexes exhibit only ligand‐based fluorescence. Emission spectra resemble those of the respective proligands closely (see Figure S57 and Table 1). However, the quantum yields are significantly reduced to below 0.1% as compared to 1.5% to 5.6% for the proligands. Fluorescence spectra and lifetimes of the complexes do not depend on whether the THF solutions are degassed or not (see Figures S55 and S56). Likewise, in the presence of dioxygen, no ^1^O_2_ emission is observed (see Figures S61 and S62). This does, however not rule out that short‐lived triplet states are populated, which, at r.t., deactivate via radiationless paths and with no signs of thermal repopulation of the emissive singlet state, i.e., thermally activated delayed fluorescence (TADF). Similar observations were reported for related complexes **(*p*‐R‐C_6_H_4_‐CH = CH‐*NCN*)PtCl** with stilbenoid *NCN* pincer ligands.^[^ ^82^ ^]^

The rather poorly soluble complexes **(*NC^Me^N*)BiX_2_** with *p*‐tolyl substituents at the diarylamine donor show a peculiarity in room‐temperature emission spectra, in that they exhibit an additional broad emission at 505 nm besides that of the ligand‐based fluorescence at 380 nm. Concentration‐dependent PL measurements reveal that the relative intensity of the 505 nm emission increases at higher concentrations *c* (see Figure S58). As shown in Figure S59 of the Supporting Information, likewise red‐shifted bands were observed in excitation spectra recorded at the maximum of the 505 nm emission. We attribute these red‐shifted bands to static excimers or higher aggregates, a behavior which has precedence in Bi complexes of ligands with an extended π‐surface.^[^ ^37^, ^71^, ^83^, ^84^ ^]^ The rather poor solubility of this particular complex suggests the presence of intermolecular interactions, as they were observed in its X‐ray structure.^[^ ^37^ ^]^ This argues for the presence as dimers or higher aggregates in more concentrated solutions, which absorb and are selectively excited at higher wavelengths than the monomers. This sets them apart from dynamic excimers, which only form after excitation. Both kinds of excimers show the same broadened, red‐shifted emission compared to the monomer.^[^ ^85^ ^]^

**Figure 3:**
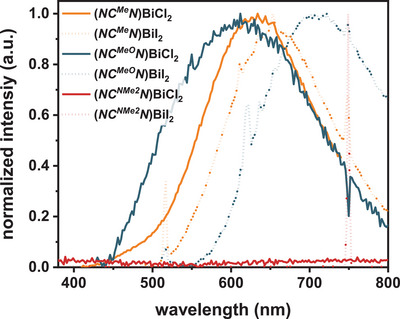
Solid‐state emissions of complexes **(*NC^R^N*)BiX_2_** when excited at 375 nm at r.t. under ambient conditions.

In the solid state at r.t., and under ambient conditions, complexes **(*NC^R^N*)BiX_2_** (R = Me, OMe; X = Cl, I) phosphoresce with quantum yields of up to 1.3% for **(*NC^Me^N*)BiCl_2_**. When compared to the frozen solvent matrices, the emissions are even further red‐shifted, peaking in the range of 612‐710 nm and extending into the NIR (see Figure 3 and Figure S60). In line with the above reasoning, the red‐shifts are likely the result of enhanced intermolecular interactions in the solid state.^[^ ^37^ ^]^ Complexes **(*NC^NMe2^N*)BiX_2_** (X = Cl, I) are nonemissive under these conditions.

### 2.4 Electrochemistry

**Figure 4:**
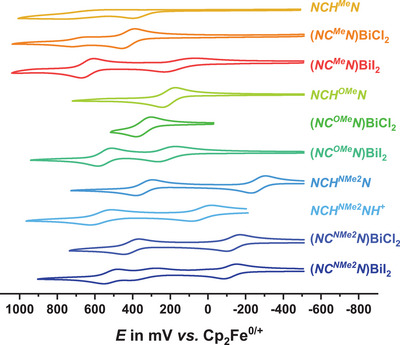
Comparison of the cyclic voltammograms of the complexes **(*NC^R^N*)BiX_2_** and the proligands ***NCH^R^N*** (in 0.1 M *^n^* Bu_4_N^+^ [BAr^F24^]^−^/CH_2_Cl_2_ at *v* = 100 mV/s, and at r.t.). Potentials are referenced to the ferrocene/ferrocenium redox standard (*E*_1/2_ (FcH/FcH^+^) = 0.000 V).

Their electron‐rich triarylamine moieties render the proligands ***NCH^R^N* and** the associated pincer Bi complexes redox‐active. Cyclic and square wave voltammograms in dichloromethane, with 0.1 M tetrabutylammonium tetrakis[3,5‐bis(trifluoromethyl)‐phenyl]borate (*^n^* Bu_4_N^+^ [BAr^F24^]^−^) as the supporting electrolyte, show that all compounds are oxidized in one to three consecutive one‐electron steps (see Figure 4 and Figures S94 to S102). Except for the proligand ***NCH^Me^N***, all oxidations are chemically reversible. This sets them apart from the related pincer complexes **(*p*‐R_2_N‐C_6_H_4_‐CH = CH‐*NCN*)PtCl** (R = Ph, Me), for which only irreversible oxidation of Pt(II) to Pt(IV) was reported.^[^ ^82^ ^]^ Pertinent data are collected in Table 2. As expected, the oxidation potentials respond to the donor qualities of the *p*‐substituents at the diarylamine entity, with the ordering NMe_2_ < OMe < Me. Half‐wave potential shifts for corresponding oxidations of the Me‐ and NMe_2_‐congeners amount to 480 mV for the proligands ***NC^R^HN*** and to 555 mV for complexes **(*NC^R^N*)BiCl_2_**. This points to pincer‐based oxidation.

**Table 2:** Electrochemical data for ***NCHRN*,** **(*NC^R^N*)BiX_2_**, and **(*NCN*)BiX_2_** measured in CH_2_Cl_2_ at r.t.

	*E*_1/2_^0/+^ [Δ*E*_p_]^[a]^	*E*_1/2_^+/2+^ [Δ*E*_p_]^[a]^	*E*_1/2_^2+/3+^ [Δ*E*_p_]^[a]^
***NCH^Me^N***	360^[b]^	670^[b]^	‐
**(*NC^Me^N*)BiCl_2_**	420 (70)	685 (75)	‐
**(*NC^Me^N*)BiI_2_**	150 (150)	640 (65)	‐
***NCH^OMe^N***	205 (70)	‐	‐
**(*NC^OMe^N*)BiCl_2_**	340 (80)	‐	‐
**(*NC^OMe^N*)BiI_2_**	215 (70)	550 (95)	‐
***NCH^NMe2^N***	−270 (75)	340 (90)	‐
***NCH^Me^NH^+^***	30 (100)	565 (110)	
**(*NC^NMe2^N*)BiCl_2_**	−135 (80)	405 (70)	‐
**(*NC^NMe2^N*)BiI_2_**	−120 (70)	340 (140)	515 (105)
**(*NCN*)BiCl_2_**	‐	‐	‐
**(*NCN*)BiI_2_**	255 (105)	‐	‐

^[a]^ All potentials are given in mV (±5 mV) with *^n^* Bu_4_N^+^ [BAr^F24^]^−^ (0.1 mM) as the supporting electrolyte in CH_2_Cl_2_ versus the ferrocene/ferrocenium standard (*E*_1/2_ (FcH/FcH^+^) = 0.000 V).

^[b]^ Peak potential.

For compounds of the same (pro)ligand, the half‐wave potential *E*_1/2_ for corresponding redox steps increases in the order ***NC^R^HN*** *<* **(*NC^R^N*)BiCl_2_** < **(*NC^R^N*)BiI_2_**. Electron withdrawal by the Lewis‐acidic BiX_2_ entity thus outweighs the negative charge at the cyclometalating phenyl ring. The anodic shift of the ligand‐based oxidations is however less pronounced than that resulting from protonation (see Figure 4 and Table 2), as probed at the example of cation [***NCH^NMe2^NH***]^+^, which was prepared by reacting the proligand ***NCH^NMe2^N*** with one equivalent of Brookhart´s acid [H(OEt_2_)]^+^ [B(C_6_H_3_(CF_3_)_2_‐3,5)_4_]^−^.^[^ ^86^ ^]^

Voltammograms of the iodido complexes feature an additional, quasireversible oxidation wave that has no equivalent in the proligands or the BiCl_2_ congeners. For the complexes with the less strongly donating *p*‐tolyl or *p*‐anisyl substituents at the diarylamino pendant, this process accounts for the first oxidation, whereas it appears in between the first and the third oxidations in **(*NC^NMe2^N*)BiI_2_**. Nonidealities of this wave are due to sluggish electron transfer kinetics, as revealed by the considerably larger peak potential splittings Δ*E*_p_ between the reverse and the forward peaks in cyclic voltammetry, a further broadening of the corresponding wave as the sweep rate *v* is increased, and the distinctly larger width at half‐height of the respective square‐wave peak as compared to the other processes (see also Figure S102).

Moreover, the complex **(*NCN*)BiI_2_**,^[^ ^76^ ^]^ while lacking the NAr_2_ donor constituent at the pincer ligand, still undergoes a quasireversible oxidation (see Figure S105) at a similar potential as the first oxidation of **(*NC^R^N*)BiI_2_** (R = Me, OMe), or the second oxidation of **(*NC^NMe2^N*)BiI_2_** (see Figure S106). No such wave is found for the corresponding chlorido complex **(*NCN*)BiCl_2_** (see Figure S107). DFT calculations on **(*NCN*)BiI_2_** and its associated radical cation (see Figure S41) indicate that the primary oxidation site is the BiI_2_ entity and that this process involves a significant structural change at the I‐Bi‐I unit. Hence, the Bi‐I bond lengths shorten from 3.071 Å to 2.911 Å while the bond angle I‐Bi‐I is compressed from 173.6° to 147.1°. In **(*NC^NMe2^N*)BiI_2_**, the strong NMe_2_ donors however direct the first oxidation to the diarylamine pendant. The anodic shift of *E*_1/2_ for BiI_2_ oxidation in **(*NC^NMe2^N*)BiI_2_** with respect to the other iodido complexes is thus the result of a decreased donor capacity of the already oxidized pincer ligand and of electrostatic repulsion (see Figure S106) between the positive charges.

The notion of a triarylamine‐based first oxidation in both complexes **(*NC^NMe2^N*)BiX_2_** is further supported by NMR and EPR spectroscopy. Owing to the low half‐wave potential, oxidation already commences when their aerated solutions in CD_2_Cl_2_ are irradiated for several minutes with 365 nm light (10 W) under ambient conditions (see Figures S21 and S22), similar to what has been observed for single crystals or films of tris(*p*‐tolyl)amine.^[^ ^36^ ^]^ This is accompanied by a continuous broadening of all proton resonances, except for those of the CH_2_NMe_2_ chelate arms, up to the point of their disappearance and a change of color from beige‐orange to green. In parallel, the oxidized proligand **[*NCH^NMe2^N*]*^+^*** and its complexes give rise to the characteristic EPR signal of an aminium species at *g* = 1.993 for **[*NCH^NMe2^N*]*^+^***, or at *g* = 1.992 for complexes **[(*NC^NMe2^N*)BiX_2_]^+^** (see Figure S108). The EPR spectrum obtained from electrochemical in situ oxidation of **(*NC^NMe2^N*)BiI_2_** is identical within error margins to that recorded on a chemically oxidized sample (see Figure S109).The addition of zinc dust as a reducing agent restores the proton NMR resonances and changes the color back to the original one. In contrast, **[(*NCN*)BiI_2_]^+^**, generated by oxidizing the neutral complex with NO^+^ [SbF_6_]^−^, is EPR silent even at 88 K.

### 2.5 Investigations of the Oxidized Forms

**Figure 5:**
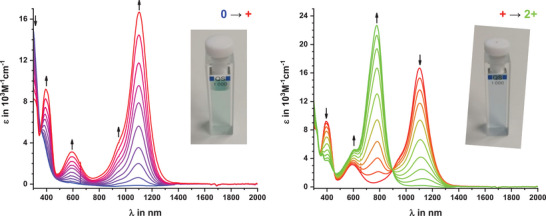
Changes of the UV/vis/NIR spectra during electrochemical oxidation of **(*NC^NMe2^N*)BiCl_2_**, to its radical cation (left) and during further oxidation to the corresponding dication (right) measured in CH_2_Cl_2_/*^n^* Bu_4_N^+^ [BAr^F24^]^−^ (0.1 M) at r.t.

Based on the low oxidation potentials of particularly the **(*NC^NMe2^N*)BiX_2_** complexes and the overall chemical reversibility of the individual oxidations, we mused that their associated one‐electron oxidized radical cations, and even the two‐ or three‐electron oxidized di‐ and trications might be amenable for further studies. As a first test, we resorted to spectroelectrochemical (SEC) experiments in an optically transparent thin‐layer electrolysis (OTTLE) cell,^[^ ^87^ ^]^ where we monitored the changes in the UV/vis/NIR spectra concomitant with the stepwise oxidations. Figure 5 shows the results of such an experiment on **(*NC^NMe2^N*)BiCl_2_**, while those for **(*NC^NMe2^N*)BiI_2_** and the proligand ***NCH^NMe2^N*** are documented in Figures S31 and S32.

The deep green radical monocations are characterized by an intense NIR absorption at ca. 1100 nm and additional, weaker bands in the vis at ca. 600 and in the near UV at ca. 400 nm. Pertinent data are compiled in Table S1. We note that the radical cations of the proligand and the BiX_2_ complexes absorb at even lower energy than the prototypical [N(C_6_H_4_‐4‐NMe_2_)_3_]^+^ aminium radical cation.^[^ ^88^, ^89^ ^]^ According to TD‐DFT calculations (see Figures S42 to S44), the NIR band originates from a π→π* transition that is confined within the [N(C_6_H_4_‐4‐NMe)_2_]^+^ constituent, and with only a small degree of charge transfer to the cyclometalating phenyl ring. This is also borne out by the computed spin density distributions.

The electronic excitation near 600 nm has no equivalent in the parent triarylaminium radical cation [N(C_6_H_4_‐4‐NMe_2_)_3_]^+^. It involves the transfer of electron density from the cyclometalating phenyl ring or, in the case of the iodido complex, the BiI_2_ complex entity, to the open‐shell aminium chromophore (see Figures S42 to S44). In the absence of atmospheric oxygen or light, the radical cations are stable over weeks in CH_2_Cl_2_ solution, with no fading of their color observed.

Further oxidation to the di‐ or trications bleaches the 1100 nm and 400 nm absorption bands, while a new, likewise very intense absorption band grows in at ca. 770 nm. The band at ca. 600 nm remains unaffected (see the right panel of Figure 5). This endows these higher oxidized forms with a deep blue color. TD‐DFT calculations indicate that **[*NCH^NMe2^N*]^2+^** and **[(*NC^NMe2^N*)BiCl_2_]^2+^** prefer the closed‐shell singlet state by ca. 30 kJ/mol over the triplet state (see Figures S45 and S46). They however fail to reproduce the experimental ordering of redox steps for **(*NC^NMe2^N*)BiI_2_** in that the second oxidation is predicted to also involve the triarylamine, with only the third oxidation occurring at the BiI_2_ entity (see Figures S47 to 49). Computed differences in calculated electronic spectra between **[(*NC^NMe2^N*)BiCl_2_]^2+^** and **[(*NC^NMe2^N*)BiCl_2_]^3+^** are only minor and predict no shift of the NIR band (see Figure S50).

**Figure 6:**
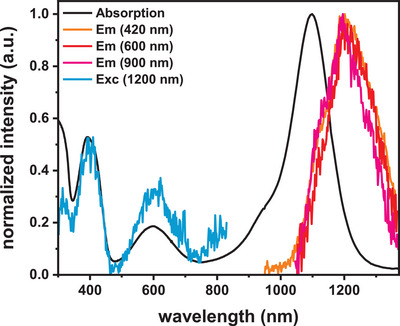
PL data of **(*NC^NMe2^N*)BiCl_2_^+^** at r.t. in degassed CH_2_Cl_2_. Absorption spectra are depicted in black, emission spectra in orange, red, and pink, and excitation spectra in blue.

PL measurements on oxidized **[*NCH^NMe2^N*]^n+^** (n = 1, 2) and **[(*NC^NMe2^N*)BiX_2_]^n+^** (X = Cl, n = 1,2; X = I, n = 1, 3) were conducted in degassed CH_2_Cl_2_ at r.t. (see Figure 6, Figures S92 and S93 and Table S1). For these studies, the respective cations were prepared by chemically oxidizing the neutral compounds with the appropriate amounts of NO^+^ [SbF_6_]^−^. UV/vis/NIR absorption spectra of the so generated species match very well with those collected during SEC experiments (see Figure S34). All three radical monocations show weak doublet emission in the NIR region at ca. 1250 nm. Their low intensities thwarted our attempts to determine lifetimes or quantum yields. Excitation spectra detected at 1250 nm nevertheless retrace the features of the absorption spectra of the radical cations, thereby proving the origin of these emissions. The higher oxidized forms proved to be nonemissive at r.t. in degassed CH_2_Cl_2_ and at 77 K in a CH_2_Cl_2_/toluene glass. The higher oxidized compounds are considerably less stable than the monocations with their solutions in MeTHF decolorizing within only a few minutes. Improved stability is observed in CH_2_Cl_2_ or CH_2_Cl_2_/toluene, where their coloration persists over a couple of hours.

### 2.6 Summary and Conclusions

In conclusion, we have prepared six new triarylamine‐modified bismuth pincer complexes and investigated their electrochemical and their versatile photophysical properties. While the proligands are strongly fluorescent, the complexes derived from the *p*‐tolyl‐ and the *p*‐anisyl‐substituted ligands phosphoresce at 77 K in frozen solvent matrices and in the solid state at r.t. This highlights the role of the bismuth ion to promote intersystem crossing. Notably, the oxidized forms of the dimethylamino‐substituted complexes display near‐infrared (NIR) fluorescence. This renders diarylamine‐modified pincer complexes of bismuth emissive in their singlet, doublet, and triplet states. Our findings demonstrate the viability of bismuth complexes as alternatives to more expensive transition‐metal‐based phosphors. Remaining challenges are to further modify such complexes as to render ISC from their excited *T*_1_ state to the *S*_0_ state so efficient as to achieve room‐temperature phosphorescence emission, which is highly desirable for wider applications.

## 3 Experimental Details

### General Procedures

All syntheses were performed under dinitrogen atmosphere and protection from light, using standard Schlenk techniques. No uncommon hazards are involved, apart from those concomitant with work under cryogenic conditions (−70 °C, cooling baths with *iso*‐propanol and dry ice; appropriate protective clothing should be worn). **(*NCN*)BiCl_2_**,^[^ ^76^ ^]^ **(*NCN*)BiI_2_**,^[^ ^76^ ^]^ ***NCH^Me^N***,^[^ ^37^ ^]^ **(*NC^Me^N*)BiCl_2_**,^[^ ^37^ ^]^ and 1‐bromo‐3,5‐bis(dimethylaminomethyl)‐benzene^[^ ^90^ ^]^ were prepared according to literature procedures. ^1^H‐ and ^13^C‐NMR spectra of all new proligands and Bi complexes can be found in the Supporting Information.

### NMR Spectroscopy

^1^H‐ (400 MHz) and ^13^C{^1^H}‐NMR (101 MHz) spectra were recorded in CD_2_Cl_2_, CDCl_3_, or C_6_D_6_ at 300 K using a Bruker Avance III 400 spectrometer. NMR spectra were referenced to residual protonated solvent (^1^H) or the solvent signal itself (^13^C). The assignment of signals is based on 2D spectra.

### Mass Spectrometry

Mass spectra of the compounds were recorded in the positive ion mode on an ESI‐calibrated LTQ Orbitrap Velos Spectrometer (flow rate: 0.5 mL min^−1^, source temperature: 320 °C, capillary voltages 3500 V) with the direct injection of their CH_2_Cl_2_ solutions.

### UV/vis/NIR Spectroscopy

UV/vis/NIR spectra of CH_2_Cl_2_, THF, and MeTHF solutions of complexes, the proligands, and the various oxidized forms of ***NCH^NMe2^N*** and **(*NC^NMe2^N*)BiX_2_** were recorded on a TIDAS fiber optic diode array spectrometer from J&M in HELLMA quartz cuvettes with 1.0 cm optical path lengths.

### TD‐DFT Calculations

The ground state electronic structures of the full models of the complexes **(*NC^R^N*)BiX_2_** as well as the oxidized forms of the *N*,*N*‐bis(4‐dimethylamino) derivatives were calculated by density functional theory (DFT) methods using the Gaussian 16 program packages.^[^ ^91^ ^]^ Open shell systems were calculated by the unrestricted Kohn‐Sham approach (UKS). Geometry optimization was performed in solvent media. Stationary states were checked by vibrational analysis for the absence of imaginary frequencies. Solvent effects were described by the SMD variation of IEFPCM implemented in the Gaussian program package with standard parameters for THF and CH_2_Cl_2_.^[^ ^92^ ^]^ The fully relativistic small‐core multiconfiguration Dirac Hartree‐Fock‐adjusted pseudopotentials and the corresponding optimized set of basis functions (ECP60MDF)^[^ ^93^ ^]^ for Bi, LAN2DZ^[^ ^94^ ^]^ for iodine, and 6–31G(d) polarized double‐ζ basis sets^[^ ^95^ ^]^ for the remaining atoms were employed together with the Perdew, Burke, Ernzerhof exchange and correlation functional PBE0.^[^ ^96^, ^97^ ^]^ The GaussSum program package was used to analyze the results. ^[^ ^98^ ^]^ The latter were visualized with the Avogadro program package.^[^ ^99^ ^]^ Graphical representations of molecular orbitals were generated with the help of GNU Parallel^[^ ^100^ ^]^ and plotted using the vmd program package^[^ ^101^ ^]^ in combination with POV‐Ray.^[^ ^102^ ^]^

### Photoluminescence

Luminescence spectra and lifetimes in THF, 2‐MeTHF and CH_2_Cl_2_ solutions were measured on a PicoQuant FluoTime 300 spectrometer. Absolute quantum yields were determined with an integrating sphere within the FluoTime 300 spectrometer. Solutions were deaerated through three cycles of freeze‐pump‐thaw. Solid‐state samples were prepared by drop‐casting 10 mM CH_2_Cl_2_ solutions of the complexes or proligands onto 1 cm × 1 cm quartz plates. Absolute quantum yields at 77 K were measured on the Absolute PL quantum yield spectrometer C9920‐02G from Hamamatsu with the A11238‐02 Dewar for low temperatures.

### Cyclic voltammetry

All electrochemical experiments were performed in a custom‐made cylindrical vacuum‐tight one‐compartment cell. A coiled Pt wire and a coiled Ag wire, serving as the counter and reference electrodes, were sealed into glass capillaries and introduced via Quickfit screws to ports located at opposite sides of the cell. A platinum electrode was used as the working electrode and inserted through the top port via a Teflon screw cap with a suitable fitting. The working electrode was polished first with 1 µm and then 0.25 µm diamond pastes (Buehler‐Wirtz) before measurements. The cell can be attached to a conventional Schlenk line via a side arm equipped with a Teflon screw valve and allows experiments to be performed under an atmosphere of argon with approximately 5 mL of analyte solution. CH_2_Cl_2_ / *^n^* Bu_4_N^+^ [BAr^F24^]^−^ (0.1 M) was used as the supporting electrolyte. Referencing was done by adding an appropriate amount of decamethylferrocene (Cp*_2_Fe) as an internal standard to the analyte solution after all data of interest had been collected. Representative sets of scans were repeated with the added standard. The final referencing was done against the ferrocene/ferrocenium (Cp_2_Fe^0/+^) redox couple with *E*_1/2_ (Cp*_2_Fe^0/+^) = −540 mV versus Cp_2_Fe^0/+^ for the electrolyte used. Electrochemical data were acquired with a computer‐controlled BASi potentiostat.

### Spectroelectrochemical measurements

The OTTLE cell for spectroelectrochemical experiments was also self‐constructed and follows the design of Hartl et al.^[^ ^87^ ^]^ It comprises of Pt‐mesh working and counter electrodes and a thin silver plate as a pseudoreference electrode. The electrodes are sandwiched in between two CaF_2_ windows of a conventional liquid IR cell. The working electrode was positioned in the center of the spectrometer beam. The required potential was applied by connecting the cell leads to a Wenking POS 2 potentiostat from Bank Elektronik‐Intelligent Controls GmbH. As supporting electrolyte, a 0.1 M solution of *^n^* Bu_4_N^+^ [BAr^F24^]^−^ in CH_2_Cl_2_ was used. UV/vis/NIR spectra were acquired on a TIDAS fiber optic diode‐array spectrophotometer (combined MCS UV/vis and PGS NIR instrumentation) from j&m Analytik AG.

### EPR spectroscopy

EPR spectra were obtained using an X‐Band tabletop MiniScope MS 400 spectrometer by Magnettech GmbH. The software MiniScopeControl V2.5.1 was used for data evaluation. Samples of the oxidized ligand and complexes were generated by using NO^+^ [SbF_6_]^−^ as the oxidant.

### Synthetic protocols

**(*NC^Me^N*)BiI_2_. (*NC^Me^N*)BiCl_2_** (0.100 g, 0.150 mmol, 1.00 eq) and KI (0.122 g, 0.735 mmol, 4.90 eq) were dissolved in a degassed solvent mixture of CH_2_Cl_2_ (5 mL), EtOH (3 mL) and H_2_O (2 mL). The mixture was stirred at r.t. for 24 hours. The phases were separated, and the organic phase was dried *in vacuo*. **(*NC^Me^N*)BiI_2_** was obtained as an orange solid (0.102 g, 0.120 mmol, 80% yield). ^1^H‐NMR (CD_2_Cl_2_, 400 MHz) δ [ppm]: 7.16 (s, 2H, H^4^), 7.14 (d, ^3^ *J*_HH_ = 8.2 Hz, 4H, H^3^), 7.06 (d, ^3^ *J*_HH_ = 8.2 Hz, 4H, H^2^), 4.35 (s, 4H, H^5^), 3.07 (s, 12H, H^6^), 2.33 (s, 6H, H^1^) (for atomic numbering, see the Supporting Information). ^13^C‐NMR (CD_2_Cl_2_, 101 MHz) δ [ppm]: 189.9 (C^e^), 155.3 (C^d^), 150.3 (C^c^), 144.6 (C^b^), 134.8 (C^a^), 130.6 (C^3^), 126.6 (C^2^), 119.8 (C^4^), 71.4 (C^5^), 51.2 (C^6^), 21.0 (C^1^). HR ESI‐MS (*m*/*z*) = 850.0615, 722.14656; calcd. for C_26_H_33_BiI_2_N_3_^+^ = 850.0562; calcd. for C_26_H_32_BiIN_3_^+^ = 722.1439.

***NCH^OMe^N***. 1‐Bromo‐3,5‐bis(dimethylaminomethyl)benzene (0.980 g, 3.61 mmol, 1.00 eq), bis(*p*‐anisyl)amine (0.829 g, 3.61 mmol, 1.00 eq), NaO*^t^* Bu (1.04 g, 10.8 mmol, 3.00 eq), [Pd_2_(dba)_3_]·CHCl_3_ (spatula tip) and a 10% solution of P*^t^* Bu_3_ in *n‐*hexane (1.59 mL, 0.542 mmol, 0.150 eq) were dissolved in toluene (10 mL). The mixture was heated to reflux for 3 days, filtered through a pad of Celite, and the solvent was removed *in vacuo*. The crude product was dissolved in *n*‐pentane and the insoluble materials were filtered off. After removing the solvent *in vacuo*, the product was purified by column chromatography (*n*‐pentane/CH_2_Cl_2_ 3/1‐0/1 with 3% Et_3_N). ***NCH^OMe^N*** was obtained as a light brown solid (1.35 g, 3.21 mmol, 89% yield). ^1^H‐NMR (CD_2_Cl_2_, 400 MHz) δ [ppm]: 7.00 (d, ^3^ *J*_HH_ = 9.1 Hz, 4H, H^3^), 6.81 (d, ^3^ *J*_HH_ = 9.1 Hz, 4H, H^2^), 6.76 (s, 1H, H^7^), 6.75 (s, 2H, H^4^), 3.78 (s, 6H, H^1^), 3.26 (s, 4H, H^5^), 2.15 (s, 12H, H^6^) (for atomic numbering, see the Supporting Information). ^13^C‐NMR (CD_2_Cl_2_, 101 MHz) δ [ppm]: 156.1 (C^a^), 149.0 (C^c^), 141.8 (C^b^), 140.4 (C^d^), 126.5 (C^3^), 122.9 (C^7^), 120.9 (C^4^), 120.9 (C^2^), 64.6 (C^5^), 55.8 (C^1^), 45.5 (C^6^). HR ESI‐MS (*m*/*z*) = 420.2657; calcd. for C_26_H_34_N_3_O_2_^+^ = 420.2646.

***NCH^NMe2^N***. 1‐Bromo‐3,5‐bis(dimethylaminomethyl)benzene (0.980 g, 3.61 mmol, 1.00 eq), 4,4′‐bis(*p*‐dimethylaminophenyl)‐amine (0.922 g, 3.61 mmol, 1.00 eq), NaO*^t^* Bu (1.04 g, 10.8 mmol, 3.00 eq), [Pd_2_(dba)_3_]·CHCl_3_ (spatula tip) and a 10% solution of P*^t^* Bu_3_ in *n‐*hexane (1.59 mL, 0.542 mmol, 0.150 eq) were dissolved in toluene (10 mL). The mixture was heated to reflux for 3 days, filtered through a pad of Celite, and the solvent was removed *in vacuo*. The crude product was dissolved in *n*‐pentane and the insoluble materials were filtered off. After removing the solvent *in vacuo*, the product was purified by column chromatography (*n*‐pentane/CH_2_Cl_2_ 3/1‐0/1 with 3% Et_3_N). ***NCH^NMe2^N*** was obtained as a brownish solid (1.49 g, 3.33 mmol, 92% yield). ^1^H‐NMR (CD_2_Cl_2_, 400 MHz) δ [ppm]: 6.98 (d, ^3^ *J*_HH_ = 9.1 Hz, 4H, H^3^), 6.69 (d, ^3^ *J*_HH_ = 9.1 Hz, 4H, H^2^), 6.69 (s, 3H, H^4,^ ^7^), 3.25 (s, 4H, H^5^), 2.92 (s, 12H, H^1^), 2.16 (s, 12H, H^6^) (for atomic numbering, see the Supporting Information). ^13^C‐NMR (CD_2_Cl_2_, 101 MHz) δ [ppm]: 149.6 (C^c^), 147.7 (C^a^), 140.1 (C^d^), 138.4 (C^b^), 126.9 (C^3^), 121.5 (C^7^), 119.2 (C^4^), 113.9 (C^2^), 64.7 (C^5^), 45.5 (C^6^), 41.2 (C^1^). HR ESI‐MS (*m*/*z*) = 446.3286, 445.3212; calcd. for C_28_H_40_N_5_^+^ = 446.3278; calcd. for C_28_H_39_N_5_^+^ = 445.3200.

***(NC^OMe^N)BiCl_2_*. *NCH^OMe^N*** (0.600 g, 1.43 mmol, 1.00 eq) was dissolved in *n‐*hexane abs. (5 mL) and a 1.6 M solution of *n‐*BuLi (0.89 mL, 1.43 mmol, 1.00 eq) was added. The reaction mixture was heated to reflux overnight. The solvent was removed *in vacuo*. The remaining solid was dissolved in 20 mL of Et_2_O and slowly cannulated into a cooled ‐78 °C solution of BiCl_3_ (0.451 g, 1.43 mmol, 1.00 eq) Et_2_O (15 mL) cold. After stirring for 1 hour the reaction mixture was warmed to r.t. and stirred overnight. The solvent was removed *in vacuo*. The solid was extracted with CH_2_Cl_2_ (4×20 mL) and the filtered solution was stripped of the solvent *in vacuo*. The remaining solid was washed with *n*‐hexane (6×15 mL), MeCN (2×20 mL) and then dried *in vacuo*. The title complex was obtained as a beige solid (0.771 g, 1.10 mmol) in a yield of 77%. ^1^H‐NMR (CD_2_Cl_2_, 400 MHz) δ [ppm]: 7.12 (d, ^3^ *J*_HH_ = 8.9 Hz, 4H, H^3^), 7.04 (s, 2H, H^4^), 6.87 (d, ^3^ *J*_HH_ = 8.9 Hz, 4H, H^2^), 4.26 (s, 4H, H^5^), 3.79 (s, 6H, H^1^), 2.86 (s, 12H, H^6^) (for atomic numbering, see the Supporting Information). ^13^C‐NMR (CD_2_Cl_2_, 101 MHz) δ [ppm]: 202.7 (C^e^), 157.3 (C^a^), 154.3 (C^d^), 150.7 (C^c^), 140.2 (C^b^), 128.3 (C^3^), 118.0 (C^4^), 115.3 (C^2^), 68.8 (C^5^), 55.9 (C^1^), 47.7 (C^6^). HR ESI‐MS (*m*/*z*) = 697.1684, calcd. for C_26_H_32_BiCl_2_N_3_O_2_^+^ = 697.1675; 662.1981, calcd. for C_26_H_32_BiClN_3_O_2_^+^ = 662.1981.

**(*NC^OMe^N*)BiI_2_**. The proligand ***NCH^OMe^N*** (1.0 eq., 1.2 mmol, 0.50 g) was dissolved in absolute *n*‐hexane (5 mL). A solution of *n*‐BuLi in *n*‐hexane (1.0 eq., 1.2 mmol, 2.5 M, 0.47 mL) was added. The mixture was kept under reflux at 70 °C for 21 hours. The solvent was removed *in vacuo* and the remaining solid was dissolved in diethyl ether (15 mL). BiI_3_ (1.0 eq., 1.2 mmol, 0.70 g) was dissolved in diethyl ether (7 mL) and the solution was cooled to ‐78 °C and protected from light with an aluminum foil. The ethereal solution of the deprotonated ligand was slowly transferred by cannula into the BiI_3_ solution. The flask was additionally rinsed with diethyl ether (10 mL). The mixture was stirred in the dark at ‐78 °C for 1 hour and was then allowed to warm to r.t. and allowed to stir for 3 days. Then, the solvent was removed *in vacuo*. The solid was extracted into dry CH_2_Cl_2_ (20 mL) and the insoluble material was filtered off. After evaporating the solvent *in vacuo*, the residue was washed with *n*‐hexane (30 mL) and acetonitrile (70 mL) and then dried *in vacuo*. **(*NC^OMe^N*)BiI_2_** was obtained as a yellow solid (0.20 g, 0.22 mmol) in a yield of 19%. ^1^H‐NMR (CD_2_Cl_2_, 400 MHz) δ [ppm]: 7.14 (d, ^3^ *J*_HH_ = 8.9 Hz, 4H, H^3^), 7.05 (d, ^3^ *J*_HH_ = 8.9 Hz, 4H, H^2^), 6.89 (s, 2H, H^4^), 4.33 (s, 4H, H^5^), 3.80 (s, 6H, H^1^), 3.07 (s, 12H, H^6^) (for atomic numbering, see the Supporting Information). ^13^C‐NMR (CD_2_Cl_2_, 101 MHz) δ [ppm]: 189.2 (C^e^), 157.5 (C^a^), 155.3 (C^d^), 151.2 (C^c^), 139.9 (C^b^), 128.6 (C^3^), 117.9 (C^4^), 115.4 (C^2^), 71.5 (C^5^), 55.9 (C^1^), 51.2 (C^6^). HR ESI‐MS 882.0494, calcd. for C_26_H_33_BiI_2_N_3_O_2_^+^ = 882.0460; 754.1312, calcd. for C_26_H_32_BiIN_3_O_2_^+^ = 754.1338.

**(*NC^NMe2^N*)BiCl_2_. *NCH^NMe2^N*** (0.700 g, 1.57 mmol, 1.00 eq) was dissolved in *n‐*hexane (5 mL) and 0.98 mL (1.57 mmol, 1.00 eq) of a 1.6 M solution of *n‐*BuLi were added. The reaction mixture was heated to reflux overnight. The solvent was removed *in vacuo*. The remaining solid was dissolved with 20 mL Et_2_O and slowly transferred by cannula into a ‐78 °C cold Et_2_O (15 mL) solution of BiCl_3_ (0.495 g, 1.57 mmol, 1.00 eq). After stirring for 1 hour the reaction mixture was warmed to r.t. and left stirring overnight. The solvent was removed *in vacuo* and the remaining solid was extracted with CH_2_Cl_2_ (4×20 mL) and the solution was filtered into another Schlenk flask. Afterwards, the solvent was removed *in vacuo*. The remaining solid was washed with *n*‐hexane (6×15 mL) and MeCN (2×20 mL) and was dried *in vacuo*. The title complex was obtained as a beige solid (0.851 g, 1.15 mmol) in a yield of 73%. ^1^H‐NMR (CD_2_Cl_2_, 400 MHz) δ [ppm]: 7.07 (d, ^3^ *J*_HH_ = 8.9 Hz, 4H, H^3^), 6.99 (s, 2H, H^4^), 6.70 (d, ^3^ *J*_HH_ = 8.9 Hz, 4H, H^2^), 4.24 (s, 4H, H^5^), 2.93 (s, 12H, H^1^), 2.86 (s, 12H, H^6^) (for atomic numbering, see the Supporting Information). ^13^C‐NMR (CD_2_Cl_2_, 101 MHz) δ [ppm]: 201.4 (C^e^), 154.3 (C^d^), 151.4 (C^c^), 148.7 (C^a^), 136.4 (C^b^), 128.3 (C^3^), 116.5 (C^4^), 113.9 (C^2^), 68.8 (C^5^), 47.7 (C^6^), 41.0 (C^1^). HR ESI‐MS 723.2328, calcd. for C_28_H_38_BiClN_5_^+^ = 723.2308; 688.2614, calcd. for C_28_H_38_BiClN_5_^+^ = 688.2614; 445.3223, calcd. for C_28_H_38_N_5_^+^ = 445.3205.

**(*NC^NMe2^N*)BiI_2_**. ***NCH^NMe2^N*** (0.45 g, 1.01 mmol, 1.00 eq) was dissolved in *n*‐hexane (5 mL) and mixed with 0.40 mL of a 2.5 M solution of *n*‐BuLi (1.00 mmol, 1.00 eq). The reaction mixture was heated to reflux for 18 hours. The solvent was removed *in vacuo*. The remaining solid was dissolved in 15 mL Et_2_O and was slowly cannula‐transferred into a cold (‐78 °C) solution of BiI_3_ (0.60 g, 1.01 mmol, 1.00 eq) in Et_2_O (15 mL). After stirring in the dark for 1 hour the reaction mixture was warmed to r.t. and stirred for 18 hours. The solvent was removed *in vacuo*. The solid was extracted with dry CH_2_Cl_2_. The solution was filtered, and the solvent was removed *in vacuo*. The remaining solid was washed with *n*‐hexane (20 mL) and then dried *in vacuo*. **(*NC^NMe2^N*)BiI_2_** was obtained as an orange solid (0.24 mg, 0.26 mmol) in a yield of 26%. ^1^H‐NMR (CD_2_Cl_2_, 400 MHz) δ [ppm]: 7.09 (d, ^3^ *J*_HH_ = 9.1 Hz, 4H, H^3^), 7.01 (s, 2H, H^4^), 6.71 (d, ^3^ *J*_HH_ = 9.1 Hz, 4H, H^2^), 4.31 (s, 4H, H^5^), 3.06 (s, 12H, H^6^), 2.93 (s, 12H, H^1^) (for atomic numbering, see the Supporting Information). ^13^C‐NMR (CD_2_Cl_2_, 101 MHz) δ [ppm]: 187.8 (C^e^), 155.3 (C^a^), 151.9 (C^c^), 148.9 (C^b^), 136.1 (C^d^), 128.5 (C^3^), 116.6 (C^4^), 113.9 (C^2^), 71.6 (C^5^), 51.7 (C^6^), 40.9 (C^1^). HR ESI‐MS 907.1047, calcd. for C_28_H_38_BiI_2_N_5_^+^ = 907.1020; 780.1981, calcd. for C_28_H_38_BiIN_5_^+^ = 780.1970.

## Conflict of Interests

The authors declare no conflict of interest.

## Supporting information



Supporting Information

## Data Availability

The data that support the findings of this study are available in the supporting information of this article.
