# Supplementary material for: Singlet, Doublet, and Triplet Emissions of Diarylamine‐Modified Bismuth Pincer Complexes
Source: Chemistry. 2025 Apr 28;31(30):e202500384. doi: 10.1002/chem.202500384 (PMC12117182; doi:10.1002/chem.202500384)
Supplement: Supplementary file 1 — Supporting Information [file CHEM-31-e202500384-s001.pdf]

Supporting Information

Singlet, Doublet, and Triplet Emissions of Diarylamine-Modified Bismuth Pincer Complexes

Marcel Geppert,[a] Michelle Müller,[a] Katharina J. Scherer,[a] Jessica Henzler,[a] and Rainer F. Winter*[a]

[a] Fachbereich Chemie, Universität Konstanz, 78457 Konstanz, Germany.

Table of Contents

NMR Data 2

ESI-MS Data 13

UV/Vis Spectroscopy 20

TD-DFT Calculations 26

Photoluminescence Data 59

Electrochemistry 86

EPR Spectroscopy 99

NMR Data

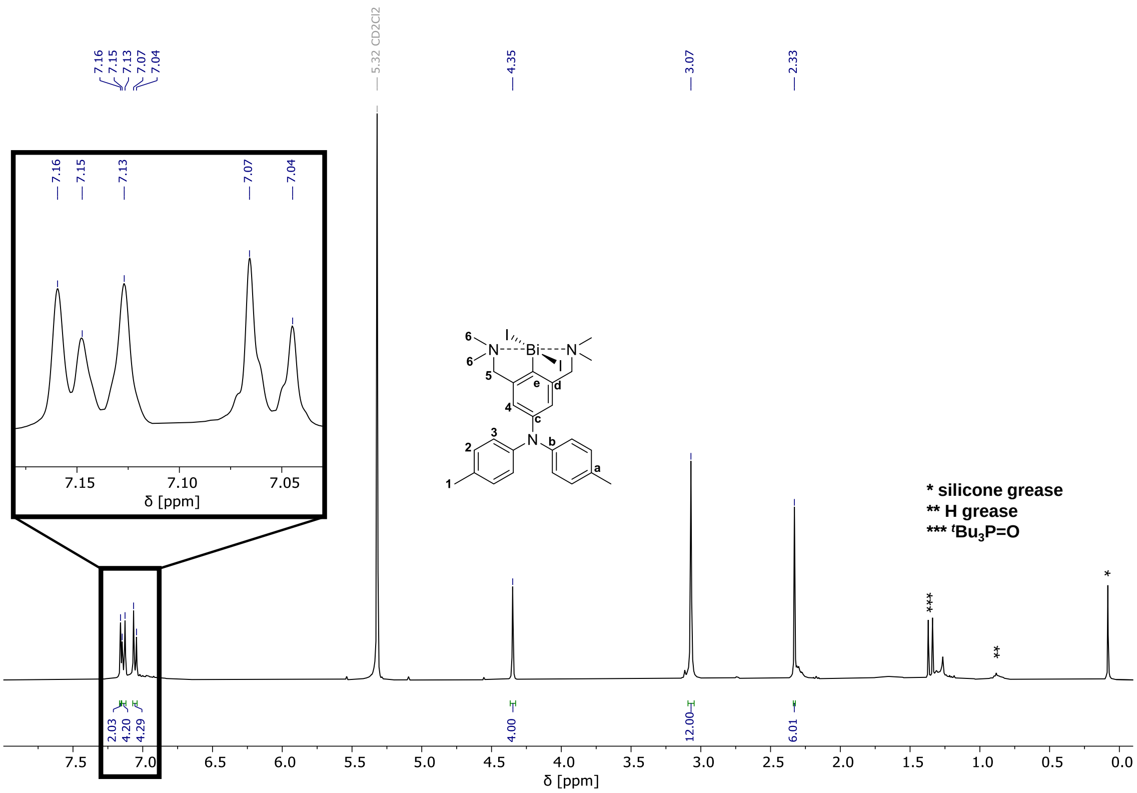

Figure S1. ^{1}H-NMR spectrum of **(*NC^{Me}N*)BiI$_2$** in CD_2Cl_2.

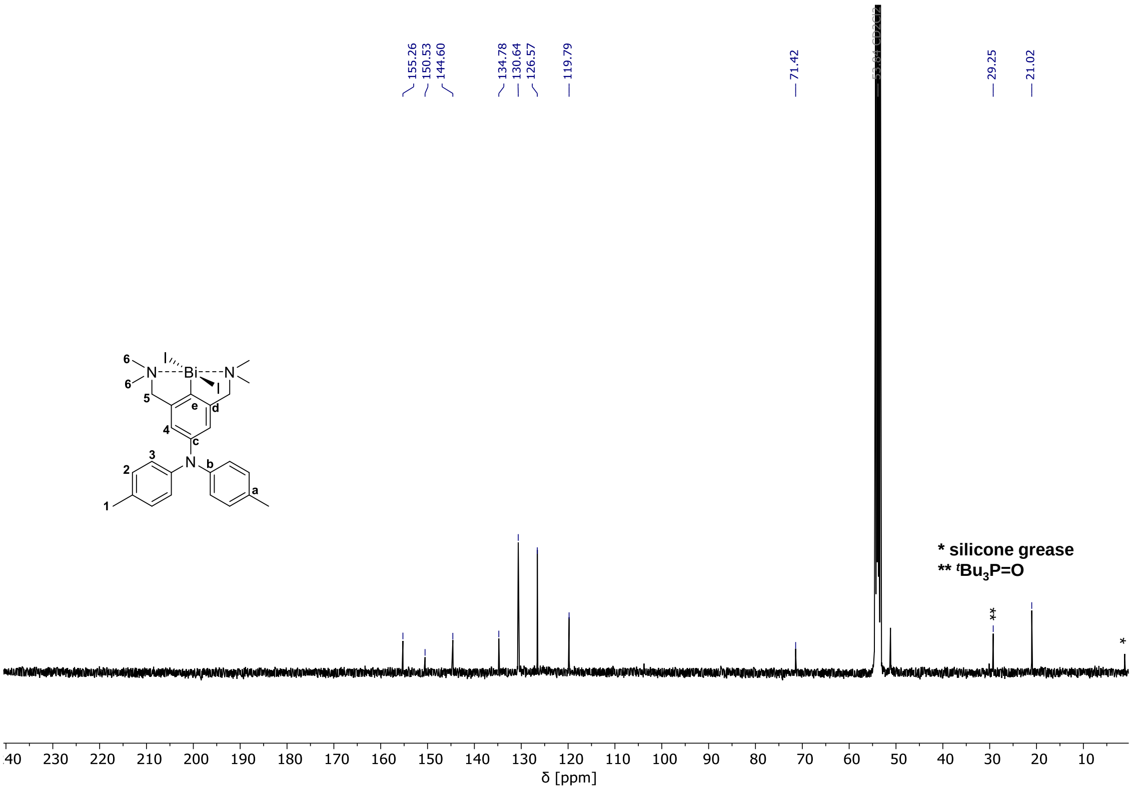

Figure S2. ^{13}C{^{1}H}-NMR spectrum of **(*NC^{Me}N*)BiI$_2$** in CD_2Cl_2.

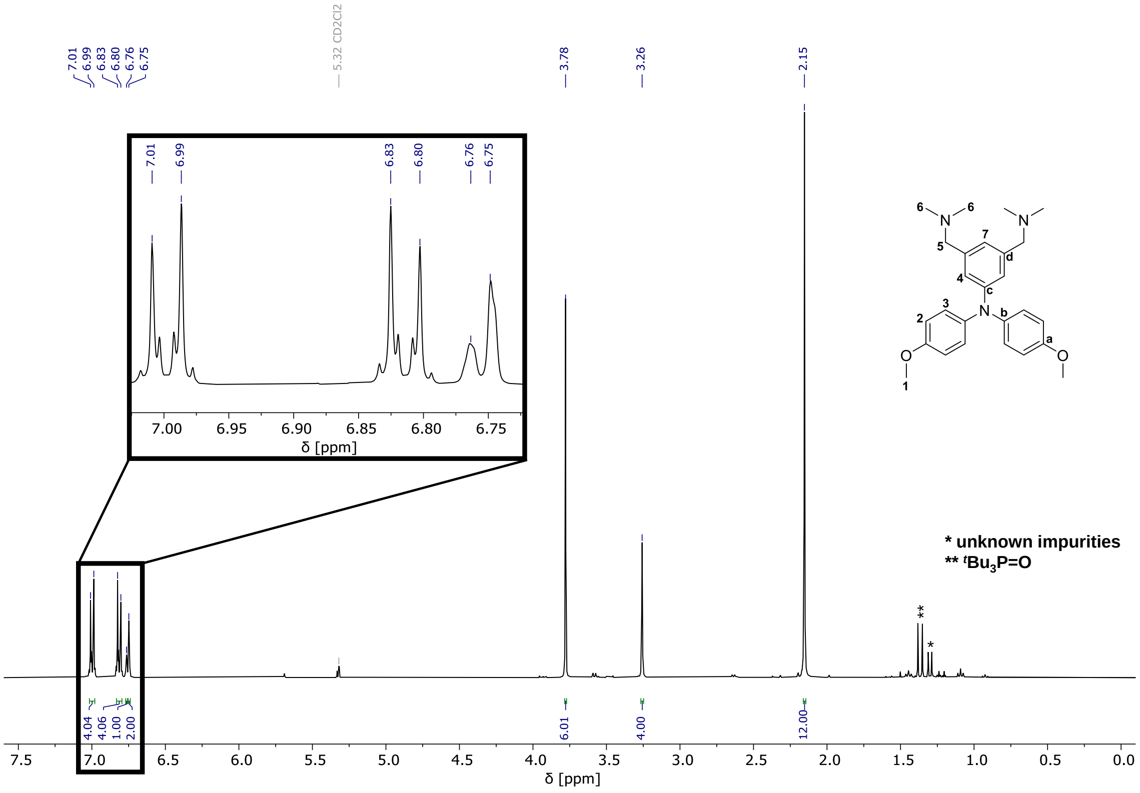

Figure S3. ^{1}H-NMR spectrum of ***NCH^{OMe}N*** in CD_2Cl_2.

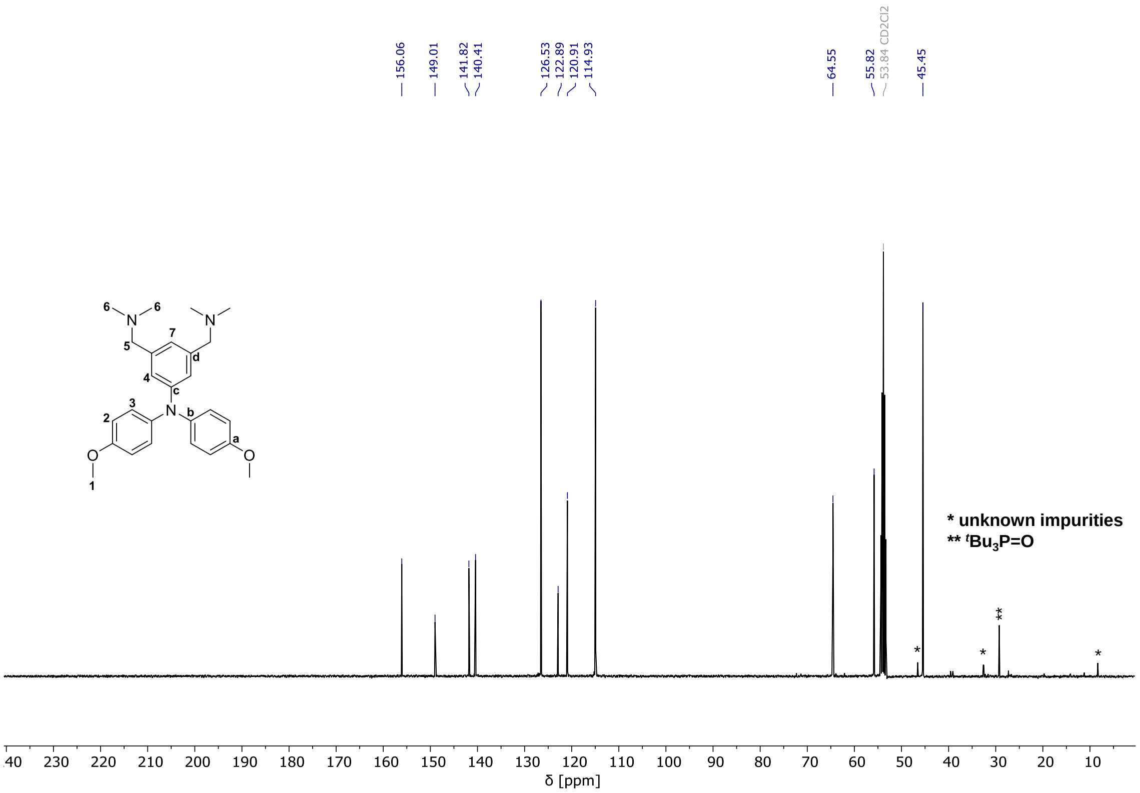

Figure S4. $^{13}C\{^{1}H\}$-NMR spectrum of ***NCH^{OMe}N*** in CD_2Cl_2.

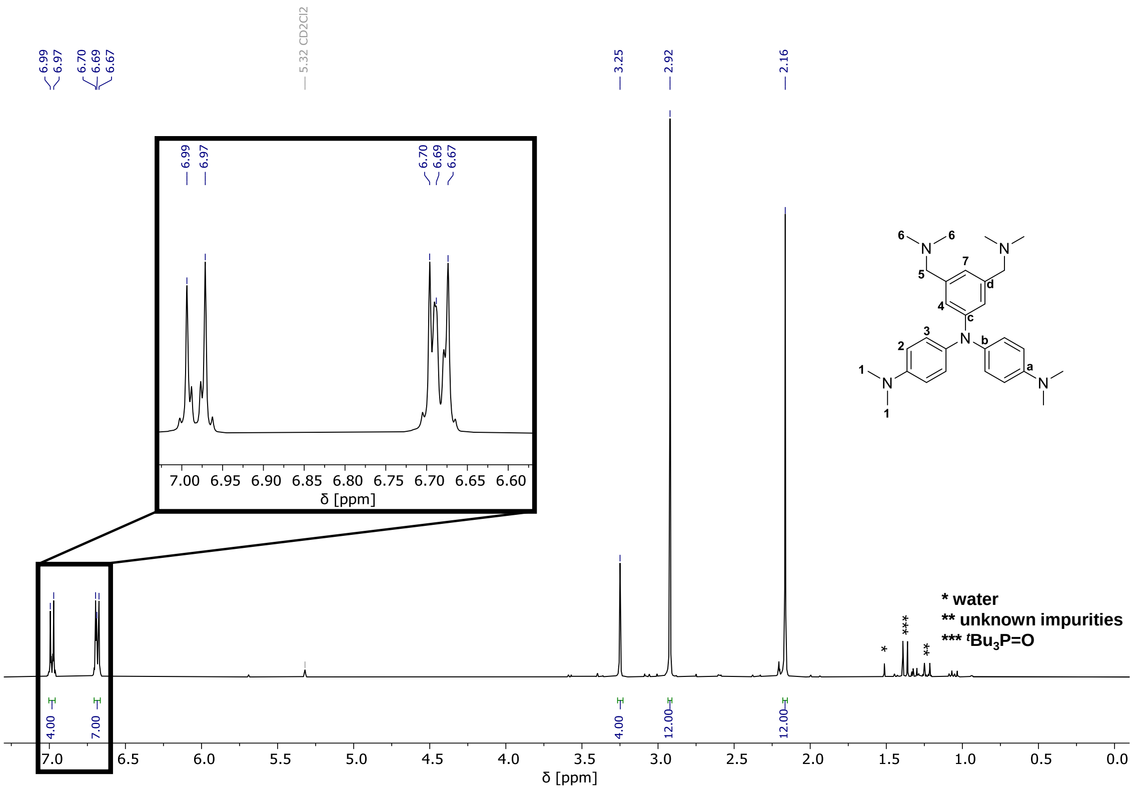

Figure S5. ^{1}H-NMR spectrum of ***NCH^{NMe2}N*** in CD_2Cl_2.

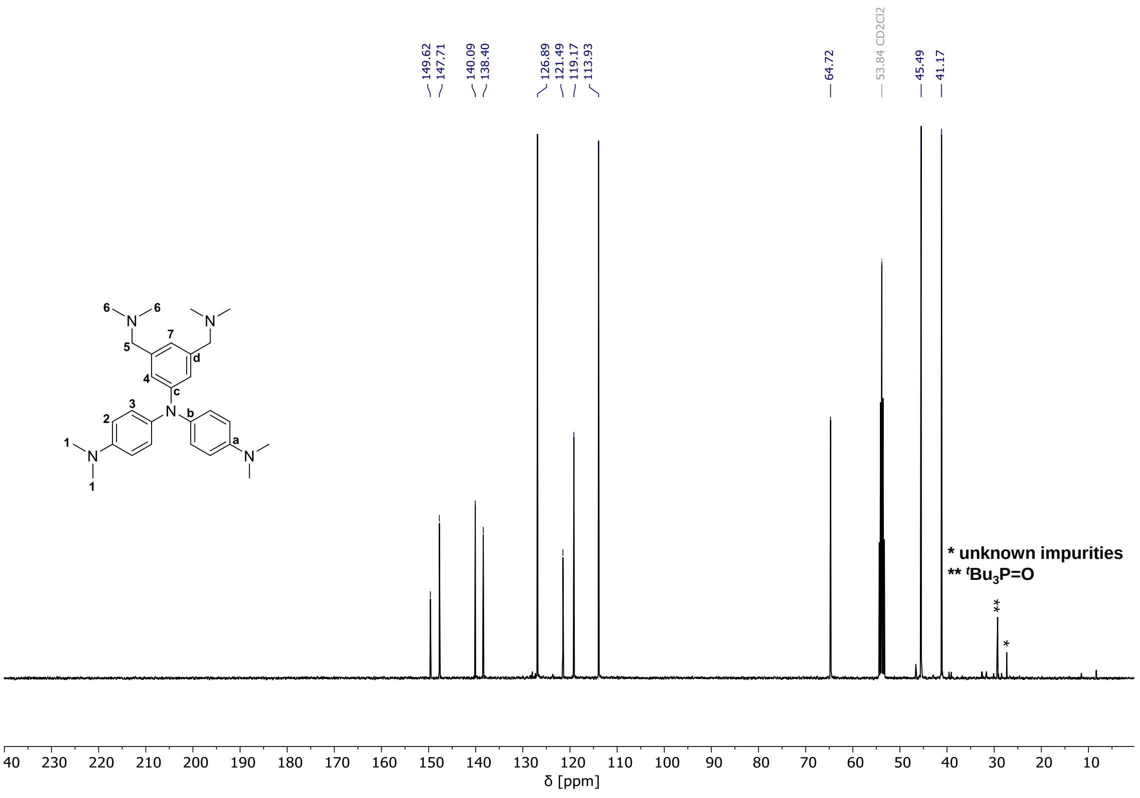

Figure S6. $^{13}C\{^1H\}$-NMR spectrum of ***NCH^{NMe2}N*** in CD_2Cl_2.

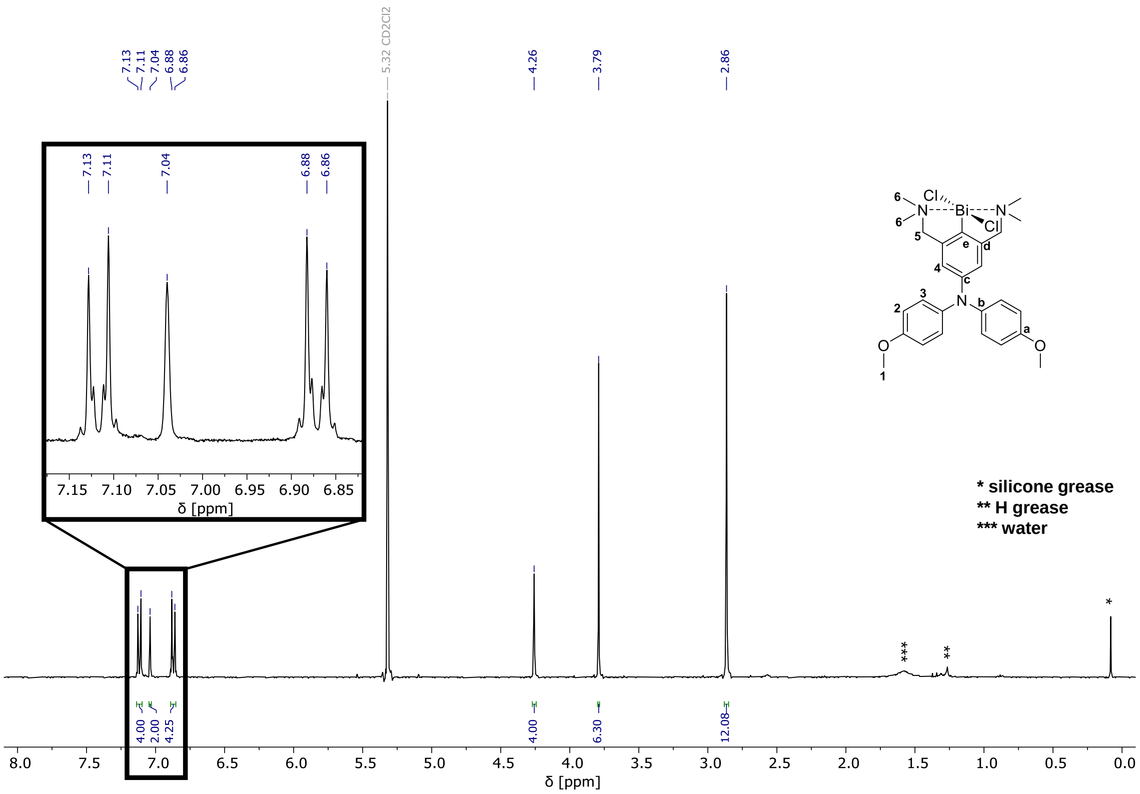

Figure S7. ^{1}H-NMR spectrum of **(*NC*^{OMe}*N*)BiCl₂** in CD_2Cl_2.

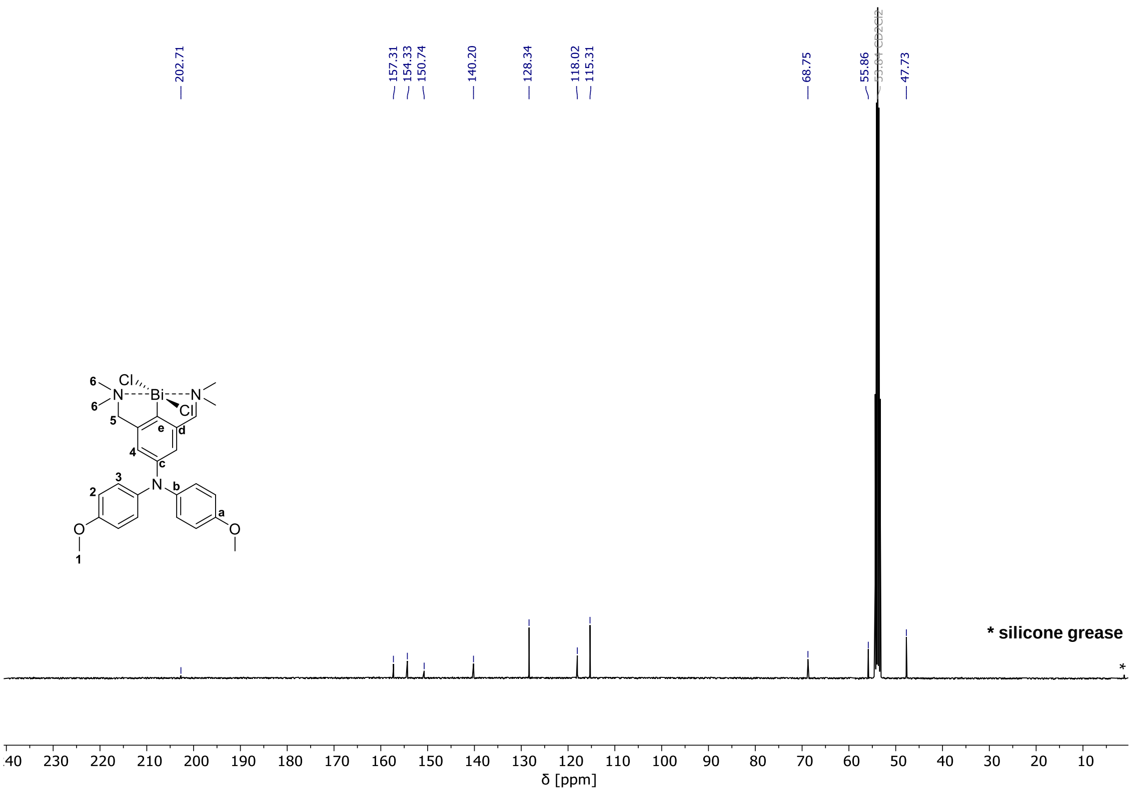

Figure S8. $^{13}C\{^{1}H\}$-NMR spectrum of **(*NC*^{OMe}*N*)BiCl₂** in CD_2Cl_2.

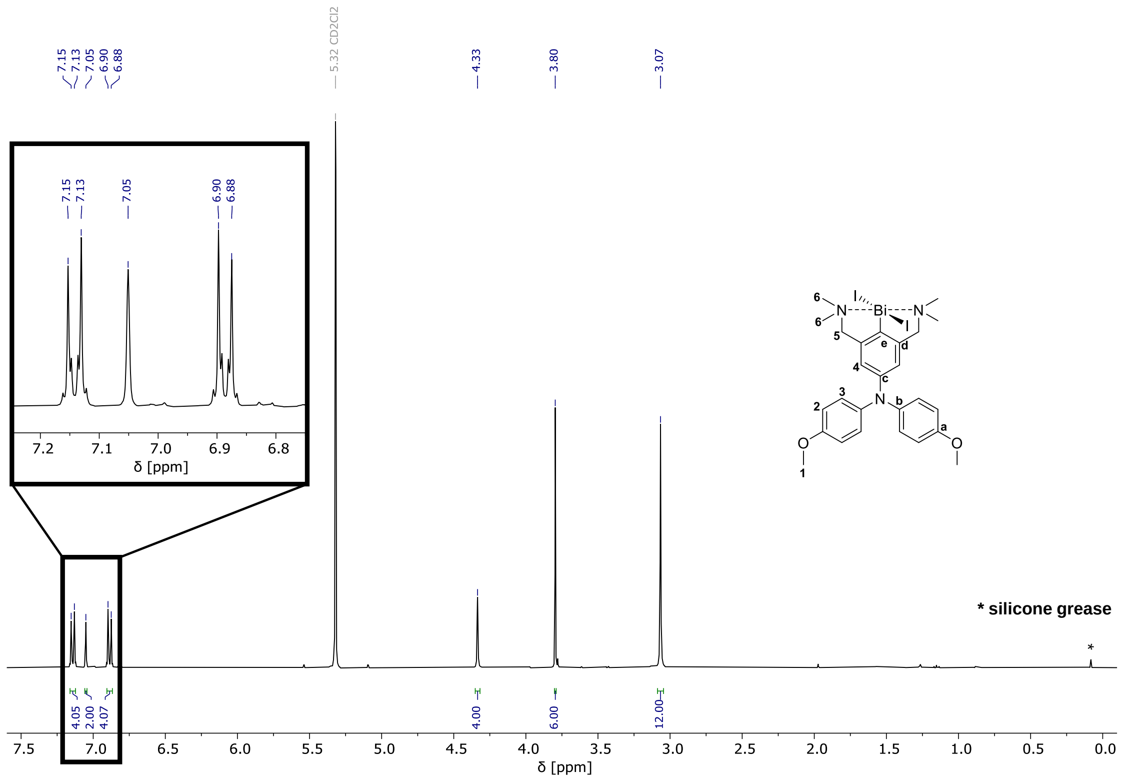

Figure S9. ^{1}H-NMR of **(*NC*^{OMe}*N*)BiI$_2$** in CD_2Cl_2.

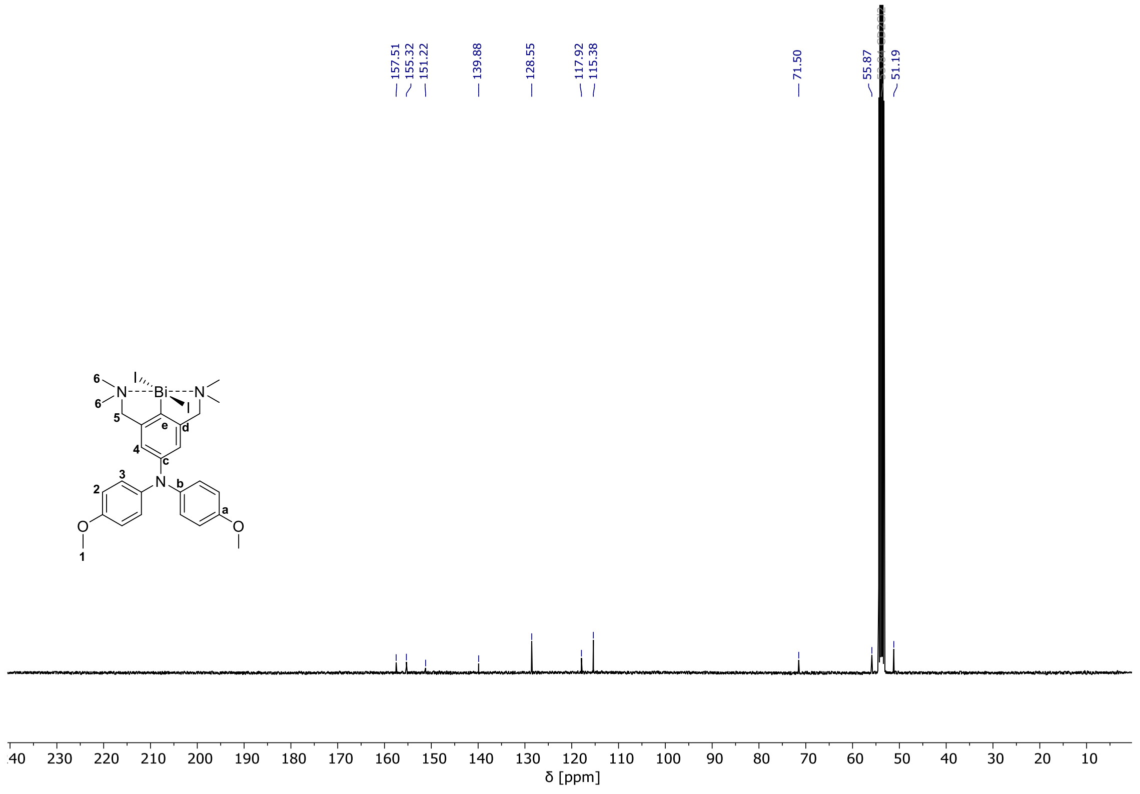

Figure S10. ^{13}C{^{1}H}-NMR spectrum of **(*NC*^{OMe}*N*)BiI$_2$** in CD_2Cl_2.

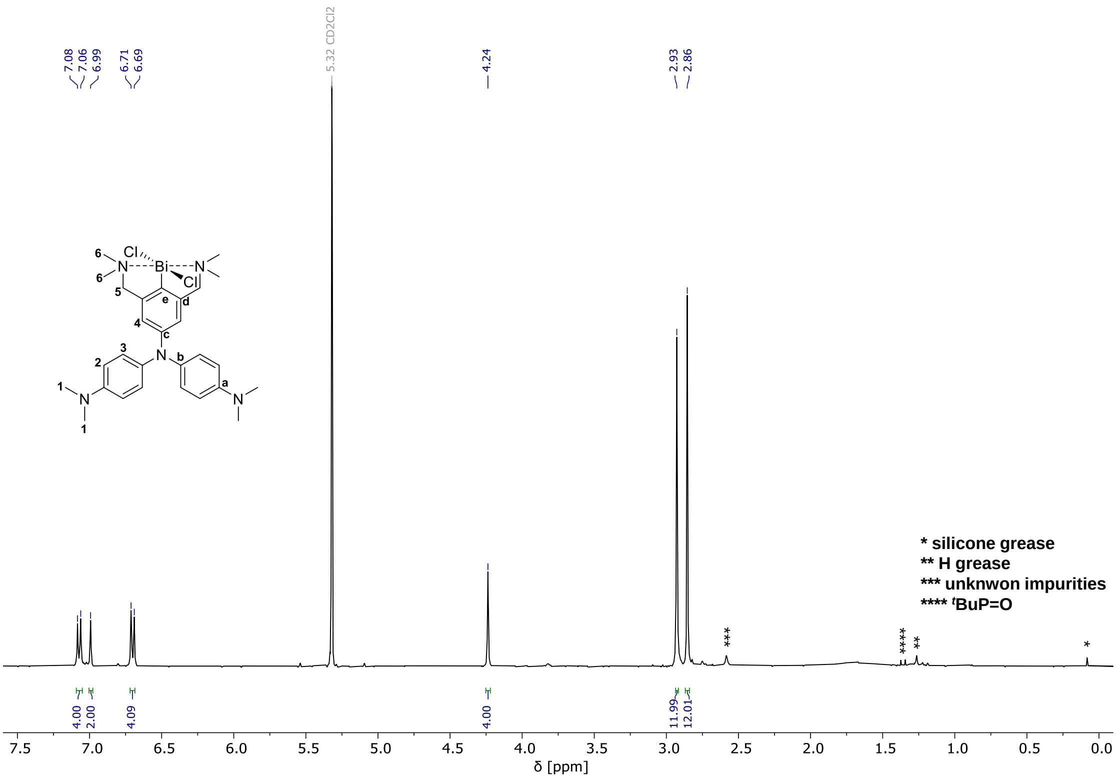

Figure S11. ^{1}H-NMR spectrum of ***(NC^{NMe2}N)*****BiCl$_2$** in CD_2Cl_2.

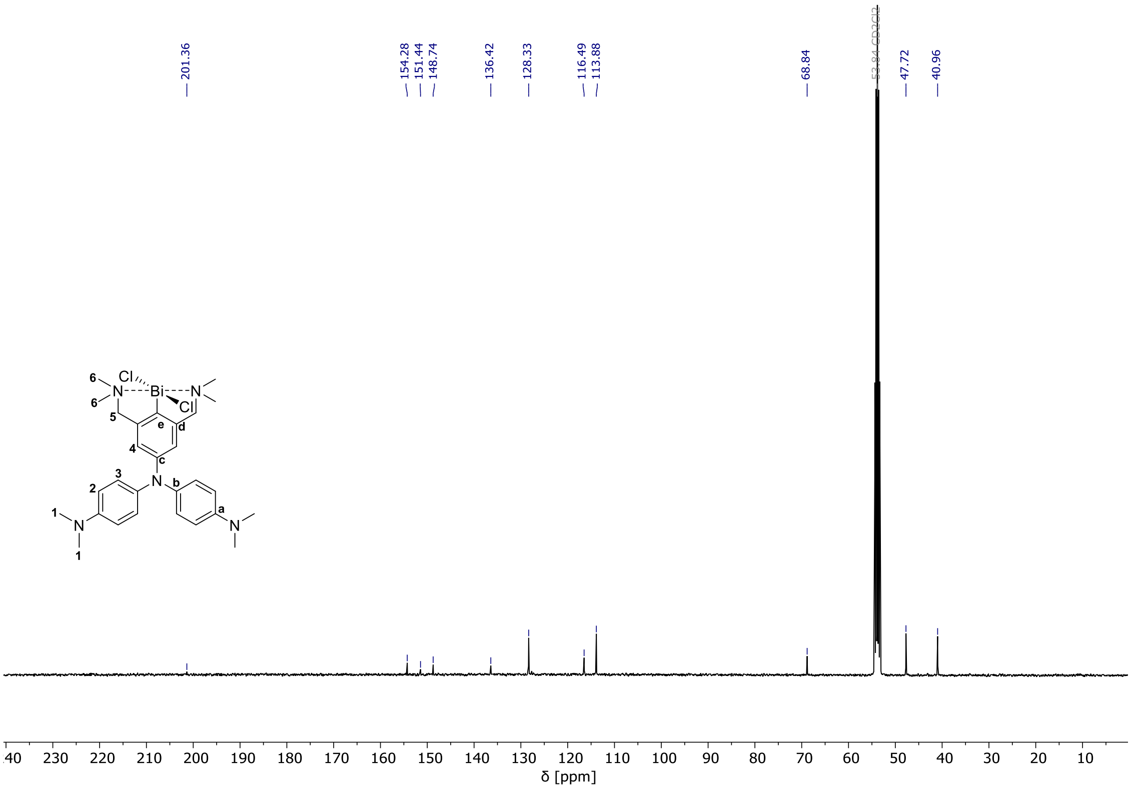

Figure S12. ^{13}C{^{1}H}-NMR spectrum of ***(NC^{NMe2}N)*****BiCl$_2$** in CD_2Cl_2.

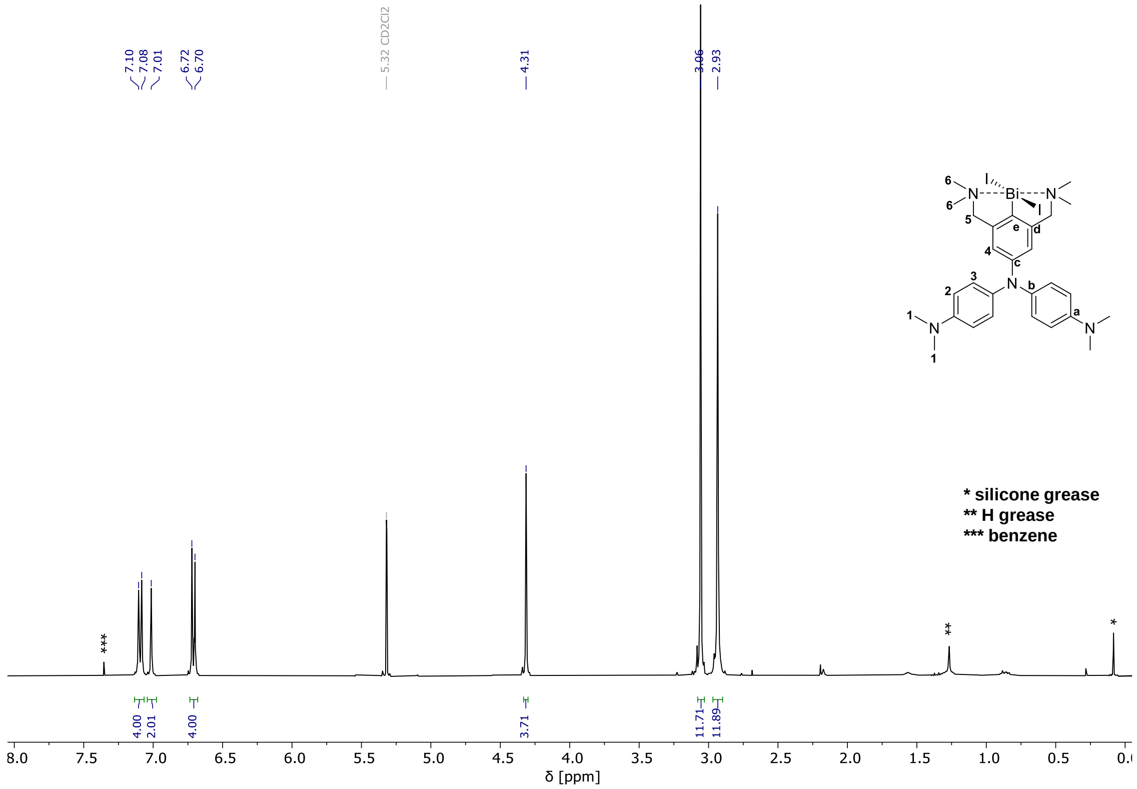

Figure S13. ^{1}H-NMR spectrum of ***(NC^{NMe2}N)*****BiI$_2$** in CD_2Cl_2.

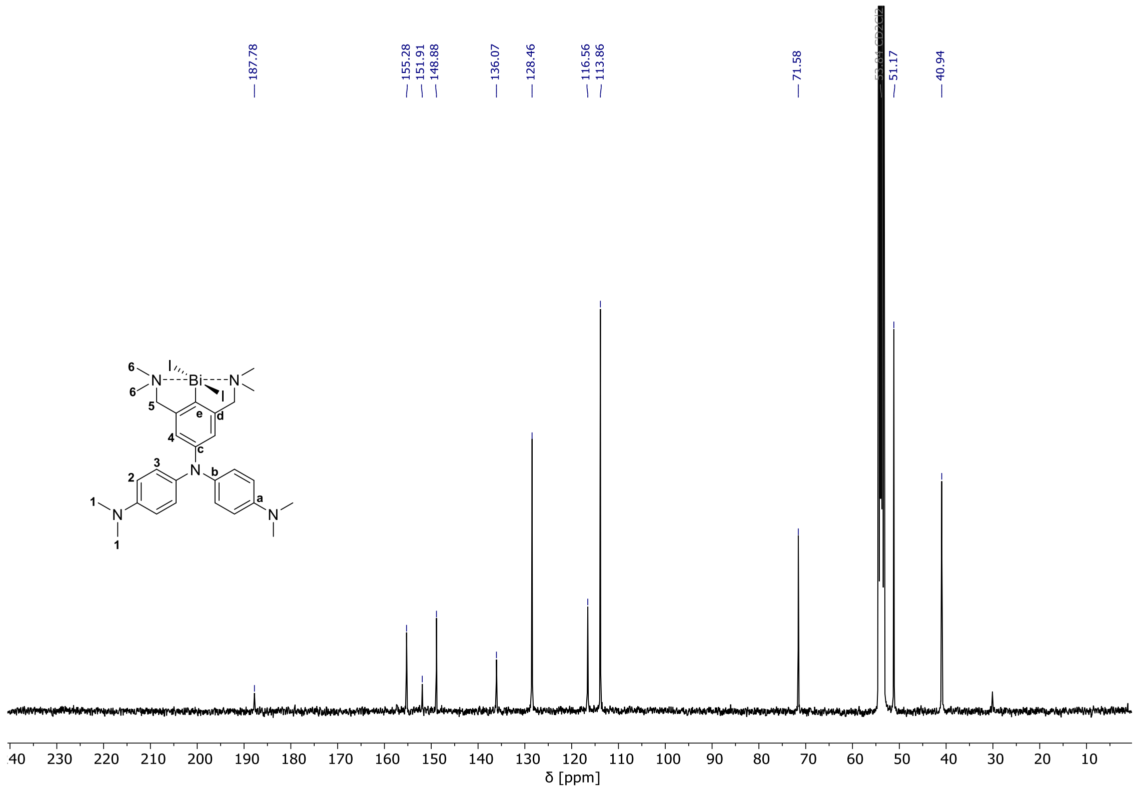

Figure S14. ^{13}C{^{1}H}-NMR spectrum of ***(NC^{NMe2}N)*****BiI$_2$** in CD_2Cl_2.

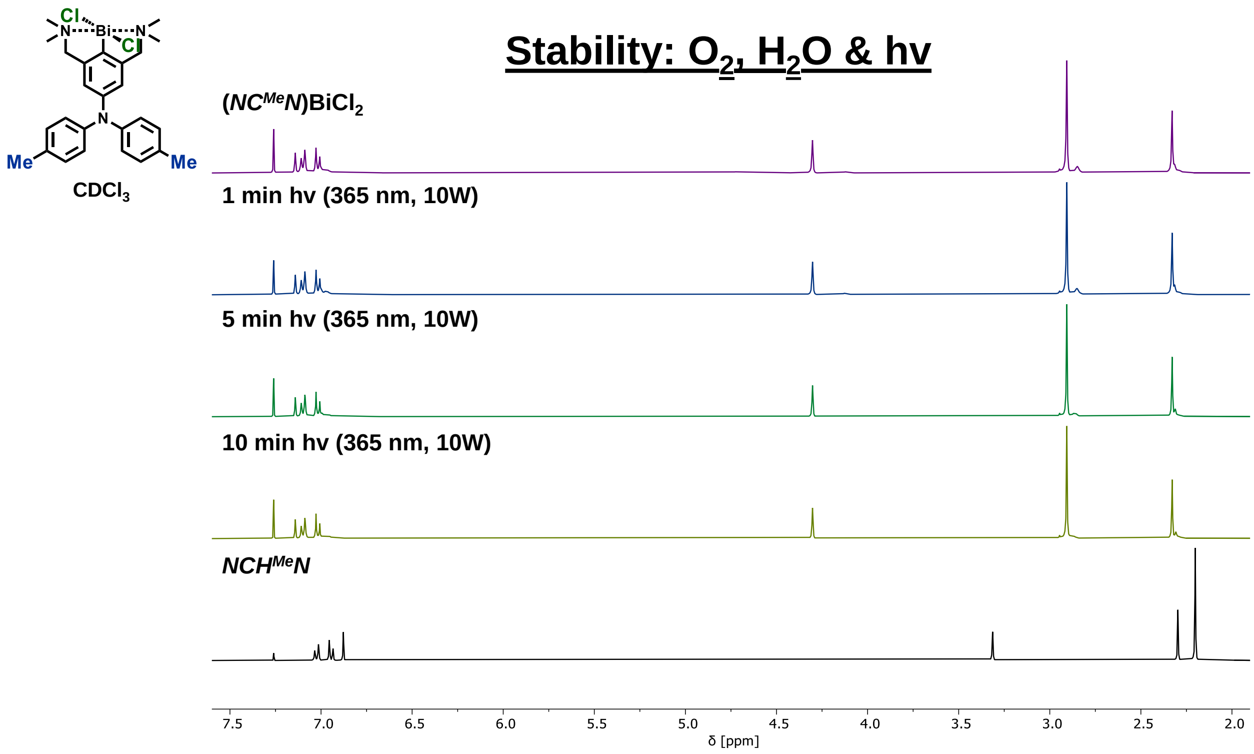

Figure S15. Stability test of ***(NC^{Me}N)*BiCl$_2$** in CDCl$_3$.

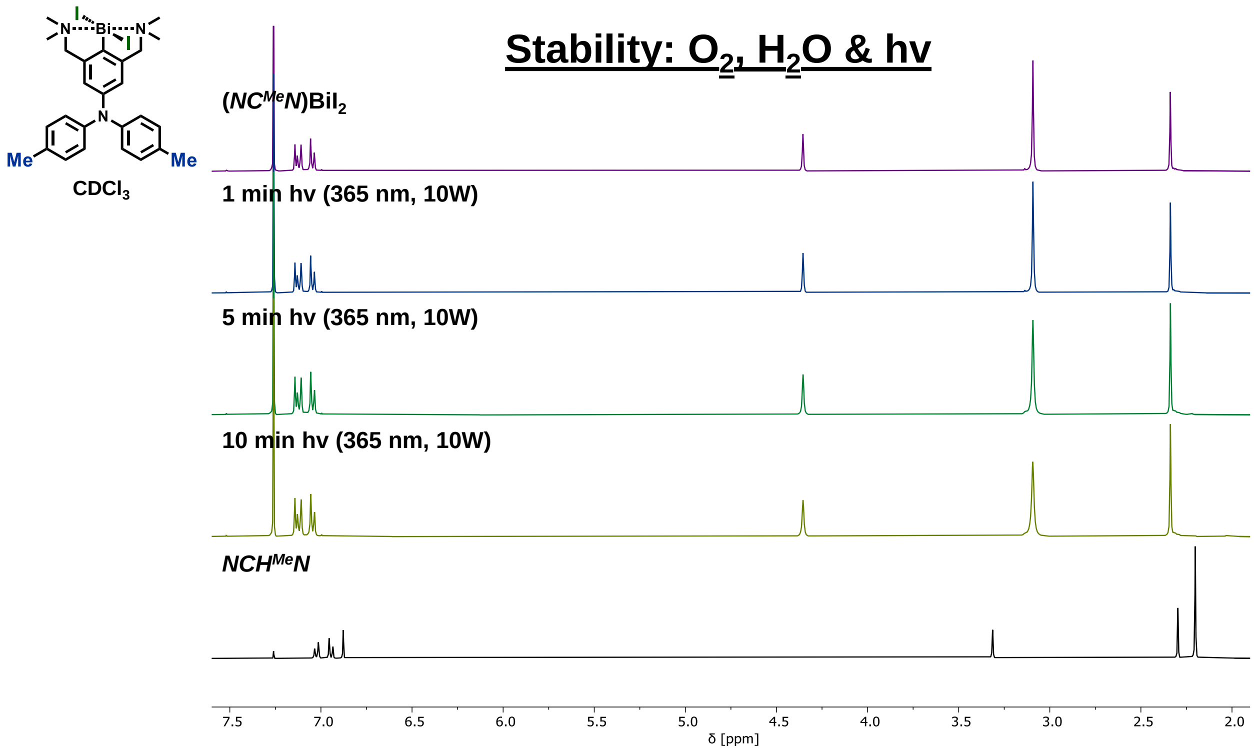

Figure S16. Stability test of ***(NC^{Me}N)*BiI$_2$** in CDCl$_3$.

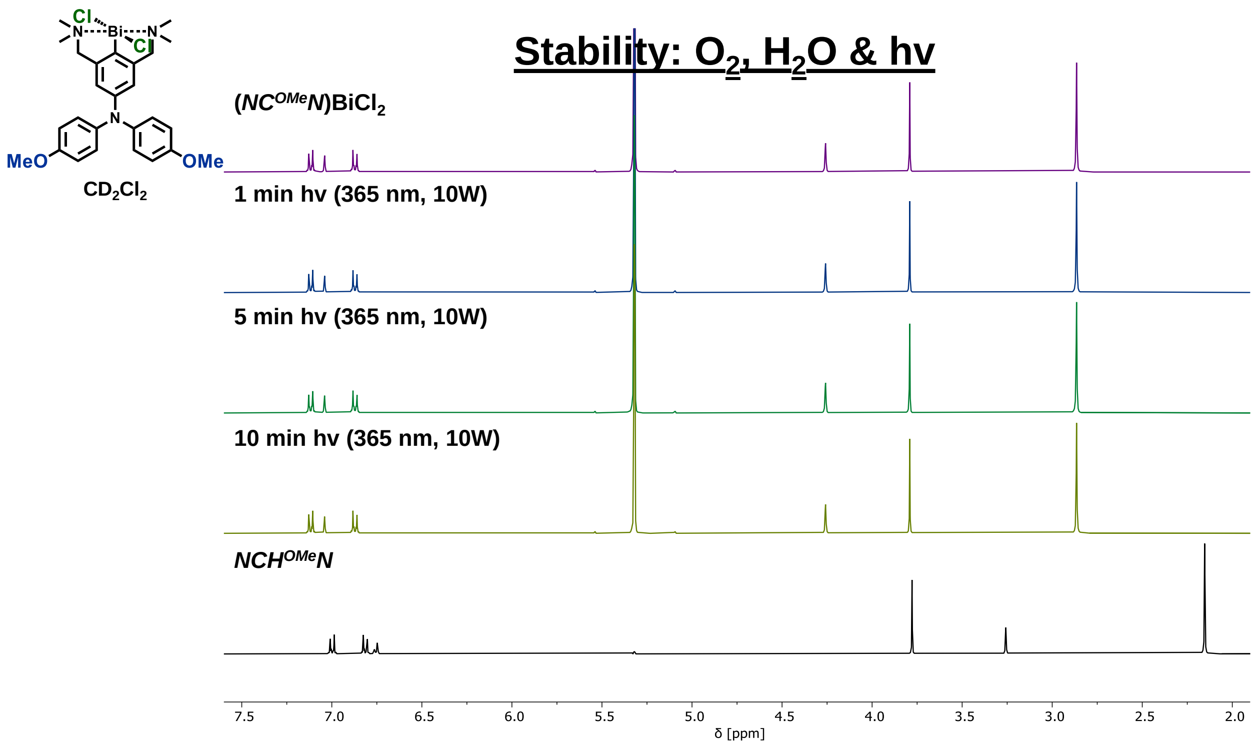

Figure S17. Stability test of ***(NC^OMe^N)*BiCl$_2$** in CD_2Cl_2.

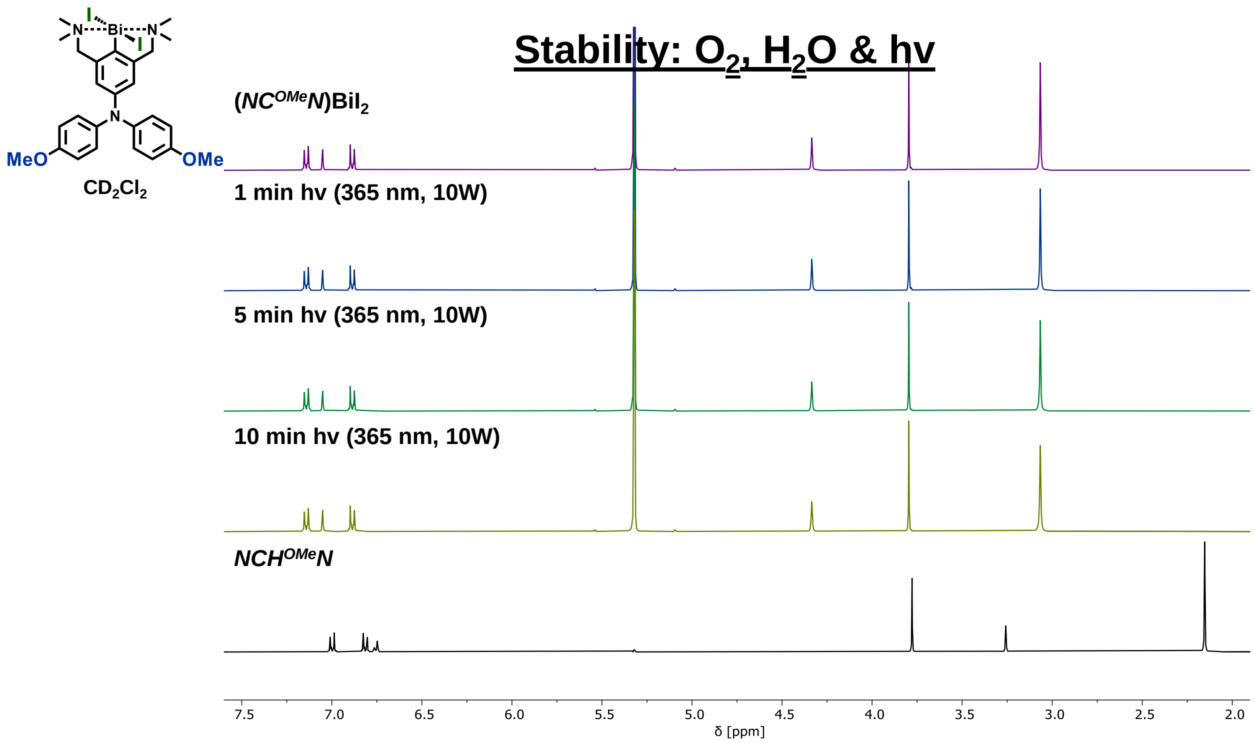

Figure S18. Stability test of ***(NC^OMe^N)*BiI$_2$** in CD_2Cl_2.

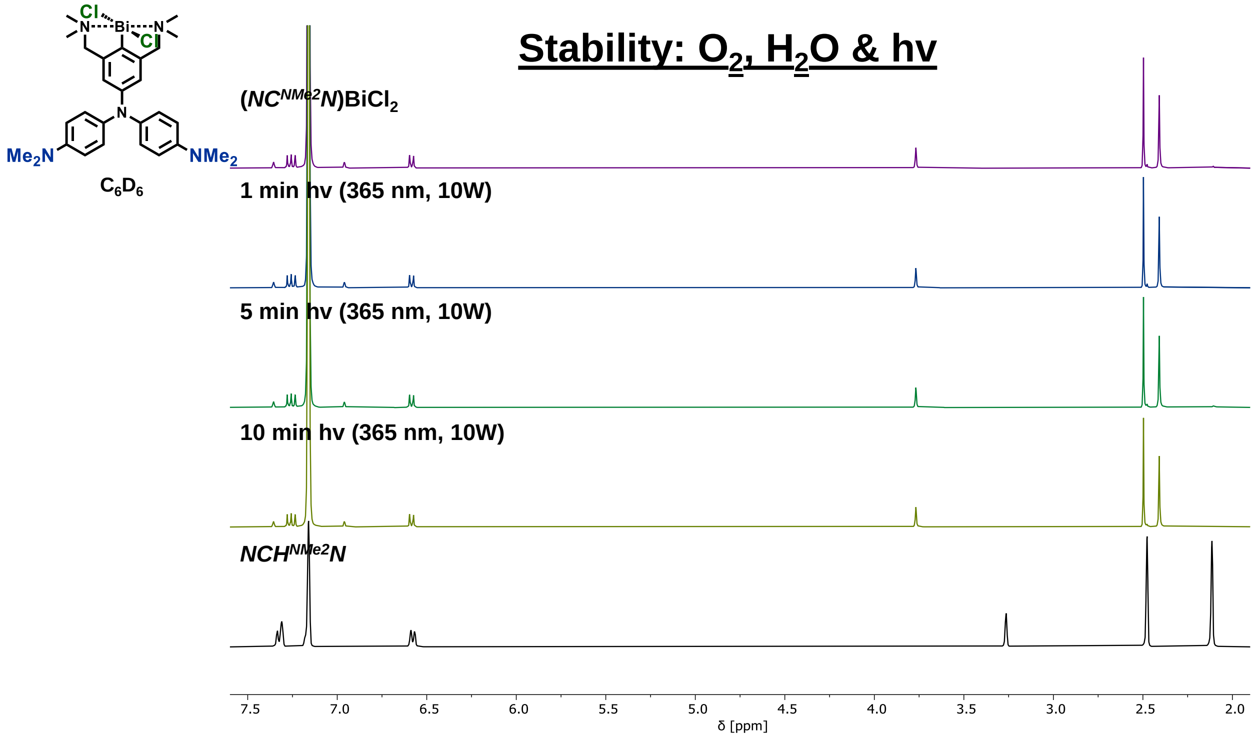

Figure S19. Stability test of **(*NC*^{NMe2}*N*)BiCl$_2$** in C_6D_6.

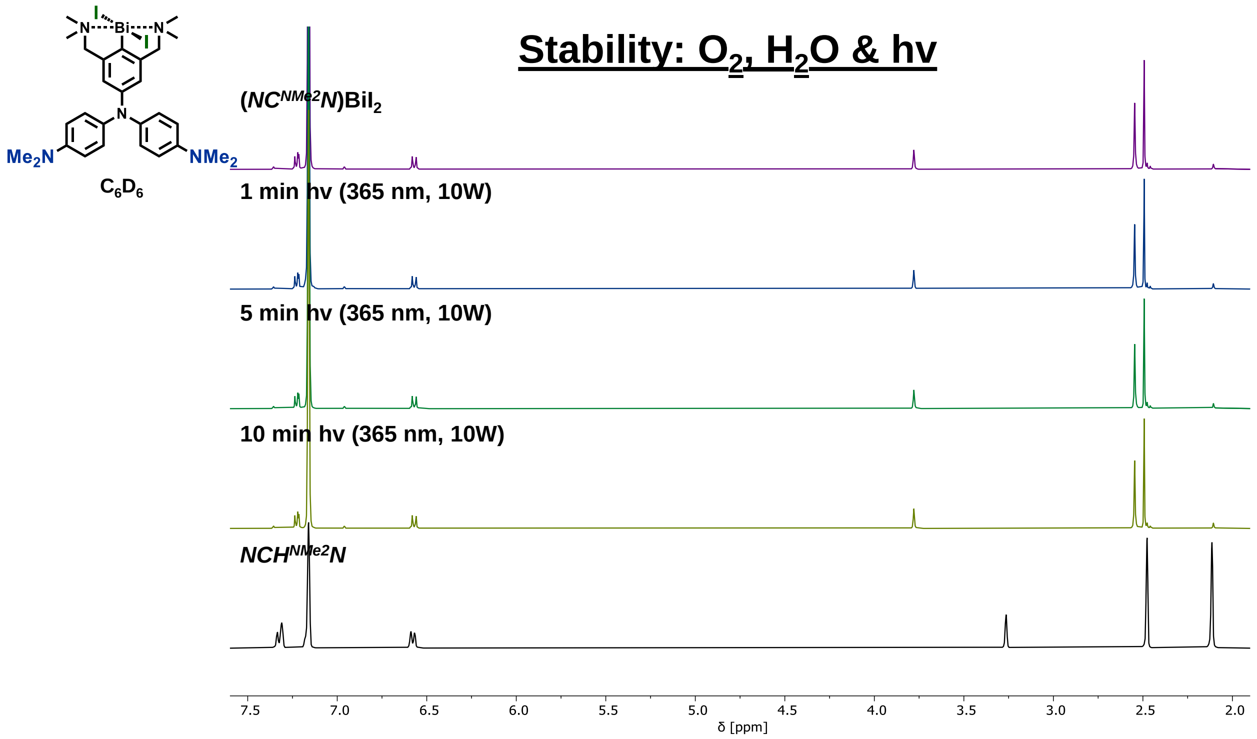

Figure S20. Stability test of **(*NC*^{NMe2}*N*)BiI$_2$** in C_6D_6.

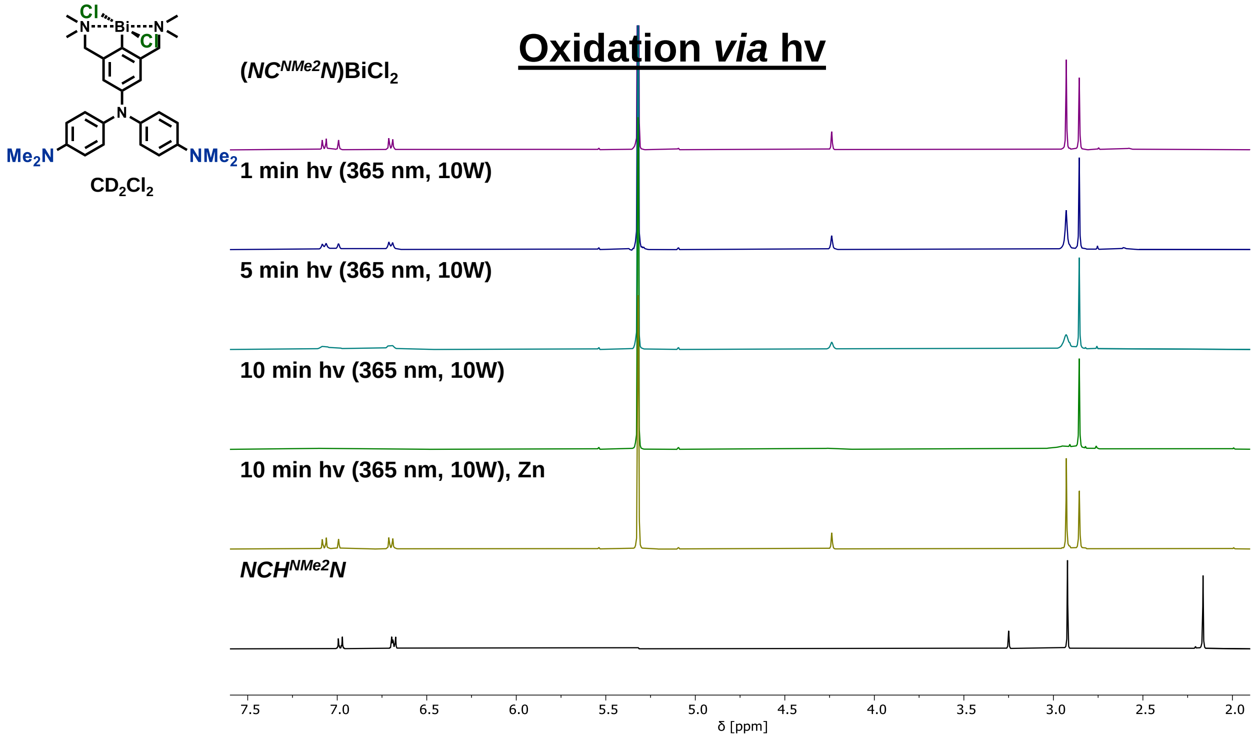

Figure S21. Photooxidation and zinc reduction of **(*NC*^{NMe2}*N*)BiCl$_2$** in CD_2Cl_2.

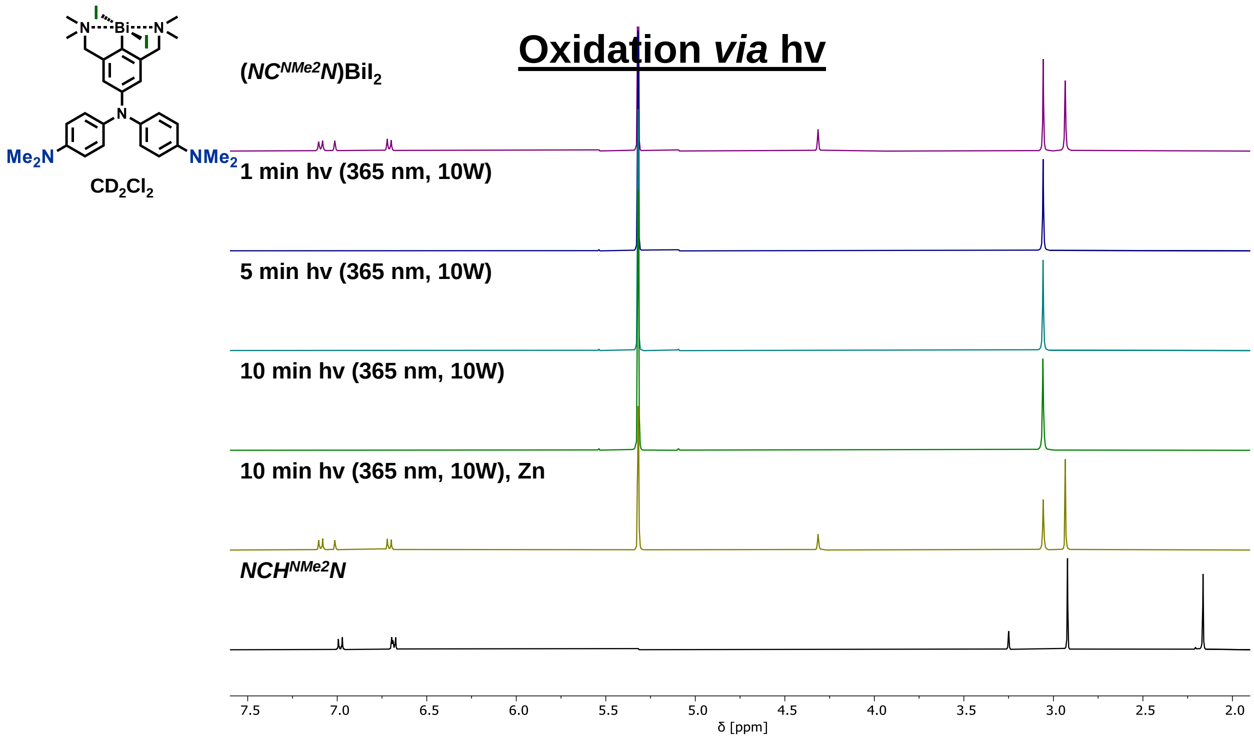

Figure S22. Photoxidation and zinc reduction of **(*NC*^{NMe2}*N*)BiI$_2$** in CD_2Cl_2.

ESI-MS Data

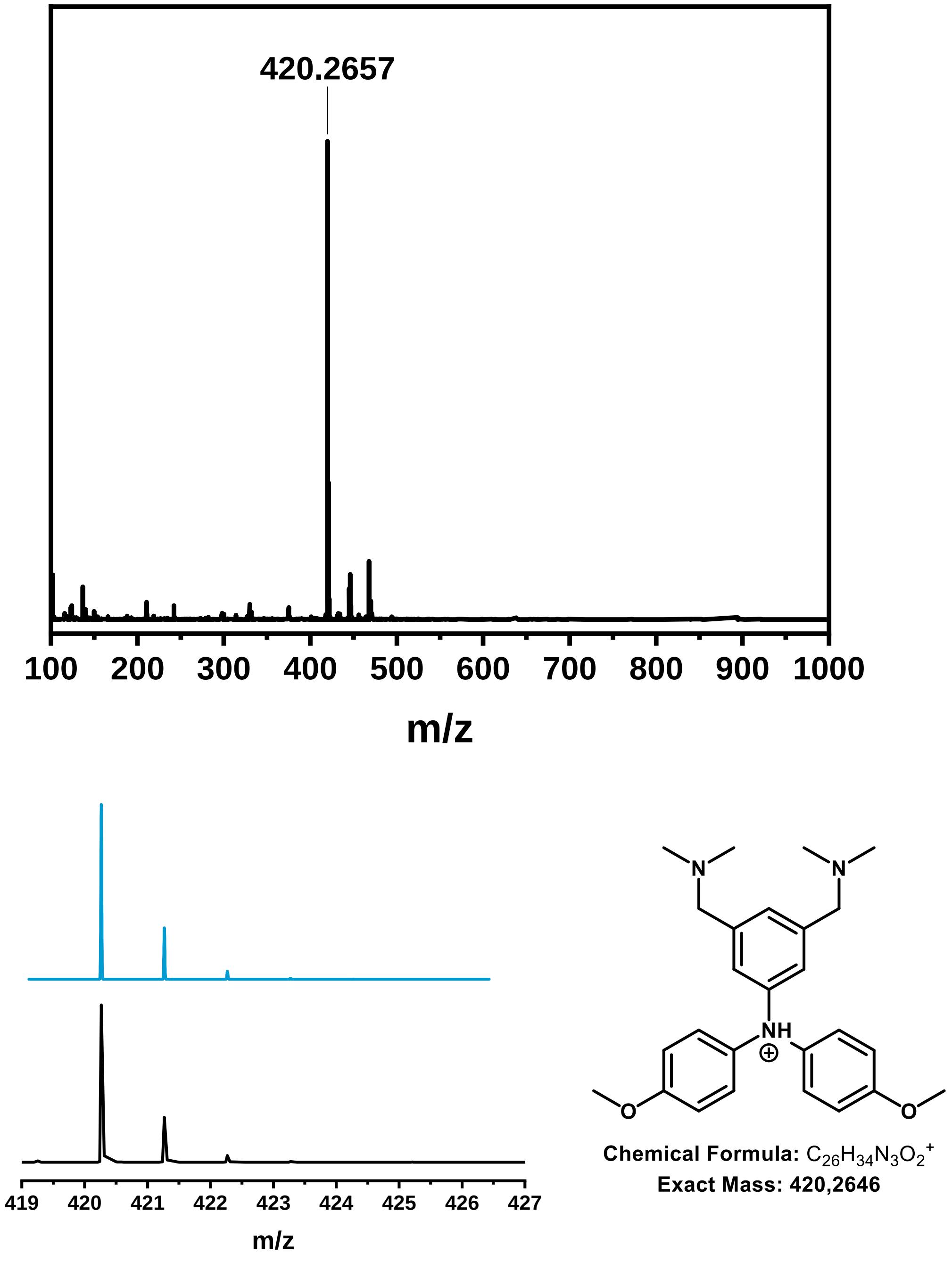

Figure S23. ESI-MS of ***NCH***$^{\boldsymbol{OMe}}$***N*** in CH_2Cl_2 (experimental data in black, calculated data in blue).

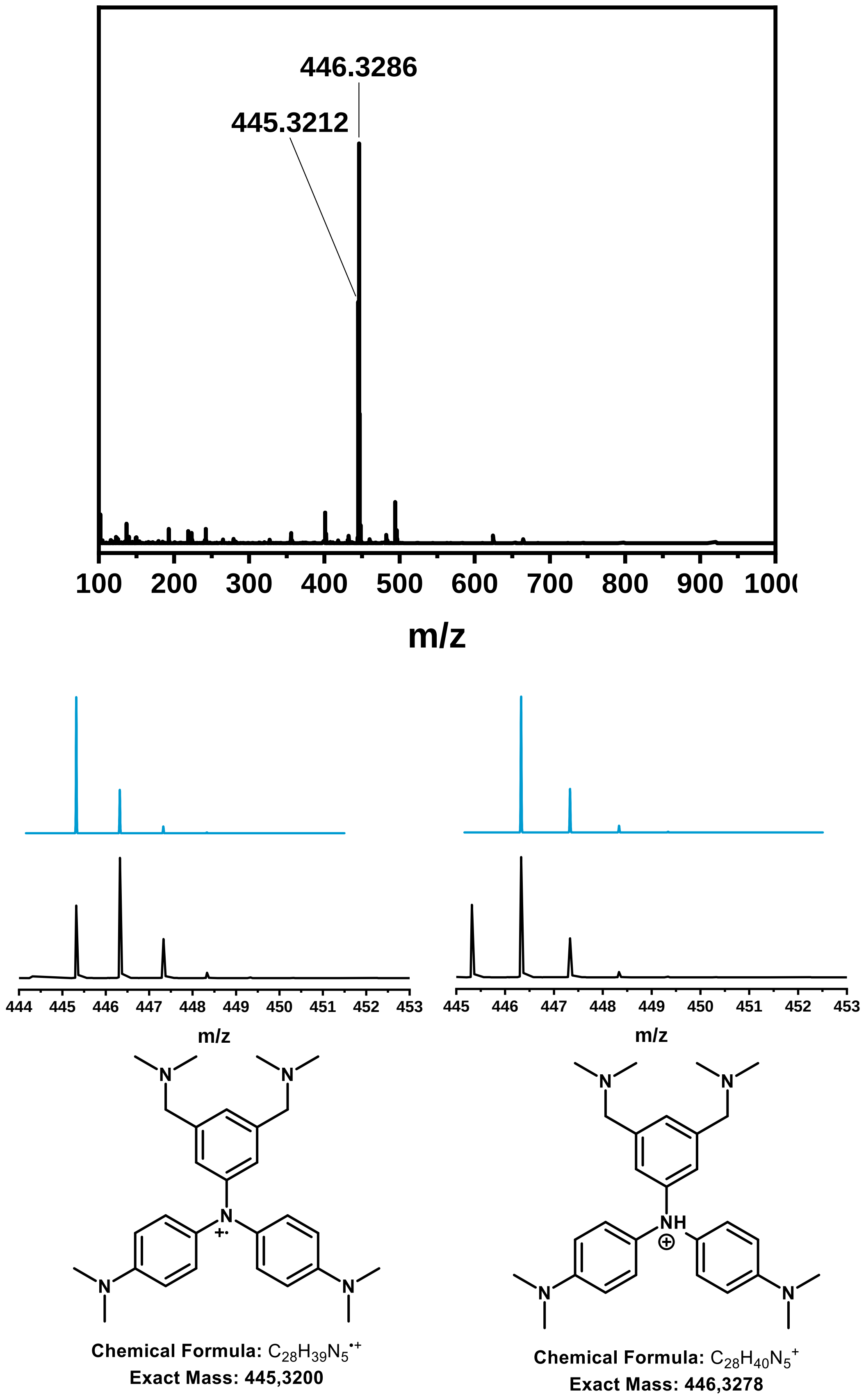

Figure S 24. ESI-MS of ***NCH***$^{\boldsymbol{NMe2}}$***N*** in CH_2Cl_2 (experimental data in black, calculated data in blue).

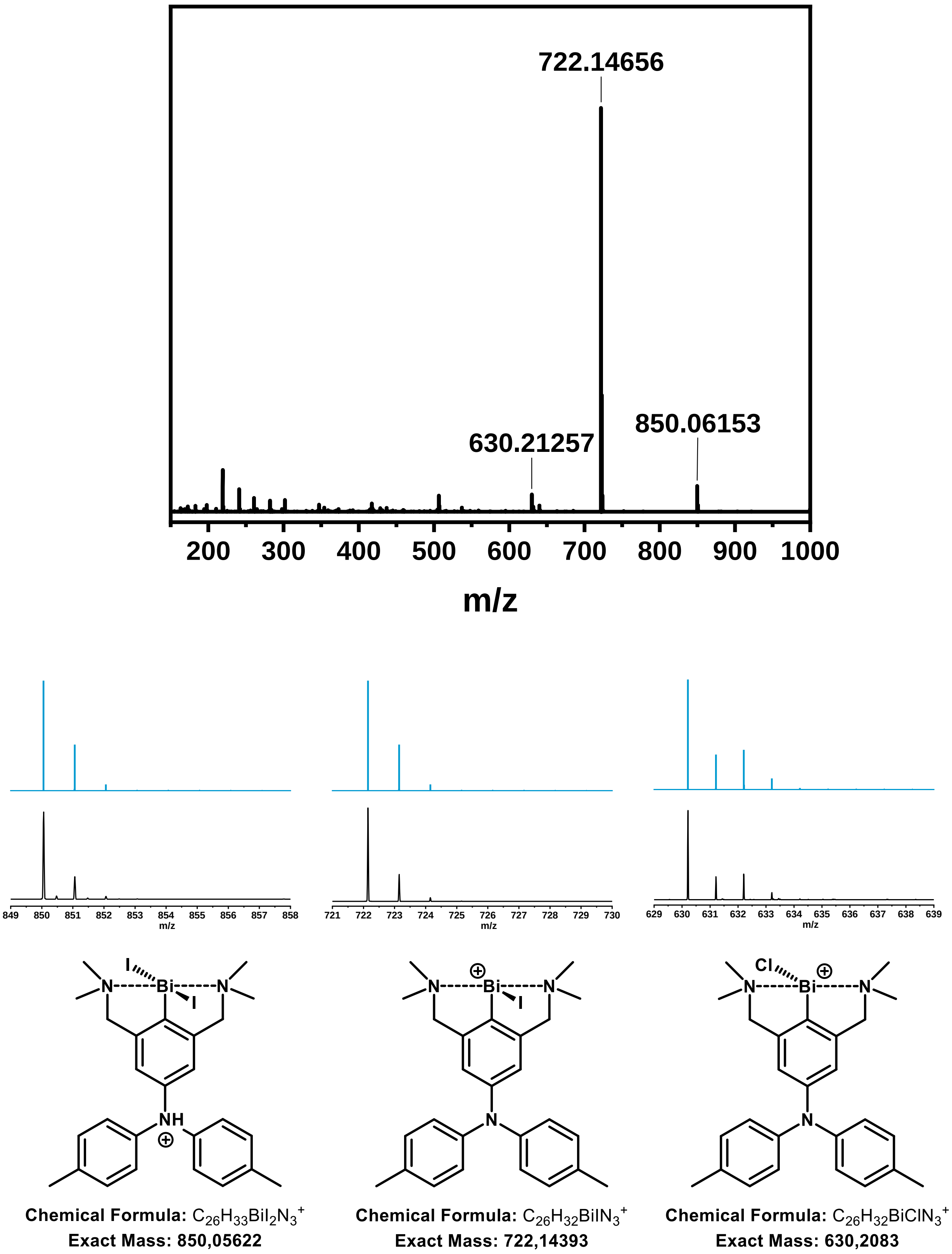

Figure S25. ESI-MS of **(*NC*^{Me}*N*)BiI$_2$** in CH_2Cl_2 (experimental data in black, calculated data in blue).

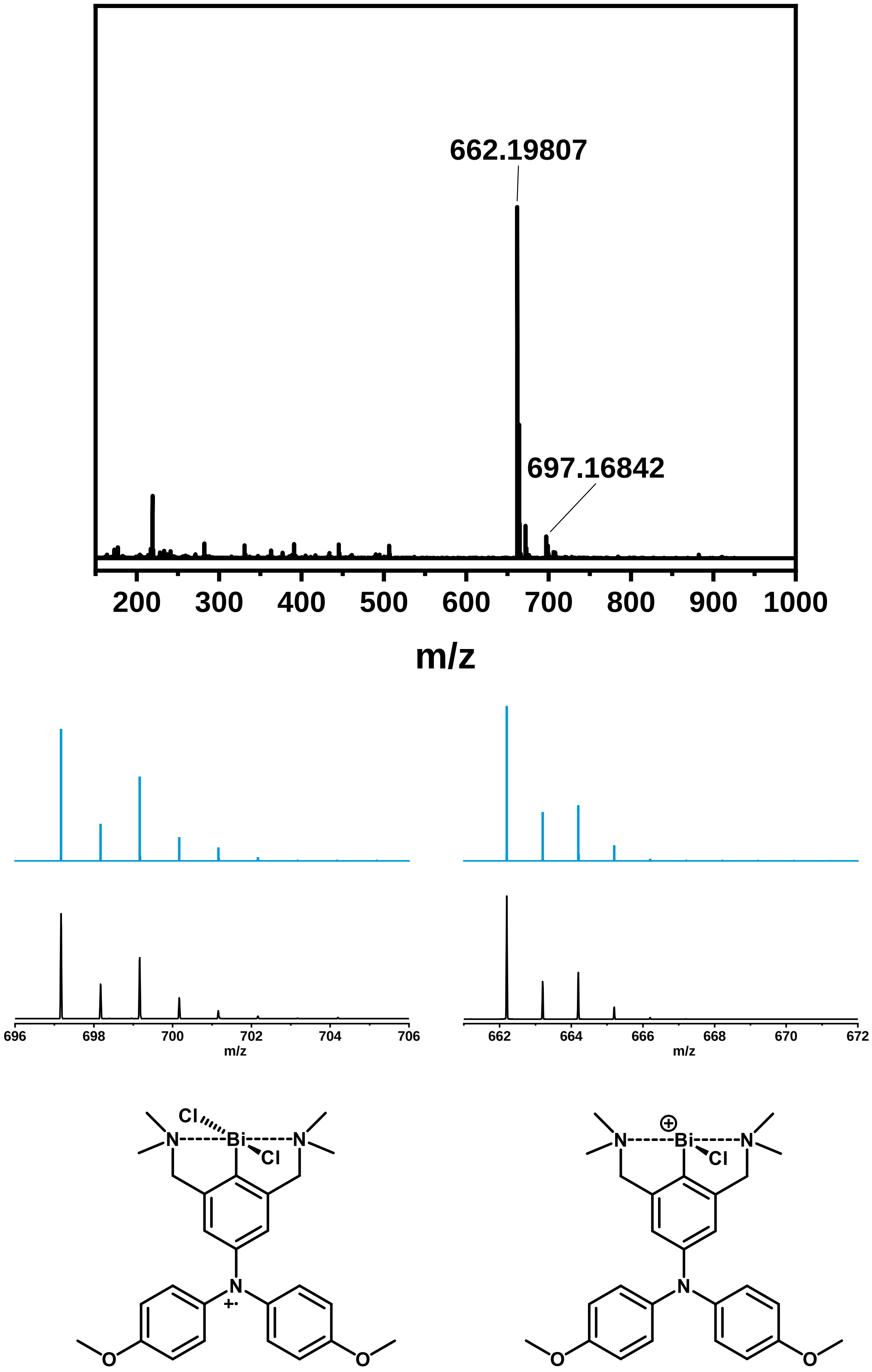

Chemical Formula: $C_{26}H_{32}BiCl_2N_3O_2^{\bullet+}$
Exact Mass: 697,17

Chemical Formula: $C_{26}H_{32}BiClN_3O_2^{+}$
Exact Mass: 662,1981

Figure S26. ESI-MS of **(*NC*^{OMe}*N*)BiCl$_2$** in CH_2Cl_2 (experimental data in black, calculated data in blue).

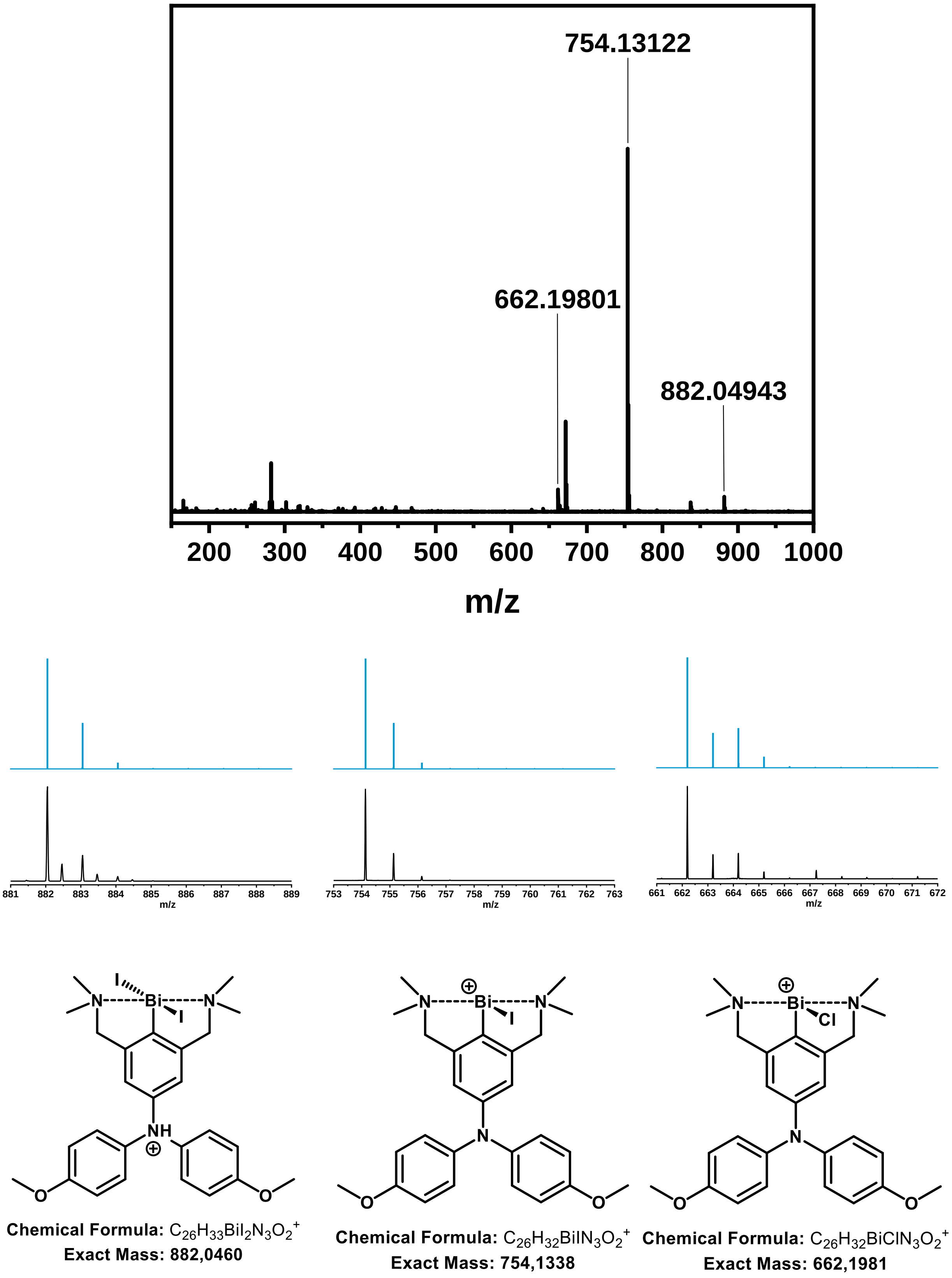

Figure S27. ESI-MS of **(*NC*^{OMe}*N*)BiI$_2$** in CH_2Cl_2 (experimental data in black, calculated data in blue).

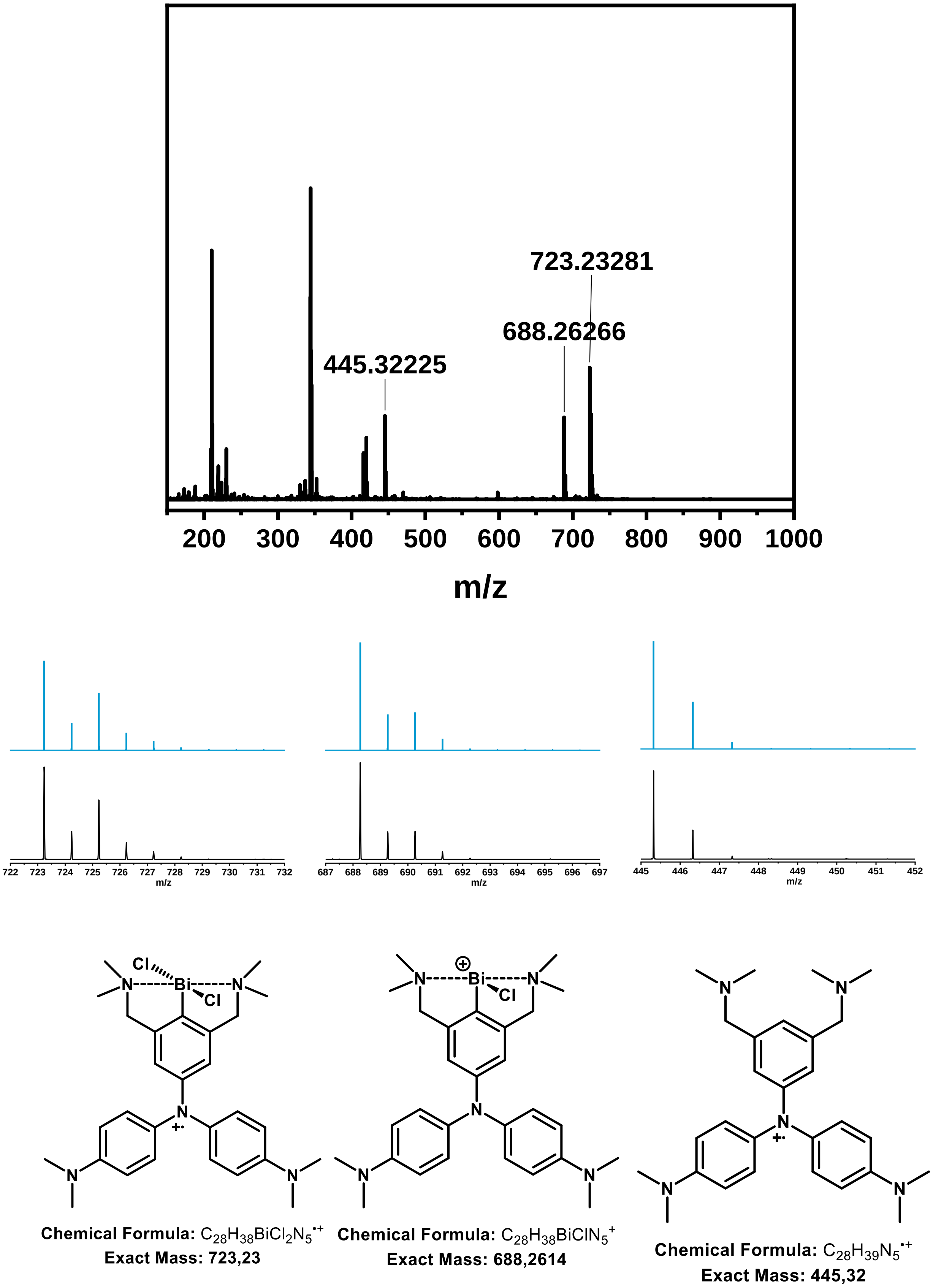

Figure S28. ESI-MS of **(*NC*^{NMe2}*N*)BiCl$_2$** in CH_2Cl_2 (experimental data in black, calculated data in blue).

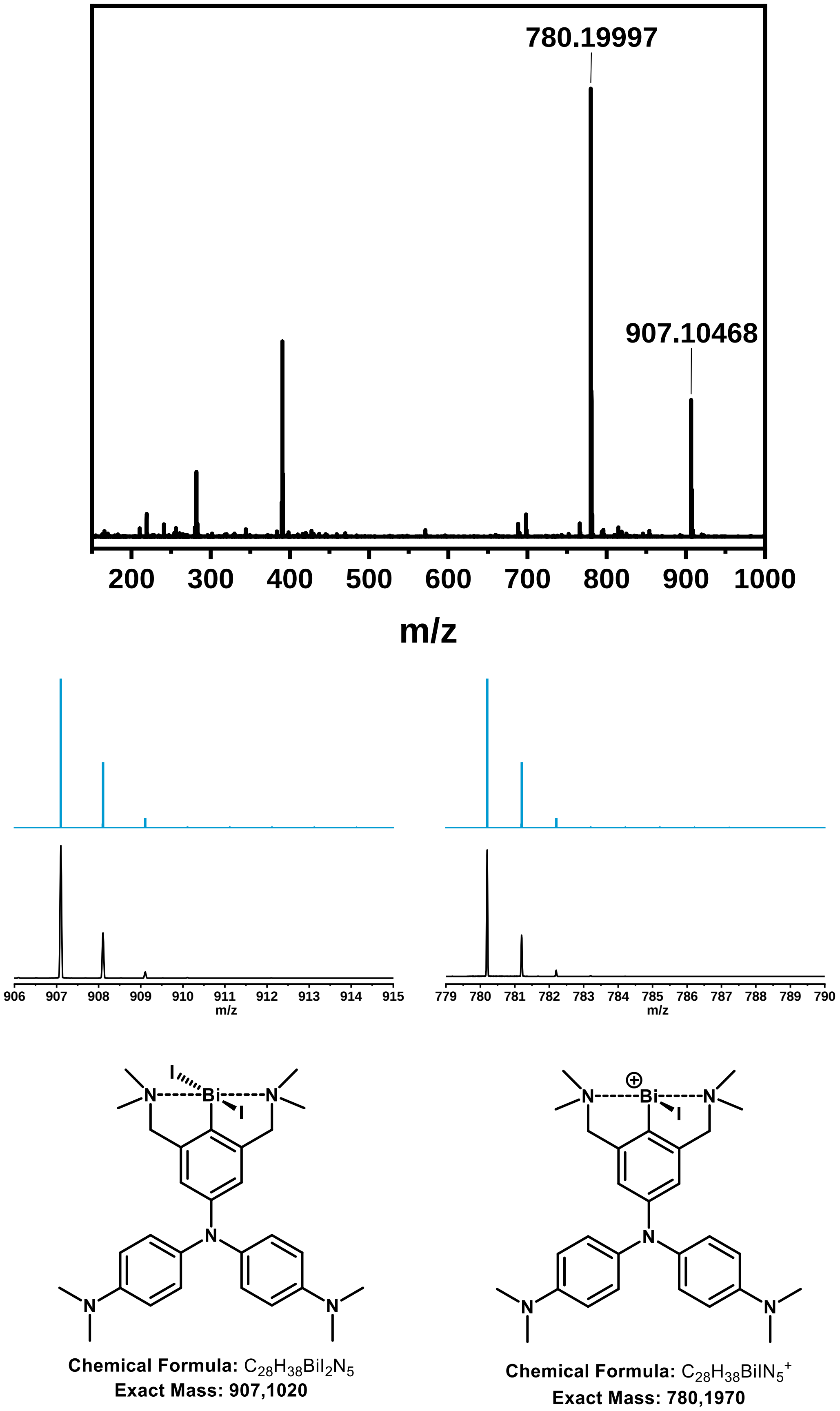

Figure S29. ESI-MS of **(*NC*^{NMe2}*N*)BiI$_2$** in CH_2Cl_2 (experimental data in black, calculated data in blue).

UV/Vis Spectroscopy

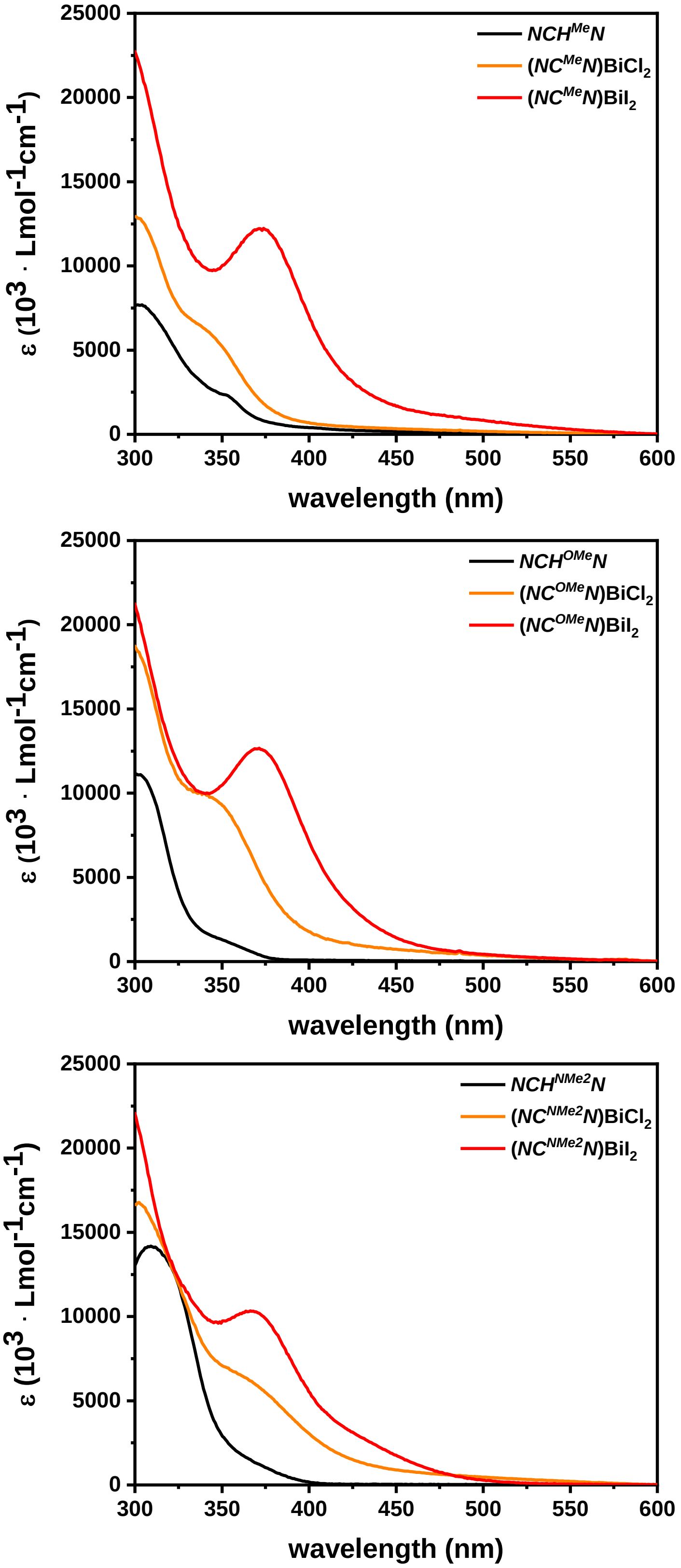

Figure S30. Top: UV/vis absorption spectra of ***NCH^{Me}N*** (black), **(*NC^{Me}N*)BiCl$_2$** (orange), and **(*NC^{Me}N*)BiI$_2$** (red) in THF. Middle: UV/vis absorption spectra of ***NCH^{OMe}N*** (black), **(*NC^{OMe}N*)BiCl$_2$** (orange), and **(*NC^{OMe}N*)BiI$_2$** (red) in THF. Bottom: UV/vis absorption spectra of ***NCH^{NMe2}N*** (black), **(*NC^{NMe2}N*)BiCl$_2$** (orange), and **(*NC^{NMe2}N*)BiI$_2$** (red) in THF.

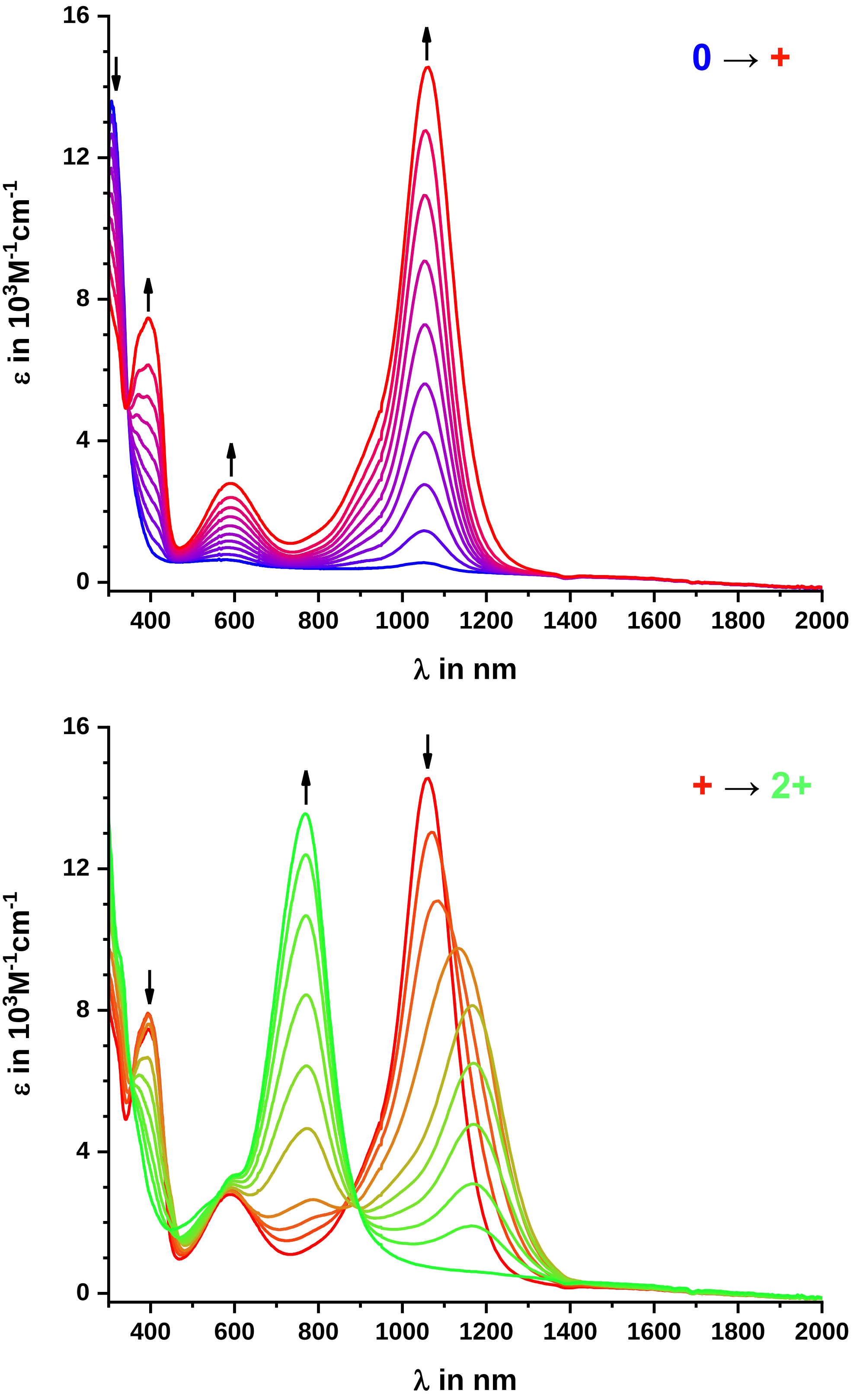

Figure S31. Changes of the UV/vis/NIR spectra during electrochemical oxidation of ***NCH***^{NMe2}***N*** to its radical cation (top), and during further oxidation to the corresponding dication (bottom), measured in CH_2Cl_2/$^{n}Bu_4N^+$ $[BAr^{F24}]^-$ (0.1 M) at room temperature.

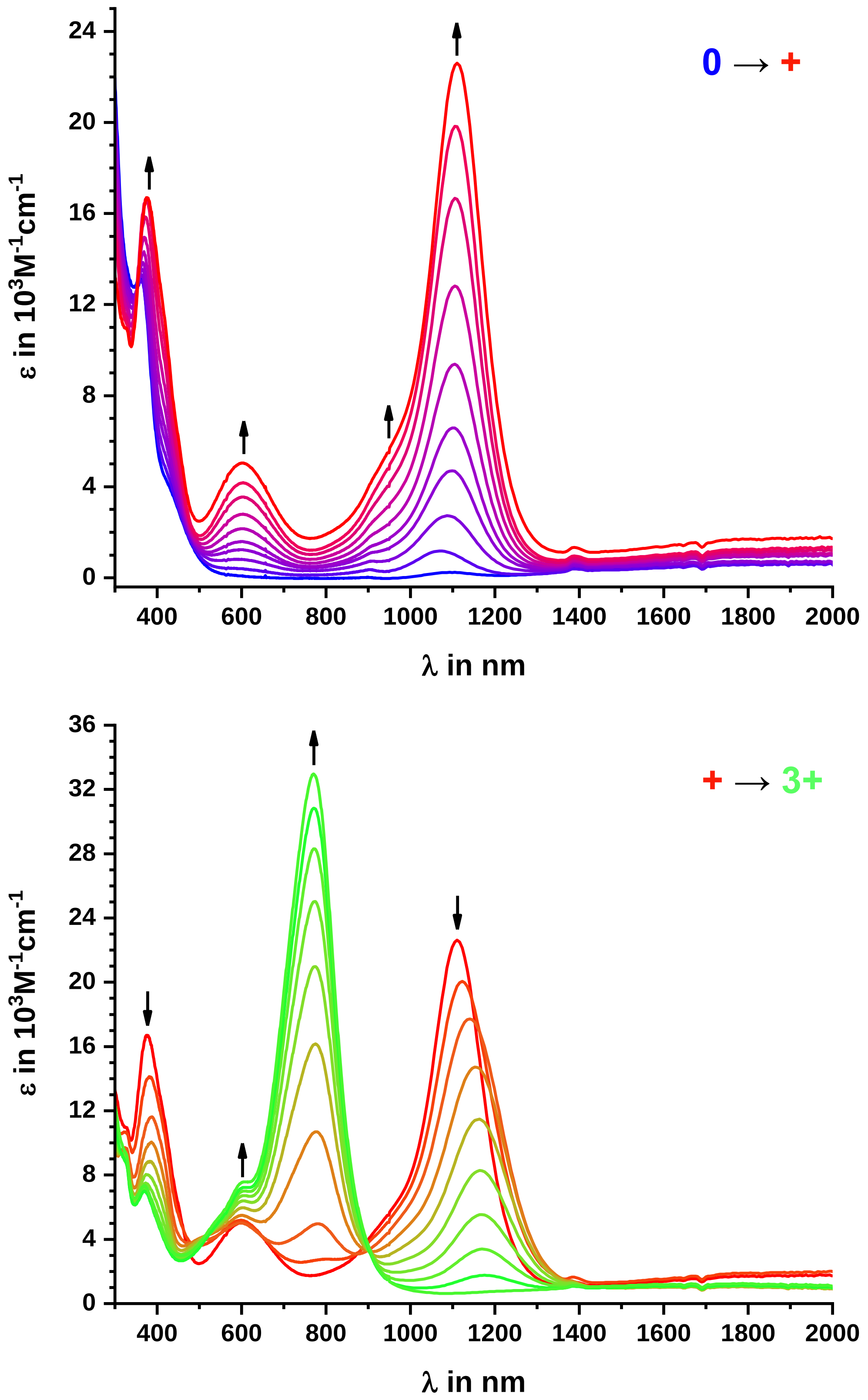

Figure S32. Changes of the UV/vis/NIR spectra during electrochemical oxidation of **(*NC*^{NMe2}*N*)BiI$_2$** to its radical cation (top), and during further oxidation to the corresponding trication (bottom). measured in CH_2Cl_2/$^{n}Bu_4N^+$ [BAr^{F24}]$^-$ (0.1 M) at room temperature.

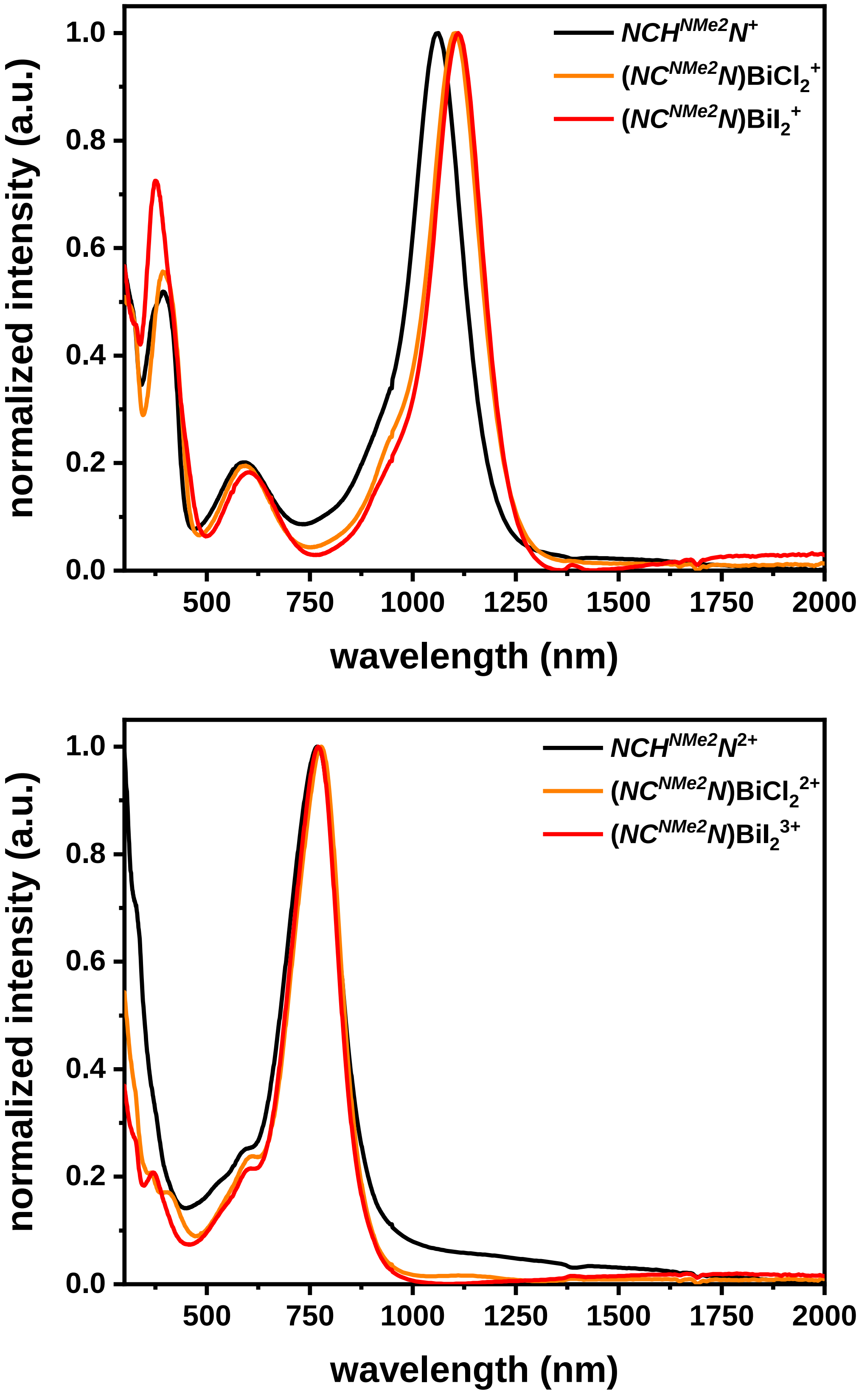

Figure S33. Top: Comparison of UV/vis/NIR spectra of the electrochemically radical cations of ***NCH^NMe2^N***, **(*NC^NMe2^N*)BiCl₂**, and **(*NC^NMe2^N*)BiI₂**, measured in $CH_2Cl_2/^{n}Bu_4N^+$ $[BAr^{F24}]^-$ (0.1 M) at room temperature. Bottom: Comparison of UV/vis/NIR spectra of the electrochemically oxidized dications of ***NCH^NMe2^N*** and **(*NC^NMe2^N*)BiCl₂**, and the trication of **(*NC^NMe2^N*)BiI₂** measured in $CH_2Cl_2/^{n}Bu_4N^+$ $[BAr^{F24}]^-$ (0.1 M) at room temperature.

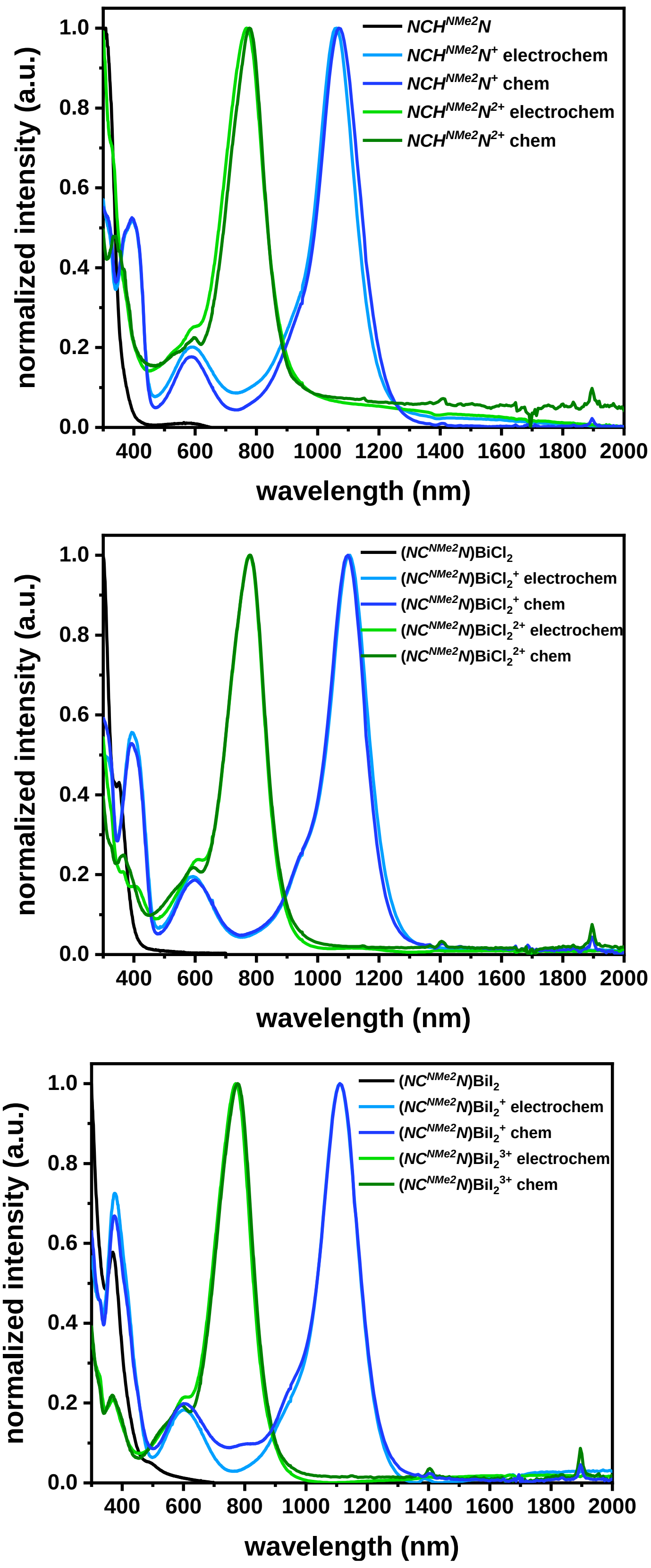

Figure S34. Top: Comparison of UV/vis/NIR spectra in CH_2Cl_2 of electrochemically ($^{n}Bu_4N^+$ $[BAr^{F24}]^-$ (0.1 M)) and chemically (with NO^+ $[SbF_6]^-$) oxidized mono and dications of ***NCH^{NMe2}N***. Middle: Comparison of UV/vis/NIR spectra in CH_2Cl_2 of electrochemically ($^{n}Bu_4N^+$ $[BAr^{F24}]^-$ (0.1 M)) and chemically (with NO^+ $[SbF_6]^-$) oxidized mono- and dications of **(*NC^{NMe2}N*)BiCl$_2$**. Bottom: Comparison of UV/vis/NIR spectra in CH_2Cl_2 of electrochemically ($^{n}Bu_4N^+$ $[BAr^{F24}]^-$ (0.1 M)) and chemically (with NO^+ $[SbF_6]^-$) oxidized mono- and trications of **(*NC^{NMe2}N*)BiI$_2$**.

Table S1. Absorption and photoluminescence data for cations of ***NCH^{NMe2}N*** and **(*NC^{Me2}N*)BiX$_2$** (X = Cl, I) in CH_2Cl_2 at r.t.

	λ_{max} (nm) [ε_λ] ($\times10^{-3}\cdot M^{-1}cm^{-1}$) [a]	λ_{Em} (nm) [a]	λ_{Exc} (nm) [a]
NCH^{NMe2}N^{+}	393 (7.5)<br>590 (2.8)<br>1063 (14)	1165	300, 385, 604
**[*NCH^{NMe2}N*]$^{2+}$**	596 (3.3)<br>769 (14)	-	-
**[(*NC^{NMe2}N*)BiCl$_2$]$^{+}$**	393 (9.2)<br>590 (3.1)<br>1104 (17)	1200	314, 395, 602
**[(*NC^{NMe2}N*)BiCl$_2$]$^{2+}$**	367 (4.5)<br>405 (3.7)<br>605 (5.2)<br>778 (23)	-	-
**[(*NC^{NMe2}N*)$_2$BiI$_2$]$^{+}$**	376 (17)<br>601 (5.0)<br>1110 (23)	1212	315, 390, 612
**[(*NC^{NMe2}N*)BiI$_2$]$^{3+}$**	329 (8.9)<br>373 (7.3)<br>604 (7.6)<br>769 (33)	-	-

[a] in degassed CH_2Cl_2 at r.t.

TD-DFT Calculations

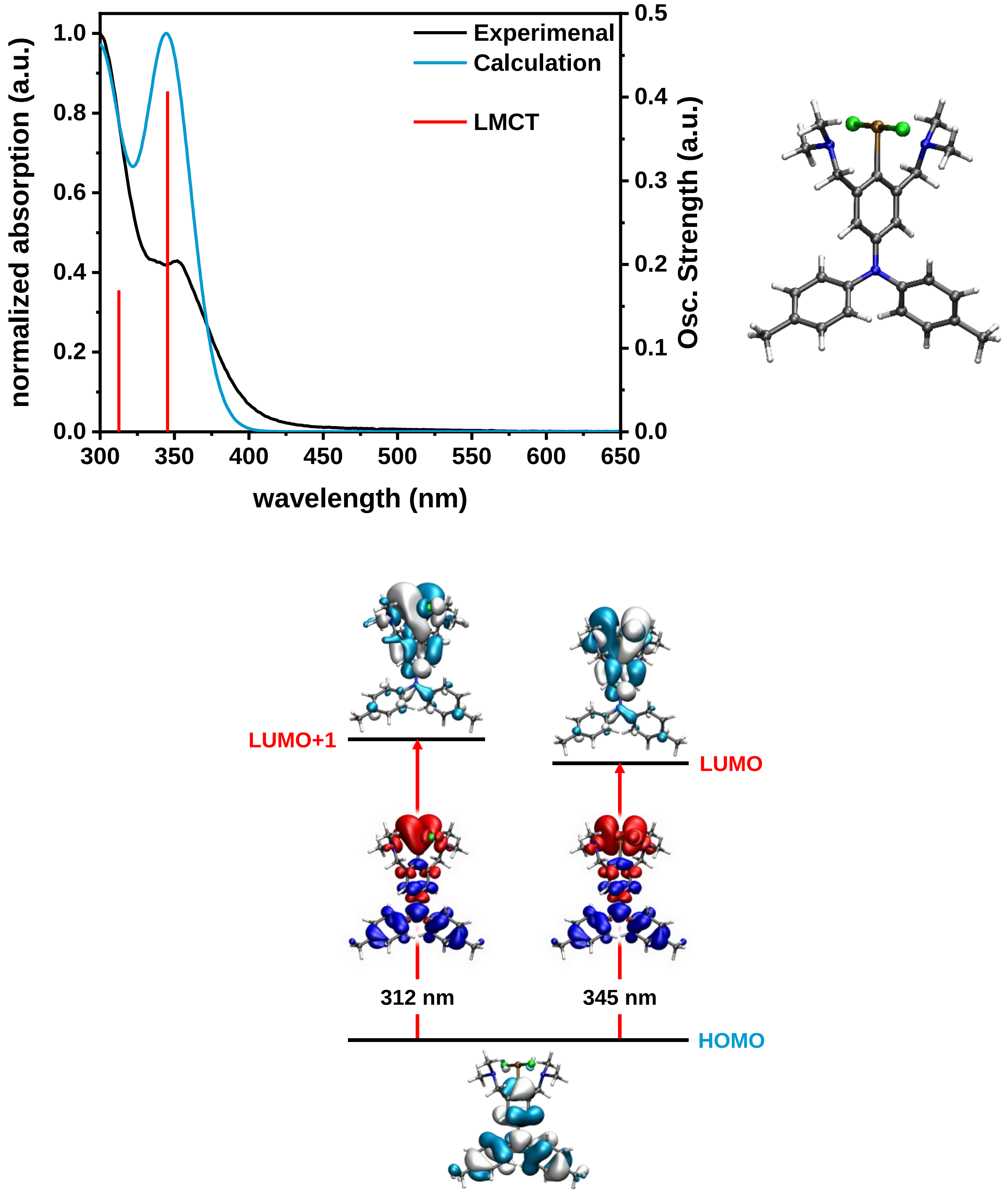

Figure S35. Left: Comparison of the experimental (black line) and the TD-DFT-calculated (blue line) absorption spectra of **(*NC*^{Me}*N*)BiCl$_2$** in THF. Individual LMCT transitions are indicated by red bars. Right: Geometry-optimized structure of **(*NC*^{Me}*N*)BiCl$_2$**. Bottom: TD-DFT-calculated transitions of **(*NC*^{Me}*N*)BiCl$_2$** with the mainly contributing molecular orbitals and the electron density difference maps (EDDMs). Blue color indicates a loss and red color a gain of electron density during the corresponding excitation.

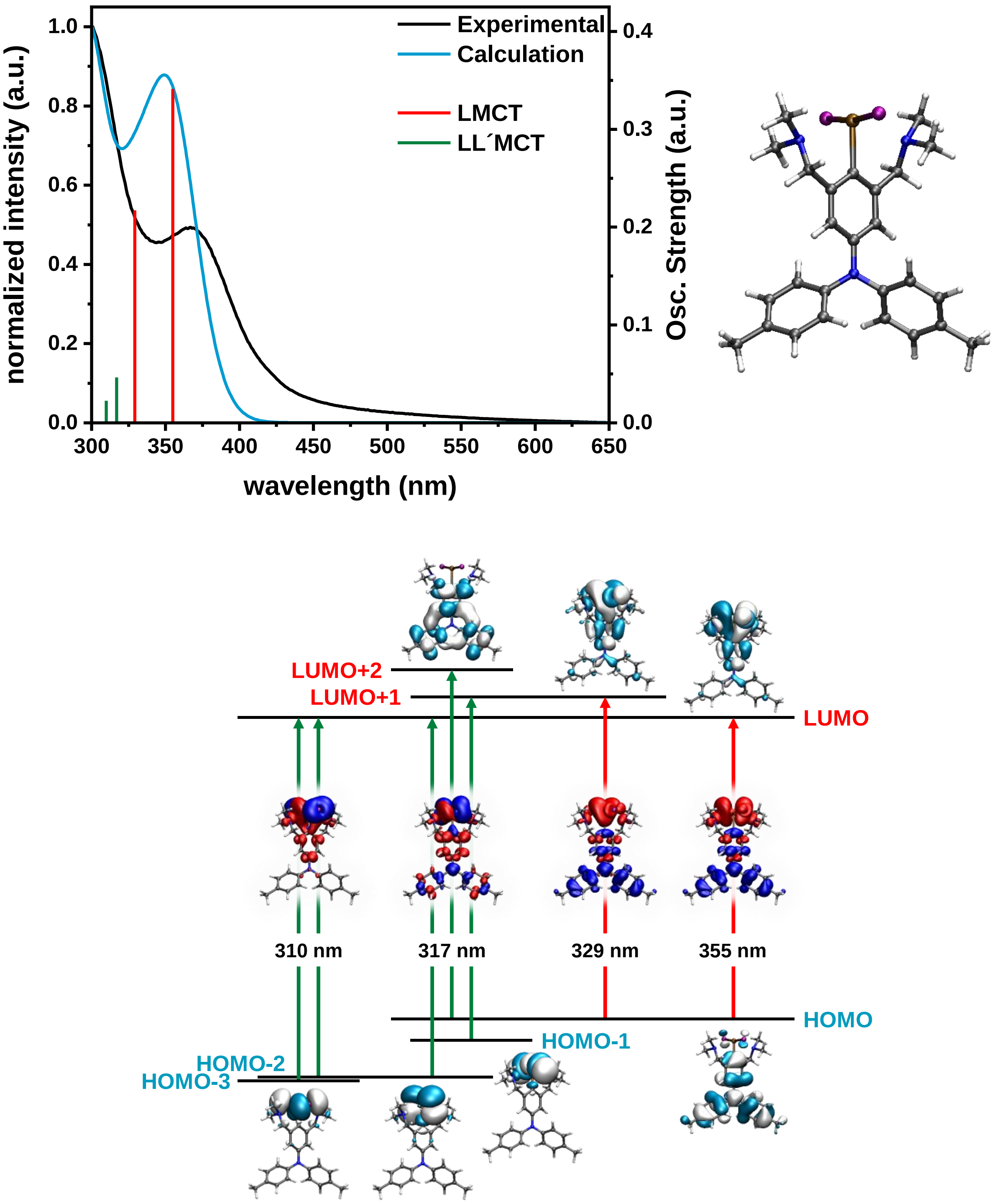

Figure S36. Left: Comparison of the experimental (black line) and the TD-DFT-calculated (blue line) absorption spectra of **(*NC*^{Me}*N*)BiI$_2$** in THF. Individual transitions are indicated by red bars for LMCT and green bars for LL´MCT. Right: Geometry-optimized structure of **(*NC*^{Me}*N*)BiI$_2$**. Bottom: TD-DFT-calculated transitions of **(*NC*^{Me}*N*)BiI$_2$** with the mainly contributing molecular orbitals and the electron density difference maps (EDDMs). Blue color indicates a loss and red color a gain of electron density during the corresponding excitation. LMCT transitions are indicated by red color and LL´MCT transitions by green color.

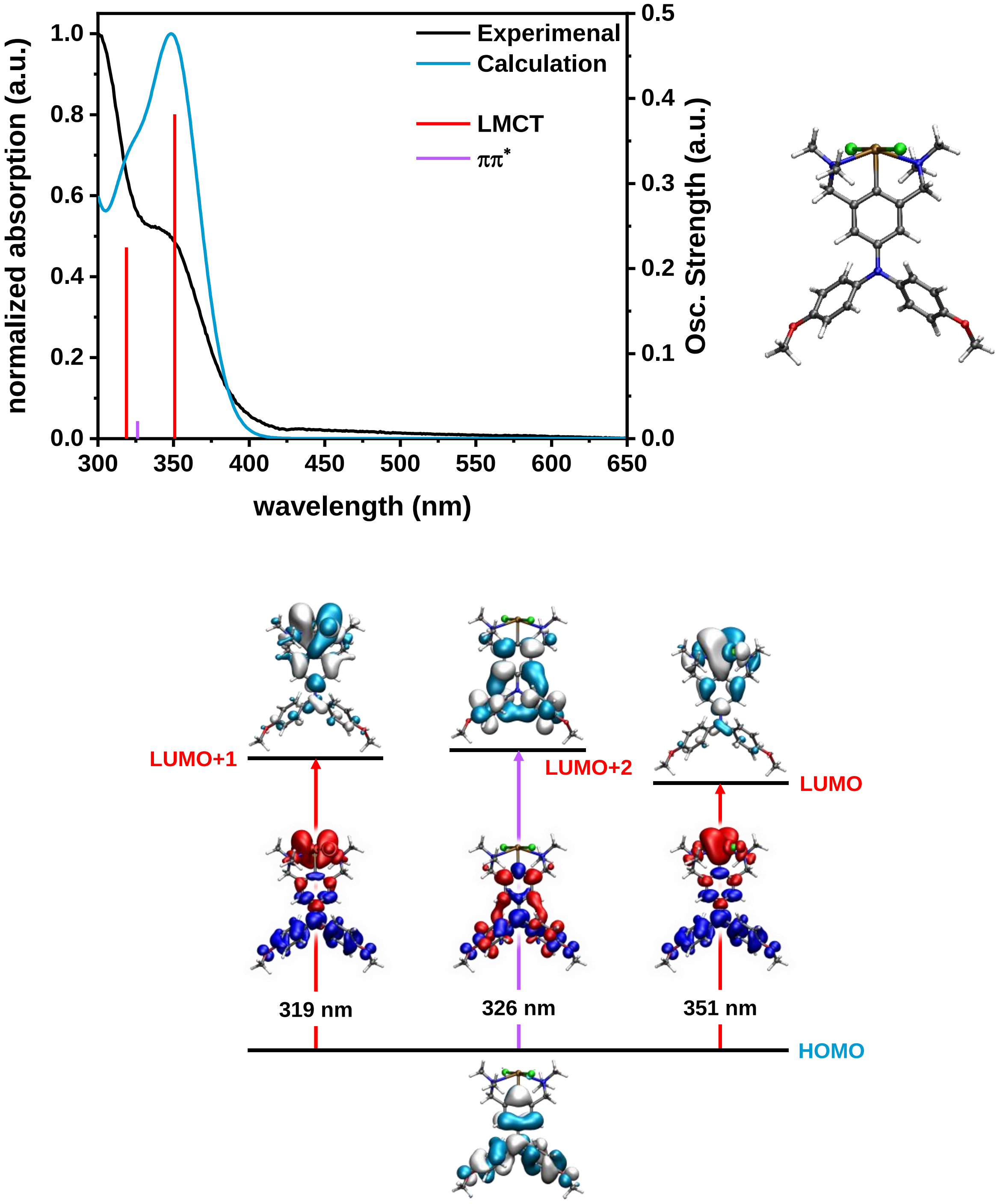

Figure S37. Left: Comparison of the experimental (black line) and the TD-DFT-calculated (blue line) absorption spectra of **(*NC*^{OMe}*N*)BiCl$_2$** in THF. Individual transitions are indicated by red bars for LMCT and purple bars for ππ*. Right: Geometry-optimized structure of **(*NC*^{OMe}*N*)BiCl$_2$**. Bottom: TD-DFT-calculated transitions of **(*NC*^{OMe}*N*)BiCl$_2$** with the mainly contributing molecular orbitals and the electron density difference maps (EDDMs). Blue color indicates a loss and red color a gain of electron density during the corresponding excitation. LMCT transitions are indicated by red color and ππ* transitions by purple color.

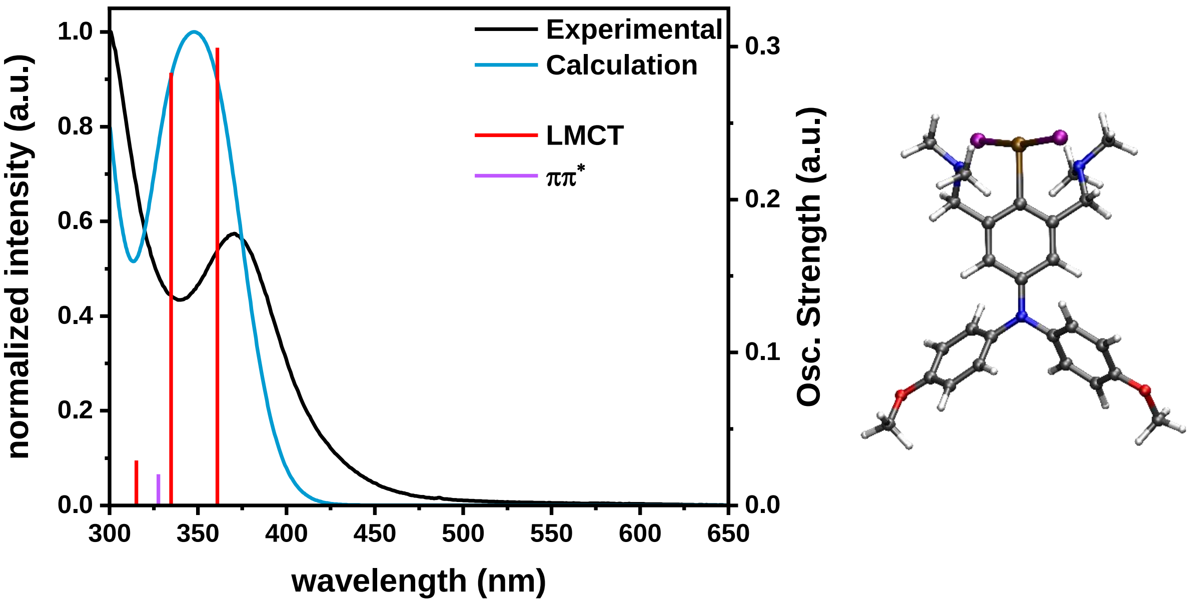

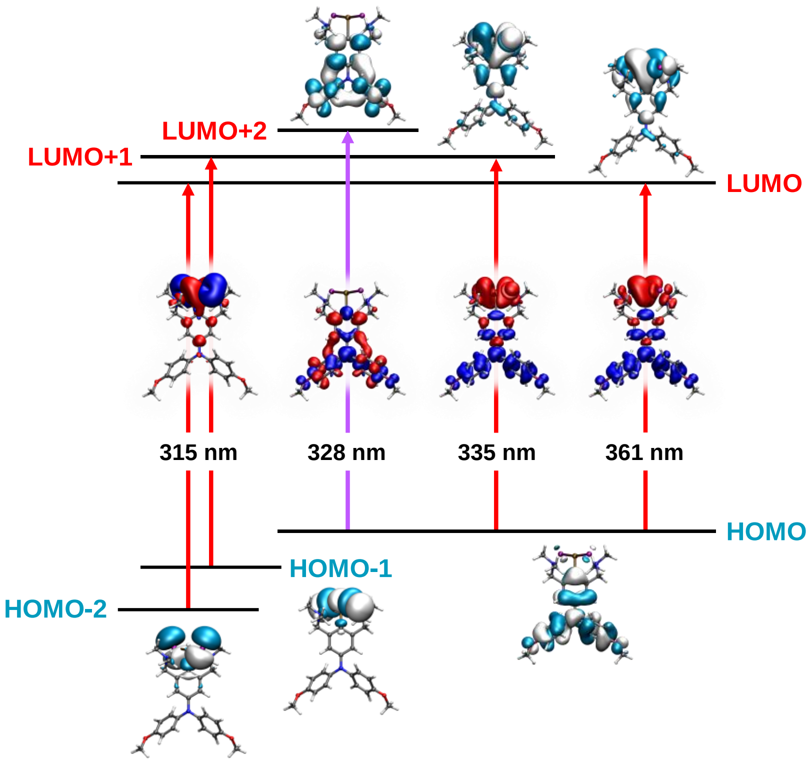

Figure S38. Left: Comparison of the experimental (black line) and the TD-DFT-calculated (blue line) absorption spectra of **(*NC*^{OMe}*N*)BiI$_2$** in THF. Individual transitions are indicated by red bars for LMCT and purple bars for $\pi\pi^*$. Right: Geometry-optimized structure of **(*NC*^{OMe}*N*)BiI$_2$**. Bottom: TD-DFT-calculated transitions of **(*NC*^{OMe}*N*)BiI$_2$** along with the mainly contributing molecular orbitals and the electron density difference maps (EDDMs). Blue color indicates a loss and red color a gain of electron density during the corresponding excitation. LMCT transitions are indicated by red color and $\pi\pi^*$ transitions by purple color.

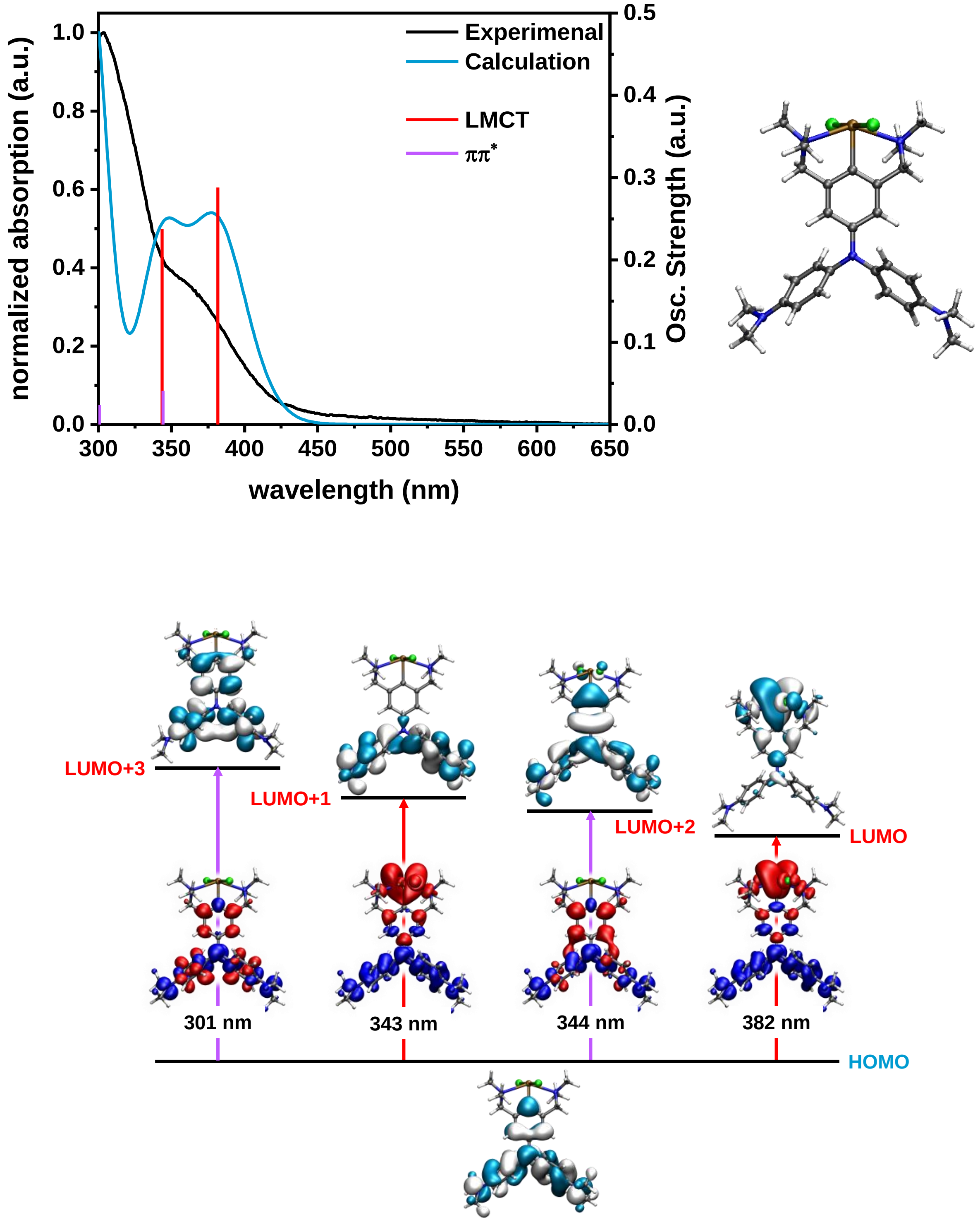

Figure S39. Left: Comparison of the experimental (black line) and the TD-DFT-calculated (blue line) absorption spectra of **($NC^{NMe2}N$)BiCl$_2$** in THF. Individual transitions are indicated by red bars for LMCT and purple bars for $\pi\pi^*$. Right: Geometry-optimized structure of **($NC^{NMe2}N$)BiCl$_2$**. Bottom: TD-DFT-calculated transitions of **($NC^{NMe2}N$)BiCl$_2$** along with the mainly contributing molecular orbitals and the electron density difference maps (EDDMs). Blue color indicates a loss and red color a gain of electron density during the corresponding excitation. LMCT transitions are indicated by red color and $\pi\pi^*$ transitions by purple color.

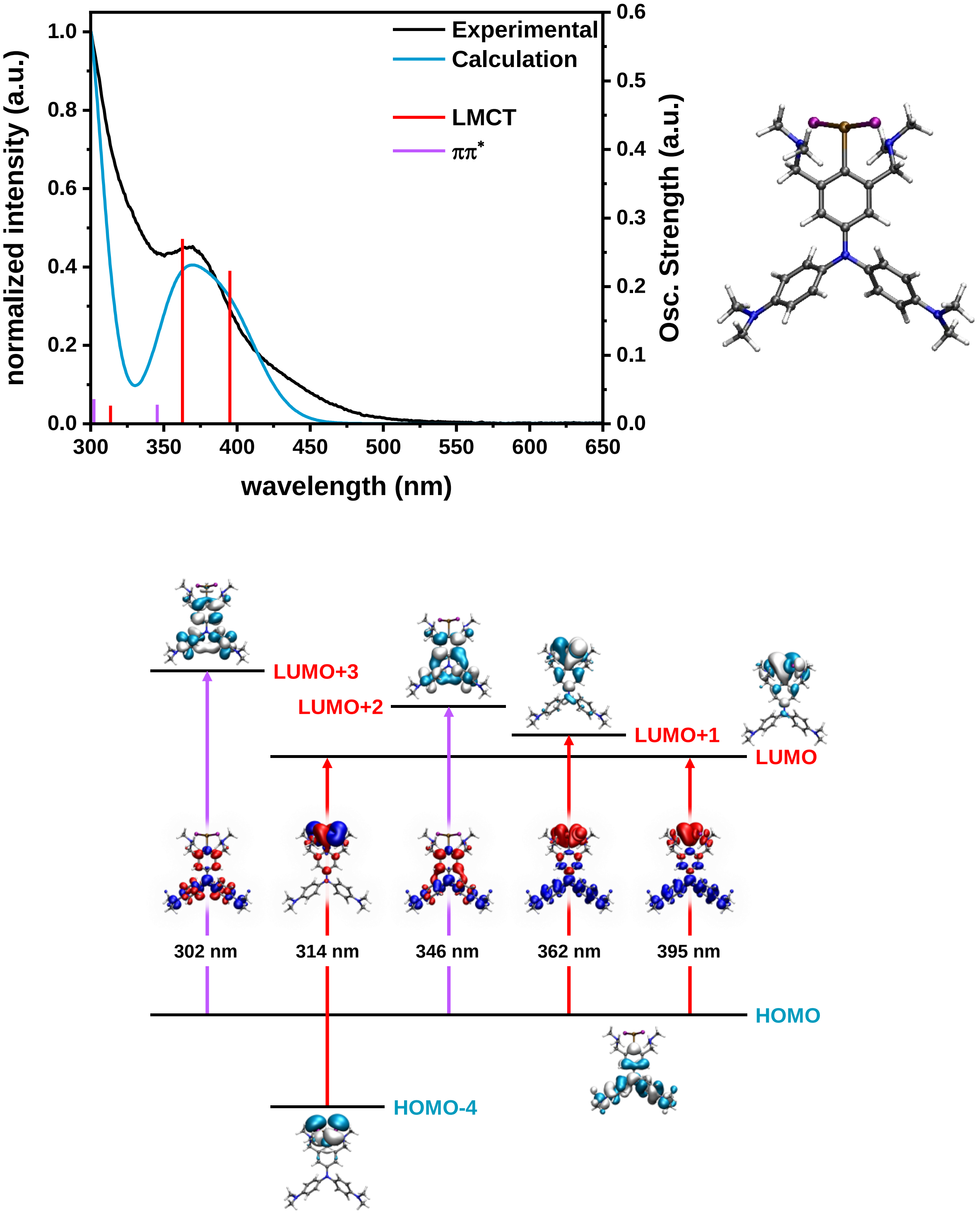

Figure S40. Left: Comparison of the experimental (black line) and the TD-DFT-calculated (blue line) absorption spectra of **(*NC*^{NMe2}*N*)BiI$_2$** in THF. Individual transitions are indicated by red bars for LMCT and purple bars for ππ*. Right: Geometry-optimized structure of **(*NC*^{NMe2}*N*)BiI$_2$**. Bottom: TD-DFT-calculated transitions of **(*NC*^{NMe2}*N*)BiI$_2$** along with the mainly contributing molecular orbitals and the electron density difference maps (EDDMs). Blue color indicates a loss and red color a gain of electron density during the corresponding excitation. LMCT transitions are indicated by red color and ππ* transitions by purple color.

Table S2. XYZ coordinates of the DFT-optimized structure of **(*NC*^{Me}*N*)BiCl$_2$** (solvent = THF).

Bi	-3.18261	-0.00000	0.00000
C	-0.99235	-0.00000	-0.00000
N	-2.39553	2.37158	-0.56918
C	-3.33839	3.06764	-1.45172
H	-3.52110	2.45950	-2.34107
H	-2.94217	4.04741	-1.75447
H	-4.28281	3.22121	-0.92229
N	-2.39553	-2.37158	0.56918
C	-2.19787	3.13780	0.66803
H	-3.14742	3.23344	1.19931
H	-1.80943	4.14036	0.43687
H	-1.49015	2.61675	1.31602
C	-1.10287	2.15192	-1.24755
H	-0.54085	3.09611	-1.30053
H	-1.32620	1.83813	-2.27402
C	-2.19788	-3.13780	-0.66803
H	-3.14742	-3.23344	-1.19931
H	-1.80943	-4.14036	-0.43687
H	-1.49015	-2.61675	-1.31602
C	-1.10287	-2.15192	1.24755
H	-1.32620	-1.83813	2.27402
H	-0.54085	-3.09611	1.30053
C	-3.33839	-3.06764	1.45172
H	-3.52110	-2.45950	2.34107
H	-2.94217	-4.04741	1.75447
H	-4.28281	-3.22121	0.92229
Cl	-3.19917	0.40706	2.66918
Cl	-3.19917	-0.40706	-2.66918
C	1.79985	-0.00000	-0.00000
C	1.08905	1.07092	-0.56918
C	-0.30139	1.06814	-0.56923
C	-0.30139	-1.06814	0.56923
C	1.08905	-1.07092	0.56918
H	1.63464	1.89438	-1.02146
H	1.63464	-1.89438	1.02146
N	3.20242	-0.00000	0.00000
C	3.92408	1.21716	0.07952
C	3.56619	2.20413	1.00512
C	5.02391	1.44382	-0.75389
C	4.28463	3.39158	1.07724
H	2.72459	2.03441	1.67077
C	5.74527	2.62866	-0.65759
H	5.31491	0.68410	-1.47366
C	5.38796	3.62990	0.25055
H	3.99087	4.14353	1.80661
H	6.60004	2.78162	-1.31287
C	3.92408	-1.21716	-0.07952
C	5.02391	-1.44382	0.75389
C	3.56619	-2.20413	-1.00512
C	5.74527	-2.62866	0.65759
H	5.31491	-0.68410	1.47366
C	4.28463	-3.39158	-1.07724
H	2.72459	-2.03441	-1.67077
C	5.38796	-3.62990	-0.25055
H	6.60004	-2.78162	1.31287
H	3.99088	-4.14353	-1.80661
C	6.14696	4.92567	0.32211
H	7.18081	4.80302	-0.01603
H	5.68465	5.69185	-0.31391
H	6.16650	5.32208	1.34272
C	6.14696	-4.92567	-0.32211
H	7.18082	-4.80302	0.01601
H	5.68467	-5.69184	0.31393
H	6.16648	-5.32209	-1.34271

Table S3. XYZ coordinates of the DFT-optimized structure of **(*NC^Me^N*)BiI$_2$** (solvent = THF).

Bi	-2.56637	0.00000	0.00000
C	-0.36623	-0.00000	0.00000
N	-1.74074	2.14200	-1.21861
C	-2.68042	2.60111	-2.24626
H	-2.89639	1.78135	-2.93616
H	-2.26491	3.45106	-2.80688
H	-3.61066	2.91848	-1.76698
N	-1.74074	-2.14200	1.21861
C	-1.48310	3.22426	-0.26194
H	-2.41394	3.51196	0.23119
H	-1.06259	4.09827	-0.78174
H	-0.77738	2.88445	0.49893
C	-0.47195	1.69368	-1.82271
H	0.10551	2.56056	-2.17785
H	-0.73004	1.08153	-2.69618
C	-1.48310	-3.22426	0.26194
H	-2.41394	-3.51196	-0.23119
H	-1.06259	-4.09827	0.78174
H	-0.77738	-2.88445	-0.49893
C	-0.47195	-1.69368	1.82271
H	-0.73004	-1.08153	2.69618
H	0.10551	-2.56056	2.17785
C	-2.68042	-2.60111	2.24626
H	-2.89639	-1.78135	2.93616
H	-2.26491	-3.45106	2.80688
H	-3.61066	-2.91848	1.76698
I	-2.76241	1.32744	2.76682
I	-2.76241	-1.32744	-2.76682
C	2.42815	-0.00000	-0.00000
C	1.71782	0.85642	-0.85755
C	0.32699	0.85444	-0.85736
C	0.32699	-0.85444	0.85736
C	1.71782	-0.85642	0.85755
H	2.26383	1.51180	-1.53068
H	2.26383	-1.51180	1.53068
N	3.83262	-0.00000	-0.00000
C	4.55039	1.19809	-0.23504
C	4.17402	2.39290	0.38929
C	5.66283	1.20492	-1.08322
C	4.88685	3.56268	0.15412
H	3.32233	2.39962	1.06363
C	6.37785	2.37845	-1.29424
H	5.96843	0.28422	-1.57188
C	6.00244	3.58221	-0.68964
H	4.57806	4.47878	0.65321
H	7.24226	2.35864	-1.95453
C	4.55039	-1.19809	0.23504
C	5.66283	-1.20492	1.08322
C	4.17402	-2.39290	-0.38929
C	6.37785	-2.37845	1.29424
H	5.96843	-0.28422	1.57188
C	4.88685	-3.56268	-0.15412
H	3.32233	-2.39962	-1.06363
C	6.00244	-3.58221	0.68964
H	7.24226	-2.35864	1.95453
H	4.57806	-4.47878	-0.65321
C	6.75583	4.85611	-0.95408
H	7.80755	4.65863	-1.18513
H	6.33136	5.39822	-1.80925
H	6.71678	5.53019	-0.09209
C	6.75583	-4.85611	0.95408
H	7.80755	-4.65863	1.18513
H	6.33136	-5.39822	1.80925
H	6.71678	-5.53019	0.09209

Table S4. XYZ coordinates of the DFT-optimized structure of **(*NC*^{OMe}*N*)BiCl$_2$** (solvent = THF).

Bi	3.52187	0.00004	-0.00032
Cl	3.54056	-1.74119	2.06530
Cl	3.53886	1.74096	-2.06548
N	2.73227	-1.99133	-1.40763
N	2.73212	1.99173	1.40809
N	-2.85916	-0.00005	0.00026
C	1.33420	0.00008	0.00023
C	0.64198	-1.13605	-0.41525
C	-0.74800	1.13404	0.43108
H	-1.29160	2.00932	0.77529
C	-0.74794	-1.13409	-0.43049
H	-1.29147	-2.00943	-0.77467
C	-4.63888	-1.51479	-0.71337
H	-4.88557	-0.82669	-1.51694
C	-5.06434	-3.57859	0.46569
C	-1.46152	-0.00004	0.00028
C	0.64192	1.13615	0.41578
C	3.67630	3.11310	1.35651
H	4.61808	2.81419	1.82488
H	3.27897	3.98642	1.89330
H	3.86435	3.38245	0.31419
C	-5.38309	2.68244	0.55756
H	-6.19659	2.88451	1.24567
C	1.44330	-2.35870	-0.78913
H	1.67244	-2.92405	0.12190
H	0.87846	-3.02150	-1.46140
C	-3.58729	-1.20910	0.15049
C	-5.06436	3.57840	-0.46573
C	3.67666	-3.11255	-1.35586
H	4.61837	-2.81359	-1.82433
H	3.27942	-3.98602	-1.89246
H	3.86478	-3.38163	-0.31348
C	-3.28471	-2.10684	1.18400
H	-2.47537	-1.87899	1.87186
C	-4.00477	3.28017	-1.33222
H	-3.77180	3.97838	-2.13075
C	-3.58730	1.20897	-0.15017
C	2.52642	-1.56283	-2.79708
H	1.81804	-0.73238	-2.82588
H	2.13459	-2.39593	-3.39863
H	3.47328	-1.22495	-3.22400
C	-5.38300	-2.68253	-0.55754
H	-6.19645	-2.88453	-1.24573
O	-5.71386	4.74487	-0.70110
C	-4.00482	-3.28043	1.33229
H	-3.77189	-3.97872	2.13076
C	-4.63895	1.51474	0.71357
H	-4.88569	0.82671	1.51719
C	2.52653	1.56283	2.79742
H	1.81835	0.73220	2.82609
H	2.13455	2.39566	3.39927
H	3.47352	1.22506	3.22414
O	-5.71384	-4.74509	0.70087
C	1.44309	2.35891	0.78971
H	1.67208	2.92434	-0.12130
H	0.87813	3.02157	1.46202
C	-3.28465	2.10660	-1.18376
H	-2.47526	1.87869	-1.87154
C	-6.78760	-5.08296	-0.15276
H	-7.16587	-6.04221	0.20336
H	-6.45594	-5.18840	-1.19335
H	-7.59034	-4.33650	-0.10350
C	-6.78769	5.08283	0.15241
H	-7.16596	6.04202	-0.20389
H	-6.45612	5.18843	1.19301
H	-7.59041	4.33634	0.10320

Table S5. XYZ coordinates of the DFT-optimized structure of **(*NC^OMe^N*)BiI$_2$** (solvent = THF).

Bi	-2.86949	0.00000	0.00000
I	-3.07815	-2.51495	-1.75999
I	-3.07815	2.51495	1.75999
N	-2.04364	-1.55234	1.91383
N	-2.04364	1.55234	-1.91383
N	3.52568	-0.00000	-0.00000
C	-0.67237	0.00000	-0.00000
C	0.02272	-0.98976	0.69505
C	1.41296	0.98317	-0.70896
H	1.95599	1.74014	-1.26787
C	1.41296	-0.98317	0.70896
H	1.95599	-1.74014	1.26787
C	5.30984	-1.28440	1.06679
H	5.56178	-0.41404	1.66598
C	5.72713	-3.58243	0.45384
C	2.12702	0.00000	-0.00000
C	0.02272	0.98976	-0.69505
C	-2.98381	2.64019	-2.19990
H	-3.91393	2.21823	-2.59071
H	-2.56915	3.33217	-2.94739
H	-3.20016	3.18692	-1.27850
C	6.05235	2.45461	-1.21123
H	6.86964	2.47519	-1.92379
C	-0.77431	-2.07519	1.37430
H	-1.03060	-2.84278	0.63313
H	-0.19458	-2.56089	2.17382
C	4.25295	-1.20793	0.15957
C	5.72713	3.58243	-0.45384
C	-2.98381	-2.64019	2.19990
H	-3.91392	-2.21822	2.59072
H	-2.56915	-3.33217	2.94739
H	-3.20016	-3.18692	1.27850
C	3.94381	-2.34025	-0.60735
H	3.13018	-2.29477	-1.32568
C	4.66235	3.51416	0.45387
H	4.42400	4.39340	1.04509
C	4.25295	1.20793	-0.15957
C	-1.78649	-0.78209	3.13573
H	-1.07775	0.02086	2.92274
H	-1.36951	-1.43601	3.91643
H	-2.71667	-0.33810	3.49643
C	6.05235	-2.45462	1.21123
H	6.86964	-2.47519	1.92379
O	6.37469	4.77171	-0.52166
C	4.66235	-3.51416	-0.45387
H	4.42400	-4.39340	-1.04509
C	5.30984	1.28440	-1.06679
H	5.56178	0.41404	-1.66598
C	-1.78650	0.78209	-3.13573
H	-1.07776	-0.02086	-2.92274
H	-1.36952	1.43601	-3.91643
H	-2.71667	0.33810	-3.49642
O	6.37469	-4.77171	0.52166
C	-0.77431	2.07519	-1.37430
H	-1.03060	2.84278	-0.63313
H	-0.19458	2.56089	-2.17382
C	3.94381	2.34025	0.60735
H	3.13018	2.29477	1.32568
C	7.45238	-4.88230	1.42831
H	7.82818	-5.90147	1.32752
H	7.12571	-4.71765	2.46280
H	8.25552	-4.17484	1.18637
C	7.45238	4.88230	-1.42831
H	7.82818	5.90146	-1.32752
H	7.12571	4.71765	-2.46279
H	8.25552	4.17484	-1.18637

Table S6. XYZ coordinates of the DFT-optimized structure of **(*NC^{NMe2}N*)BiCl$_2$** (solvent = THF).

Bi	3.77799	-0.01569	0.01233
Cl	3.77681	-1.78659	2.05702
Cl	3.84288	1.75280	-2.03241
N	2.99797	-1.98608	-1.43049
N	2.98174	1.96253	1.43706
N	-2.59995	-0.00296	-0.03434
C	1.59406	-0.00763	-0.01107
C	0.90071	-1.13592	-0.44697
C	-0.49071	1.12342	0.42222
H	-1.03367	1.99597	0.77353
C	-0.48834	-1.13275	-0.46990
H	-1.02939	-2.00402	-0.82728
C	-4.35685	-1.46893	-0.90773
H	-4.57834	-0.72813	-1.67147
C	-4.84244	-3.62959	0.11494
C	-1.20750	-0.00396	-0.02757
C	0.89851	1.12260	0.41626
C	3.92811	3.08253	1.40782
H	4.86554	2.77602	1.88007
H	3.52794	3.94988	1.95228
H	4.12546	3.36453	0.37056
C	-5.09402	2.63988	0.78713
H	-5.86861	2.79218	1.53022
C	1.70126	-2.35634	-0.82998
H	1.91964	-2.93539	0.07511
H	1.14070	-3.00754	-1.51713
C	-3.33511	-1.21862	0.01097
C	-4.84637	3.62054	-0.19755
C	3.93698	-3.11179	-1.38560
H	4.88463	-2.81029	-1.84037
H	3.54156	-3.97594	-1.93853
H	4.11364	-3.39657	-0.34537
C	-3.08894	-2.18003	0.99520
H	-2.31300	-2.00040	1.73483
C	-3.80425	3.35994	-1.11356
H	-3.56013	4.08021	-1.88619
C	-3.33382	1.21350	-0.08305
C	2.80784	-1.53632	-2.81526
H	2.10287	-0.70283	-2.83824
H	2.41868	-2.35870	-3.43320
H	3.76021	-1.19584	-3.22768
C	-5.10505	-2.63654	-0.85300
H	-5.89634	-2.77586	-1.58081
N	-5.59672	4.77672	-0.26919
C	-3.81557	-3.36096	1.04582
H	-3.58811	-4.07187	1.83206
C	-4.34983	1.46992	0.84052
H	-4.56209	0.73659	1.61406
C	2.76317	1.51618	2.81870
H	2.05338	0.68649	2.82992
H	2.36704	2.34172	3.42795
H	3.70584	1.17134	3.24945
N	-5.55240	-4.81319	0.14595
C	1.69862	2.33931	0.81181
H	1.93721	2.91571	-0.08987
H	1.12914	2.99490	1.48737
C	-3.08141	2.17636	-1.06394
H	-2.29640	2.00114	-1.79501
C	-6.73807	-4.94148	-0.67042
H	-7.16418	-5.93589	-0.52614
H	-6.49597	-4.83923	-1.73484
H	-7.51036	-4.19572	-0.42335
C	-6.48833	5.10451	0.81986
H	-7.00255	6.03946	0.59040
H	-5.96350	5.22596	1.78075
H	-7.25499	4.33110	0.94760

C	-5.16218	5.84286	-1.14271
H	-5.14385	5.51325	-2.18828
H	-4.16005	6.22249	-0.88756
H	-5.86941	6.67117	-1.07341
C	-5.41533	-5.68415	1.29113
H	-4.37868	-6.02192	1.40641
H	-6.03290	-6.57126	1.13994
H	-5.72443	-5.20461	2.23350

Table S7. XYZ coordinates of the DFT-optimized structure of **(*NC*^{NMe2}*N*)BiI$_2$** (solvent = THF).

Bi	-3.09414	0.00000	-0.00000
I	-3.31579	2.43627	1.87063
I	-3.31579	-2.43627	-1.87063
N	-2.26919	1.63633	-1.84273
N	-2.26919	-1.63633	1.84273
N	3.29758	0.00000	0.00000
C	-0.90038	0.00000	0.00000
C	-0.20375	1.02054	-0.64806
C	1.18583	-1.01665	0.66018
H	1.72818	-1.80128	1.17994
C	1.18583	1.01665	-0.66018
H	1.72818	1.80128	-1.17994
C	5.05965	1.29680	-1.10052
H	5.28293	0.43810	-1.72811
C	5.54255	3.59936	-0.45590
C	1.90366	0.00000	0.00000
C	-0.20375	-1.02054	0.64806
C	-3.20890	-2.73526	2.08357
H	-4.13793	-2.33058	2.49501
H	-2.79248	-3.45942	2.79902
H	-3.42790	-3.24135	1.13983
C	5.80315	-2.45937	1.24624
H	6.58829	-2.47809	1.99363
C	-1.00140	2.13547	-1.27718
H	-1.26139	2.86726	-0.50179
H	-0.42161	2.65890	-2.05264
C	4.03061	1.20654	-0.16009
C	5.54255	-3.59936	0.45590
C	-3.20890	2.73526	-2.08357
H	-4.13793	2.33058	-2.49501
H	-2.79248	3.45942	-2.79902
H	-3.42790	3.24135	-1.13983
C	3.76473	2.32903	0.62900
H	2.96939	2.28568	1.36852
C	4.48707	-3.50415	-0.47652
H	4.23153	-4.35017	-1.10441
C	4.03061	-1.20654	0.16009
C	-2.00898	0.92018	-3.09637
H	-1.30332	0.10647	-2.91671
H	-1.58704	1.60687	-3.84565
H	-2.93892	0.49545	-3.48017
C	5.80315	2.45937	-1.24624
H	6.58829	2.47809	-1.99363
N	6.29385	-4.75116	0.58064
C	4.48707	3.50415	0.47652
H	4.23153	4.35017	1.10441
C	5.05965	-1.29680	1.10052
H	5.28293	-0.43810	1.72811
C	-2.00898	-0.92018	3.09637
H	-1.30332	-0.10647	2.91671
H	-1.58704	-1.60687	3.84565
H	-2.93892	-0.49545	3.48017
N	6.29385	4.75116	-0.58064
C	-1.00140	-2.13547	1.27718

H	-1.26139	-2.86726	0.50179
H	-0.42161	-2.65890	2.05264
C	3.76473	-2.32903	-0.62900
H	2.96939	-2.28568	-1.36852
C	7.19167	4.88417	-1.70533
H	7.70333	5.84607	-1.64111
H	6.67258	4.83324	-2.67570
H	7.96005	4.10220	-1.69038
C	7.19167	-4.88417	1.70533
H	7.70333	-5.84607	1.64111
H	6.67258	-4.83324	2.67570
H	7.96005	-4.10220	1.69038
C	5.84702	-5.95541	-0.08174
H	5.80931	-5.81630	-1.16861
H	4.85070	-6.28420	0.25444
H	6.55837	-6.75818	0.12027
C	5.84702	5.95541	0.08174
H	4.85070	6.28420	-0.25444
H	6.55837	6.75818	-0.12027
H	5.80931	5.81630	1.16861

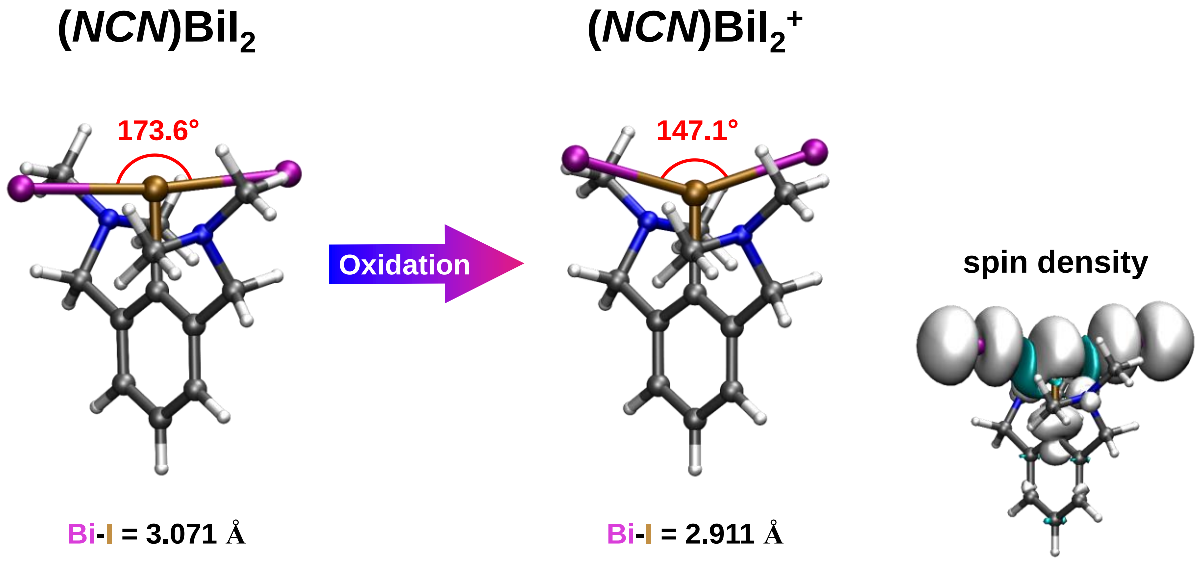

Figure S41. Structural reorganization of **(*NCN*)BiI$_2$** upon oxidation.

Table S8. XYZ coordinates of the DFT-optimized structure of **(*NCN*)BiI$_2$** (solvent = CH_2Cl_2).

Bi	0.00005	-0.00012	-0.64056
I	-3.05125	0.30214	-0.81293
I	3.05111	-0.30248	-0.81283
N	-0.40998	-2.42963	0.17914
C	-0.51844	-1.09986	2.25165
N	0.41010	2.42970	0.17822
C	0.00003	0.00031	1.56878
C	-0.00004	0.00085	4.33721
H	-0.00008	0.00107	5.42378
C	-0.50904	-1.09883	3.64743
H	-0.90385	-1.95089	4.19656
C	0.51847	1.10074	2.25124
C	-1.13026	-2.22009	1.44918
H	-1.15970	-3.15726	2.02521
H	-2.16332	-1.95224	1.19376
C	0.85841	-3.12206	0.43420
H	1.45864	-2.54581	1.14129
H	1.41911	-3.22241	-0.49739
H	0.66716	-4.12167	0.85232
C	0.50899	1.10026	3.64703
H	0.90377	1.95254	4.19584
C	1.21956	3.20836	-0.76473
H	0.65916	3.34299	-1.69403
H	2.14606	2.67042	-0.98108
H	1.45988	4.19860	-0.35120
C	1.13032	2.22066	1.44837
H	1.15973	3.15806	2.02404
H	2.16339	1.95272	1.19310
C	-0.85833	3.12219	0.43295
H	-0.66714	4.12197	0.85069
H	-1.45857	2.54620	1.14024
H	-1.41899	3.22214	-0.49870
C	-1.21942	-3.20861	-0.76356
H	-1.45985	-4.19867	-0.34965
H	-0.65897	-3.34366	-1.69276
H	-2.14588	-2.67070	-0.98019

Table S9. XYZ coordinates of the DFT-optimized structure of **[(*NCN*)BiI$_2$]$^+$** (solvent = CH_2Cl_2).

Bi	0.00080	-0.38734	-0.00050
I	-2.77324	-1.21497	-0.30212
I	2.77667	-1.20895	0.30083
N	-0.37131	0.37407	2.40984
C	-0.52612	2.46163	1.10784
N	0.37012	0.37896	-2.40952
C	-0.00244	1.80148	0.00140
C	-0.00724	4.54300	0.00324
H	-0.00914	5.62907	0.00397
C	-0.52063	3.85679	1.10400
H	-0.91812	4.40721	1.95305
C	0.51879	2.46494	-1.10421
C	-1.11436	1.63898	2.22787
H	-1.12820	2.20339	3.17012
H	-2.15297	1.37990	1.98676
C	0.92595	0.64089	3.05086
H	1.50290	1.34579	2.44764
H	1.48718	-0.29030	3.14747
H	0.76911	1.07071	4.04942
C	0.50847	3.86006	-1.09848
H	0.90395	4.41299	-1.94685
C	1.13878	-0.57464	-3.22102
H	0.56849	-1.50180	-3.31935
H	2.09330	-0.79156	-2.73632
H	1.32771	-0.16448	-4.22194
C	1.10934	1.64588	-2.22570
H	1.12090	2.21193	-3.16699
H	2.14885	1.38973	-1.98544
C	-0.92773	0.64294	-3.05055
H	-0.77176	1.07467	-4.04842
H	-1.50687	1.34532	-2.44650
H	-1.48626	-0.28972	-3.14874
C	-1.13717	-0.58247	3.22051
H	-1.32629	-0.17406	4.22210
H	-0.56477	-1.50849	3.31714
H	-2.09151	-0.80093	2.73615

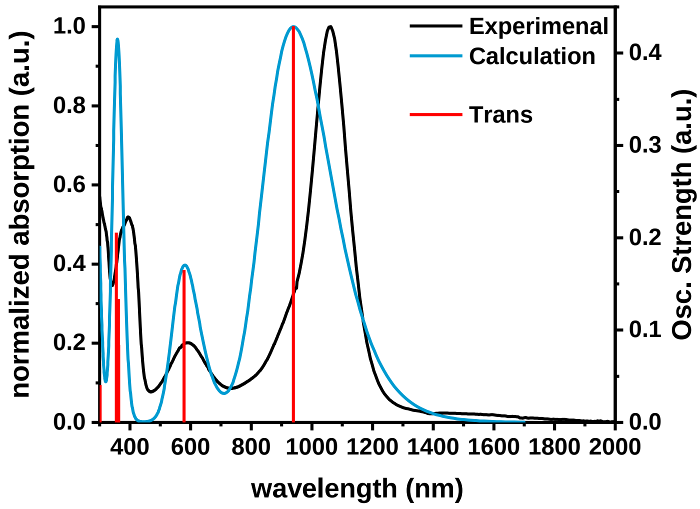

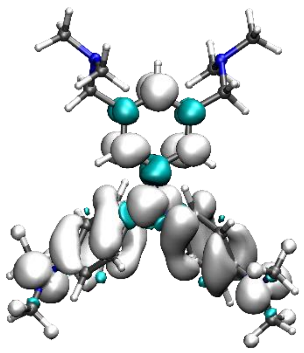

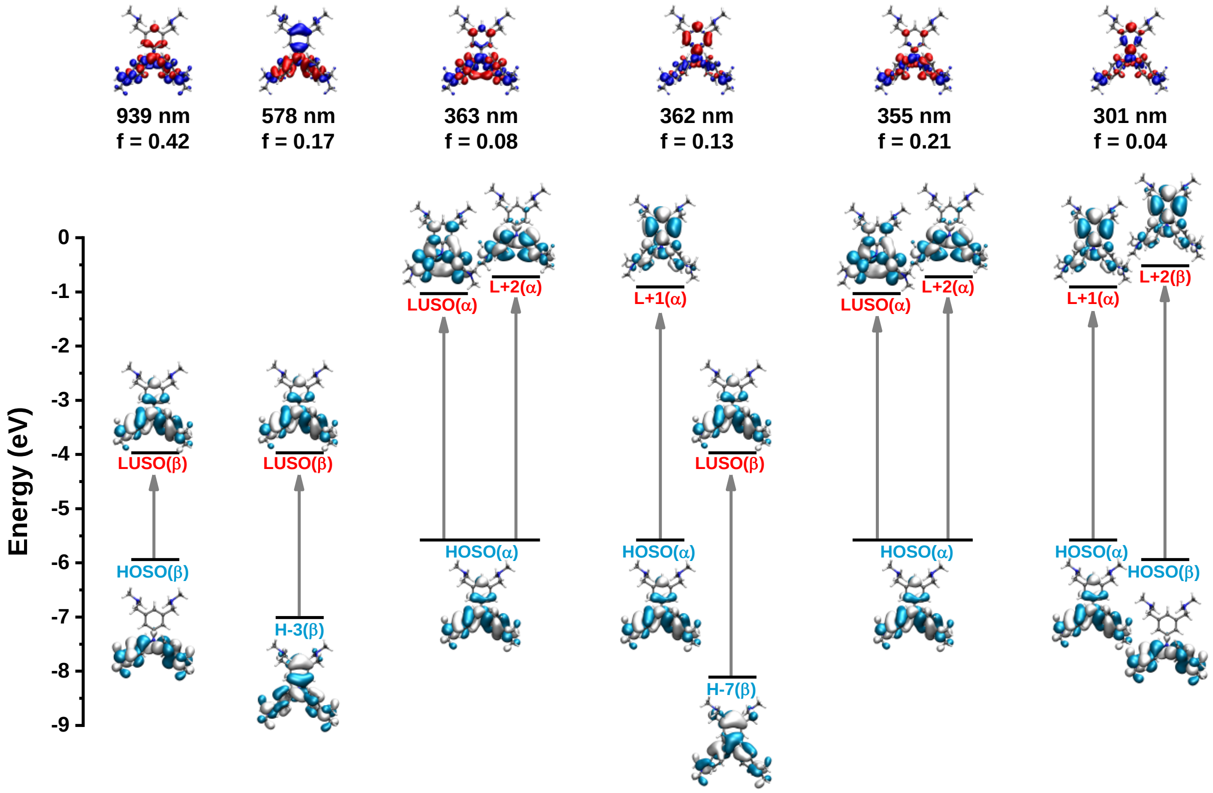

Figure S42. Left: Comparison of the experimental (black line) and the TD-DFT-calculated (blue line) absorption spectra of **[*NCH*^{NMe2}*N*]$^+$** in CH_2Cl_2. Transitions are indicated by red bars. Right: Spin-density of **[*NCH*^{NMe2}*N*]$^+$**. Bottom: TD-DFT-calculated transitions of **[*NCH*^{NMe2}*N*]$^+$** along with the mainly contributing molecular orbitals and the electron density difference maps (EDDMs). Blue color indicates a loss and red color a gain of electron density during the corresponding excitation.

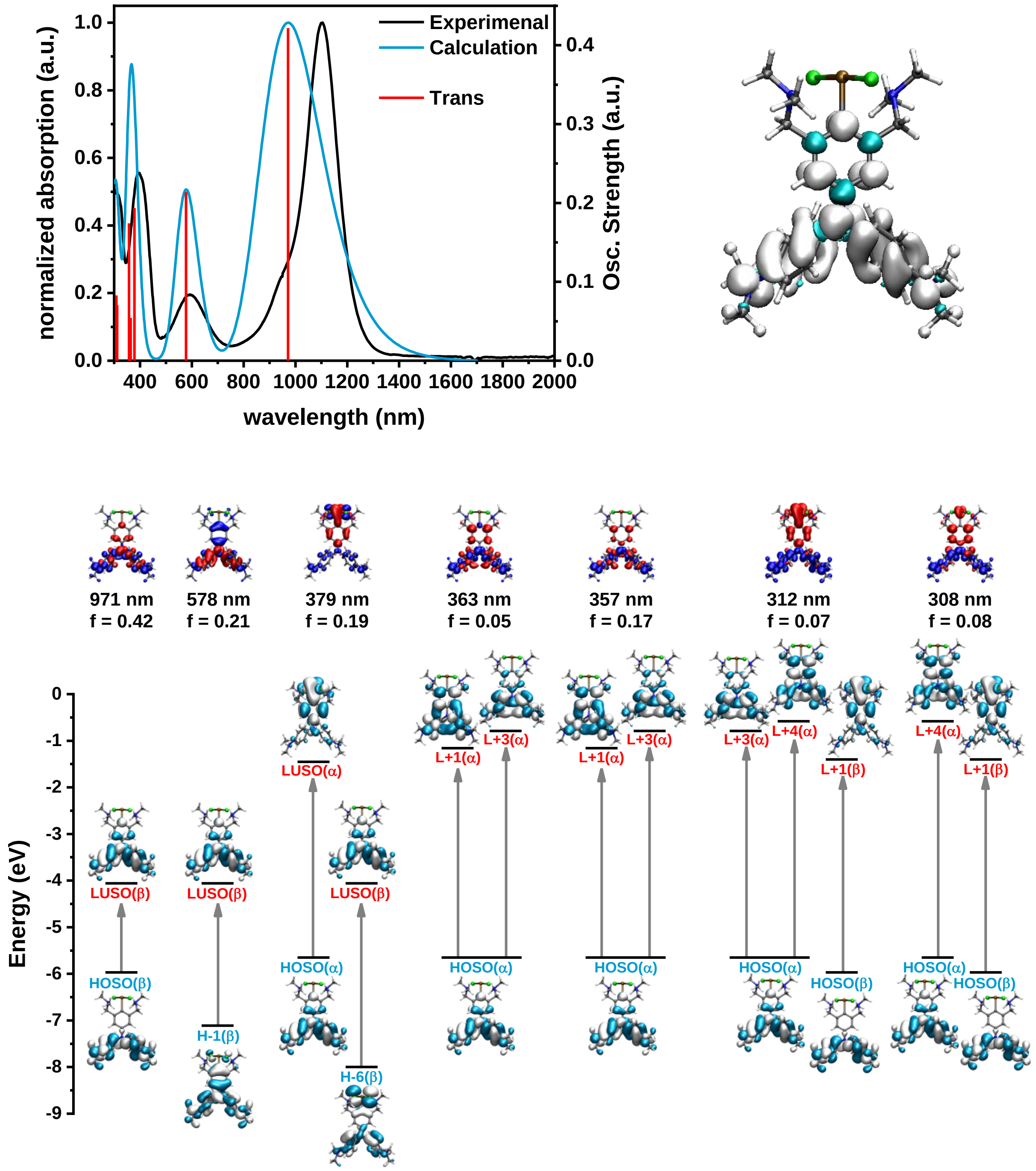

Figure S43. Left: Comparison of the experimental (black line) and the TD-DFT-calculated (blue line) absorption spectra of **[(*NC*^{NMe2}*N*)BiCl$_2$]$^+$** in CH_2Cl_2. Transitions are indicated by red bars. Right: Spin-density of **[(*NC*^{NMe2}*N*)BiCl$_2$]$^+$**. Bottom: TD-DFT-calculated transitions of **[(*NC*^{NMe2}*N*)BiCl$_2$]$^+$** along with the mainly contributing molecular orbitals and the electron density difference maps (EDDMs). Blue color indicates a loss and red color a gain of electron density during the corresponding excitation.

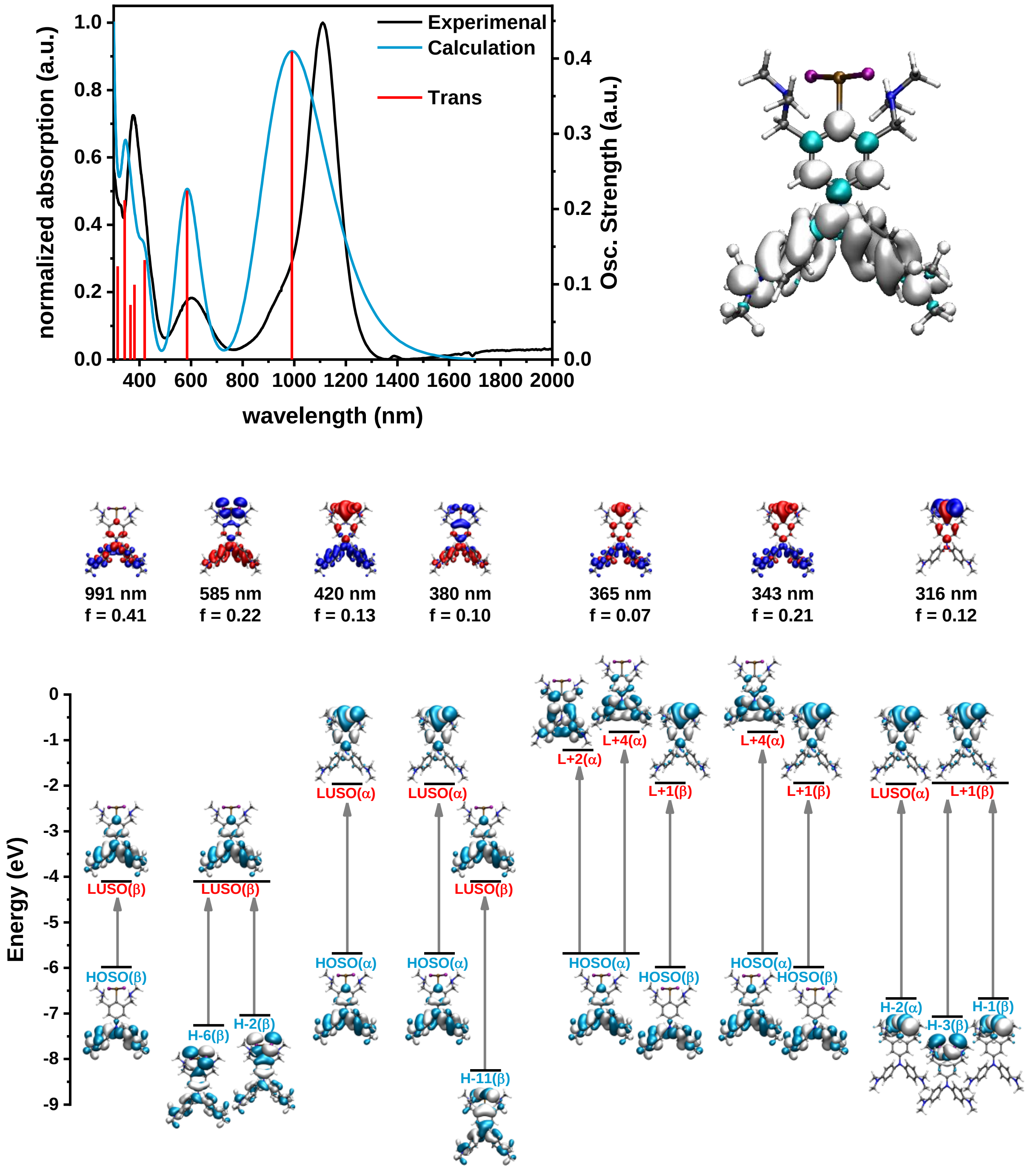

Figure S44. Left: Comparison of the experimental (black line) and the TD-DFT-calculated (blue line) absorption spectra of **[(*NC*^{NMe2}*N*)BiI$_2$]$^+$** in CH_2Cl_2. Transitions are indicated by red bars. Right: Spin-density of **[(*NC*^{NMe2}*N*)BiI$_2$]$^+$**. Bottom: TD-DFT-calculated transitions of **[(*NC*^{NMe2}*N*)BiI$_2$]$^+$** along with the mainly contributing molecular orbitals and the electron density difference maps (EDDMs). Blue color indicates a loss and red color a gain of electron density during the corresponding excitation.

Table S10. XYZ coordinates of the DFT-optimized structure of **[*NCH*^{NMe2}*N*]**$^{+}$ (solvent = CH_2Cl_2).

H	4.46526	-0.00008	0.00023
N	4.62183	-1.47000	-2.21357
N	4.62233	1.47005	2.21310
N	-0.81087	0.00003	0.00010
C	3.37943	-0.00009	0.00016
C	2.69527	-0.95711	-0.75362
C	1.30274	0.94881	0.75739
H	0.74994	1.66533	1.35898
C	1.30272	-0.94883	-0.75718
H	0.74989	-1.66533	-1.35877
C	-2.71315	-1.43419	-0.52682
H	-3.10393	-0.67601	-1.19810
C	-2.89504	-3.65416	0.45994
C	0.60927	0.00001	0.00009
C	2.69529	0.95698	0.75390
C	5.48838	2.53536	2.67925
H	6.38122	2.10389	3.14386
H	5.00241	3.19454	3.42399
H	5.80761	3.15371	1.83348
C	-3.39203	2.62223	0.38045
H	-4.30797	2.76688	0.94051
C	3.45707	-2.00464	-1.53209
H	3.80890	-2.77034	-0.82710
H	2.76552	-2.51886	-2.22899
C	-1.49775	-1.20517	0.14809
C	-2.89482	3.65434	-0.45966
C	5.48814	-2.53512	-2.67969
H	6.38059	-2.10344	-3.14486
H	5.00214	-3.19480	-3.42397
H	5.80800	-3.15301	-1.83382
C	-0.98704	-2.22752	0.97208
H	-0.06040	-2.06625	1.51345
C	-1.66409	3.41488	-1.12859
H	-1.24737	4.16444	-1.79046
C	-1.49769	1.20528	-0.14784
C	4.24482	-0.59513	-3.30754
H	3.62605	0.22669	-2.93396
H	3.67750	-1.12204	-4.09911
H	5.14409	-0.16597	-3.76136
C	-3.39239	-2.62186	-0.37988
H	-4.30845	-2.76635	-0.93976
N	-3.56487	4.82353	-0.61318
C	-1.66403	-3.41495	1.12847
H	-1.24709	-4.16470	1.78996
C	-2.71288	1.43451	0.52737
H	-3.10355	0.67645	1.19886
C	4.24612	0.59467	3.30694
H	3.62756	-0.22730	2.93337
H	3.67891	1.12110	4.09891
H	5.14577	0.16577	3.76025
N	-3.56525	-4.82330	0.61356
C	3.45708	2.00448	1.53235
H	3.80828	2.77055	0.82743
H	2.76568	2.51824	2.22974
C	-0.98713	2.22744	-0.97217
H	-0.06069	2.06596	-1.51381
C	-4.83056	-5.03531	-0.06265
H	-5.22271	-6.01356	0.21333
H	-4.71204	-5.00943	-1.15299
H	-5.56808	-4.27715	0.22659
C	-4.83037	5.03544	0.06273
H	-5.22212	6.01401	-0.21267
H	-4.71214	5.00882	1.15307
H	-5.56807	4.27769	-0.22718
C	-3.01910	5.87544	-1.44943
H	-2.03400	6.19801	-1.09160

H	-3.68840	6.73460	-1.42338
H	-2.92275	5.54861	-2.49214
C	-3.01887	-5.87581	1.44863
H	-2.03469	-6.19922	1.08902
H	-3.68900	-6.73436	1.42374
H	-2.92038	-5.54915	2.49116

Table S11. XYZ coordinates of the DFT-optimized structure of **[(*NC*^{NMe2}*N*)BiCl$_2$]$^+$** (solvent = CH_2Cl_2).

Bi	-3.73521	-0.00000	-0.00000
Cl	-3.66835	2.37141	1.26171
Cl	-3.66835	-2.37141	-1.26171
N	-2.94170	1.31312	-2.05055
N	-2.94170	-1.31312	2.05055
N	2.65337	-0.00000	0.00000
C	-1.52850	-0.00000	0.00000
C	-0.84686	0.90057	-0.81708
C	0.54467	-0.89501	0.82735
H	1.10180	-1.56695	1.47462
C	0.54467	0.89501	-0.82734
H	1.10180	1.56695	-1.47461
C	4.55651	1.39154	-0.62856
H	4.94835	0.58573	-1.24112
C	4.73593	3.67893	0.19041
C	1.23397	-0.00000	0.00000
C	-0.84686	-0.90057	0.81708
C	-3.89284	-2.37203	2.41155
H	-4.83495	-1.91731	2.72922
H	-3.50286	-2.98435	3.23651
H	-4.07643	-3.00822	1.54228
C	5.23562	-2.58652	0.56856
H	6.15210	-2.68955	1.13681
C	-1.65281	1.88901	-1.62287
H	-1.87713	2.75477	-0.98886
H	-1.09041	2.25019	-2.49595
C	3.33971	1.21365	0.05929
C	4.73594	-3.67893	-0.19041
C	-3.89283	2.37203	-2.41155
H	-4.83494	1.91731	-2.72923
H	-3.50285	2.98435	-3.23651
H	-4.07643	3.00822	-1.54228
C	2.82759	2.29408	0.80491
H	1.90084	2.17412	1.35693
C	3.50353	-3.49013	-0.87282
H	3.08595	-4.28639	-1.47696
C	3.33971	-1.21365	-0.05928
C	-2.74162	0.40183	-3.18592
H	-2.03008	-0.38009	-2.91292
H	-2.35840	0.95553	-4.05490
H	-3.68951	-0.07126	-3.45096
C	5.23561	2.58653	-0.56858
H	6.15207	2.68957	-1.13684
N	5.40461	-4.85645	-0.25615
C	3.50353	3.49012	0.87284
H	3.08596	4.28639	1.47699
C	4.55653	-1.39153	0.62855
H	4.94837	-0.58572	1.24110
C	-2.74162	-0.40183	3.18592

H	-2.03008	0.38009	2.91292
H	-2.35841	-0.95553	4.05490
H	-3.68951	0.07126	3.45096
N	5.40459	4.85645	0.25615
C	-1.65281	-1.88902	1.62287
H	-1.87713	-2.75477	0.98886
H	-1.09042	-2.25020	2.49595
C	2.82759	-2.29409	-0.80489
H	1.90083	-2.17413	-1.35690
C	6.67865	5.01312	-0.41926
H	7.08053	6.00097	-0.19708
H	6.57036	4.92327	-1.50735
H	7.40314	4.26509	-0.07684
C	6.67867	-5.01310	0.41924
H	7.08055	-6.00095	0.19707
H	6.57039	-4.92324	1.50734
H	7.40316	-4.26507	0.07681
C	4.84870	-5.97505	-0.99406
H	4.75631	-5.74409	-2.06244
H	3.86047	-6.25311	-0.60928
H	5.50874	-6.83516	-0.88739
C	4.84869	5.97504	0.99407
H	3.86044	6.25309	0.60932
H	5.50870	6.83517	0.88738
H	4.75633	5.74409	2.06246

Table S12. XYZ coordinates of the DFT-optimized structure of **[(*NC*^{NMe2}*N*)BiI$_2$]$^+$** (solvent = CH_2Cl_2).

Bi	-3.07916	-0.00000	-0.00000
I	-3.19601	2.88350	1.13080
I	-3.19600	-2.88350	-1.13080
N	-2.27417	1.04609	-2.21454
N	-2.27418	-1.04609	2.21454
N	3.30773	-0.00000	0.00001
C	-0.87368	0.00000	0.00000
C	-0.19137	0.79651	-0.92049
C	1.19968	-0.78948	0.92830
H	1.75578	-1.37937	1.65165
C	1.19969	0.78948	-0.92829
H	1.75579	1.37937	-1.65164
C	5.20618	1.29007	-0.82590
H	5.59141	0.40741	-1.32655
C	5.39361	3.66712	-0.32681
C	1.88976	0.00000	0.00000
C	-0.19137	-0.79651	0.92050
C	-3.21942	-2.04168	2.73895
H	-4.15803	-1.54485	2.99758
H	-2.81527	-2.52361	3.63970
H	-3.41432	-2.80161	1.97877
C	5.88607	-2.48105	0.93538
H	6.79757	-2.50417	1.52021
C	-0.99308	1.67817	-1.84475
H	-1.22662	2.61466	-1.32347
H	-0.42354	1.93487	-2.74959
C	3.99580	1.20966	-0.10924
C	5.39361	-3.66712	0.32681
C	-3.21941	2.04168	-2.73895
H	-4.15802	1.54484	-2.99758

H	-2.81526	2.52361	-3.63970
H	-3.41432	2.80161	-1.97877
C	3.49228	2.38099	0.49048
H	2.57146	2.33830	1.06349
C	4.16939	-3.57389	-0.38975
H	3.75838	-4.44505	-0.88528
C	3.99580	-1.20965	0.10925
C	-2.04042	0.00663	-3.22857
H	-1.33498	-0.73476	-2.84786
H	-1.63070	0.45973	-4.14252
H	-2.97956	-0.49611	-3.46700
C	5.88606	2.48106	-0.93539
H	6.79755	2.50417	-1.52023
N	6.06088	-4.84237	0.43022
C	4.16940	3.57389	0.38977
H	3.75839	4.44504	0.88530
C	5.20619	-1.29007	0.82590
H	5.59142	-0.40740	1.32654
C	-2.04043	-0.00663	3.22857
H	-1.33499	0.73477	2.84786
H	-1.63071	-0.45973	4.14253
H	-2.97957	0.49611	3.46700
N	6.06087	4.84238	-0.43022
C	-0.99308	-1.67817	1.84475
H	-1.22662	-2.61466	1.32348
H	-0.42354	-1.93487	2.74960
C	3.49228	-2.38099	-0.49047
H	2.57144	-2.33830	-1.06347
C	7.31980	4.90855	-1.14796
H	7.71177	5.92316	-1.08769
H	7.19035	4.65350	-2.20700
H	8.06142	4.22892	-0.71204
C	7.31982	-4.90854	1.14794
H	7.71179	-5.92315	1.08767
H	7.19038	-4.65348	2.20698
H	8.06143	-4.22891	0.71200
C	5.52033	-6.04685	-0.17147
H	5.44123	-5.94811	-1.26093
H	4.52840	-6.28245	0.23182
H	6.18343	-6.88297	0.04710
C	5.52033	6.04685	0.17147
H	4.52840	6.28245	-0.23180
H	6.18342	6.88297	-0.04709
H	5.44124	5.94811	1.26094

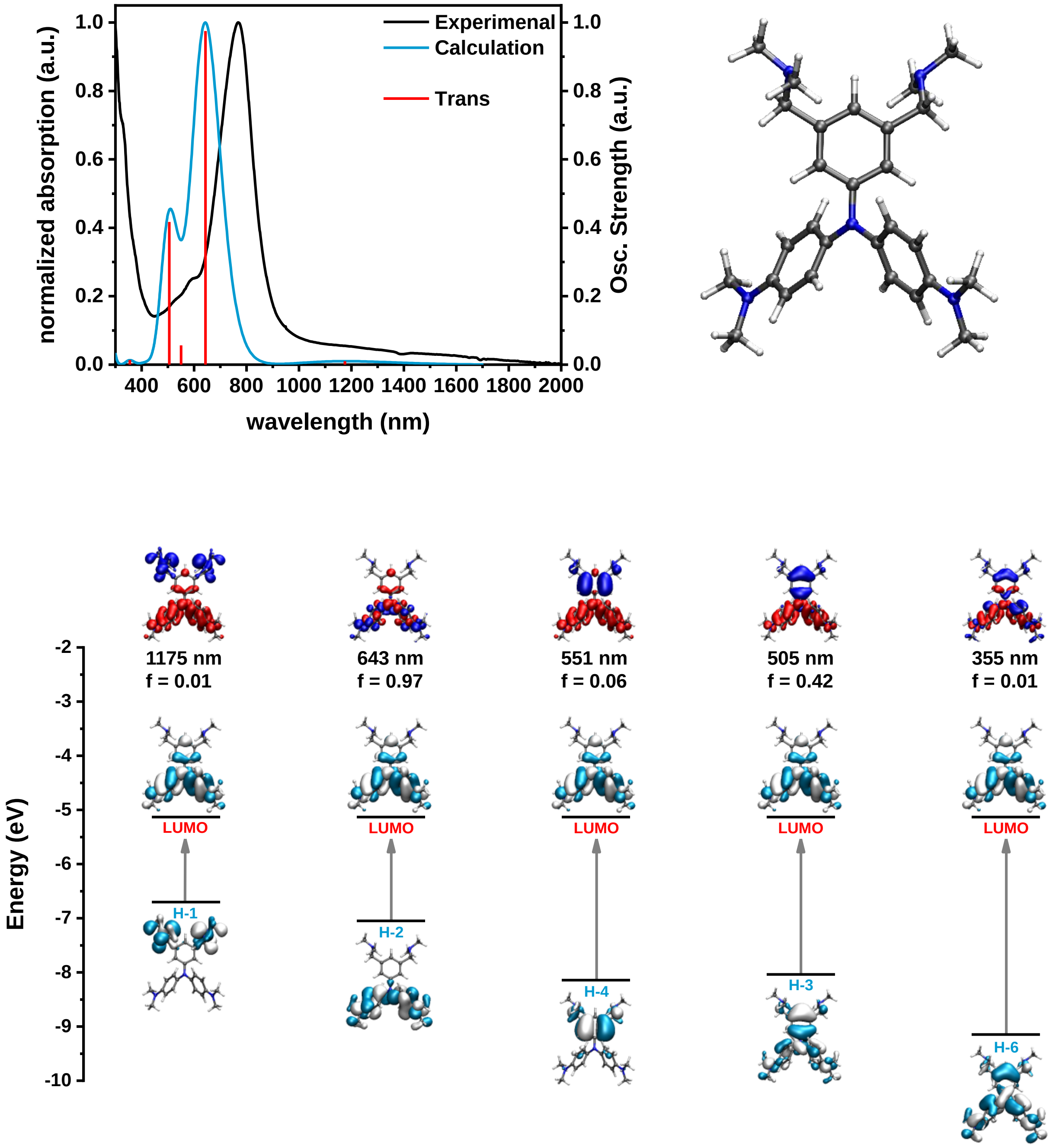

Figure S45. Left: Comparison of the experimental (black line) and the TD-DFT-calculated (blue line) absorption spectra of **[*NCH*^{NMe2}*N*]$^{2+}$** in CH_2Cl_2 in the electronic singlet ground state. Transitions are indicated by red bars. Right: Geometry-optimized structure of **[*NCH*^{NMe2}*N*]$^{2+}$**. Bottom: TD-DFT-calculated transitions of **[*NCH*^{NMe2}*N*]$^{2+}$** along with the mainly contributing molecular orbitals and the electron density difference maps (EDDMs). Blue color indicates a loss and red color a gain of electron density during the corresponding excitation.

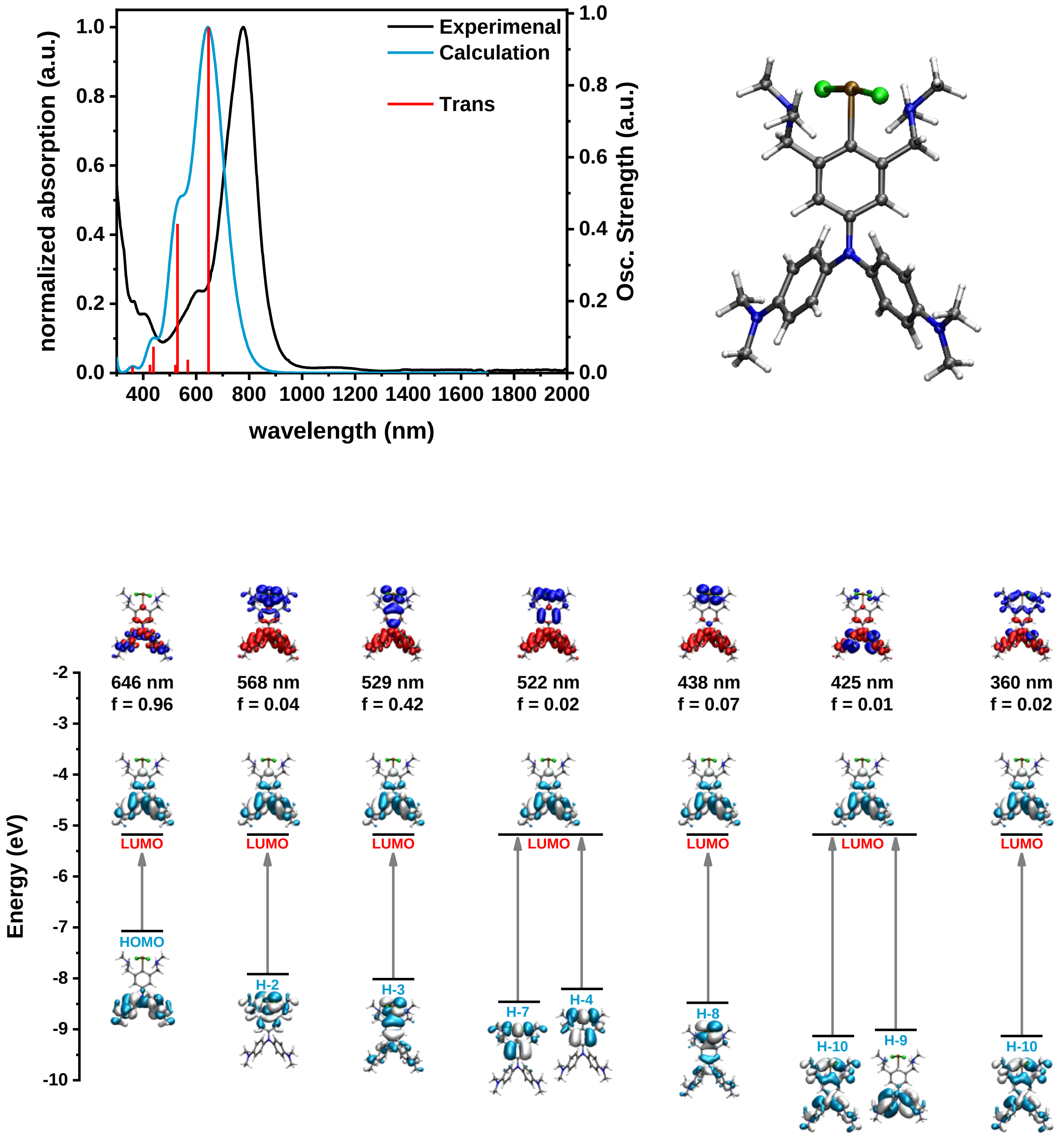

Figure S46. Left: Comparison of the experimental (black line) and the TD-DFT-calculated (blue line) absorption spectra of **[(*NC*^{NMe2}*N*)BiCl$_2$]$^{2+}$** in the singlet ground state and in CH_2Cl_2. Transitions are indicated by red bars. Right: Geometry-optimized structure of **[(*NC*^{NMe2}*N*)BiCl$_2$]$^{2+}$**. Bottom: TD-DFT-calculated transitions of **[(*NC*^{NMe2}*N*)BiCl$_2$]$^{2+}$** along with the mainly contributing molecular orbitals and the electron density difference maps (EDDMs). Blue color indicates a loss and red color a gain of electron density during the corresponding excitation.

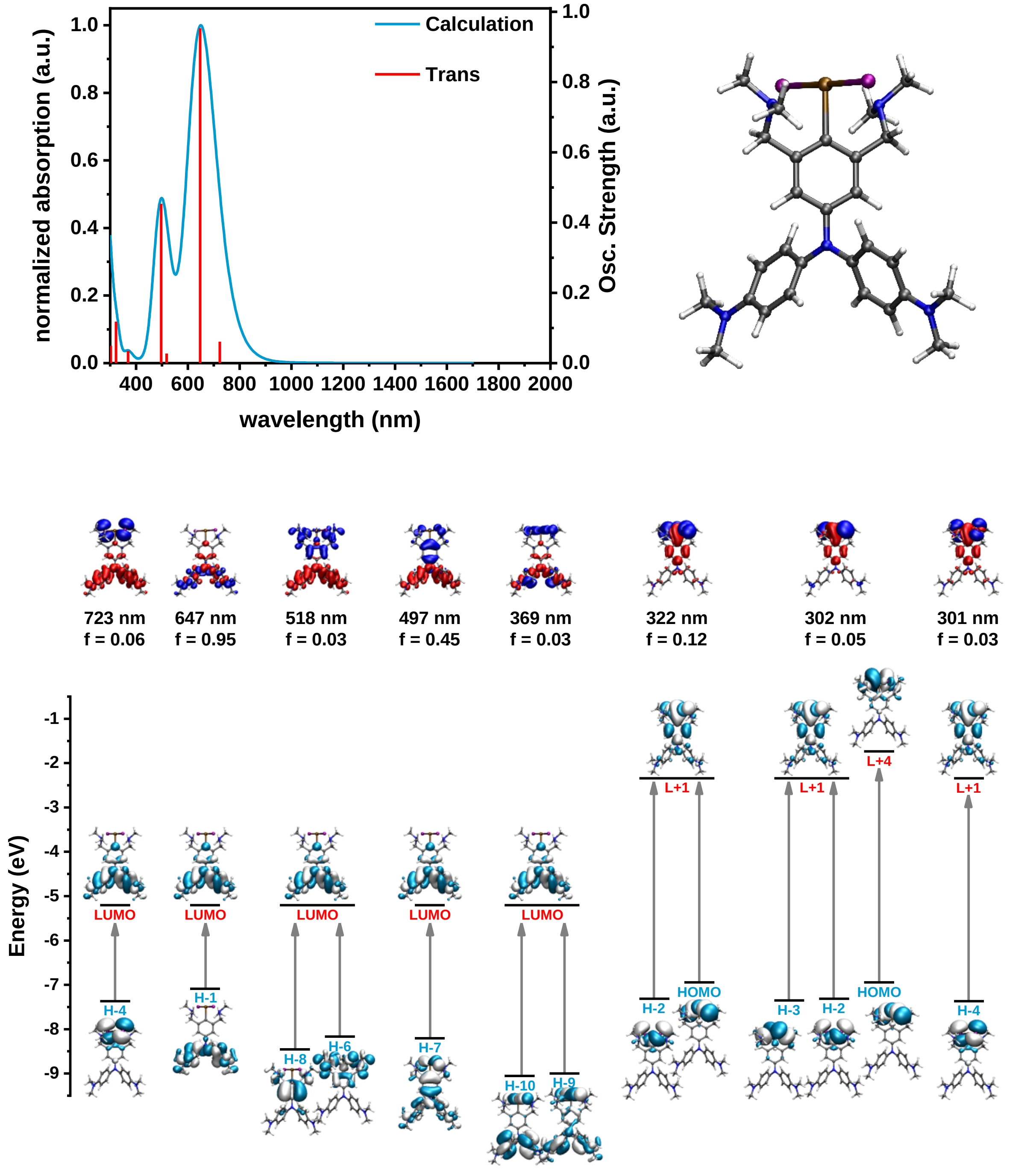

Figure S47. Left: TD-DFT-calculated absorption spectra of **[(*NC*^{NMe2}*N*)BiI$_2$]$^{2+}$** (blue line) in the singlet ground state and in CH_2Cl_2. Transitions are indicated by red bars. Right: Geometry-optimized structure of **[(*NC*^{NMe2}*N*)BiI$_2$]$^{2+}$**. Bottom: TD-DFT-calculated transitions of **[(*NC*^{NMe2}*N*)BiI$_2$]$^{2+}$** along with the mainly contributing molecular orbitals and the electron density difference maps (EDDMs). Blue color indicates a loss and red color a gain of electron density during the corresponding excitation.

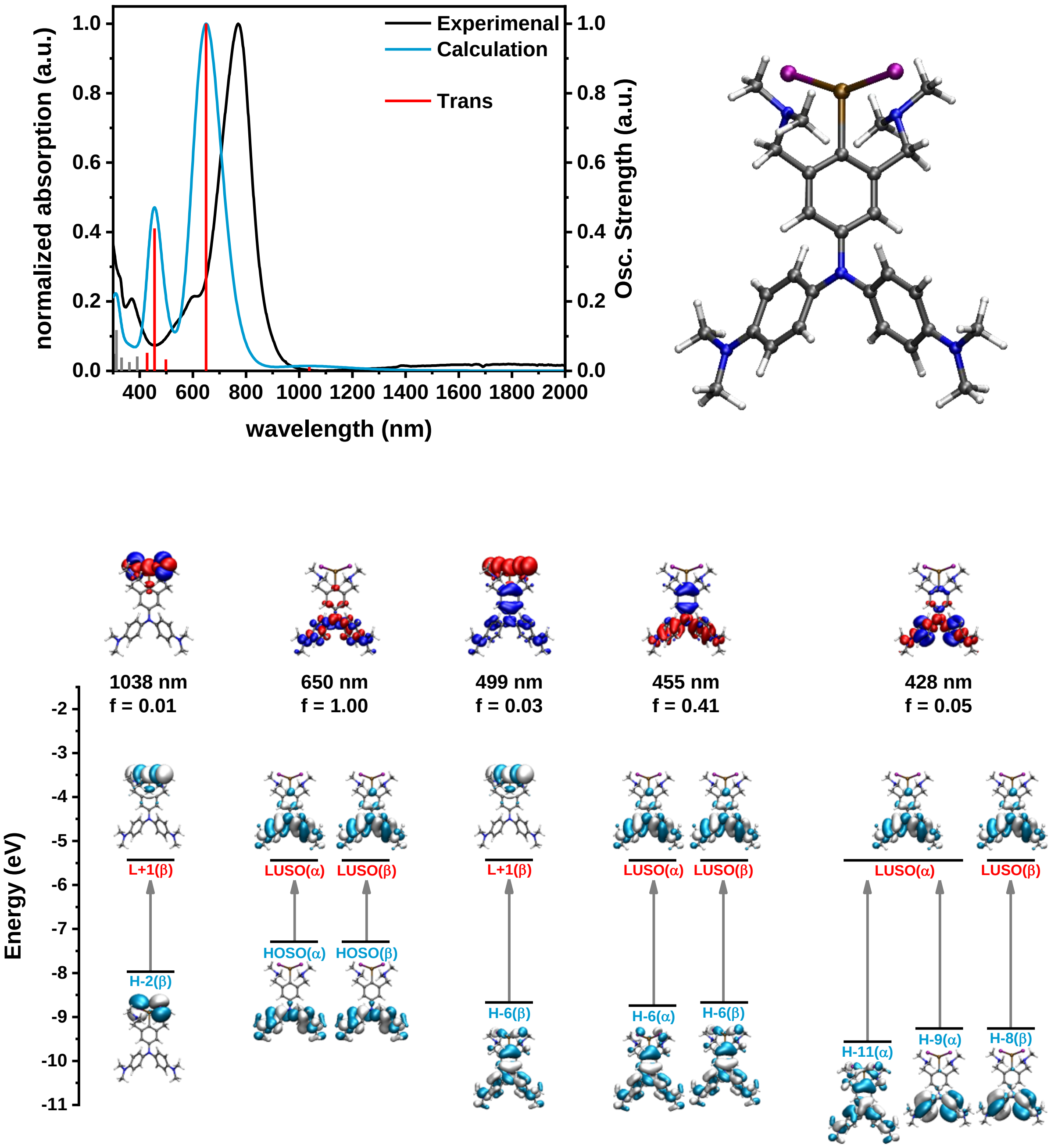

Figure S48. Left: Comparison of the experimental (black line) and the TD-DFT-calculated (blue line) absorption spectra of **[(*NC*^{NMe2}*N*)BiI$_2$]$^{3+}$** in CH_2Cl_2. Transitions are indicated by red bars. Right: Geometry-optimized structure of **[(*NC*^{NMe2}*N*)BiI$_2$]$^{3+}$**. Bottom: TD-DFT-calculated transitions of **[(*NC*^{NMe2}*N*)BiI$_2$]$^{3+}$** along with the mainly contributing molecular orbitals and the electron density difference maps (EDDMs). Blue color indicates a loss and red color a gain of electron density during the corresponding excitation.

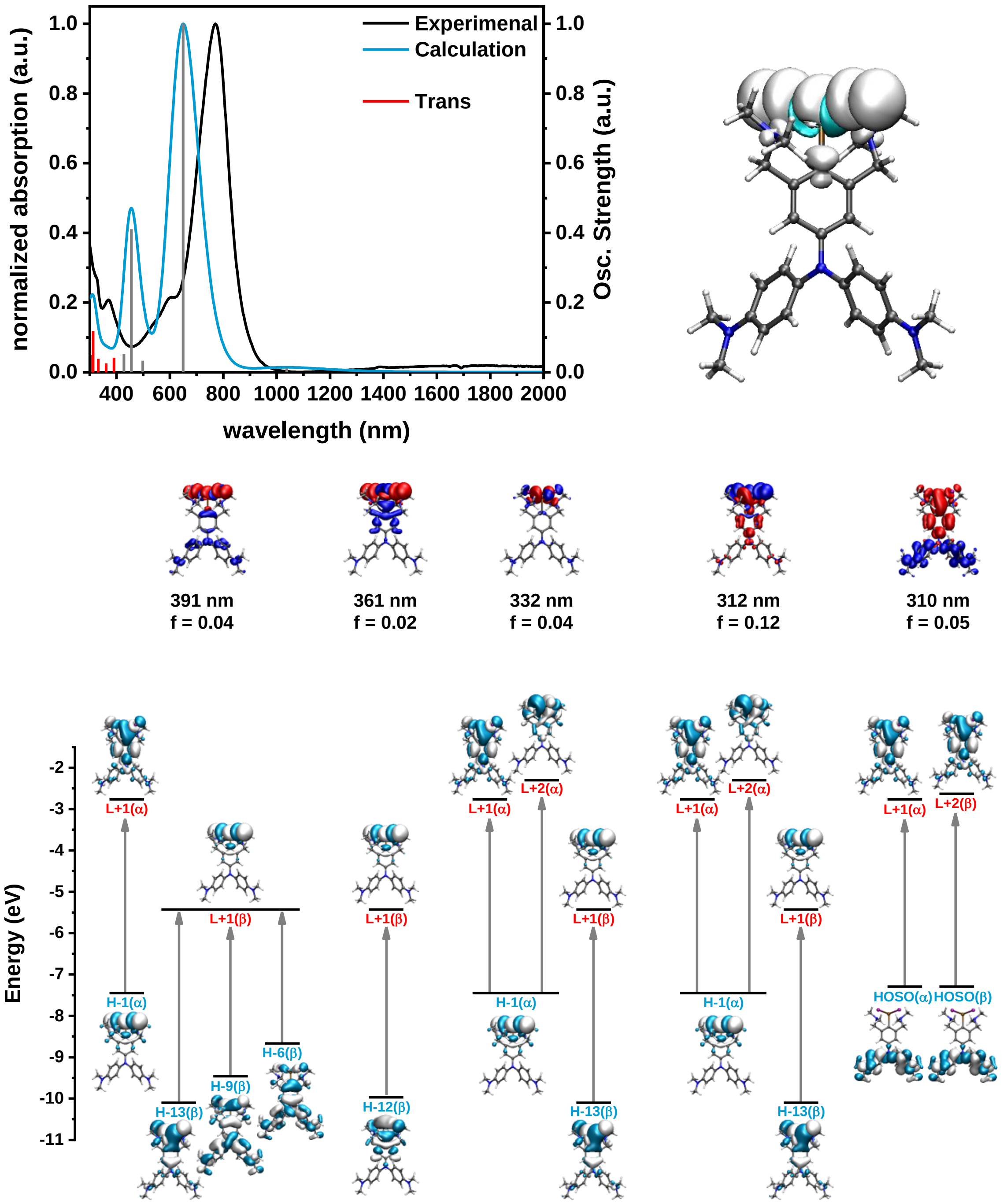

Figure S49. Left: Comparison of the experimental (black line) and the TD-DFT-calculated (blue line) absorption spectra of **[(*NC*^{NMe2}*N*)BiI$_2$]$^{3+}$** in CH_2Cl_2. Transitions are indicated by red bars. Right: Spin-density of **[(*NC*^{NMe2}*N*)BiI$_2$]$^{3+}$**. Bottom: TD-DFT-calculated transitions of **[(*NC*^{NMe2}*N*)BiI$_2$]$^{3+}$** along with the mainly contributing molecular orbitals and the electron density difference maps (EDDMs). Blue color indicates a loss and red color a gain of electron density during the corresponding excitation.

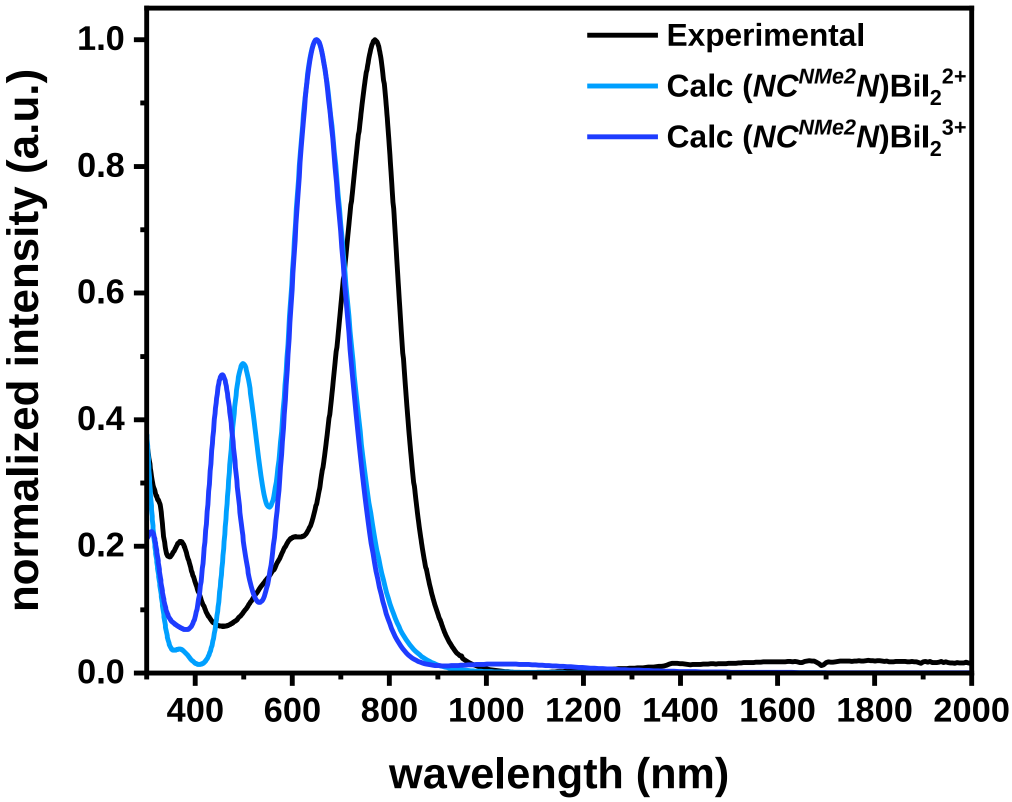

Figure S50. Comparison of the experimental (black line) and the TD-DFT-calculated absorption spectra of **[(*NC*^{NMe2}*N*)BiI$_2$]$^{2+}$** (light blue line) and **[(*NC*^{NMe2}*N*)BiI$_2$]$^{3+}$** (dark blue line) in CH_2Cl_2.

Table S13. XYZ coordinates of the DFT-optimized structure of **[*NCH*^{NMe2}*N*]$^{2+}$** (solvent = CH_2Cl_2).

H	4.43163	-0.00253	-0.00189
N	4.59656	-1.39055	-2.24567
N	4.60355	1.38783	2.23791
N	-0.82894	-0.00055	0.00140
C	3.34569	-0.00235	-0.00095
C	2.66672	-0.94477	-0.78041
C	1.27793	0.93705	0.78199
H	0.72310	1.62809	1.41078
C	1.27613	-0.94086	-0.78046
H	0.71997	-1.63177	-1.40820
C	-2.74962	-1.39921	-0.50150
H	-3.13907	-0.63951	-1.17173
C	-2.88220	-3.63583	0.44868
C	0.59461	-0.00153	0.00110
C	2.66855	0.94034	0.77968
C	5.50643	2.42400	2.70270
H	6.39650	1.96106	3.14077
H	5.05314	3.08200	3.46784
H	5.82431	3.04690	1.86011
C	-3.41833	2.57684	0.35729
H	-4.32996	2.73168	0.92040
C	3.43844	-1.96234	-1.58725
H	3.79538	-2.74028	-0.89872
H	2.75310	-2.46548	-2.29743
C	-1.49856	-1.19118	0.14106
C	-2.87936	3.63603	-0.44481
C	5.49890	-2.42561	-2.71395
H	6.38642	-1.96157	-3.15600
H	5.04328	-3.08456	-3.47689
H	5.82127	-3.04774	-1.87250
C	-0.94350	-2.24042	0.92346
H	-0.00913	-2.07813	1.44949
C	-1.60818	3.41832	-1.07276
H	-1.19021	4.17161	-1.72861
C	-1.49707	1.18964	-0.13813
C	4.21360	-0.50650	-3.33096
H	3.57294	0.29712	-2.95399
H	3.66869	-1.03294	-4.13774
H	5.10907	-0.05073	-3.76509
C	-3.41562	-2.58090	-0.36146
H	-4.32821	-2.73512	-0.92282
N	-3.53149	4.79073	-0.59028
C	-1.61560	-3.41583	1.08389
H	-1.19691	-4.16987	1.73824
C	-2.75227	1.39574	0.49800
H	-3.14321	0.63522	1.16645
C	4.22637	0.50291	3.32453
H	3.58507	-0.30137	2.95005
H	3.68427	1.02845	4.13378
H	5.12426	0.04811	3.75465
N	-3.53624	-4.79010	0.59475
C	3.44185	1.95821	1.58468
H	3.79468	2.73747	0.89552
H	2.75851	2.45948	2.29808
C	-0.93799	2.24202	-0.91429
H	-0.00312	2.07998	-1.43948
C	-4.87989	-4.96696	0.05754
H	-5.31623	-5.86395	0.49410
H	-4.85268	-5.08403	-1.03198
H	-5.51375	-4.11577	0.31663
C	-4.89790	4.96144	-0.11348
H	-5.34985	5.79540	-0.65013
H	-4.91277	5.18378	0.95984
H	-5.49110	4.06735	-0.31146
C	-2.91100	5.94465	-1.22896
H	-1.86868	6.04283	-0.92144

H	-3.44408	6.84205	-0.91566
H	-2.96742	5.86422	-2.32053
C	-2.92974	-5.91815	1.29159
H	-1.91073	-6.08841	0.93656
H	-3.51694	-6.81196	1.08764
H	-2.91599	-5.74697	2.37374

Table S14. XYZ coordinates of the DFT-optimized structure of **[(*NC*^{NMe2}*N*)BiCl$_2$]$^{2+}$** in the singlet ground state (solvent = CH_2Cl_2).

Bi	3.73072	-0.00001	-0.00001
Cl	3.58932	2.40328	-1.17074
Cl	3.58929	-2.40330	1.17072
N	2.93408	1.23253	2.09657
N	2.93404	-1.23253	-2.09658
N	-2.66003	0.00002	0.00004
C	1.51193	0.00001	-0.00000
C	0.83422	0.86788	0.85722
C	-0.55629	-0.86524	-0.86721
H	-1.11648	-1.49901	-1.54927
C	-0.55627	0.86526	0.86727
H	-1.11644	1.49903	1.54934
C	-4.58539	1.36021	0.58847
H	-4.98385	0.55314	1.19462
C	-4.70433	3.66359	-0.18906
C	-1.23384	0.00001	0.00003
C	0.83420	-0.86786	-0.85721
C	3.88871	-2.28118	-2.48242
H	4.83485	-1.81682	-2.77188
H	3.50827	-2.86233	-3.33344
H	4.06030	-2.94802	-1.63406
C	-5.25006	-2.54877	-0.52896
H	-6.16796	-2.66204	-1.09160
C	1.64186	1.82136	1.70213
H	1.85647	2.71298	1.10166
H	1.08173	2.14281	2.59150
C	-3.32525	1.19954	-0.05141
C	-4.70437	-3.66355	0.18906
C	3.88876	2.28117	2.48241
H	4.83488	1.81679	2.77189
H	3.50832	2.86235	3.33342
H	4.06037	2.94799	1.63404
C	-2.75631	2.30851	-0.73709
H	-1.81361	2.18896	-1.25981
C	-3.42436	-3.49400	0.81398
H	-2.99545	-4.29582	1.40165
C	-3.32525	-1.19952	0.05144
C	2.74661	0.28638	3.20620
H	2.03427	-0.48939	2.91820
H	2.37245	0.81383	4.09506
H	3.69833	-0.19182	3.44633
C	-5.25012	2.54877	0.52882
H	-6.16808	2.66204	1.09136
N	-5.35912	-4.82337	0.26055
C	-3.42426	3.49405	-0.81388
H	-2.99531	4.29588	-1.40150
C	-4.58533	-1.36021	-0.58857
H	-4.98374	-0.55315	-1.19477
C	2.74661	-0.28637	-3.20621
H	2.03427	0.48941	-2.91821

H	2.37246	-0.81381	-4.09508
H	3.69834	0.19182	-3.44632
N	-5.35910	4.82338	-0.26051
C	1.64181	-1.82133	-1.70216
H	1.85640	-2.71298	-1.10171
H	1.08166	-2.14273	-2.59153
C	-2.75637	-2.30847	0.73719
H	-1.81370	-2.18891	1.25996
C	-6.72792	4.95528	0.22259
H	-7.17642	5.83337	-0.24119
H	-6.74657	5.08315	1.31121
H	-7.32122	4.08228	-0.05497
C	-6.72777	-4.95556	-0.22295
H	-7.17638	-5.83346	0.24109
H	-6.74606	-5.08391	-1.31151
H	-7.32121	-4.08246	0.05402
C	-4.73998	-6.01643	0.82480
H	-4.77481	-5.99297	1.91999
H	-3.70440	-6.10795	0.49339
H	-5.28768	-6.89098	0.47516
C	-4.74000	6.01663	-0.82437
H	-3.70441	6.10802	-0.49297
H	-5.28766	6.89106	-0.47438
H	-4.77487	5.99357	-1.91956

Table S15. XYZ coordinates of the DFT-optimized structure of **[(*NC^{NMe2}N*)BiI$_2$]$^{2+}$** (singlet ground state; solvent = CH_2Cl_2).

Bi	3.09041	-0.00000	-0.00001
I	3.12230	2.91604	-1.01686
I	3.12235	-2.91604	1.01685
N	2.28434	0.95392	2.25357
N	2.28432	-0.95393	-2.25357
N	-3.29796	-0.00001	0.00001
C	0.87311	-0.00001	0.00001
C	0.19554	0.75510	0.96001
C	-1.19499	-0.75076	-0.96773
H	-1.75548	-1.29529	-1.72246
C	-1.19499	0.75073	0.96776
H	-1.75547	1.29527	1.72249
C	-5.22014	1.26971	0.76750
H	-5.60992	0.39089	1.27105
C	-5.34845	3.65234	0.28752
C	-1.87192	-0.00001	0.00001
C	0.19553	-0.75512	-0.95999
C	3.23771	-1.93283	-2.79816
H	4.17965	-1.42692	-3.02546
H	2.84681	-2.38272	-3.72054
H	3.42200	-2.71793	-2.06153
C	-5.88699	-2.45462	-0.86398
H	-6.79822	-2.49470	-1.44747
C	0.99924	1.59559	1.92060
H	1.22197	2.55455	1.43685
H	0.43232	1.80950	2.83771
C	-3.96475	1.19502	0.10305
C	-5.34851	-3.65233	-0.28751
C	3.23772	1.93283	2.79816
H	4.17966	1.42693	3.02545
H	2.84682	2.38271	3.72054
H	3.42200	2.71793	2.06152
C	-3.40241	2.38239	-0.44218
H	-2.46430	2.33177	-0.98430

C	-4.07352	-3.56593	0.36465
H	-3.65267	-4.43631	0.85178
C	-3.96477	-1.19502	-0.10302
C	2.06307	-0.11595	3.23919
H	1.34941	-0.84383	2.84810
H	1.66986	0.31131	4.17235
H	3.00406	-0.62822	3.44734
C	-5.88694	2.45465	0.86403
H	-6.79816	2.49475	1.44753
N	-6.00327	-4.81121	-0.36810
C	-4.07348	3.56592	-0.36465
H	-3.65264	4.43628	-0.85183
C	-5.22017	-1.26969	-0.76745
H	-5.60994	-0.39085	-1.27099
C	2.06303	0.11593	-3.23919
H	1.34938	0.84381	-2.84810
H	1.66982	-0.31133	-4.17235
H	3.00402	0.62820	-3.44736
N	-6.00319	4.81124	0.36811
C	0.99923	-1.59561	-1.92058
H	1.22197	-2.55457	-1.43683
H	0.43230	-1.80953	-2.83769
C	-3.40243	-2.38241	0.44218
H	-2.46431	-2.33181	0.98428
C	-7.37049	4.88174	0.86759
H	-7.82564	5.80183	0.50191
H	-7.38491	4.89252	1.96359
H	-7.95912	4.03964	0.50076
C	-7.37056	-4.88167	-0.86761
H	-7.82571	-5.80181	-0.50202
H	-7.38496	-4.89236	-1.96361
H	-7.95920	-4.03961	-0.50071
C	-5.38775	-6.06879	0.03824
H	-5.44280	-6.19589	1.12546
H	-4.34632	-6.11222	-0.28426
H	-5.92657	-6.88741	-0.43825
C	-5.38763	6.06879	-0.03828
H	-4.34614	6.11211	0.28403
H	-5.92629	6.88743	0.43837
H	-5.44289	6.19595	-1.12548

Table S16. XYZ coordinates of the DFT-optimized structure of **[(*NC*^{NMe2}*N*)BiI$_2$]$^{3+}$** (solvent = CH_2Cl_2).

Bi	-2.91622	0.00011	0.00012
I	-3.58457	2.75747	0.63353
I	-3.58479	-2.75721	-0.63331
N	-2.12635	0.66696	-2.34408
N	-2.12578	-0.66683	2.34410
N	3.44821	-0.00000	-0.00059
C	-0.71081	0.00005	-0.00018
C	-0.04716	0.66996	-1.02481
C	1.34524	-0.67573	1.02286
H	1.91010	-1.16745	1.81023
C	1.34502	0.67577	-1.02373
H	1.90970	1.16752	-1.81122
C	5.37607	1.30501	-0.69762
H	5.78840	0.44506	-1.21534
C	5.47593	3.67313	-0.14277
C	2.01598	0.00001	-0.00052
C	-0.04694	-0.66988	1.02427
C	-3.08222	-1.52863	3.05764
H	-3.99905	-0.96565	3.24882

H	-2.66074	-1.85997	4.01530
H	-3.32036	-2.40355	2.44899
C	6.03459	-2.49683	0.74348
H	6.95907	-2.55997	1.30349
C	-0.86868	1.38940	-2.06697
H	-1.12927	2.38736	-1.69289
H	-0.30136	1.52569	-2.99721
C	4.10560	1.20137	-0.06450
C	5.47504	-3.67356	0.14268
C	-3.08300	1.52873	-3.05737
H	-3.99993	0.96576	-3.24815
H	-2.66186	1.85995	-4.01522
H	-3.32089	2.40372	-2.44874
C	3.51632	2.37205	0.49014
H	2.56132	2.30239	0.99951
C	4.17888	-3.56204	-0.46449
H	3.73435	-4.41735	-0.95748
C	4.10543	-1.20146	0.06344
C	-1.84524	-0.53296	-3.14988
H	-1.14481	-1.18504	-2.62294
H	-1.40427	-0.24247	-4.11263
H	-2.77178	-1.07908	-3.33367
C	6.03538	2.49634	-0.74357
H	6.96015	2.55926	-1.30310
N	6.12725	-4.83505	0.16402
C	4.17931	3.56193	0.46349
H	3.73454	4.41735	0.95603
C	5.37560	-1.30535	0.69713
H	5.78787	-0.44548	1.21505
C	-1.84445	0.53310	3.14980
H	-1.14398	1.18505	2.62276
H	-1.40346	0.24264	4.11255
H	-2.77091	1.07937	3.33359
N	6.12860	4.83442	-0.16331
C	-0.86819	-1.38929	2.06666
H	-1.12891	-2.38724	1.69266
H	-0.30063	-1.52559	2.99675
C	3.51620	-2.37200	-0.49157
H	2.56157	-2.30206	-1.00161
C	7.50599	4.92864	-0.63161
H	7.95131	5.83299	-0.21815
H	7.53980	4.98678	-1.72557
H	8.08972	4.07277	-0.29053
C	7.50467	-4.92945	0.63219
H	7.94963	-5.83417	0.21913
H	7.53860	-4.98703	1.72617
H	8.08856	-4.07391	0.29059
C	5.49923	-6.07396	-0.28033
H	5.51043	-6.14238	-1.37416
H	4.47215	-6.13868	0.08202
H	6.05953	-6.91336	0.13008
C	5.50084	6.07318	0.28195
H	4.47444	6.13932	-0.08212
H	6.06255	6.91279	-0.12606
H	5.51015	6.13977	1.37592

Photoluminescence Data

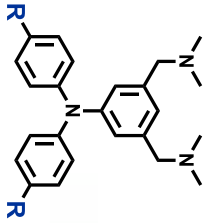

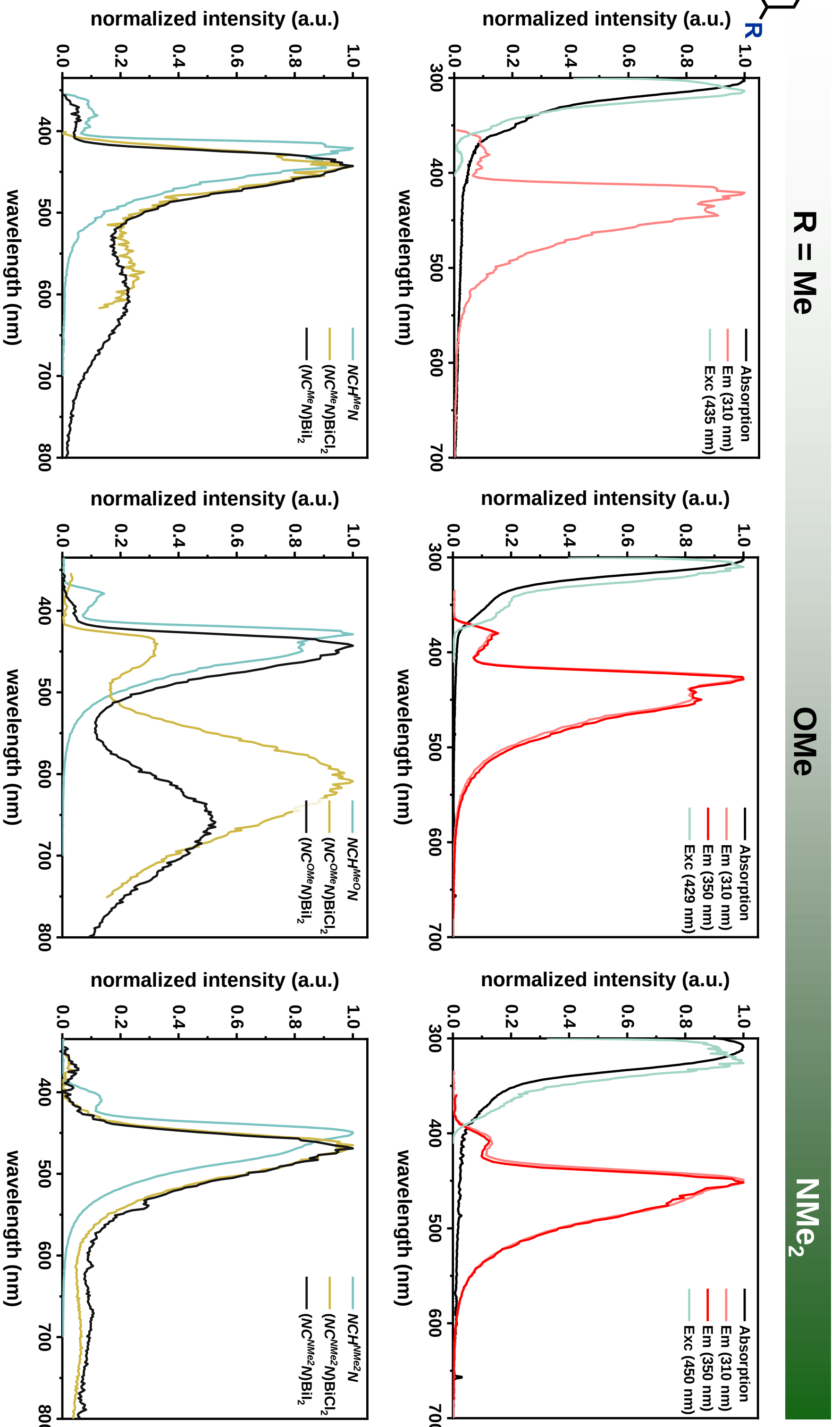

Figure S51. Top panel: PL data of 10 μM solutions of ***NCH^RN*** (R = Me, OMe, NMe_2) at 77 K in MeTHF. Absorption spectra are shown as black, emission spectra as red, and excitation spectra as cyan lines. Bottom panel: Comparison of the emission spectra of complexes **(*NC^RN*)BiX_2** (R = Me, OMe, NMe_2; X = Cl, I) with those of the proligands ***NCH^RN*** (R = Me, OMe, NMe_2).

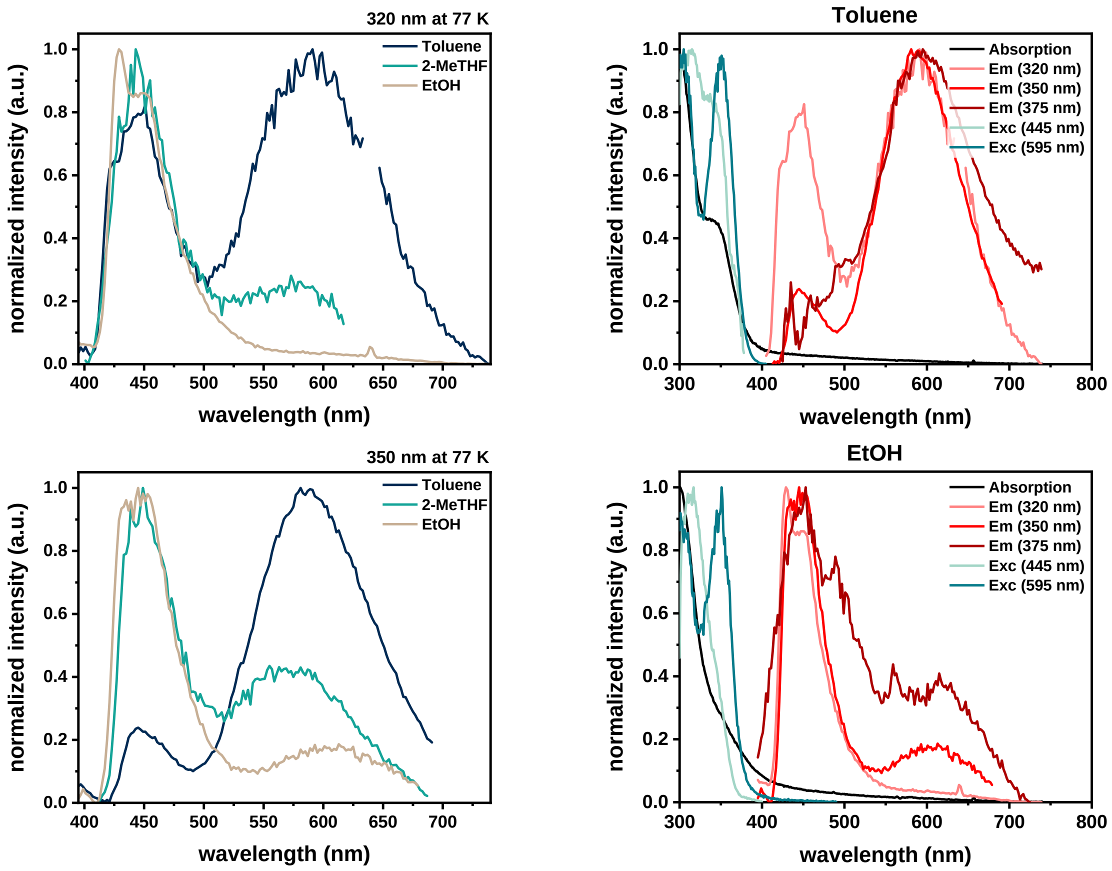

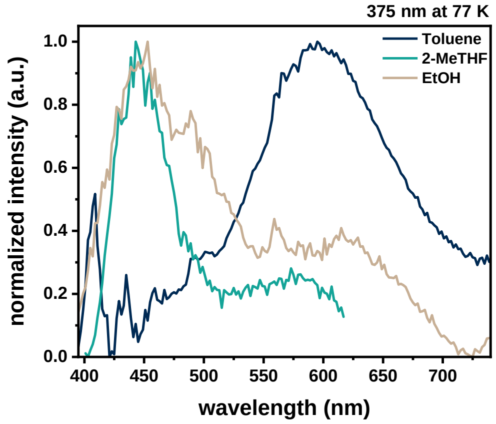

Excitation @350 nm	**($NC^{Me}N$)BiCl$_2$**		
	ϕ_F (445 nm)	ϕ_P (595 nm)	ϕ_P/ϕ_F
Toluene	4%	47%	12
2-MeTHF	28%	47%	1.7
EtOH	18%	16%	0.89

Figure S52. Left: Emission spectra of **(*NC*^{Me}*N*)BiCl$_2$** in toluene, 2-MeTHF and EtOH at 77 K, excited at 320 nm (top), 350 nm (middle) and 375 nm (bottom); right: PL data of 10 µM solutions of**(*NC*^{Me}*N*)BiCl$_2$** at 77 K in toluene (top) and EtOH (middle). Absorption spectra are shown as black, emission spectra as red, and excitation spectra as cyan lines; bottom right: Table with quantum yields of **(*NC*^{Me}*N*)BiCl$_2$** emissions in different solvents, excited at 350 nm at 77 K.

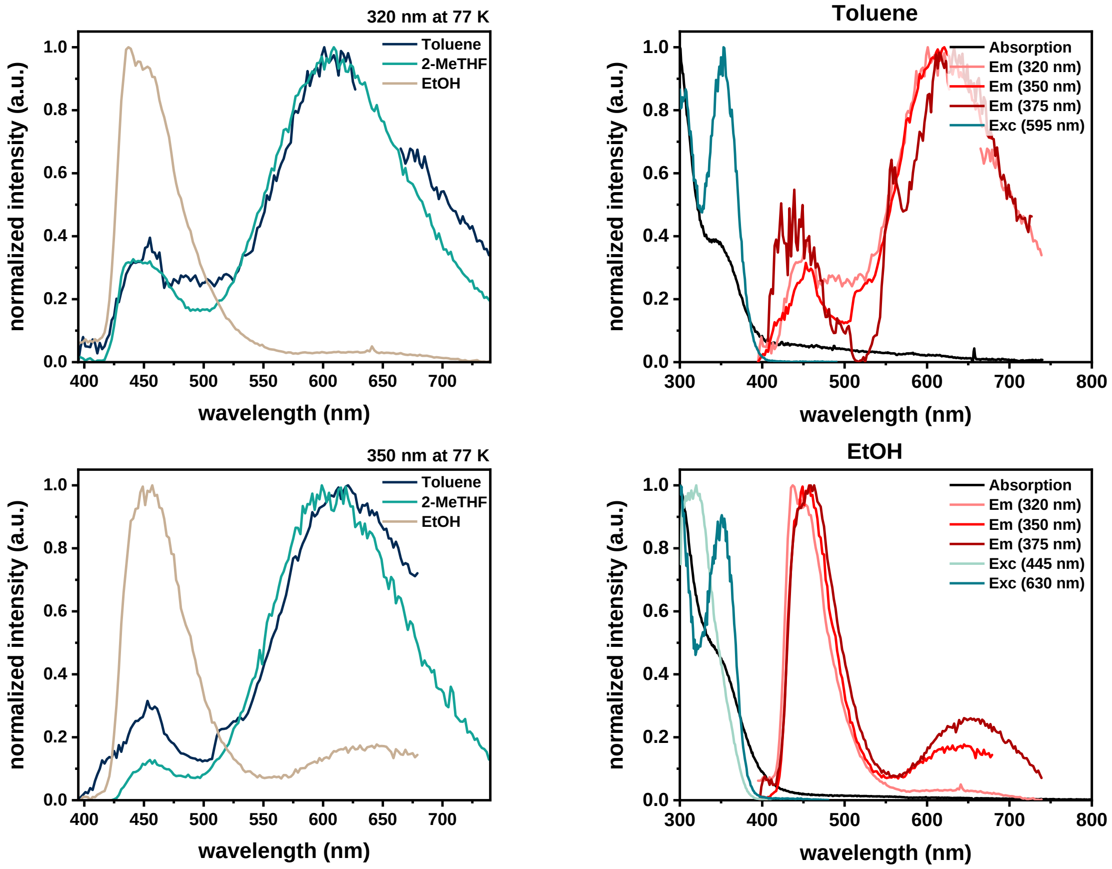

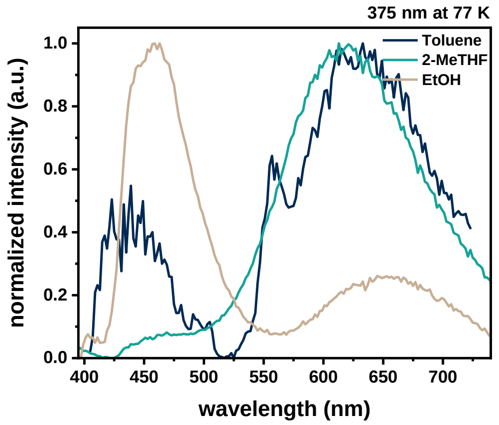

Excitation @350 nm	$(NC^{OMe}N)BiCl_2$		
	ϕ_F (445 nm)	ϕ_P (620 nm)	ϕ_P/ϕ_F
Toluene	6%	94%	16
2-MeTHF	3%	47%	16
EtOH	14%	7%	0.50

Figure S53. Left: Emission spectra of $\mathbf{(\mathit{NC}^{\mathit{OMe}}\mathit{N})BiCl_2}$ in toluene, 2-MeTHF and EtOH at 77 K, excited at 320 nm (top), 350 nm (middle) and 375 nm (bottom); right: PL data of 10 µM solutions of$\mathbf{(\mathit{NC}^{\mathit{OMe}}\mathit{N})BiCl_2}$ at 77 K in toluene (top) and EtOH (middle). Absorption spectra are shown as black, emission spectra as red, and excitation spectra as cyan lines; bottom right: Table with quantum yields of $\mathbf{(\mathit{NC}^{\mathit{OMe}}\mathit{N})BiCl_2}$ emissions in different solvents, excited at 350 nm at 77 K.

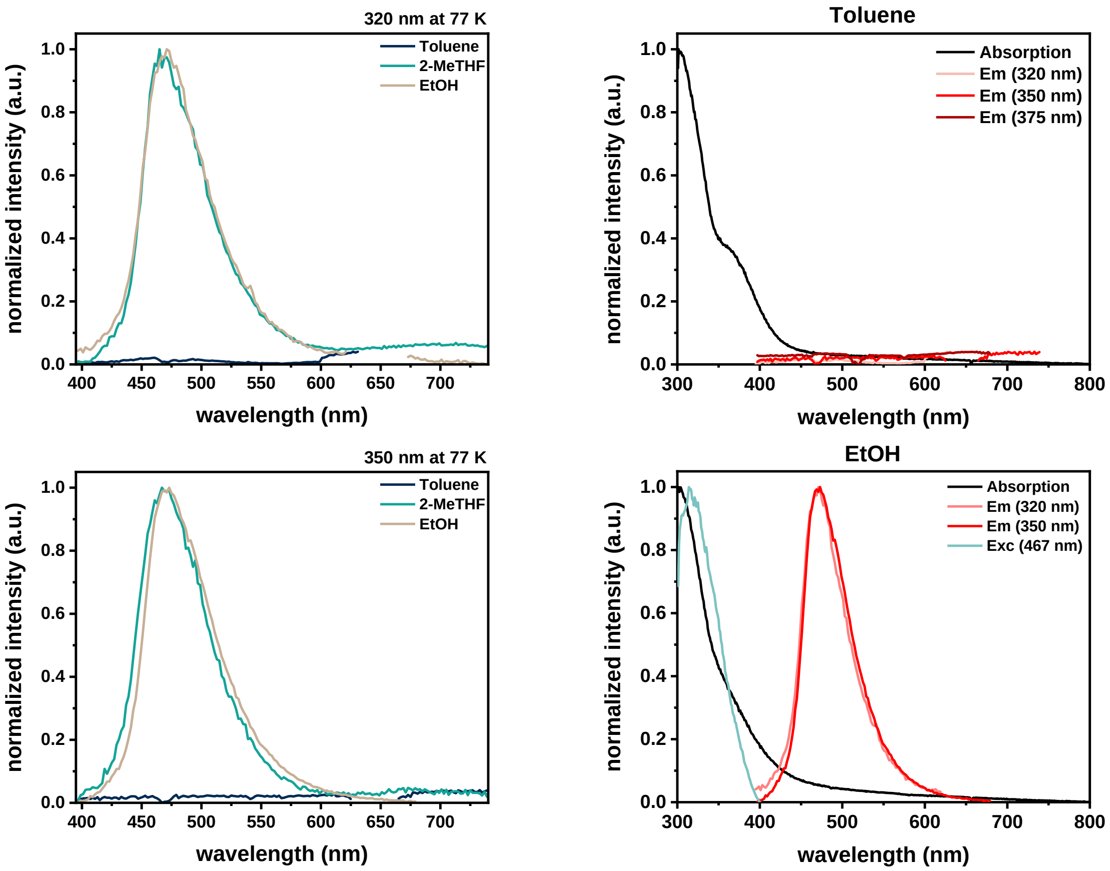

Excitation @350 nm	**$(NC^{NMe2}N)BiCl_2$**		
	ϕ_F (467 nm)	ϕ_P	ϕ_P/ϕ_F
Toluene	0%	-	-
2-MeTHF	1.3%	-	-
EtOH	10%	-	-

Figure S 54. Left: Emission spectra of **$(NC^{NMe2}N)BiCl_2$** in toluene, 2-MeTHF and EtOH at 77 K, excited at 320 nm (top) and 350 nm (middle); right: PL data of 10 µM solutions of**$(NC^{NMe2}N)BiCl_2$** at 77 K in toluene (top) and EtOH (middle). Absorption spectra are shown as black, emission spectra as red, and excitation spectra as cyan lines; bottom: Table with quantum yields of **$(NC^{NM2e}N)BiCl_2$** emissions in different solvents, excited at 350 nm at 77 K.

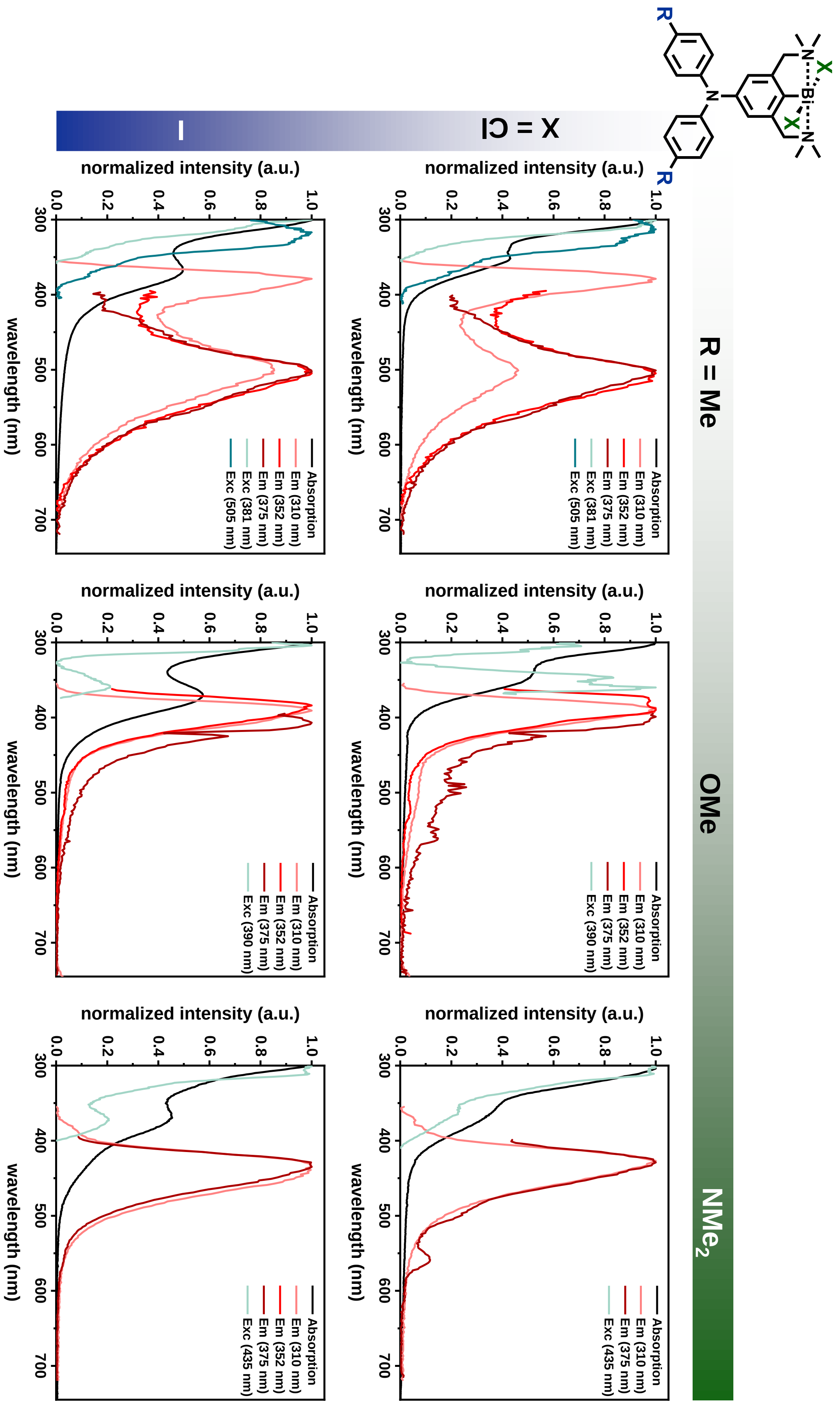

Figure S55. PL data of 10 μM solutions of complexes **(*NC^R N*)BiX$_2$** (R = Me, OMe, NMe$_2$; X = Cl, I) at r.t. in degassed THF. Absorption spectra are depicted as black, emission spectra as red, and excitation spectra as cyan lines.

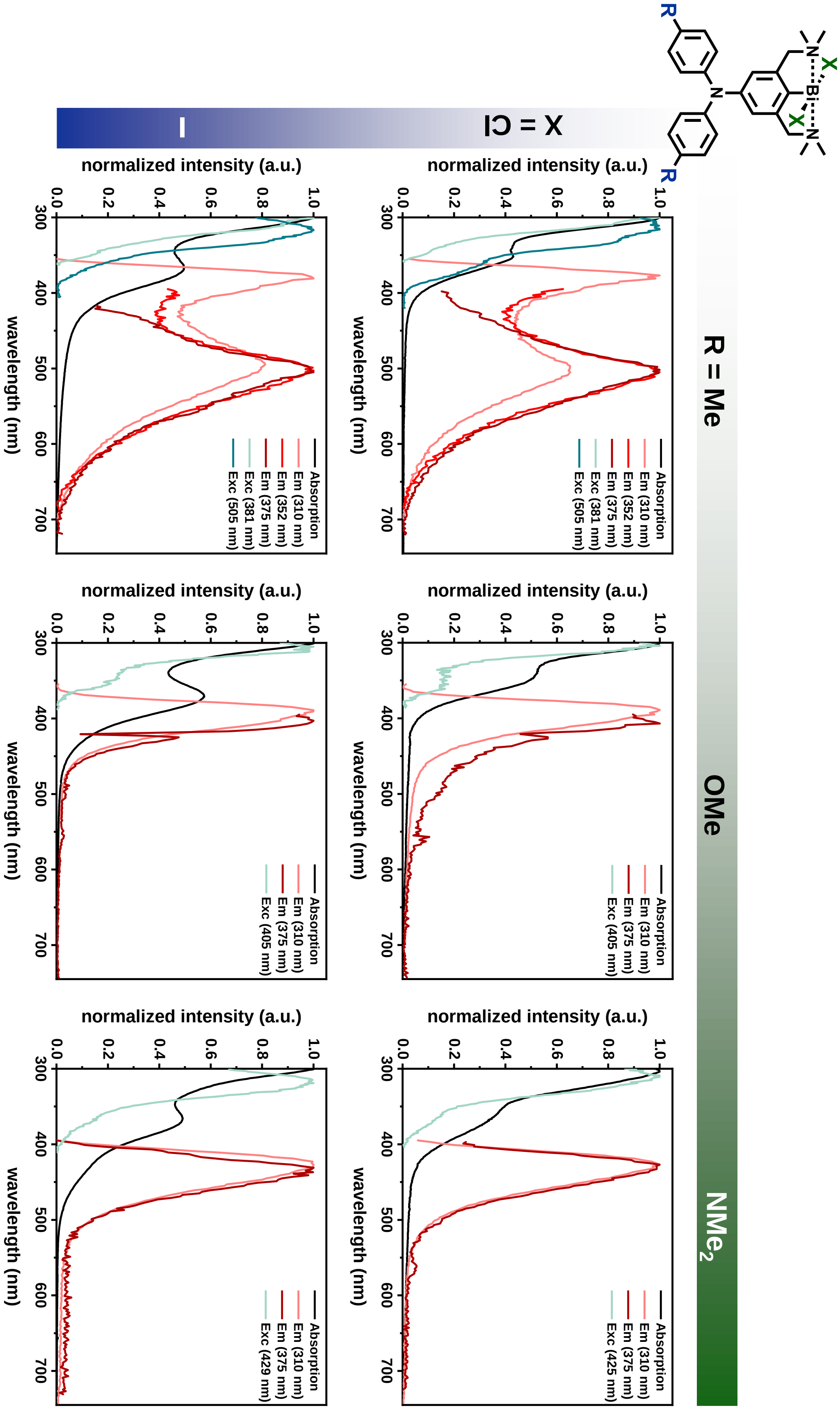

Figure S56. PL data of 10 μM solutions of complexes **(*NC^RN*)BiX$_2$** (R = Me, OMe, NMe$_2$; X = Cl, I) at r.t. in aerated THF. Absorption spectra are depicted as black, emission spectra as red, and excitation spectra as cyan lines.

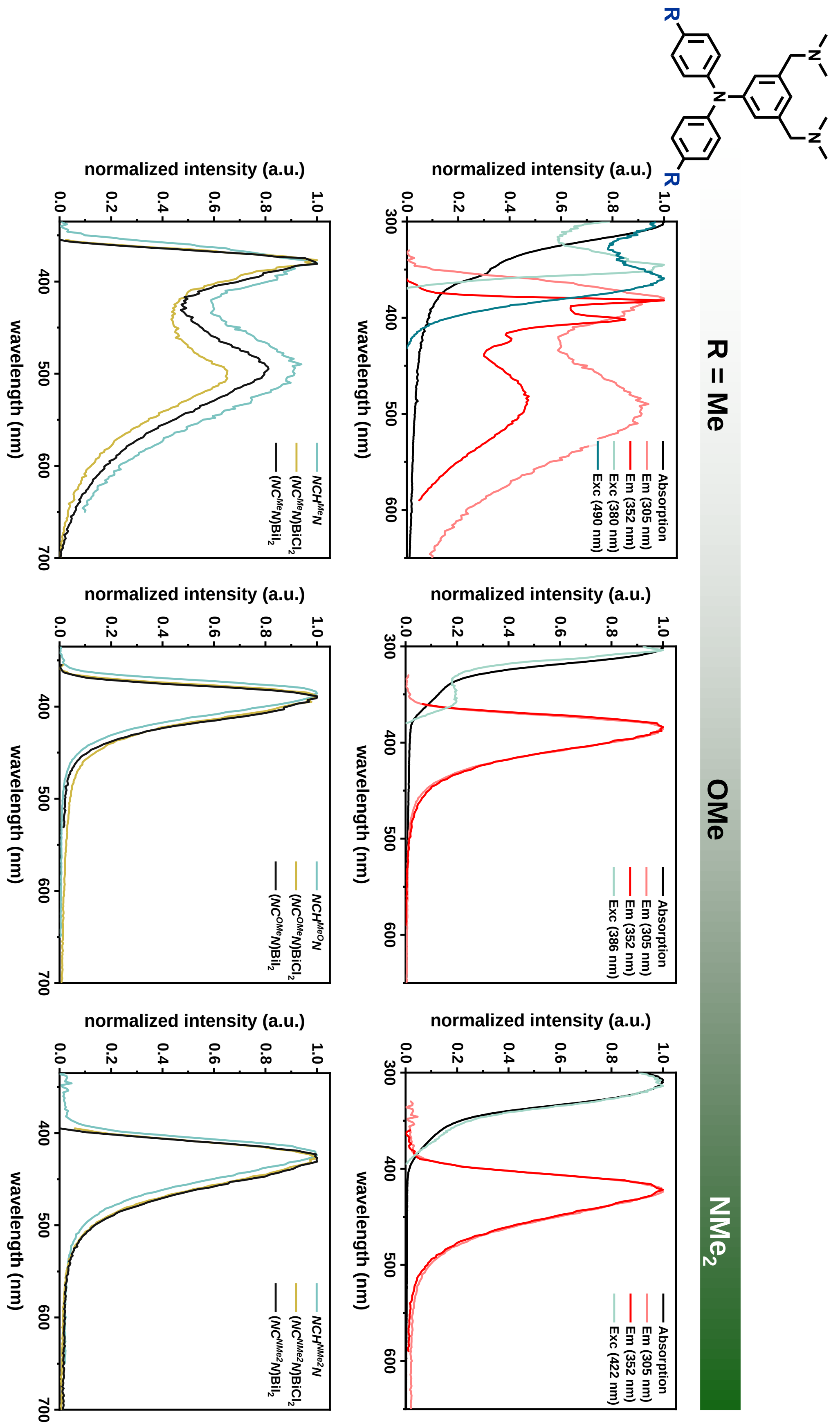

Figure S57. Top panel: PL data of 10 μM solutions of proligands ***NCH^RN*** (R = Me, OMe, NMe_2) at r.t. in THF. Absorption spectra are depicted as black, emission spectra as red, and excitation spectra as cyan lines. Bottom panel: Comparison of the emission spectra of the complexes **(*NC^RN*)BiX_2** (R = Me, OMe, NMe_2; X = Cl, I) with those of the corresponding proligands ***NCH^RN***.

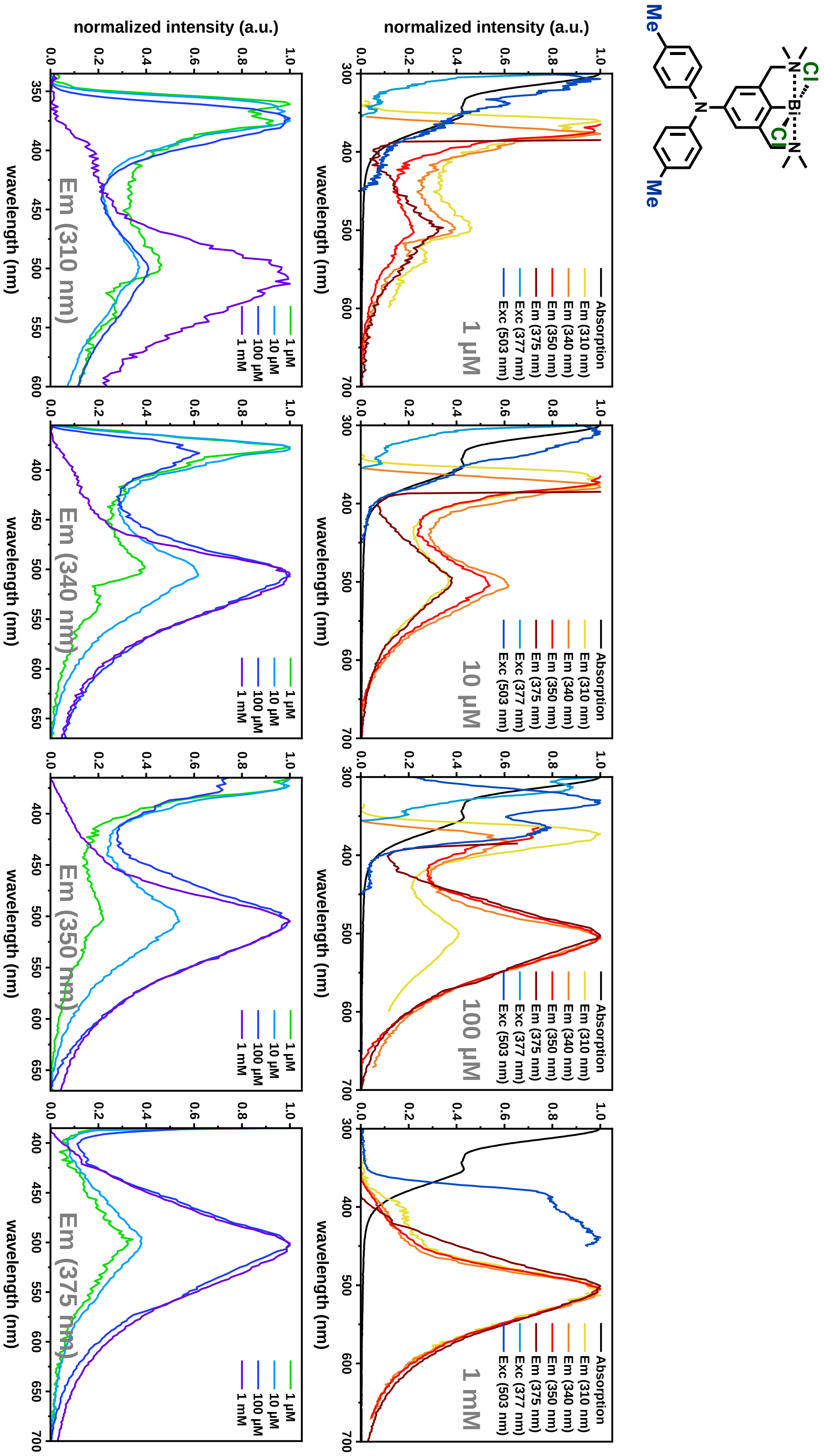

Figure S58. PL data of 1-1000 µM solutions of **(*NC*^{Me}*N*)BiCl$_2$** at r.t. in THF and at varied excitation wavelengths. Absorption spectra are depicted as black, emission spectra as yellow, orange and red, and excitation spectra as blue lines.

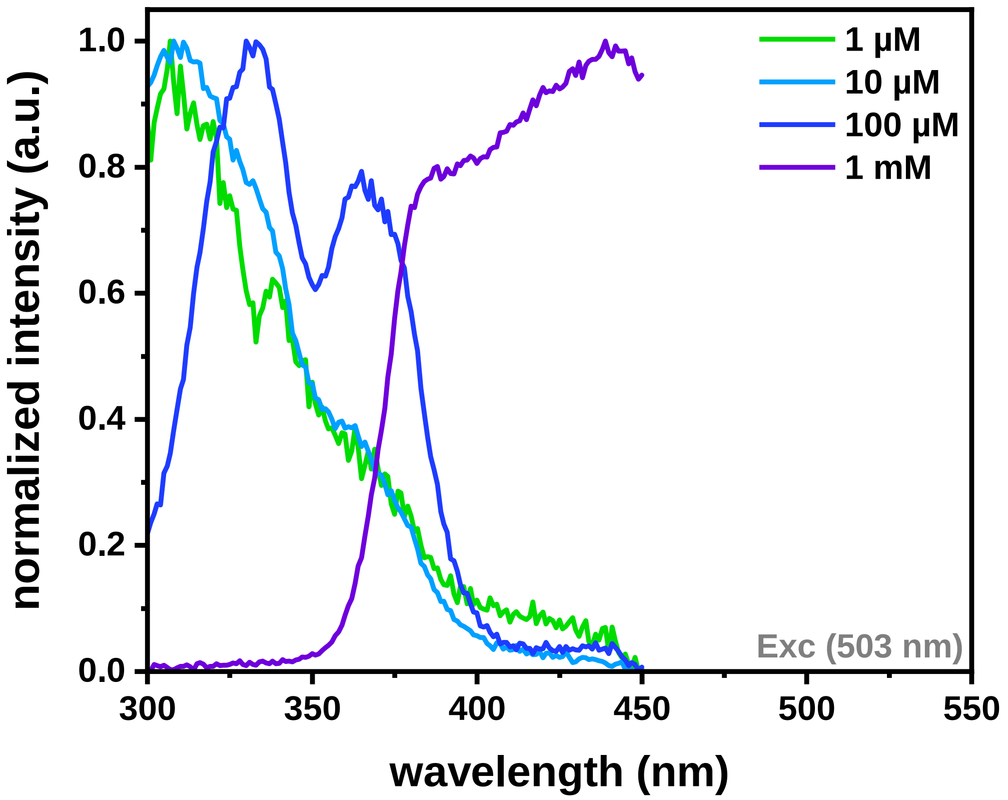

Figure S59. Excitation spectra of **(*NC^Me^N*)BiCl$_2$** at different concentrations (1-1000 µM) in THF at r.t., detected at λ = 503 nm

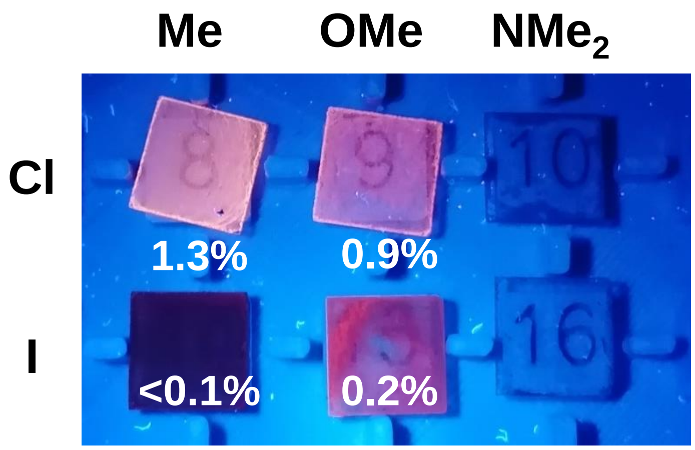

Figure S60. Picture of solid-state emission of films of **(*NC^R^N*)BiX$_2$** (R = Me, OMe, NMe$_2$; X = Cl, I) under ambient conditions with excitation at 365 nm.

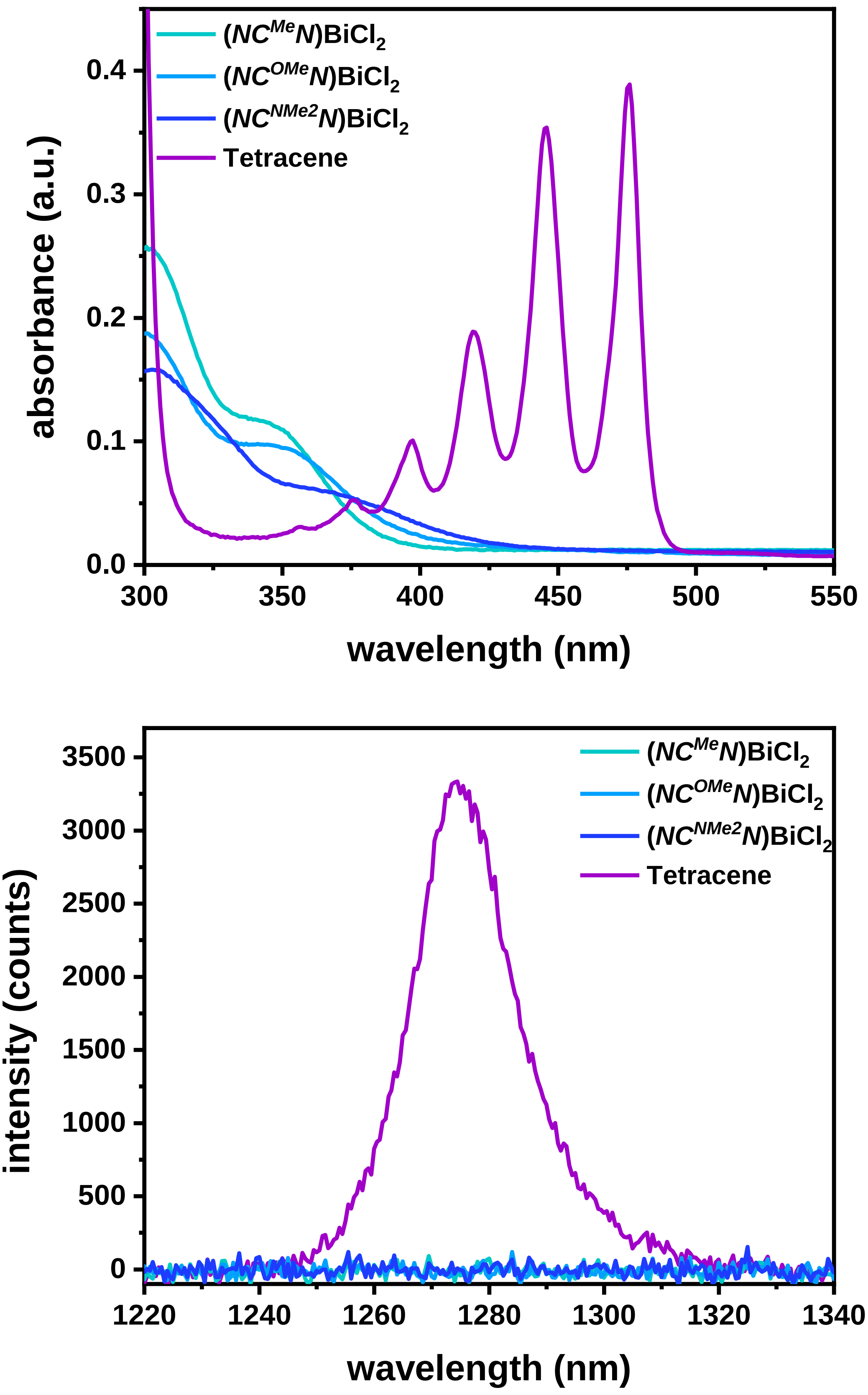

Figure S61. Top: UV/vis absorption spectra of **(*NC*^{R}*N*)BiCl$_2$** (R = Me, OMe, NMe$_2$) and tetracene in CH_2Cl_2 at r.t. prepared for $^1O_2$-QY measurement. Bottom: $^1O_2$-QY measurements. Emission spectra in NIR region of **(*NC*^{R}*N*)BiCl$_2$** (R = Me, OMe, NMe$_2$) and tetracene excited at 370 nm.

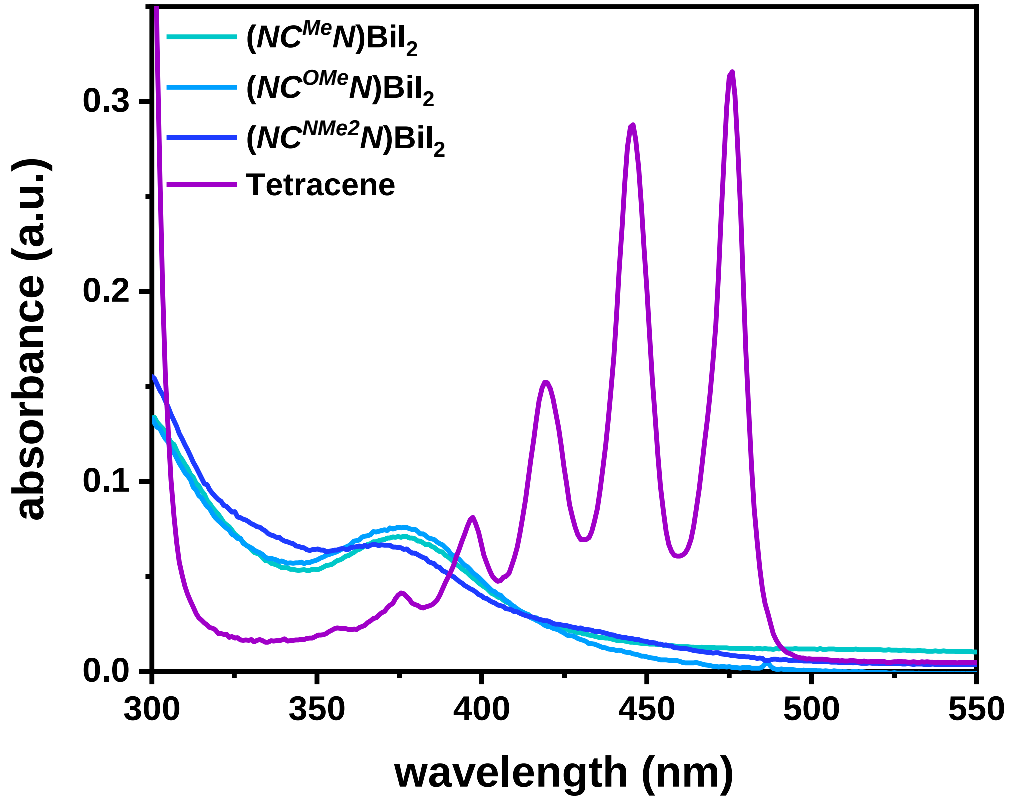

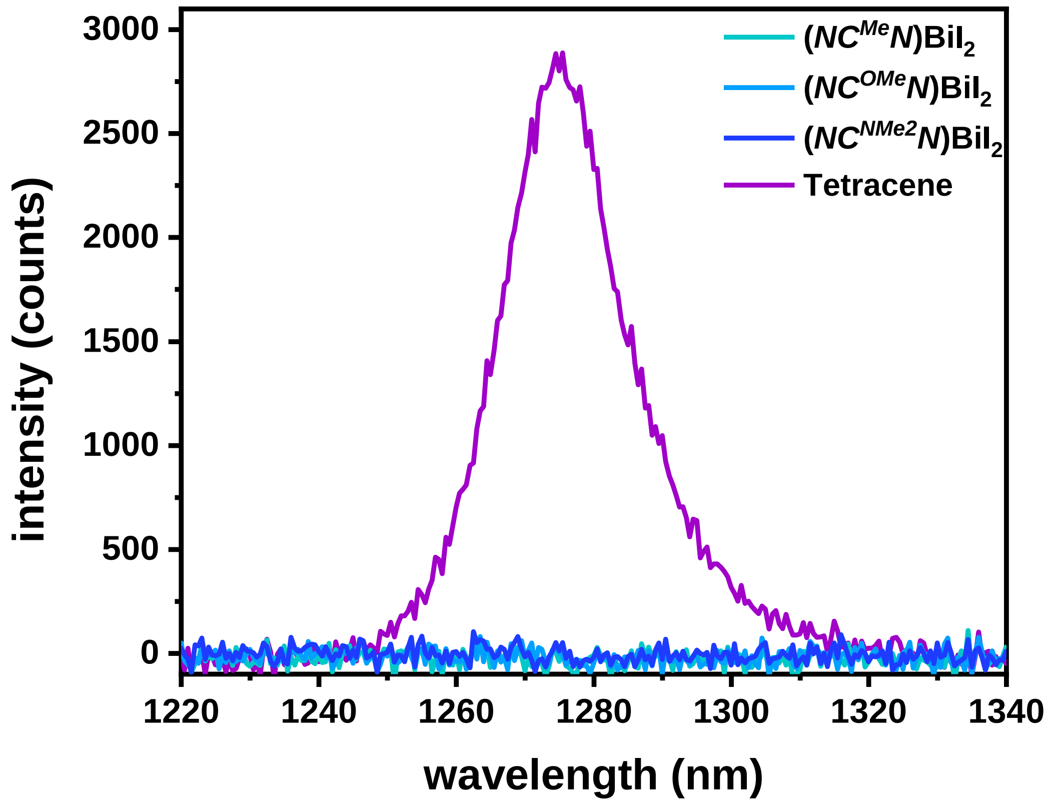

Figure S62. Top: UV/Vis absorption spectra of ***(NC^{R}N)*BiI$_2$** (R = Me, OMe, NMe_2) and tetracene in CH_2Cl_2 at r.t. prepared for $^1O_2$-QY measurement. Bottom: $^1O_2$-QY measurements. Emission spectra in NIR region of ***(NC^{R}N)*BiI$_2$** (R = Me, OMe, NMe_2) and tetracene excited at 390 nm.

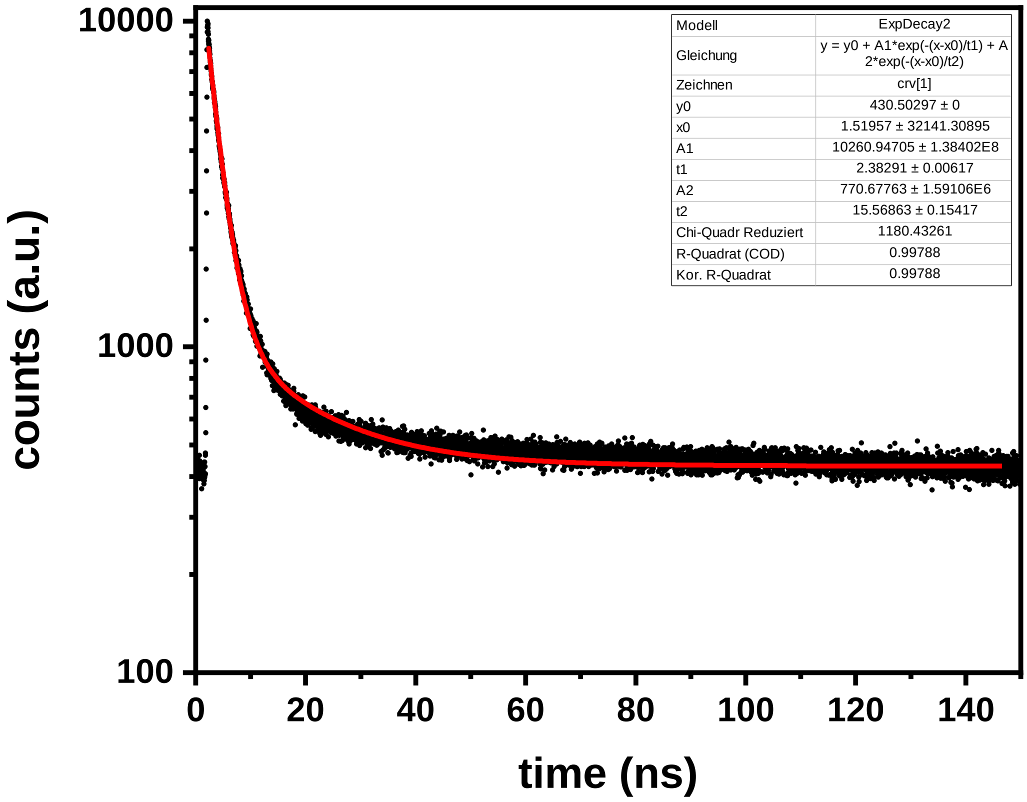

Figure S63. Lifetime of the 435 nm emission of ***NCH^{Me}N*** in MeTHF at 77 K.

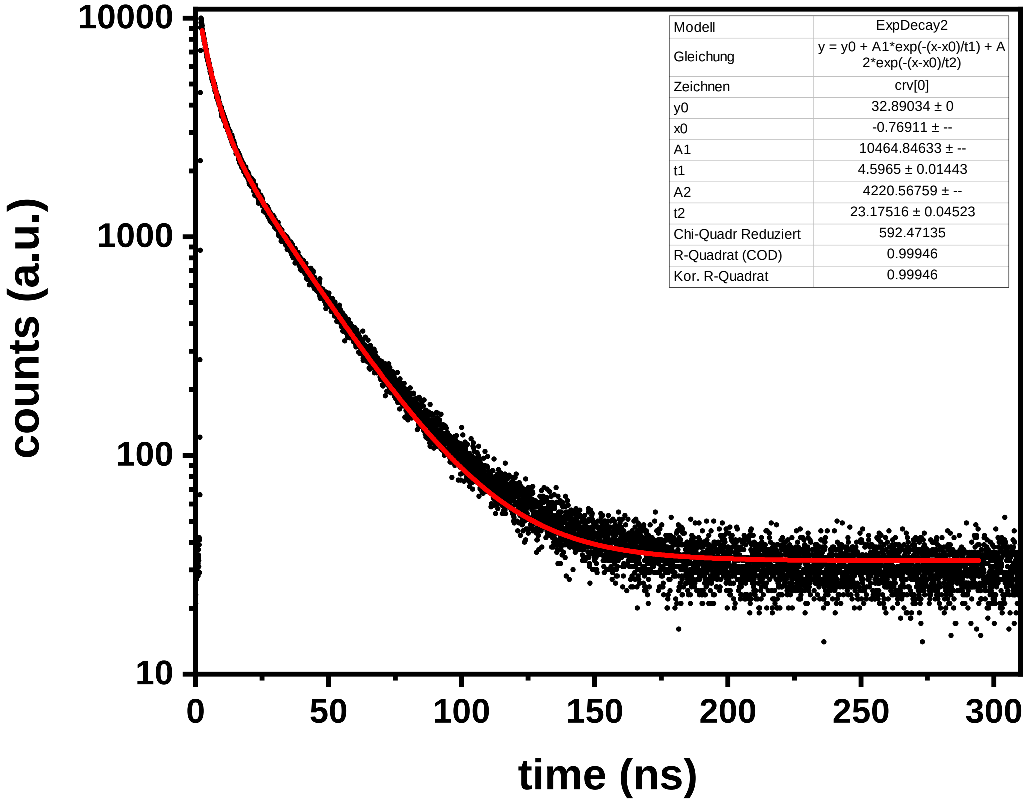

Figure S64. Lifetime of the 380 nm emission of ***NCH^{Me}N*** in THF at r.t.

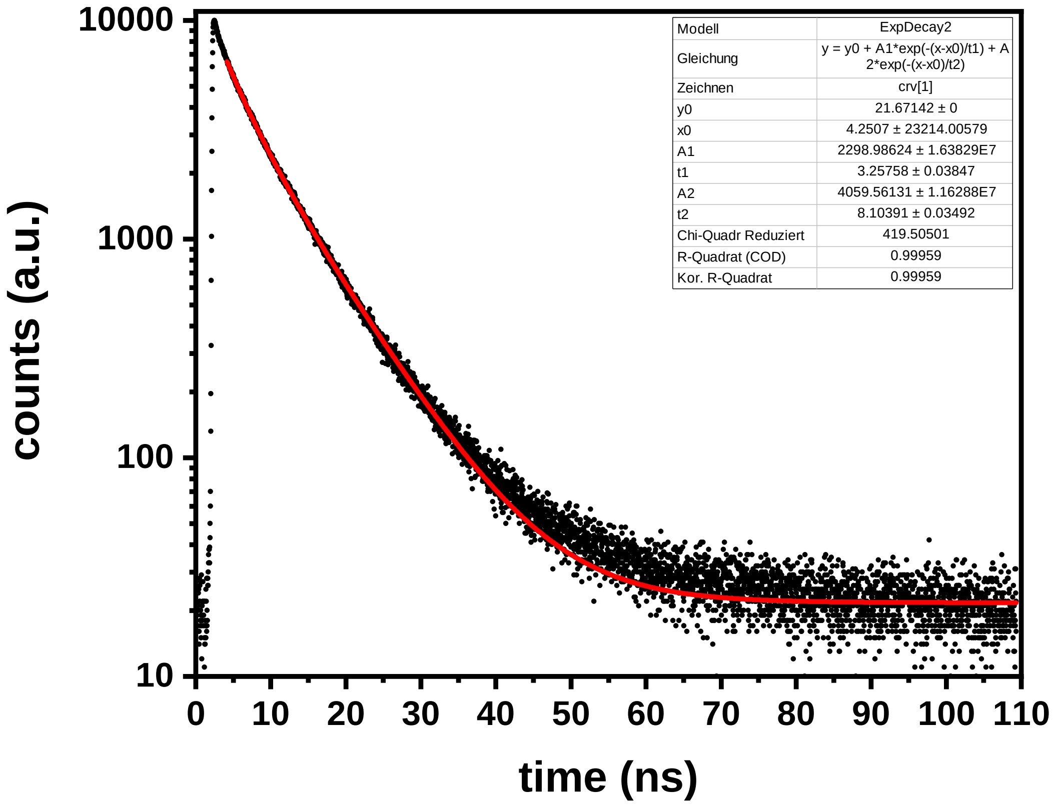

Figure S65. Lifetime of the 490 nm emission of ***NCH^{Me}N*** in THF at r.t.

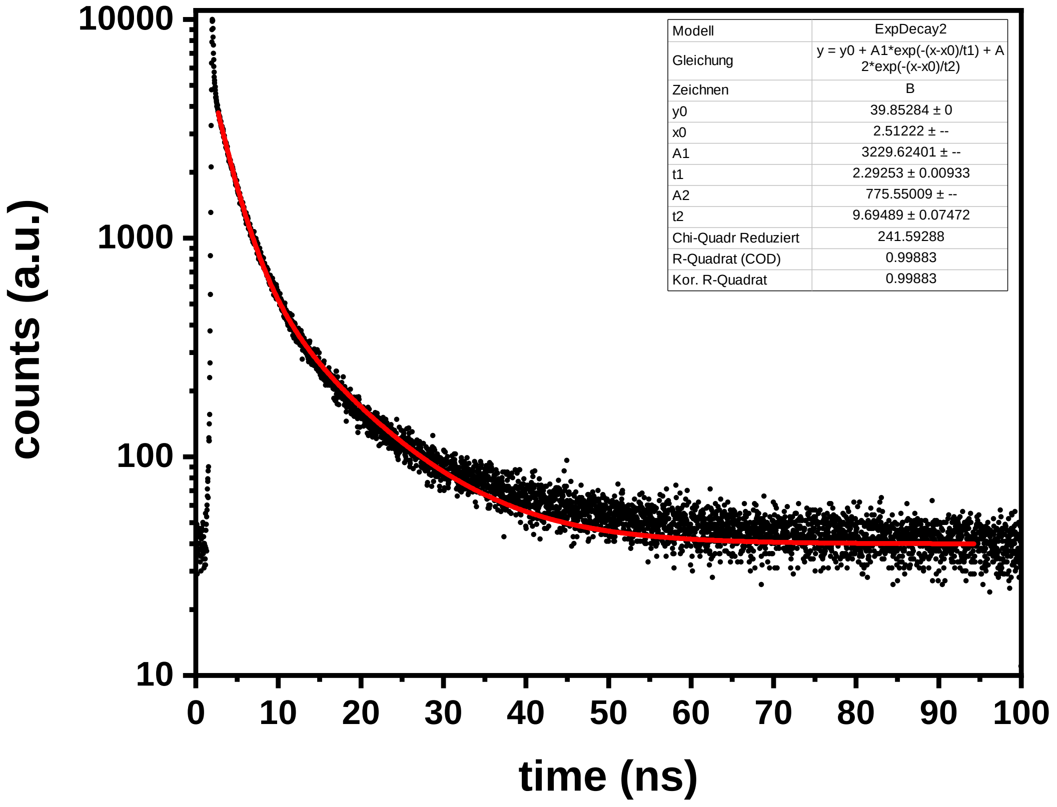

Figure S66. Lifetime of the 460 nm emission of **(*NC^{Me}N*)BiCl$_2$** in MeTHF at 77 K.

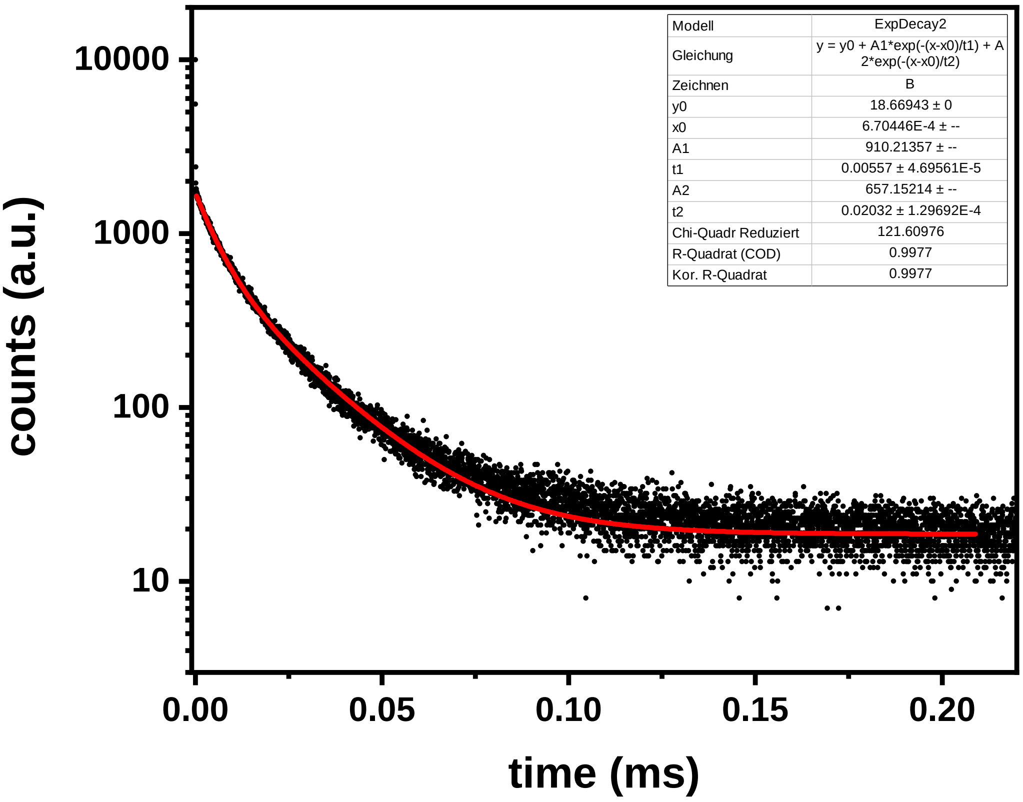

Figure S67. Lifetime of the 590 nm emission of **(*NC*^{Me}*N*)BiCl$_2$** in MeTHF at 77 K.

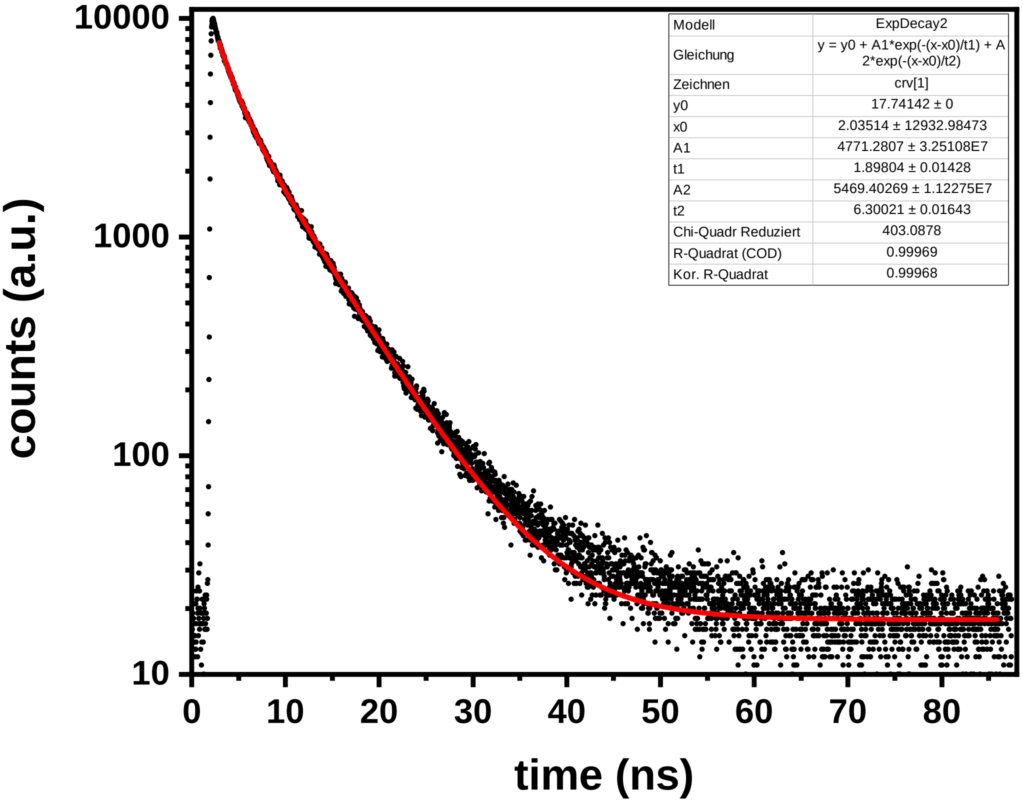

Figure S68. Lifetime of the 381 nm emission of **(*NC*^{Me}*N*)BiCl$_2$** in THF at r.t.

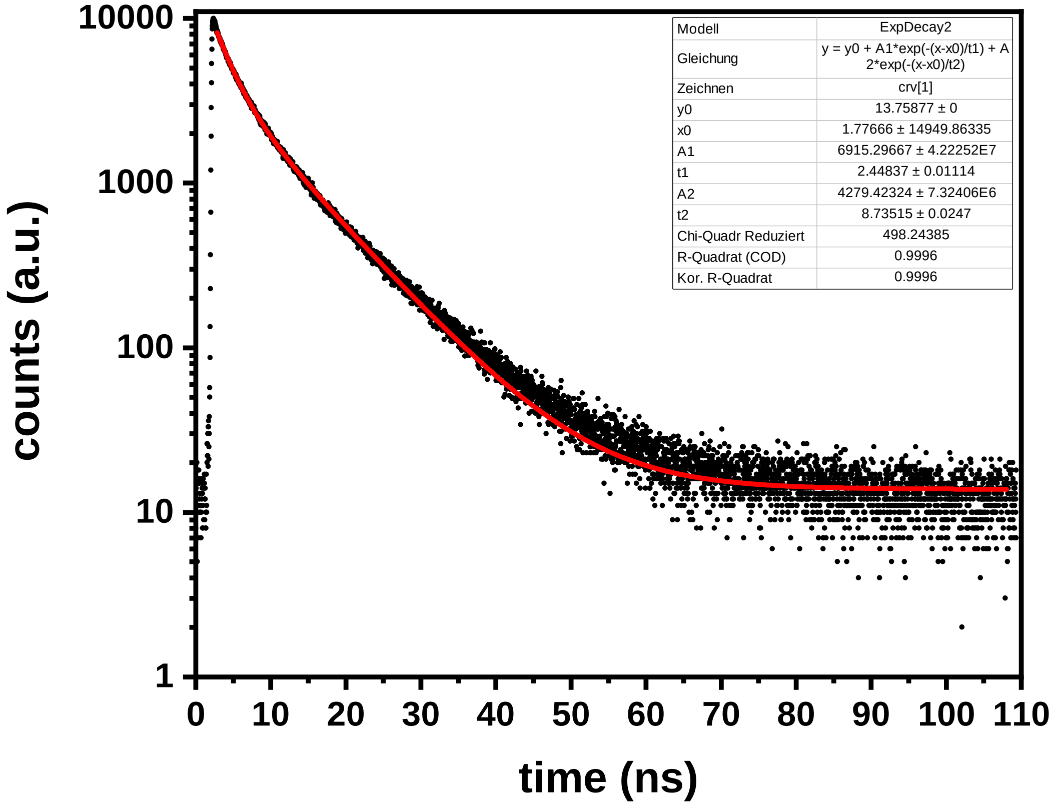

Figure S69. Lifetime of the 505 nm emission of **($NC^{Me}N$)$BiCl_2$** in THF at r.t.

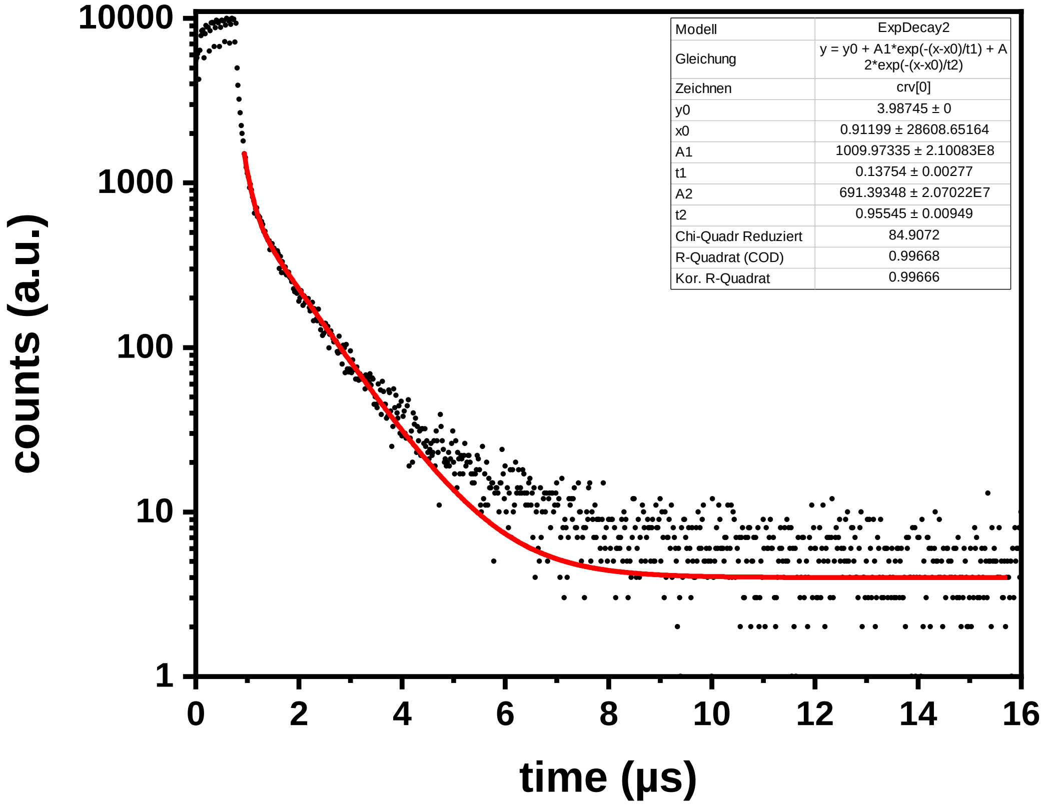

Figure S70. Lifetime of the 630 nm emission of **($NC^{Me}N$)$BiCl_2$** in the solid state at r.t.

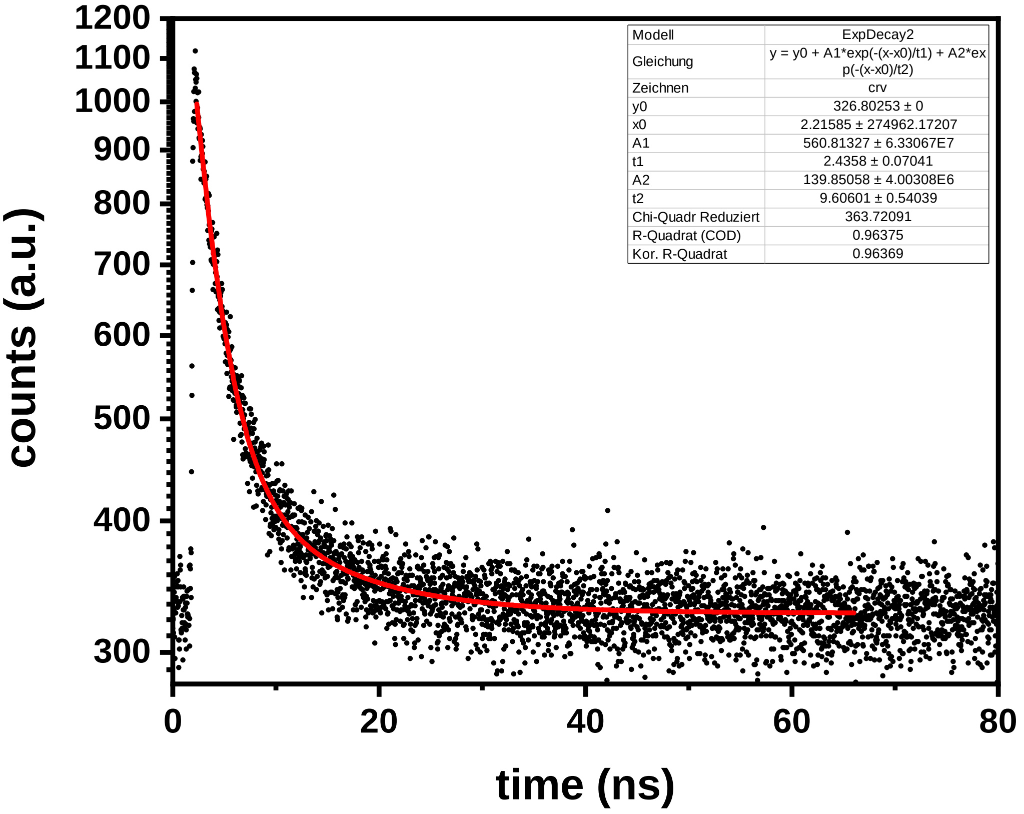

Figure S71. Lifetime of the 445 nm emission of **(*NC*^{Me}*N*)BiI$_2$** in MeTHF at 77 K.

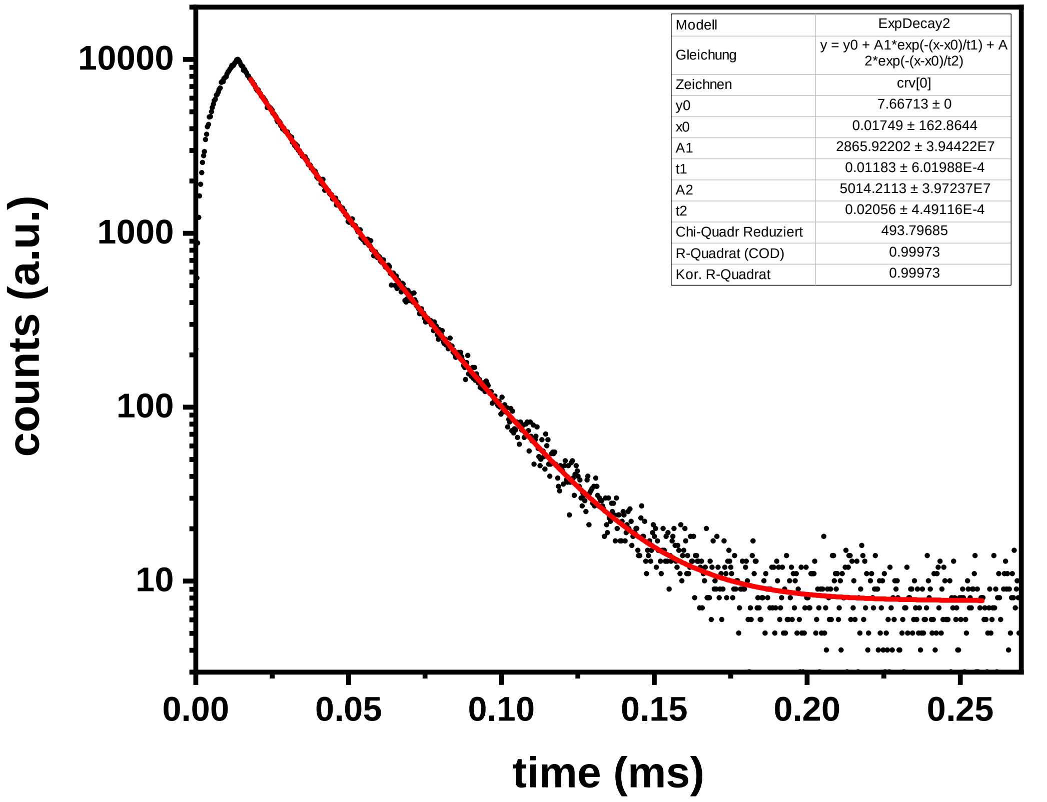

Figure S72. Lifetime of the 445 nm emission of **(*NC*^{Me}*N*)BiI$_2$** in MeTHF at 77 K.

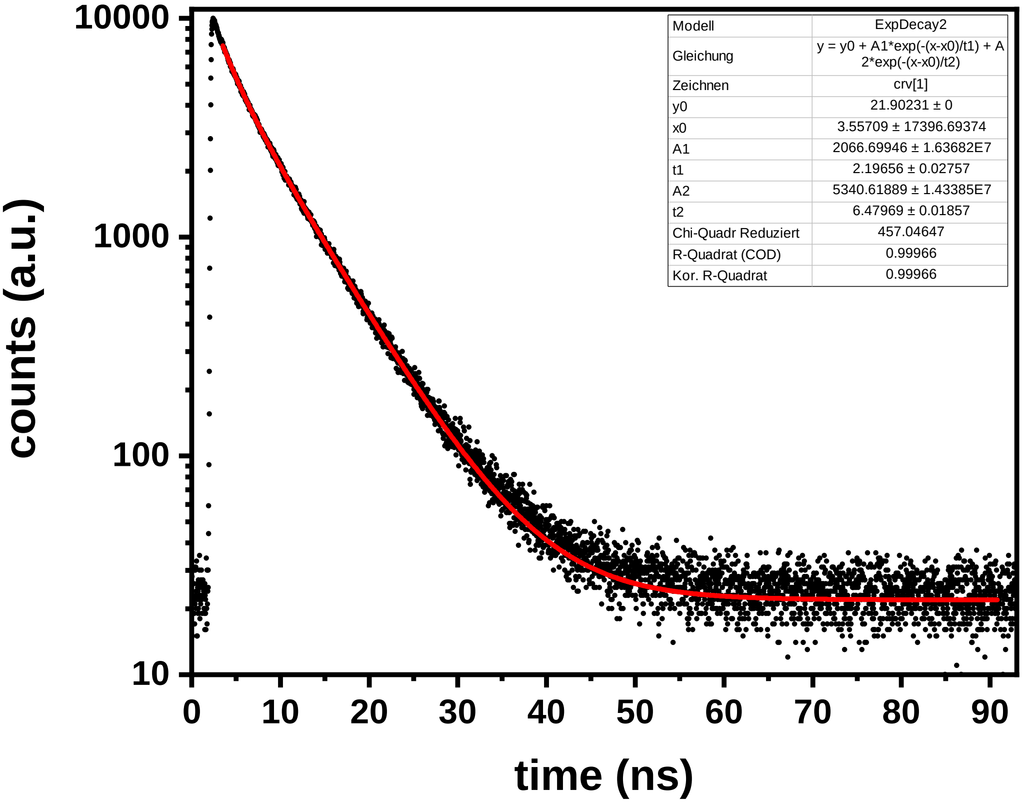

Figure S73. Lifetime of the 381 nm emission of **(*NC*^{Me}*N*)BiI$_2$** in THF at r.t.

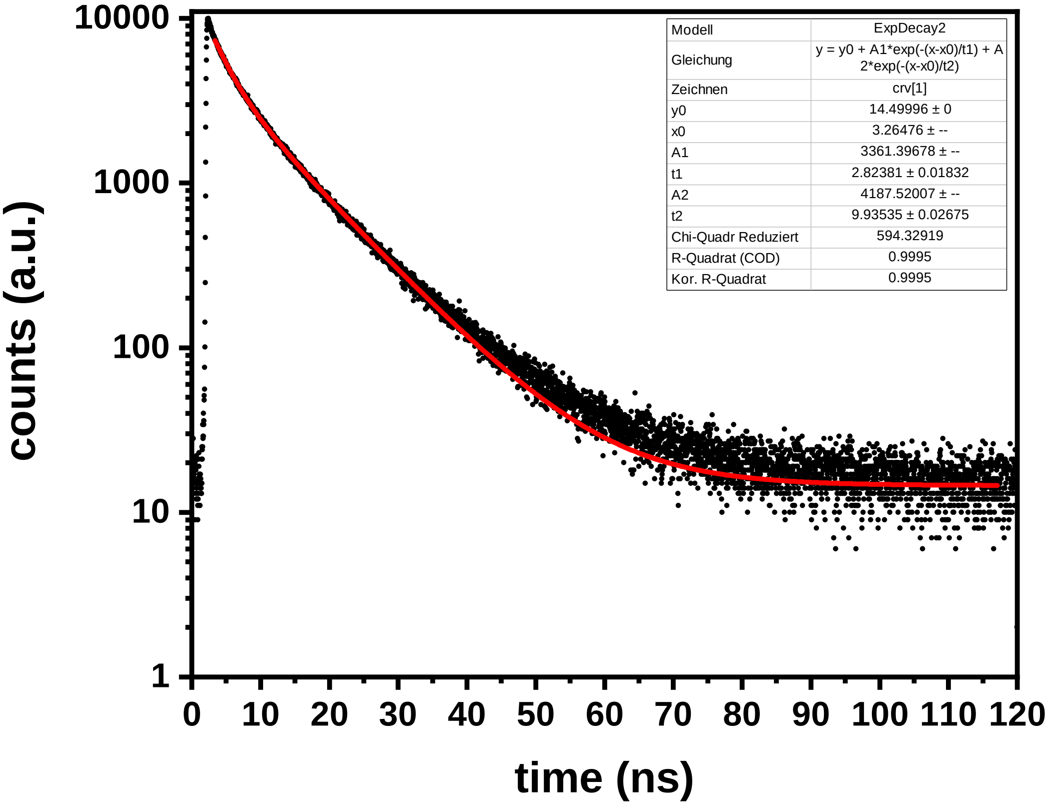

Figure S74. Lifetime of the 505 nm emission of **(*NC*^{Me}*N*)BiI$_2$** in THF at r.t.

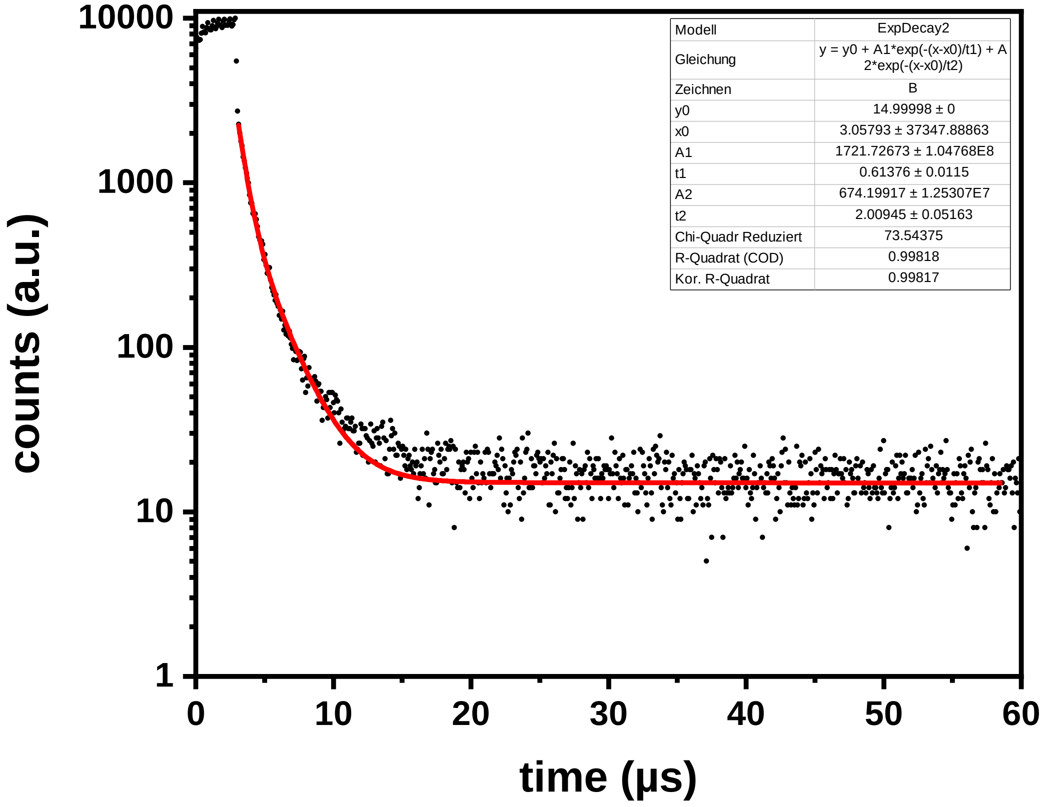

Figure S75. Lifetime of the 655 nm emission of **(*NC*^{Me}*N*)BiI$_2$** in the solid state at r.t.

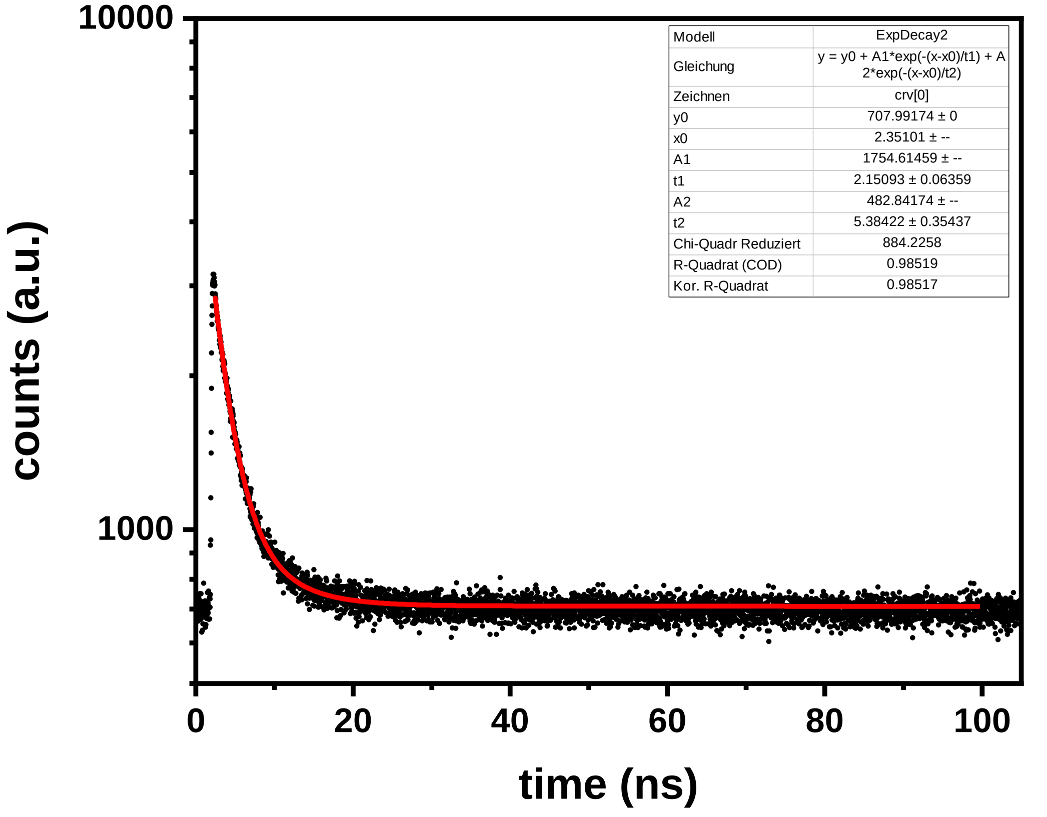

Figure S76. Lifetime of the 435 nm emission of ***NCH*^{OMe}*N*** in MeTHF at 77 K.

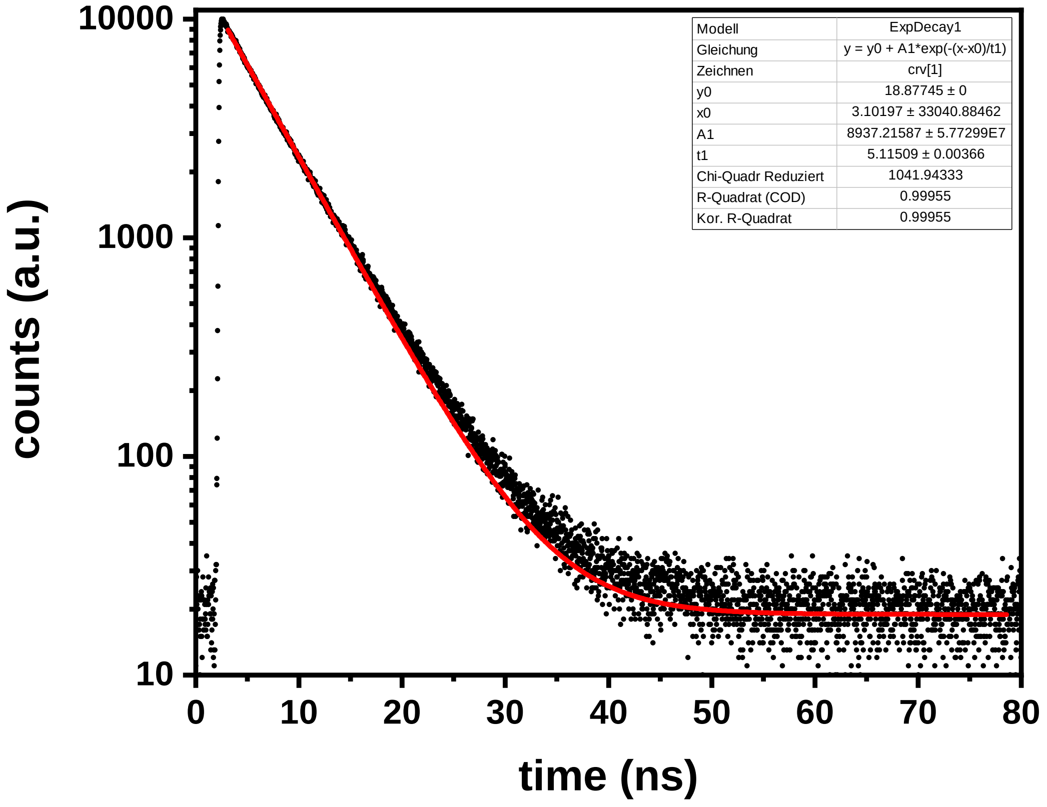

Figure S77. Lifetime of the 386 nm emission of ***NCH^{OMe}N*** in THF at r.t.

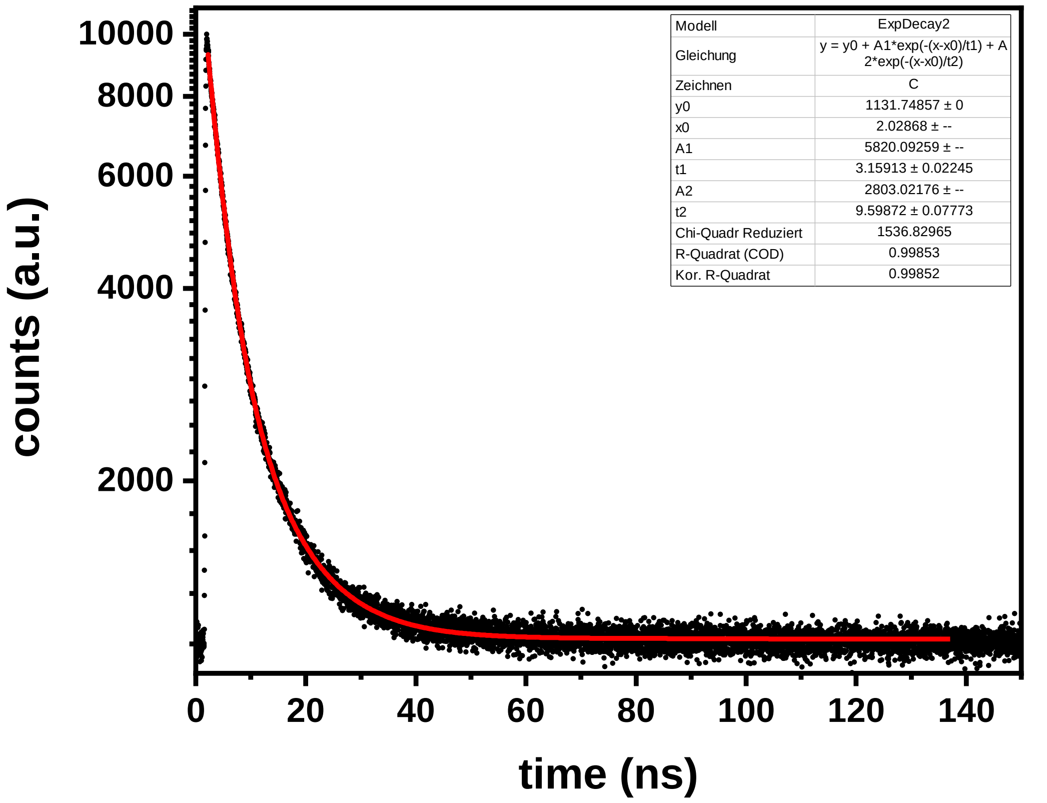

Figure S78. Lifetime of the 445 nm emission of **(*NC^{OMe}N*)BiCl$_2$** in MeTHF at 77 K.

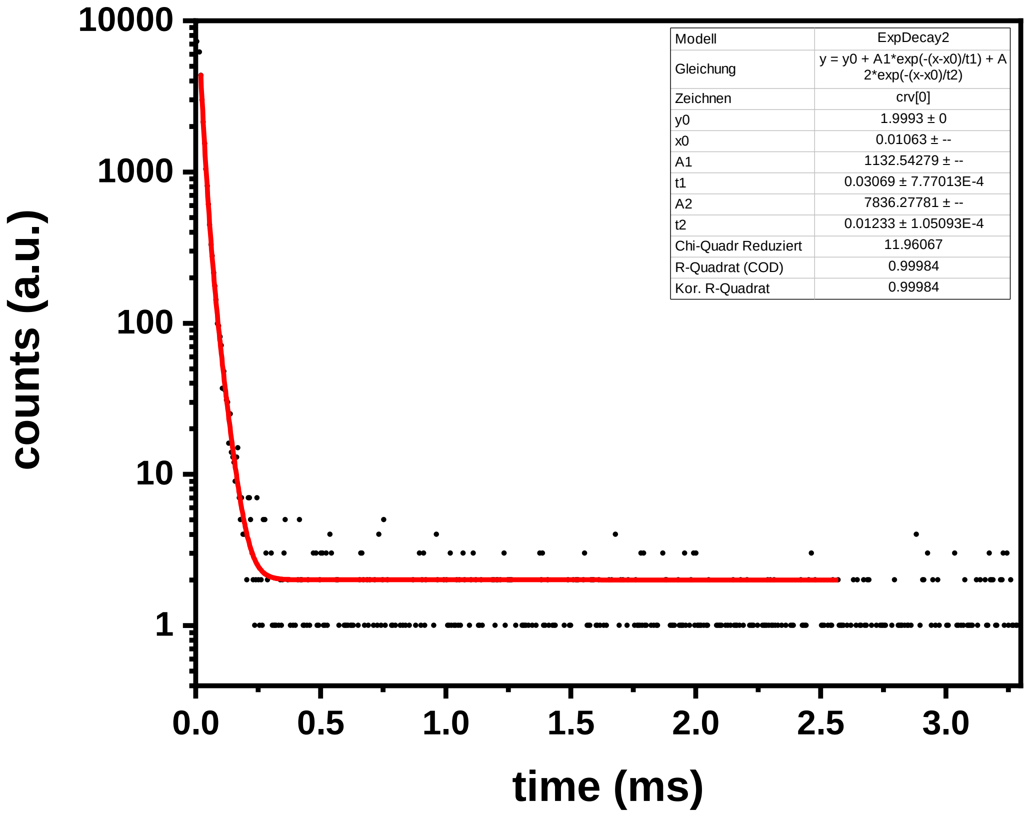

Figure S79. Lifetime of the 605 nm emission of **(*NC*^{OMe}*N*)BiCl$_2$** in MeTHF at 77 K.

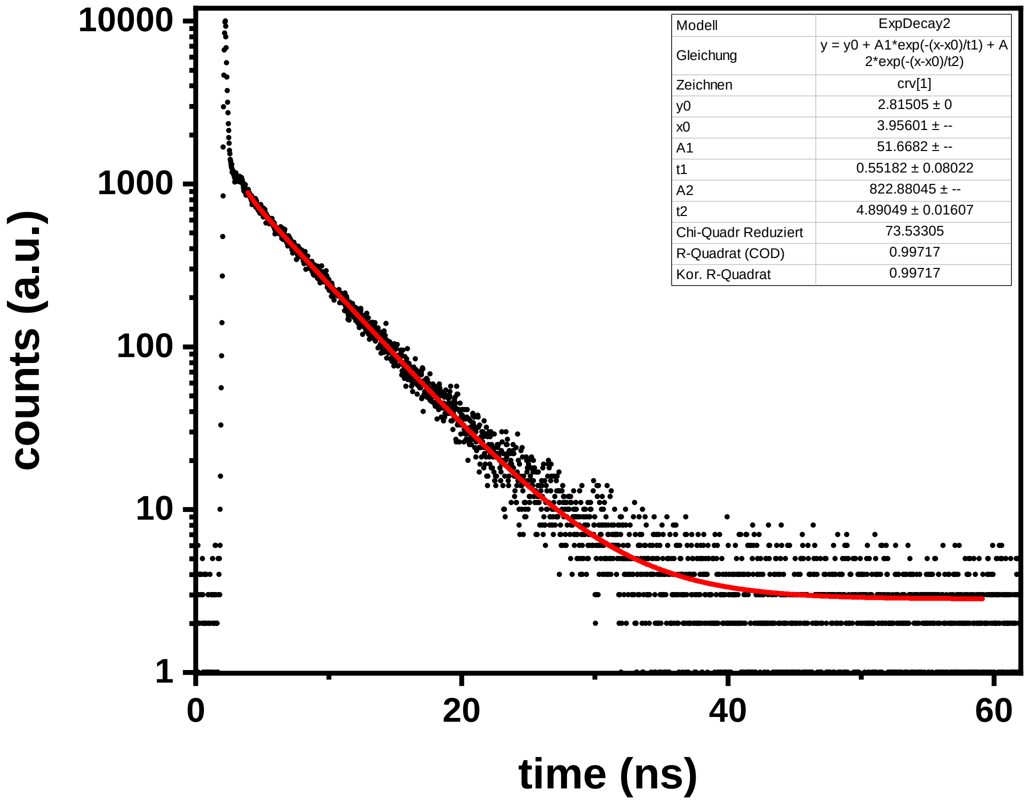

Figure S80. Lifetime of the 395 nm emission of **(*NC*^{OMe}*N*)BiCl$_2$** in THF at r.t.

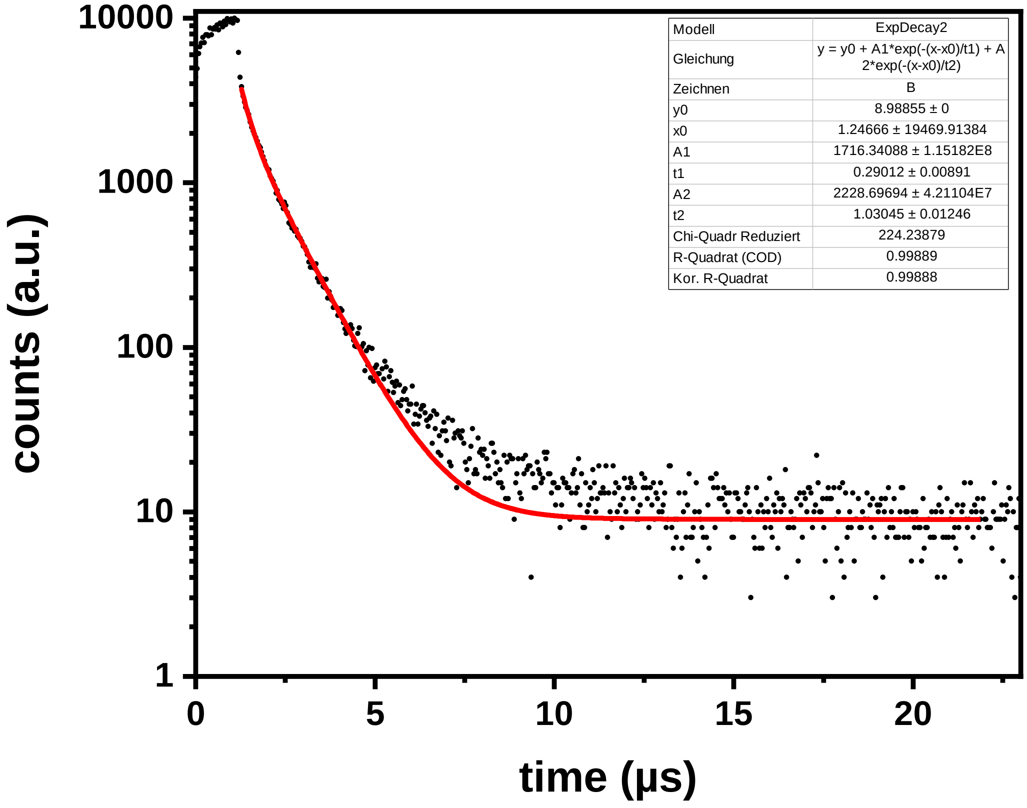

Figure S81. Lifetime of the 615 nm emission of **(*NC*^{OMe}*N*)BiCl$_2$** in the solid state at r.t.

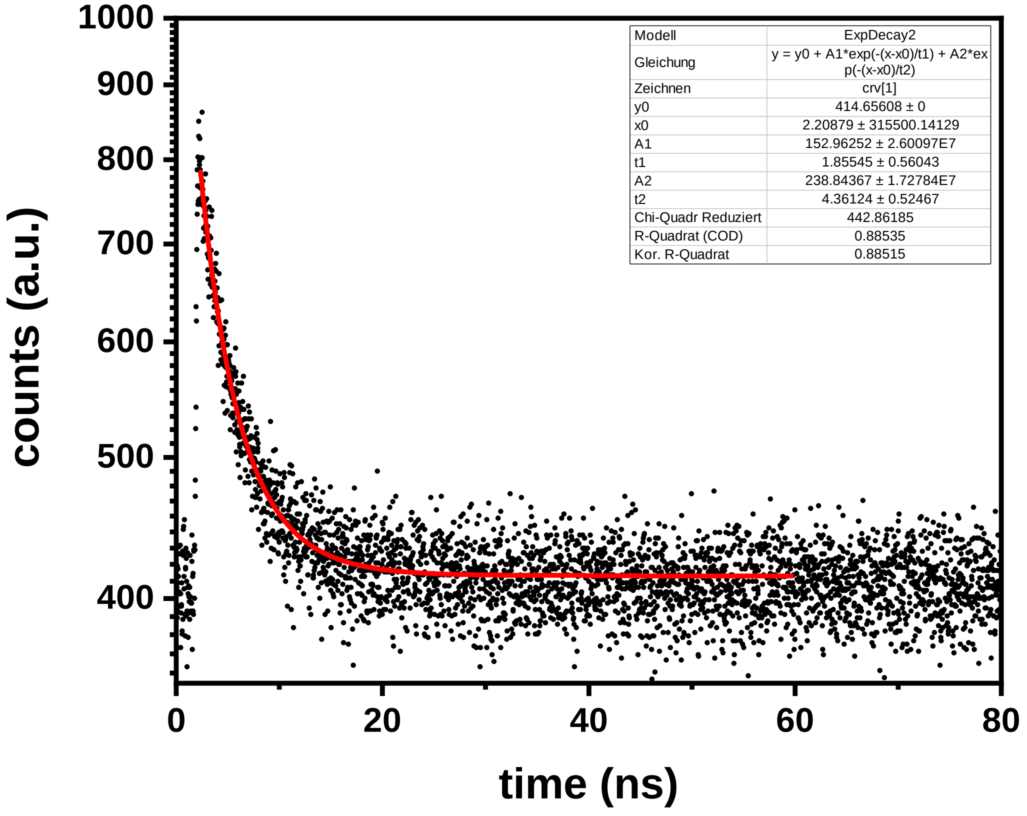

Figure S82. Lifetime of the 450 nm emission of **(*NC*^{OMe}*N*)BiI$_2$** in MeTHF at 77 K.

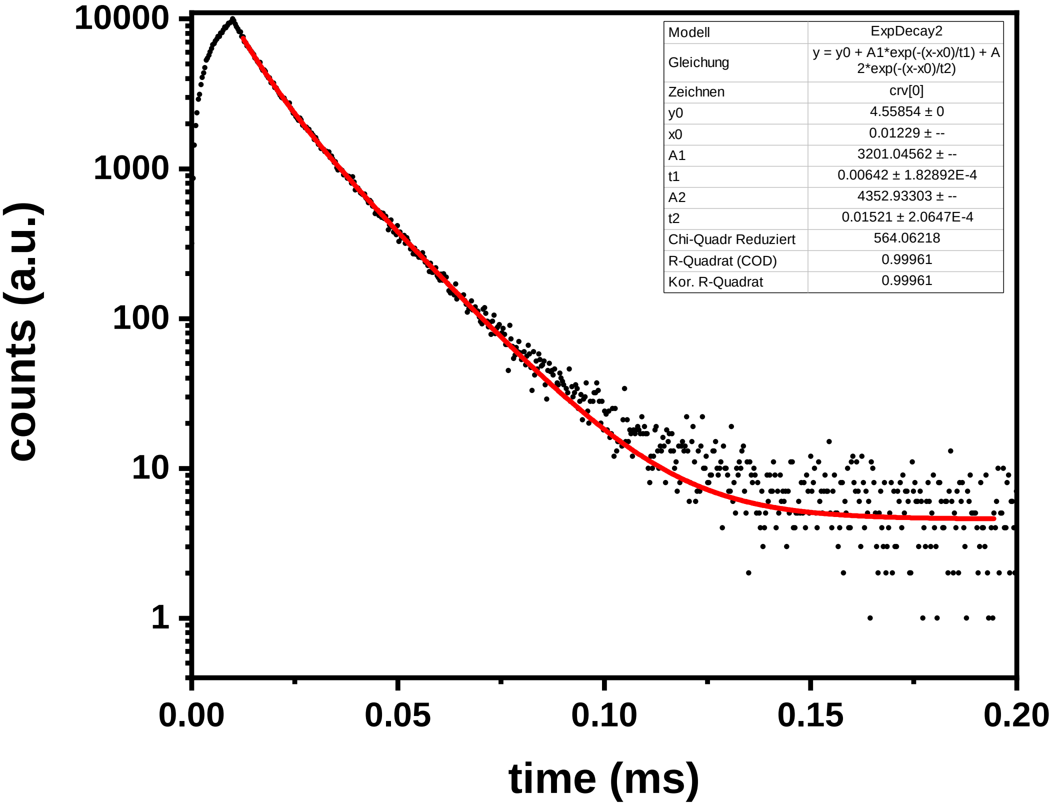

Figure S83. Lifetime of the 655 nm emission of **(*NC*^{OMe}*N*)BiI$_2$** in MeTHF at 77 K.

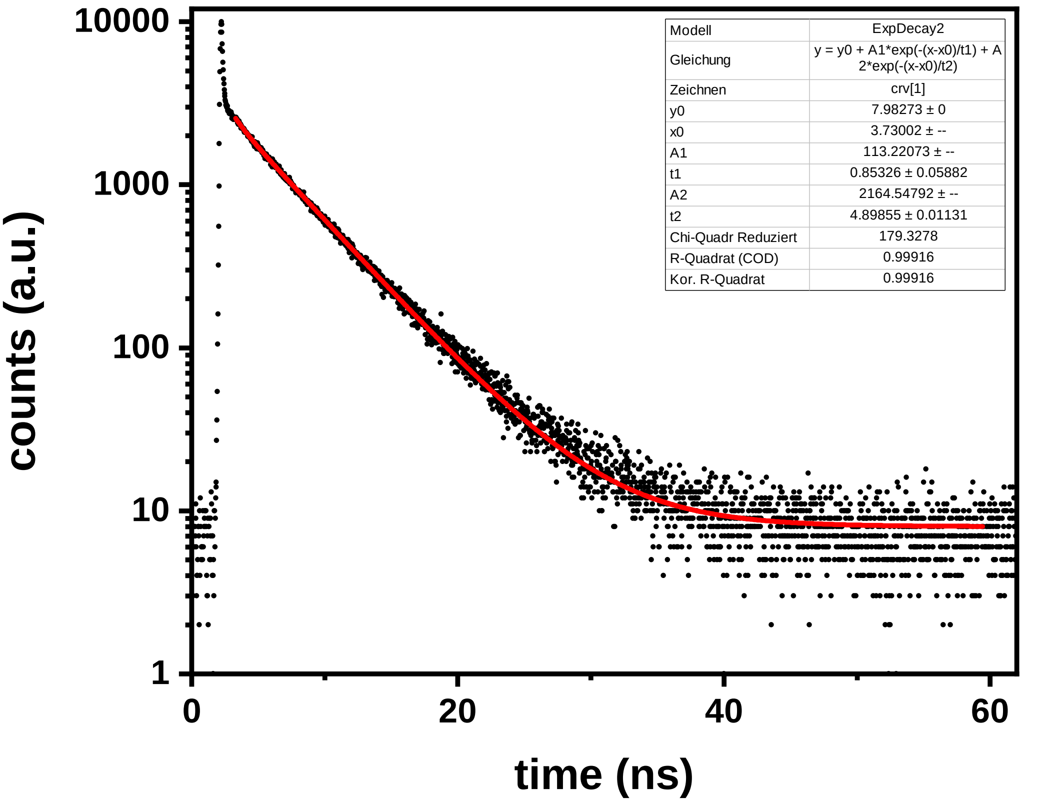

Figure S84. Lifetime of the 395 nm emission of **(*NC*^{OMe}*N*)BiI$_2$** in THF at r.t.

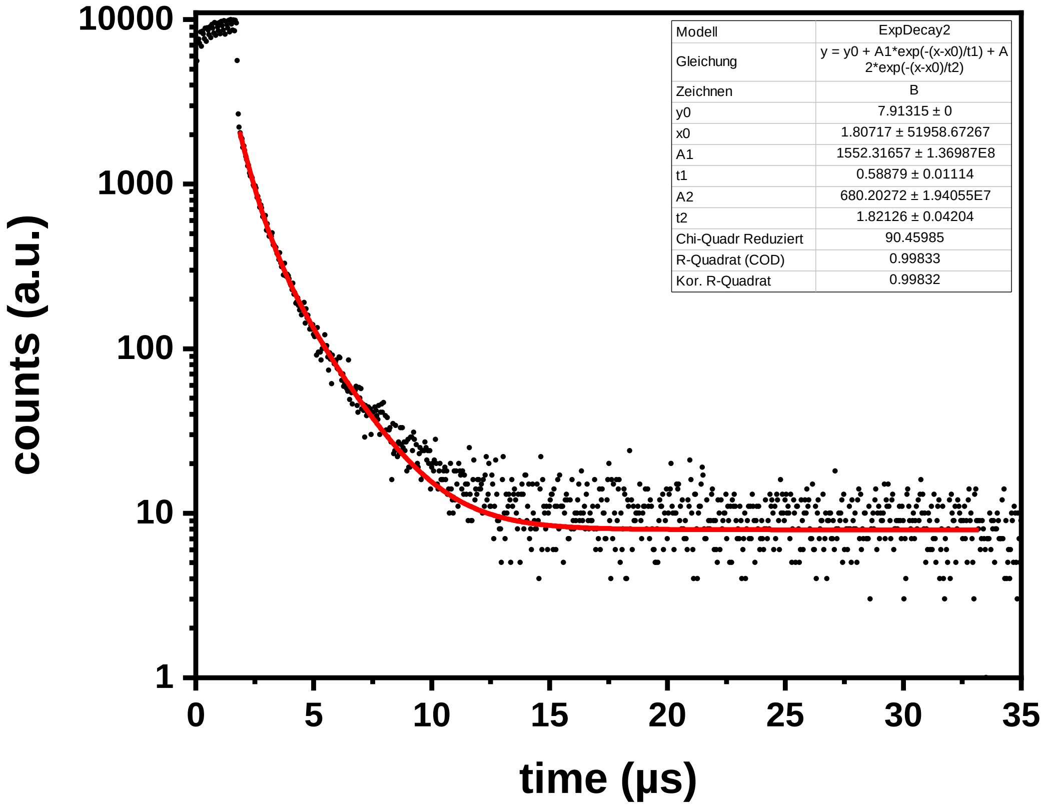

Figure S85. Lifetime of the 720 nm emission of **(*NC*$^{\boldsymbol{OMe}}$*N*)BiI$_2$** in the solid state at r.t.

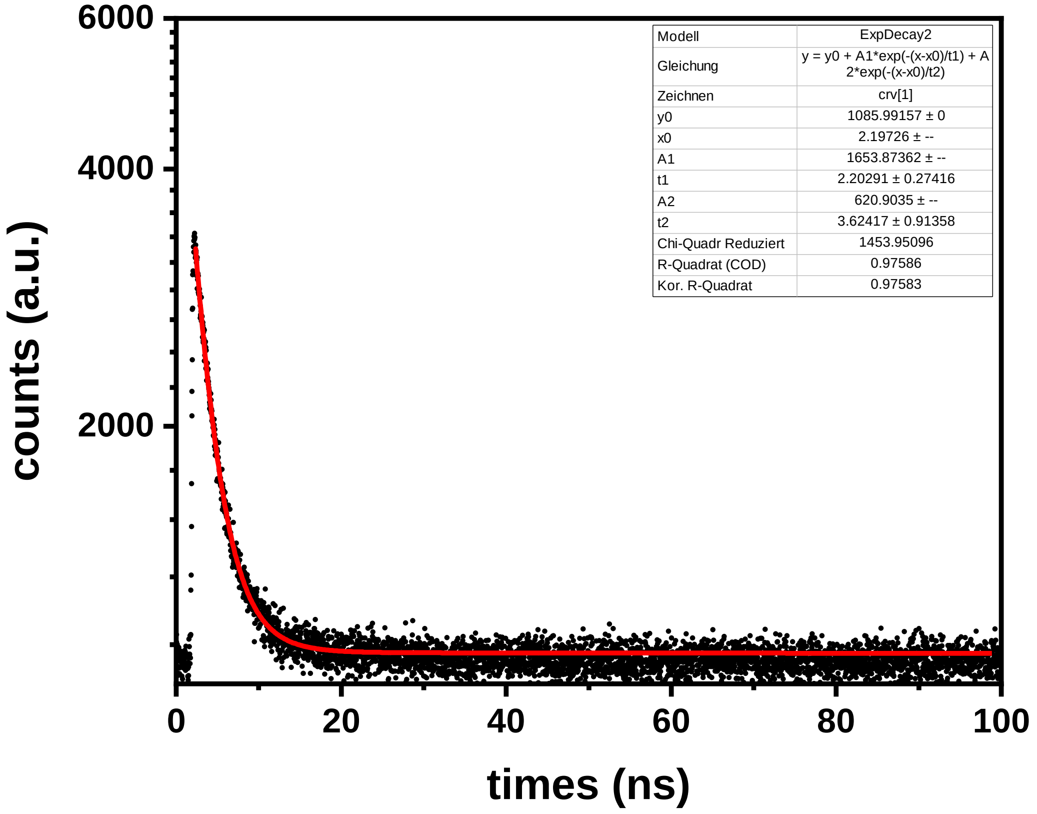

Figure S86. Lifetime of the 450 nm emission of ***NCH*$^{\boldsymbol{NMe2}}$*N*** in MeTHF at 77 K.

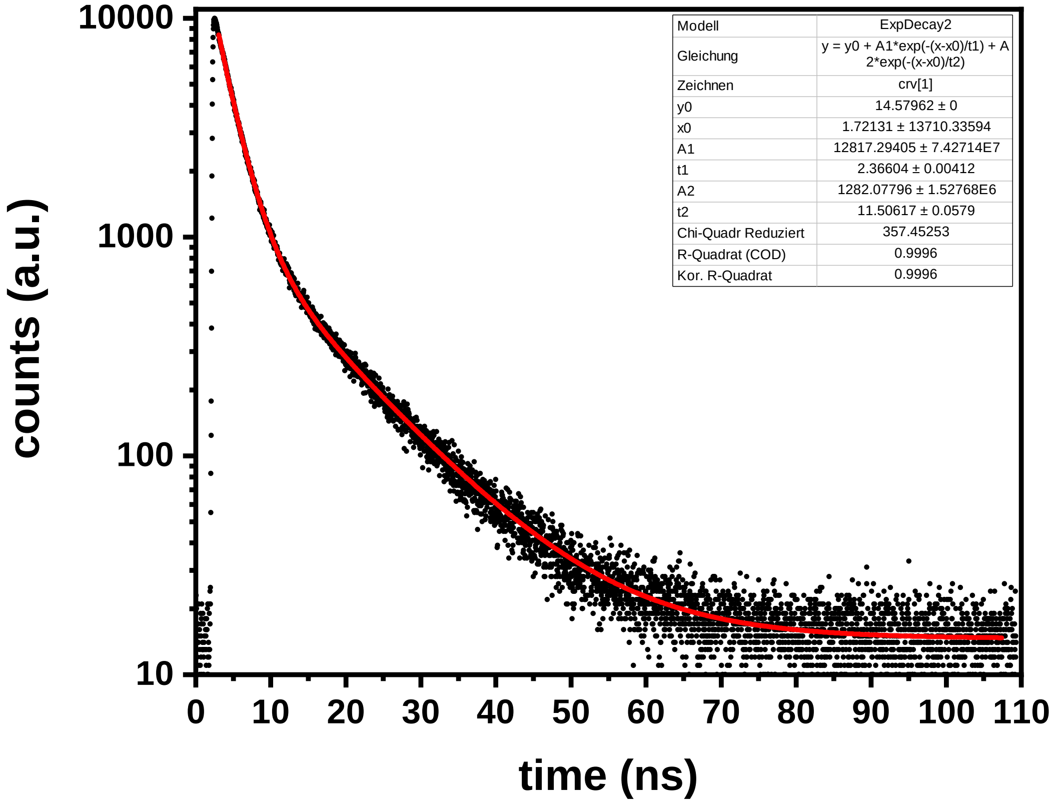

Figure S87. Lifetime of the 422 nm emission of ***NCH***$^{\boldsymbol{NMe2}}$***N*** in THF at r.t.

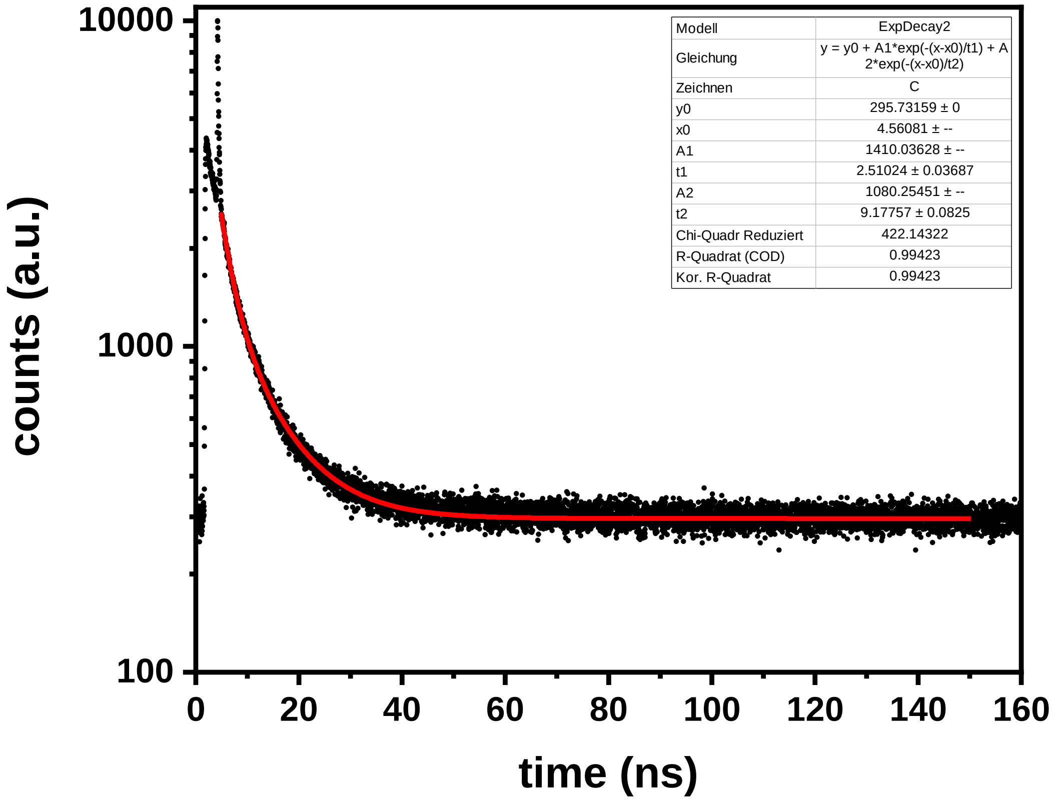

Figure S88. Lifetime of the 470 nm emission of **(*NC*$^{\boldsymbol{NMe2}}$*N*)BiCl$_2$** in MeTHF at 77 K.

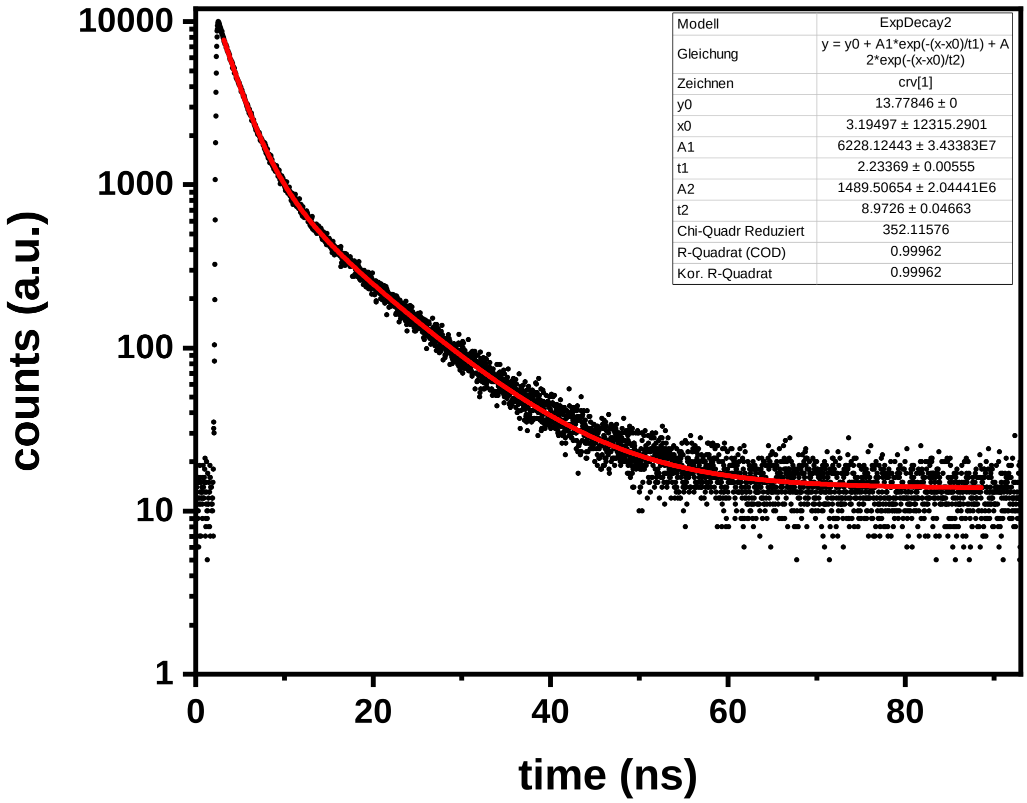

Figure S89. Lifetime of the 425 nm emission of **(*NC*^{NMe2}*N*)BiCl$_2$** in THF at r.t.

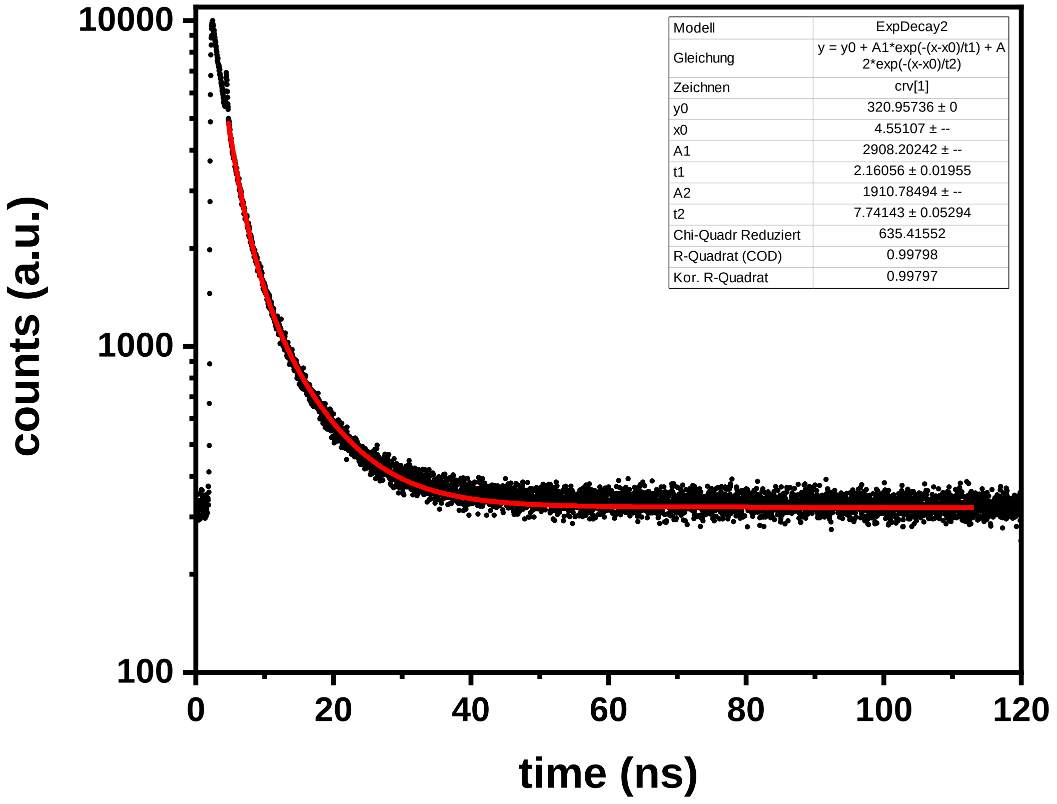

Figure S90. Lifetime of the 460 nm emission of **(*NC*^{NMe2}*N*)BiI$_2$** in MeTHF at 77 K.

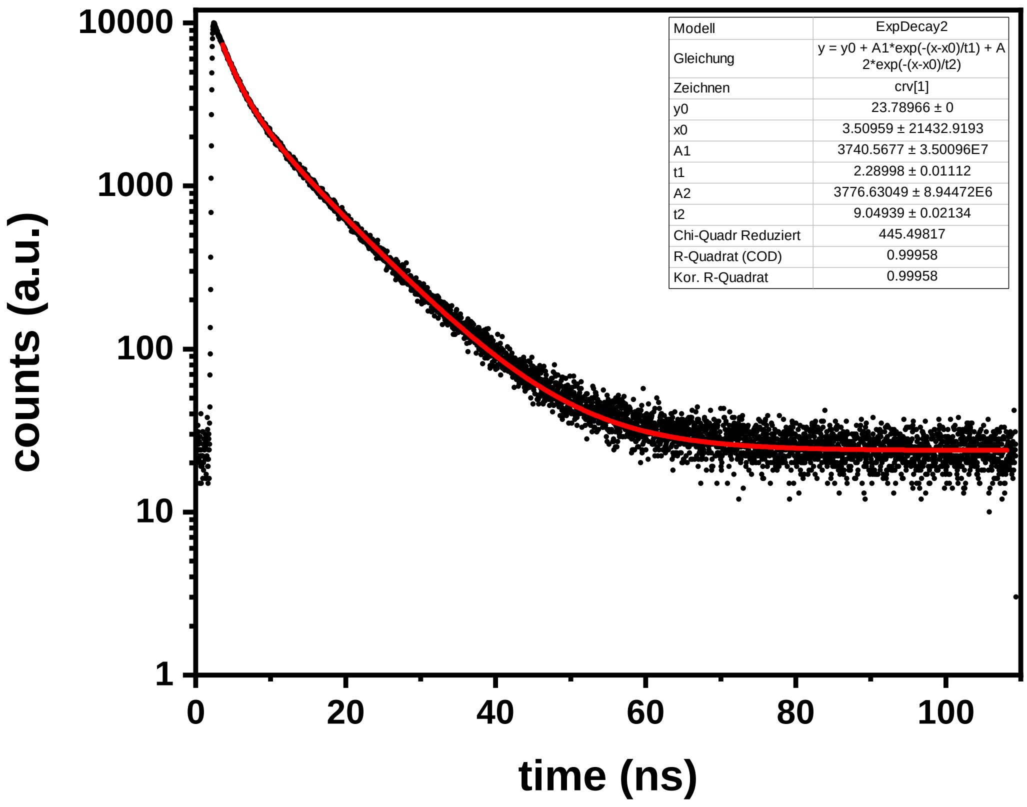

Figure S91. Lifetime of the 425 nm emission of **(*NC*^{NMe2}*N*)BiI$_2$** in THF at r.t.

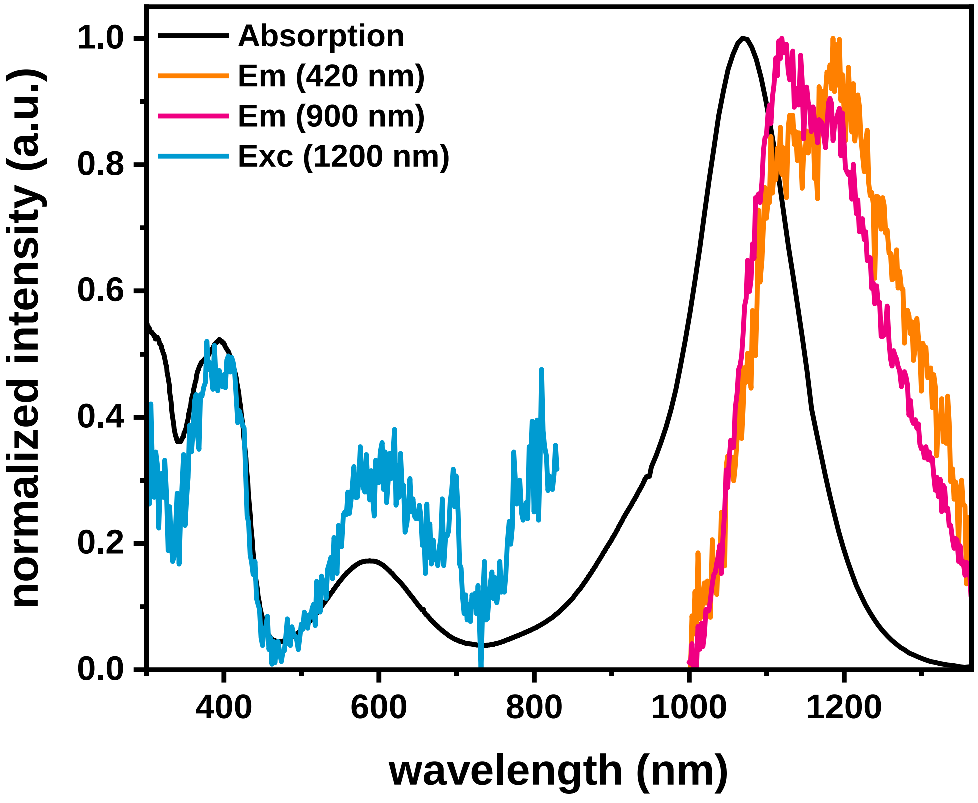

Figure S92. PL data of **[*NCH*^{NMe2}*N*]$^+$** at r.t. in degassed CH_2Cl_2. Absorption spectra are shown as black, emission spectra as orange and pink, and excitation spectra as blue lines.

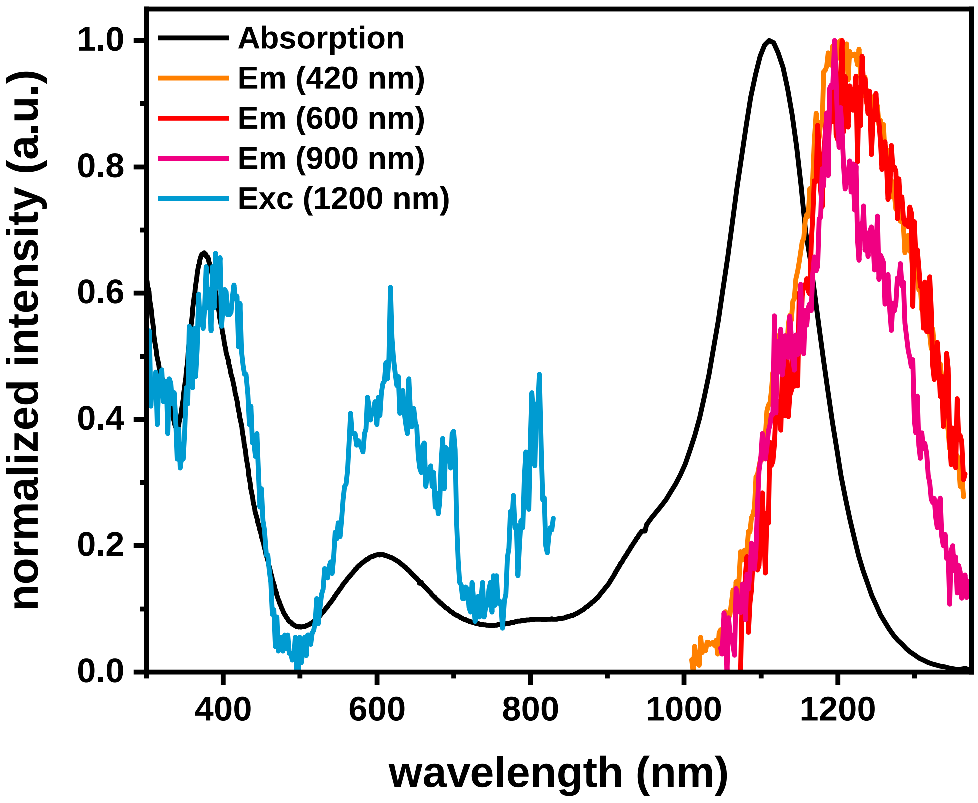

Figure S93. PL data of **[(*NC*^{NMe2}*N*)BiI$_2$]$^+$** at r.t. in degassed CH_2Cl_2. Absorption spectra are depicted as black, emission spectra as orange, red, and pink, and excitation spectra as blue lines.

Electrochemistry

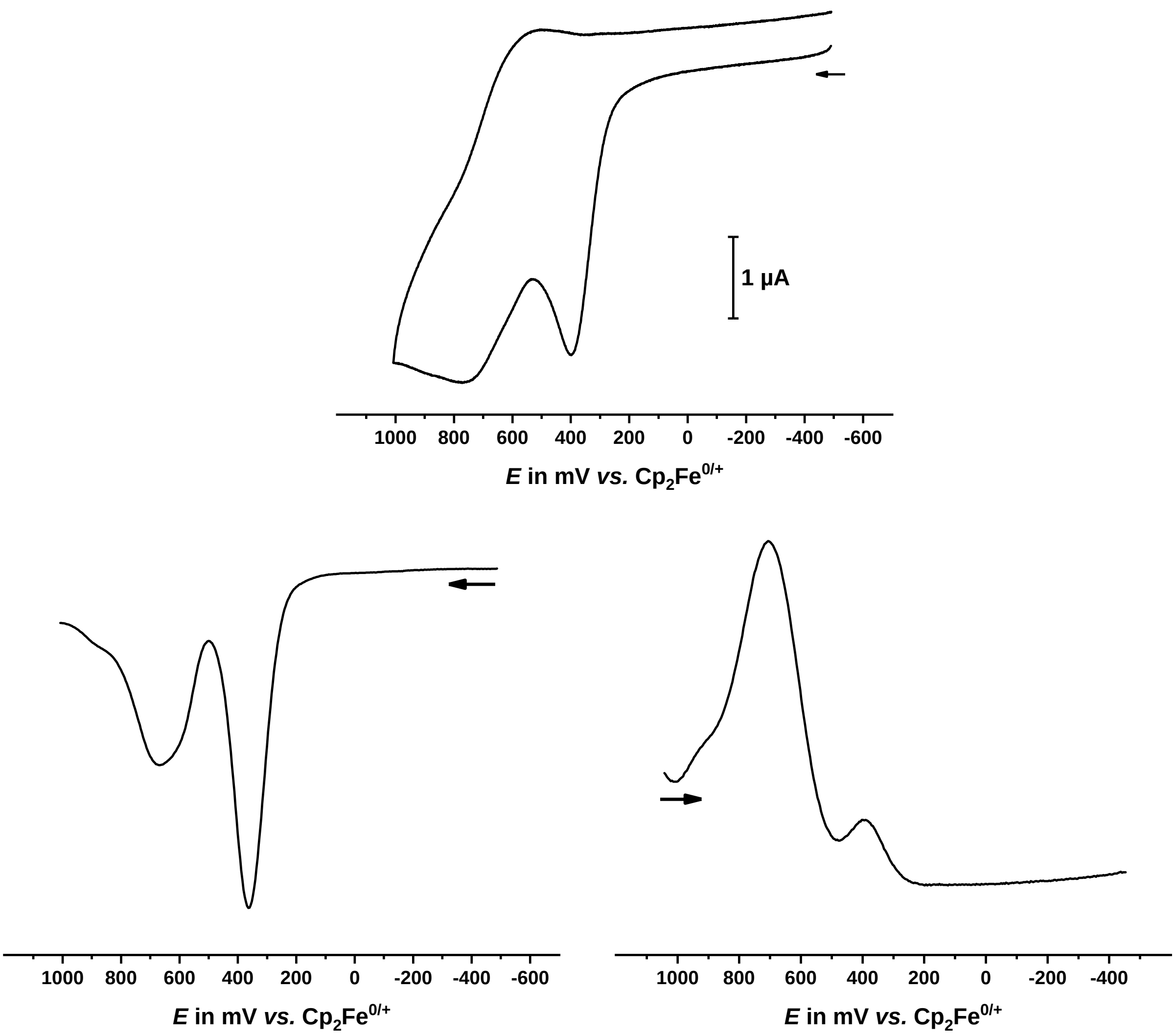

Figure S94. Top and bottom right: Cyclic (top) and square wave (bottom left and bottom right) voltammograms of ***NCH^{Me}N*** (0.1 M $^{n}Bu_4N^+$ $[BAr^{F24}]^-/CH_2Cl_2$ at v = 100 mV/s, r.t.).

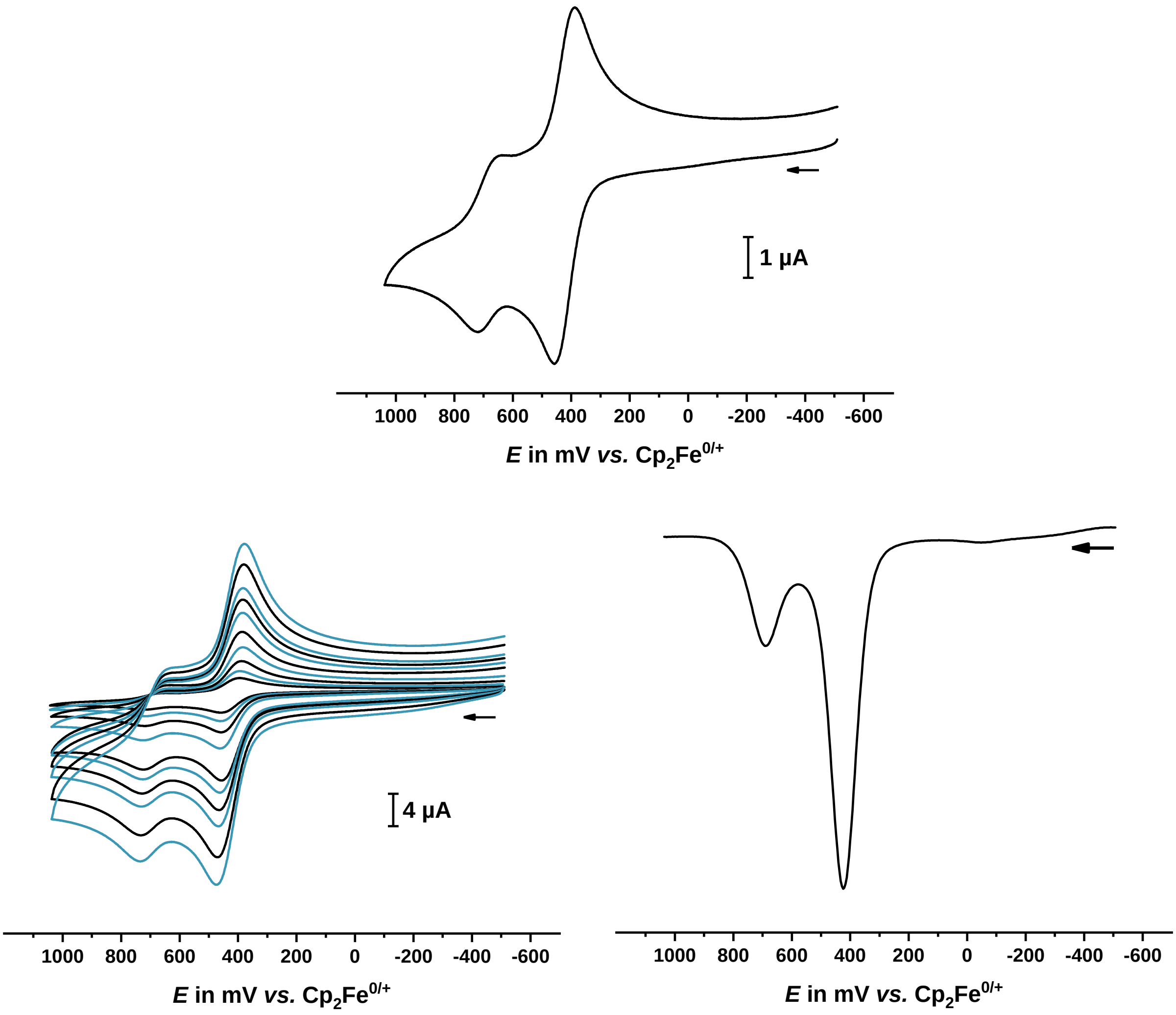

Figure S95. Top and bottom right: Cyclic and square wave voltammograms of **(*NC*^{Me}*N*)BiCl$_2$** (0.1 M $^{n}Bu_4N^+$ $[BAr^{F24}]^-/CH_2Cl_2$ at *v* = 100 mV/s, r.t.); bottom left: cyclic voltammograms of **(*NC*^{Me}*N*)BiCl$_2$** at v = 25, 50, 100, 200, 400, 600, 800, 1000, 1500, and 2000 mV/s.

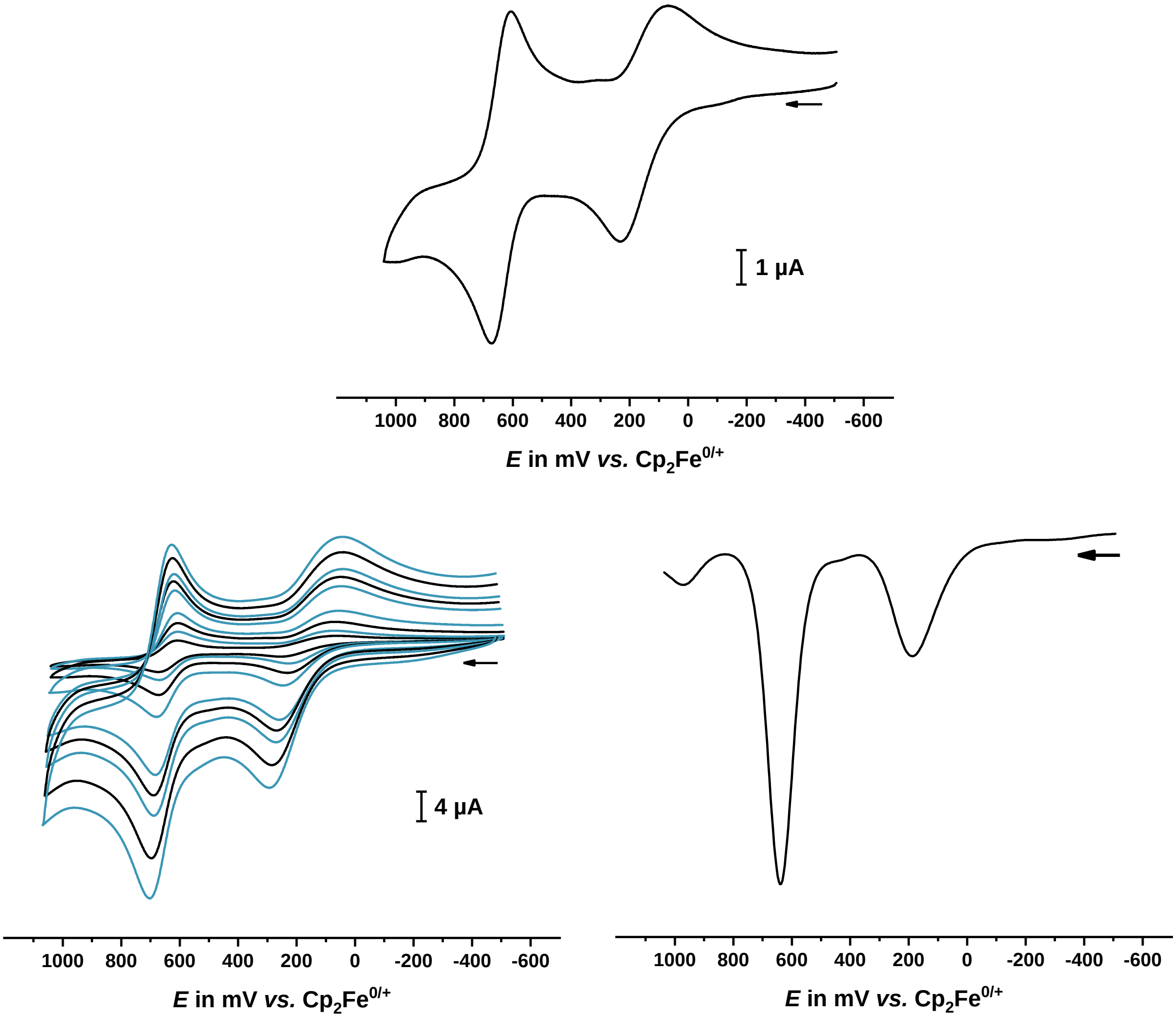

Figure S96. Top and bottom right: Cyclic and square wave voltammograms of **(*NC*^{Me}*N*)BiI$_2$** (0.1 M ^{n}Bu$_4$N$^+$ [BAr^{F24}]$^-$/CH$_2$Cl$_2$ at *v* = 100 mV/s, r.t.); bottom left: cyclic voltammograms of **(*NC*^{Me}*N*)BiI$_2$** at v = 25, 50, 100, 200, 600, 800, 1000, 1500, and 2000 mV/s.

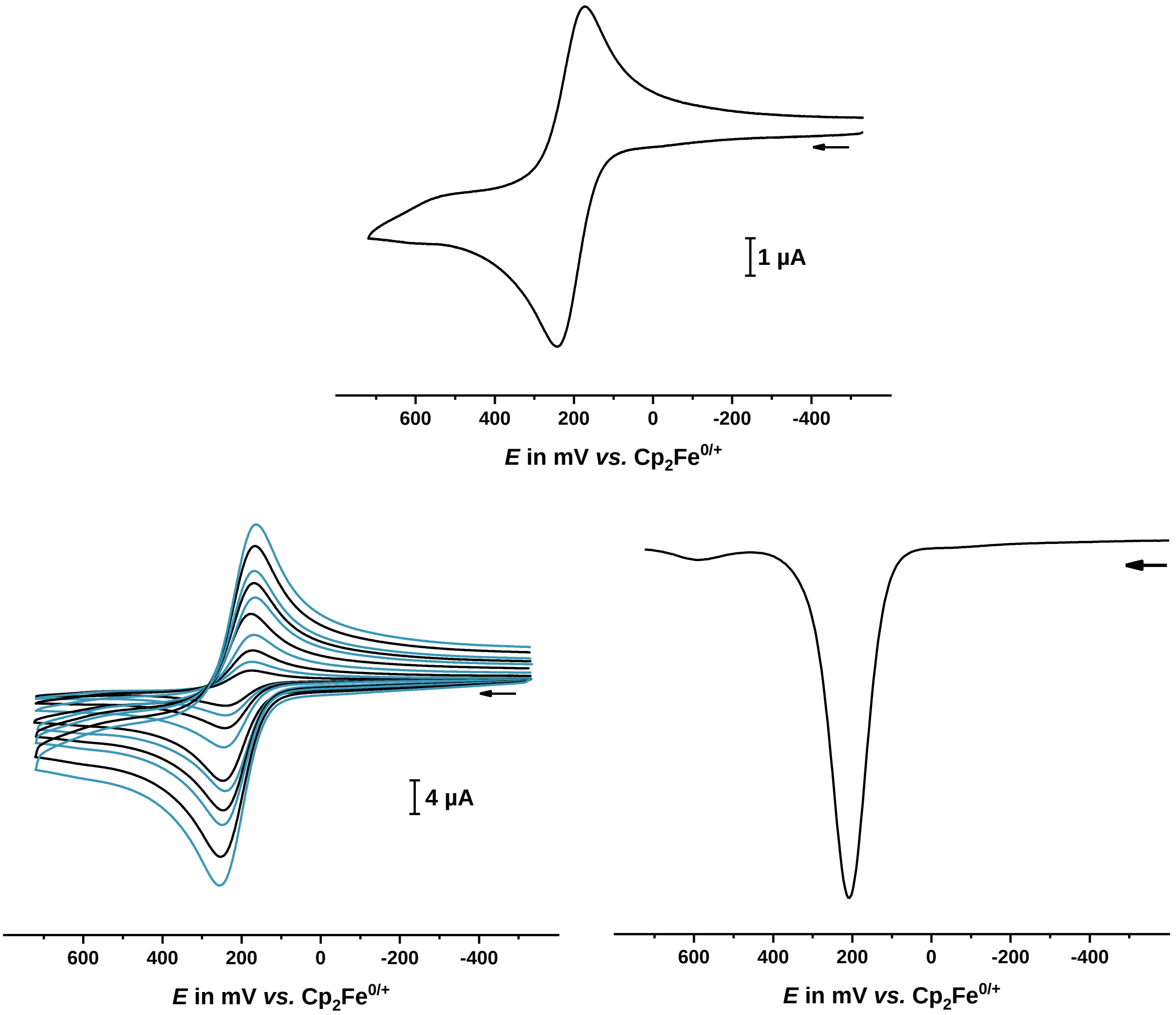

Figure S97. Top and bottom right: Cyclic and square wave voltammograms of ***NCH***$^{\mathbf{\mathit{OMe}}}$***N*** (0.1 M $^{n}Bu_4N^+$ $[BAr^{F24}]^-/CH_2Cl_2$ at v = 100 mV/s, r.t.); bottom left: cyclic voltammograms of ***NCH***$^{\mathbf{\mathit{OMe}}}$***N*** at v = 25, 50, 100, 200, 400, 600, 800, 1000, 1500, and 2000 mV/s.

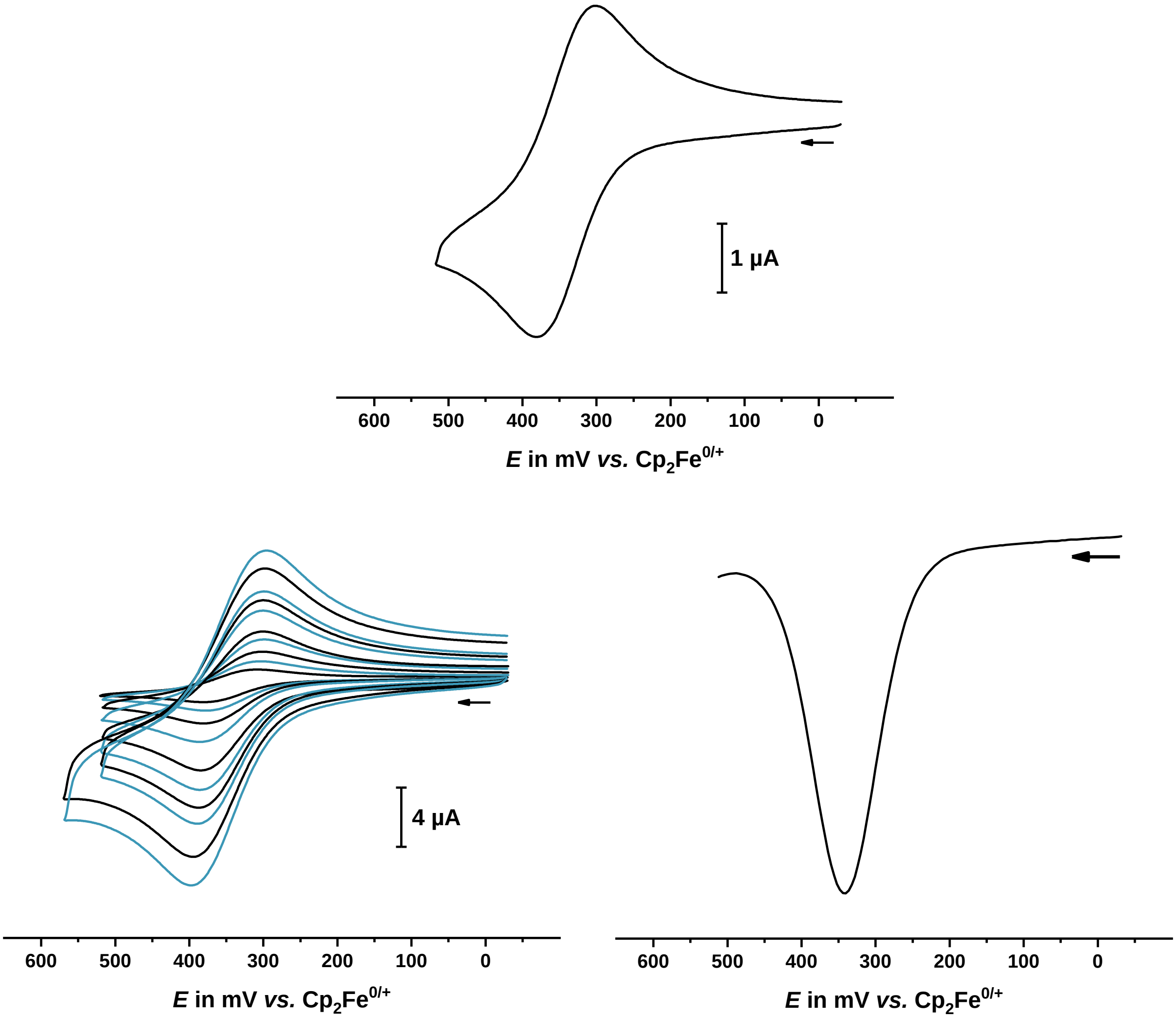

Figure S98. Top and bottom right: Cyclic and square wave voltammograms of **(*NC*^{OMe}*N*)BiCl$_2$** (0.1 M ^{n}Bu$_4$N$^+$ [BAr^{F24}]$^-$/CH$_2$Cl$_2$ at *v* = 100 mV/s, r.t.); bottom left: cyclic voltammograms of **(*NC*^{OMe}*N*)BiCl$_2$** at *v* = 25, 50, 100, 200, 400, 600, 800, 1000, 1500, and 2000 mV/s.

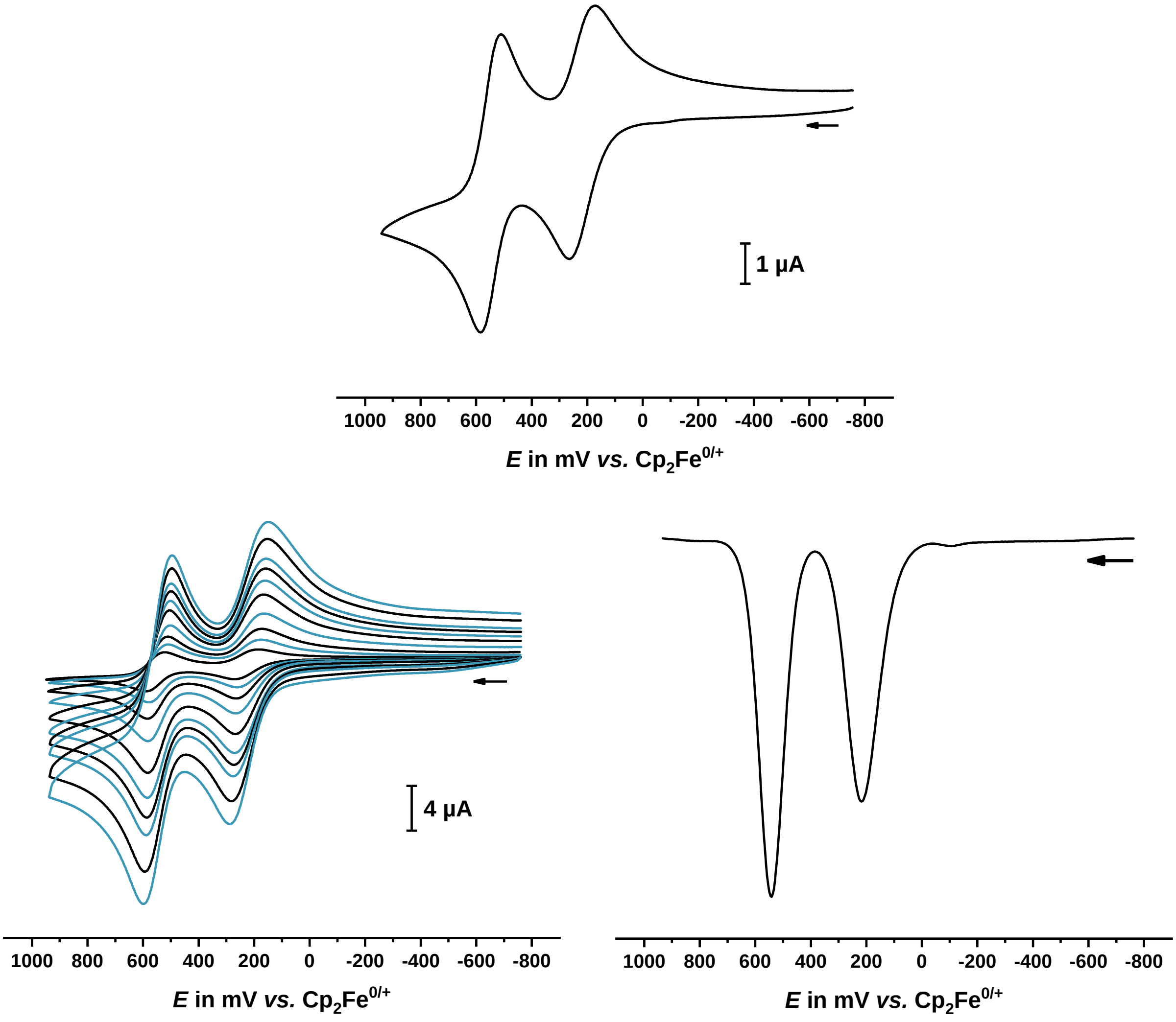

Figure S99. Top and bottom right: Cyclic and square wave voltammograms of **(*NC*^{OMe}*N*)BiI$_2$** (0.1 M $^{n}Bu_4N^+$ $[BAr^{F24}]^-/CH_2Cl_2$ at v = 100 mV/s, r.t.); bottom left: cyclic voltammograms of **(*NC*^{OMe}*N*)BiI$_2$** at v = 25, 50, 100, 200, 400, 600, 800, 1000, 1500, and 2000 mV/s.

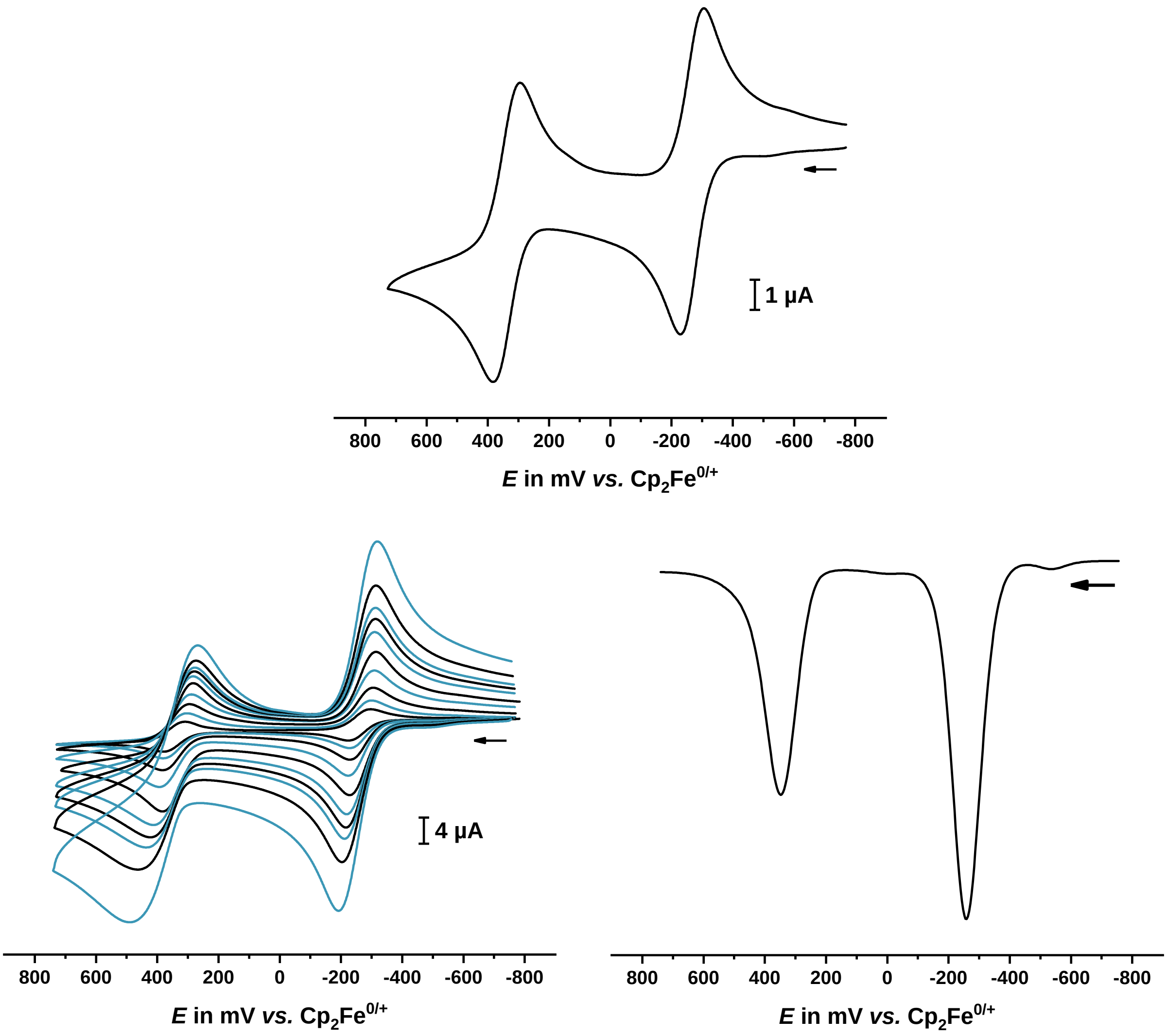

Figure S100. Top and bottom right: Cyclic and square wave voltammograms of ***NCH^{NMe2}N*** (0.1 M $^{n}Bu_4N^+$ $[BAr^{F24}]^-/CH_2Cl_2$ at v = 100 mV/s, r.t.); bottom left: cyclic voltammograms of ***NCH^{NMe2}N*** at v = 25, 50, 100, 200, 400, 600, 800, 1000, 1500, and 2000 mV/s.

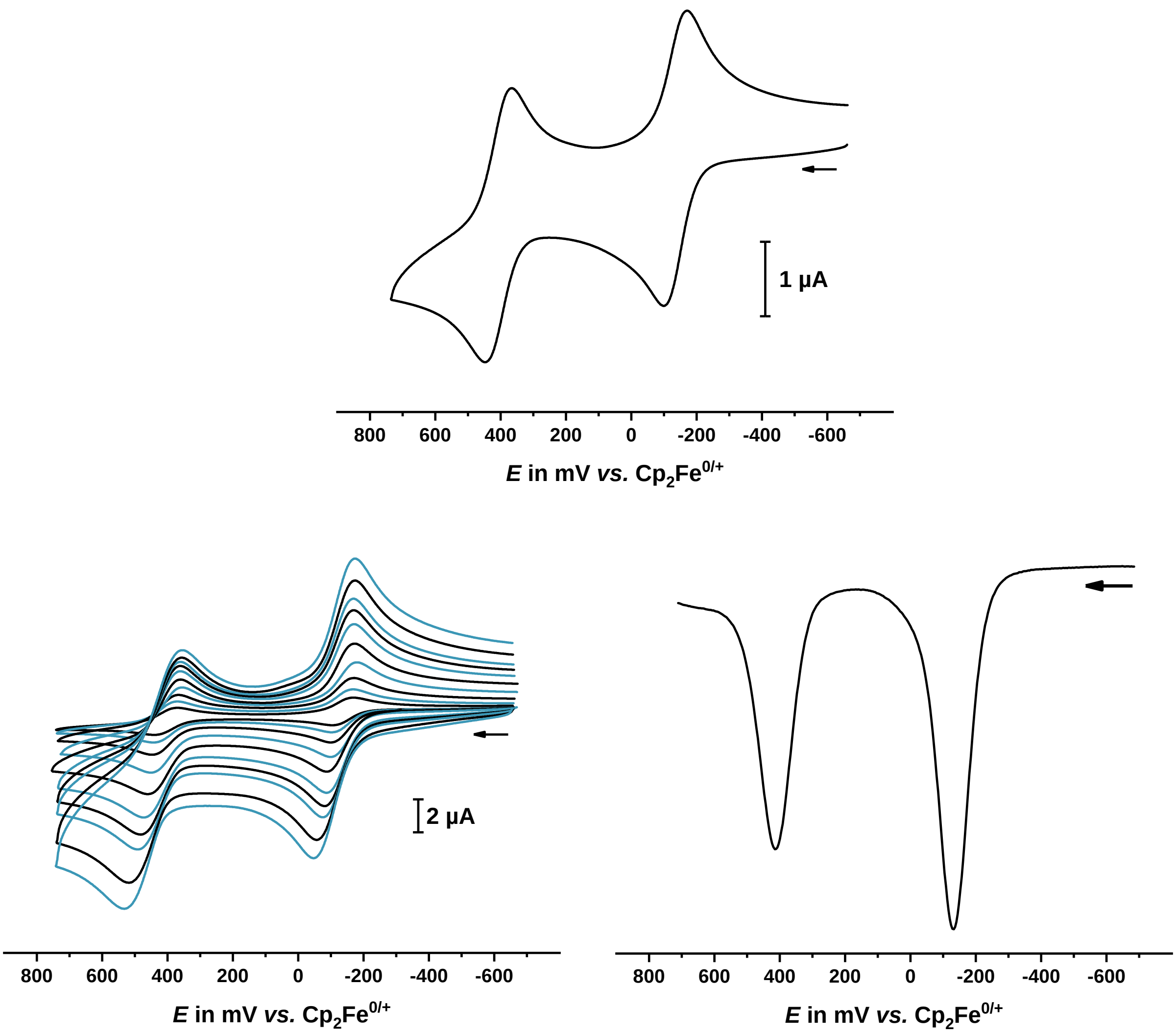

Figure S101. Top and bottom right: Cyclic and square wave voltammograms of **(*NC*^{NMe2}*N*)BiCl$_2$** (0.1 M $^{n}Bu_4N^+$ $[BAr^{F24}]^-/CH_2Cl_2$ at *v* = 100 mV/s, r.t.); bottom left: cyclic voltammograms of **(*NC*^{NMe2}*N*)BiCl$_2$** at *v* = 25, 50, 100, 200, 400, 600, 800, 1000, 1500, and 2000 mV/s.

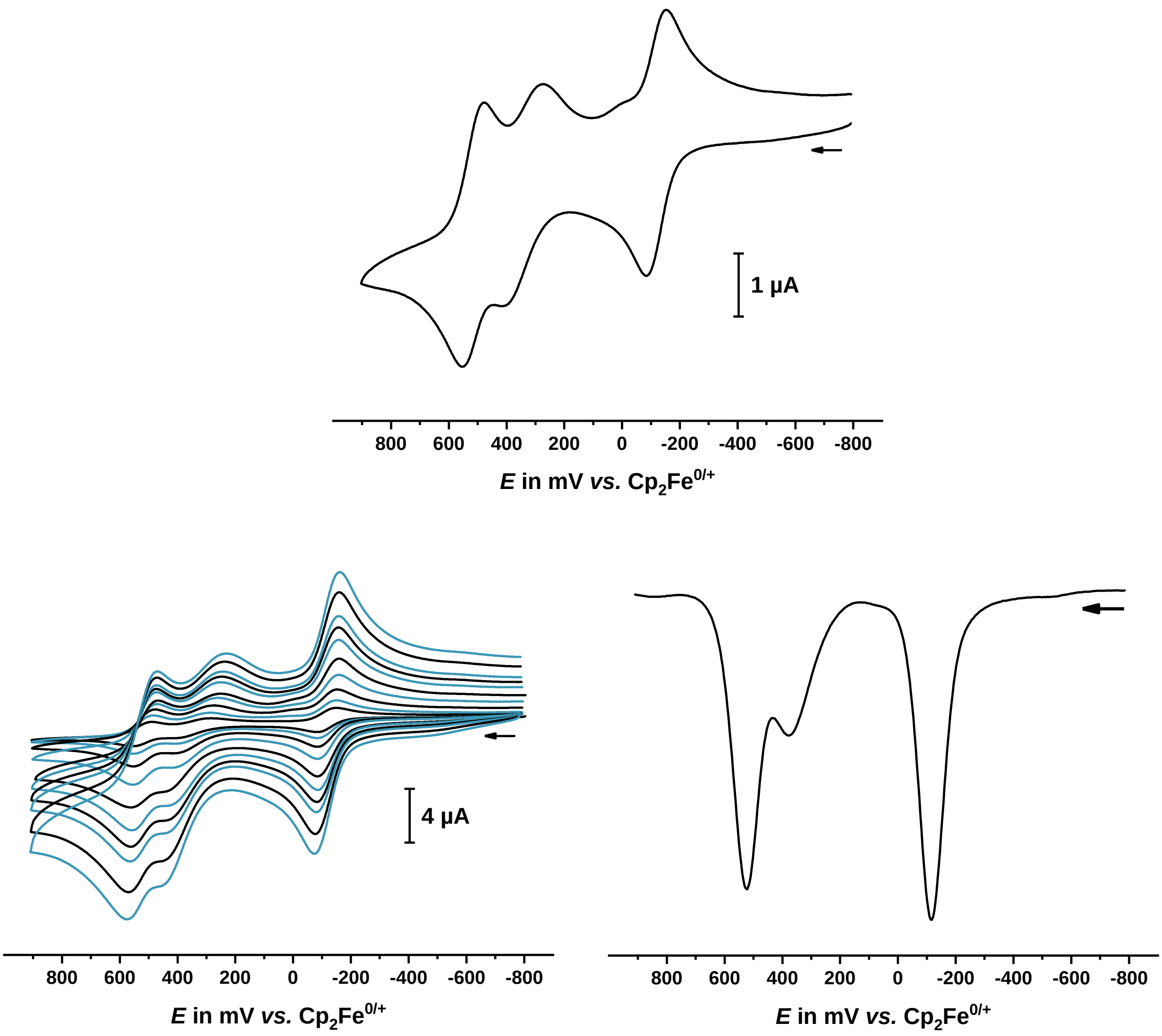

Figure S102. Top and bottom right: Cyclic and square wave voltammograms of **(NC^{NMe2}N)BiI$_2$** (0.1 M $^{n}Bu_4N^+$ $[BAr^{F24}]^-/CH_2Cl_2$ at v = 100 mV/s, r.t.); bottom left: cyclic voltammograms of **(NC^{NMe2}N)BiI$_2$** at v = 25, 50, 100, 200, 400, 600, 800, 1000, 1500, and 2000 mV/s.

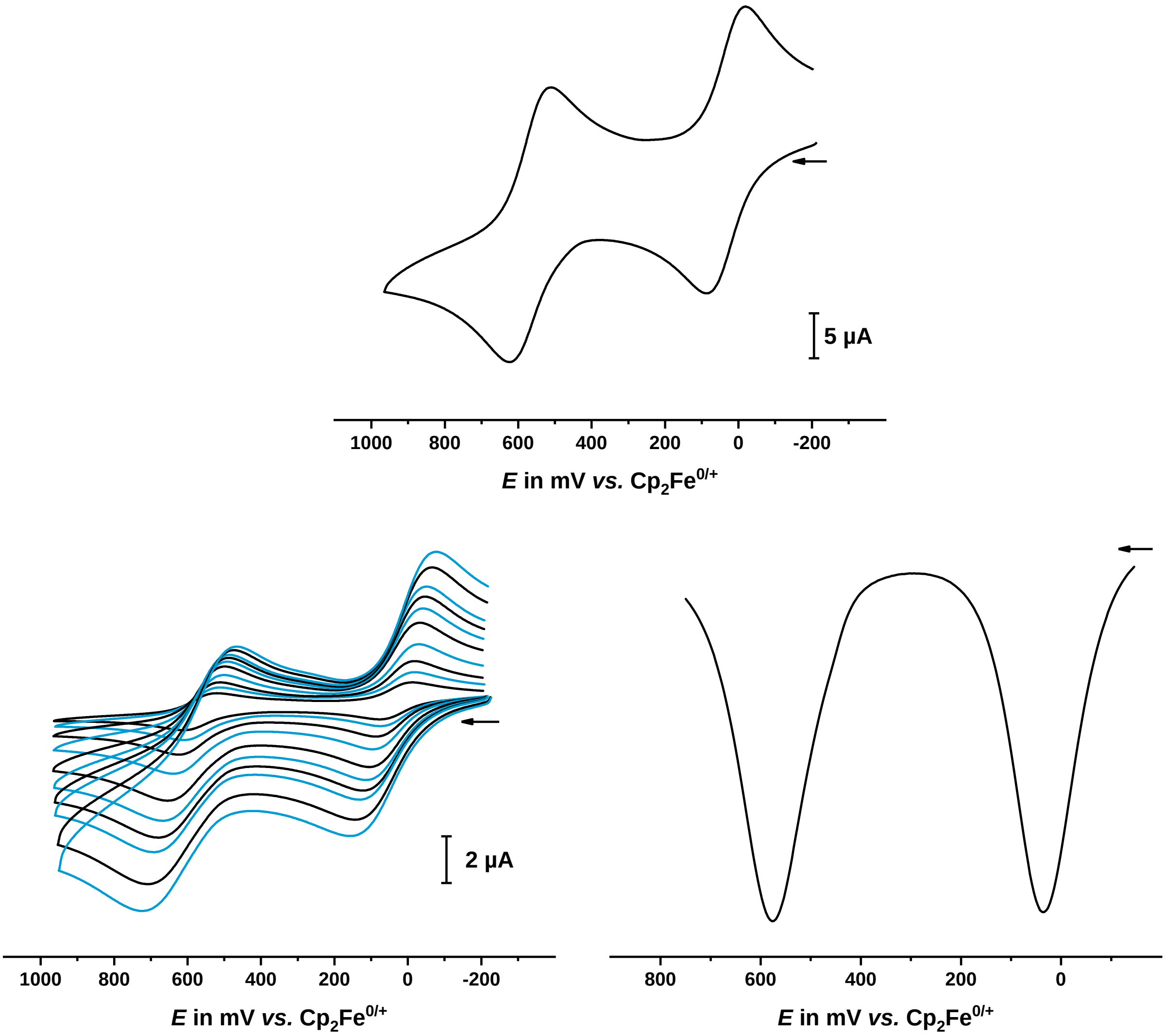

Figure S103. Top and bottom right: Cyclic and square wave voltammograms of ***NCH^{NMe2}NH^{+}*** (0.1 M $^{n}Bu_4N^+$ $[BAr^{F24}]^-/CH_2Cl_2$ at *v* = 100 mV/s, r.t.); bottom left: cyclic voltammograms of ***NCH^{NMe2}NH^{+}*** at v = 25, 50, 100, 200, 400, 600, 800, 1000, 1500, and 2000 mV/s. The protonation was performed by adding one equivalent of $HBAr^{F24}$ to ***NCH^{NMe2}NH^{+}*** in the CV cell.

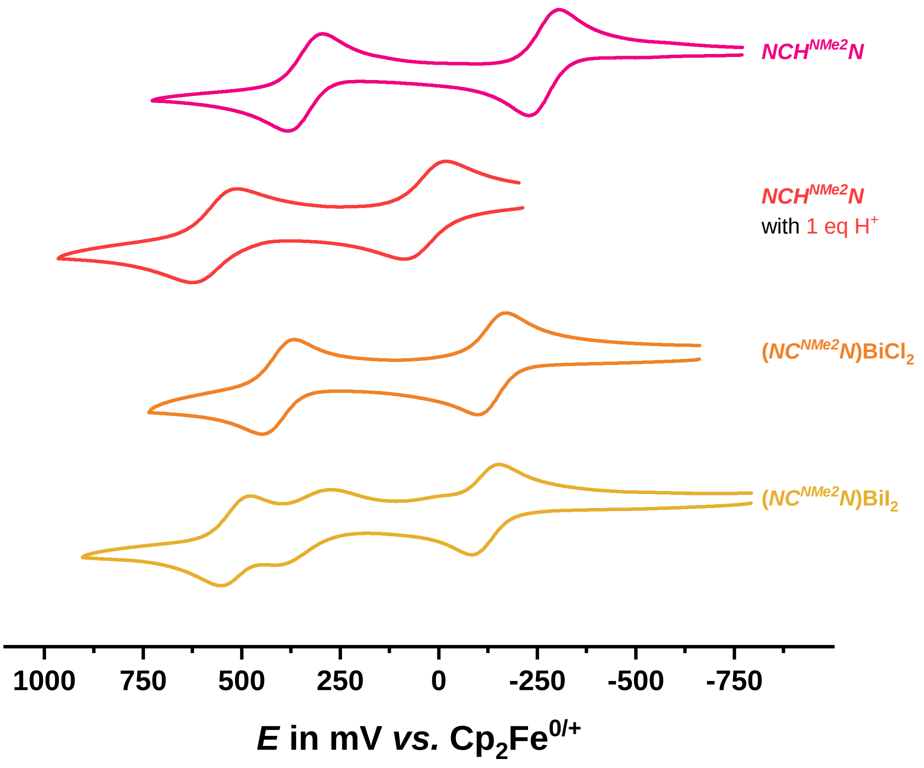

Figure S104. Comparison of the cyclic voltammograms of ***NCHN^{Me2}N***, ***NCHN^{Me2}NH$^+$***, **(*NC^{NMe2}N*)BiCl$_2$** and **(*NC^{NMe2}N*)BiI$_2$** (0.1 M ^{n}Bu$_4$N$^+$ [BAr^{F24}]$^-$/CH$_2$Cl$_2$ at v = 100 mV/s, r.t.).

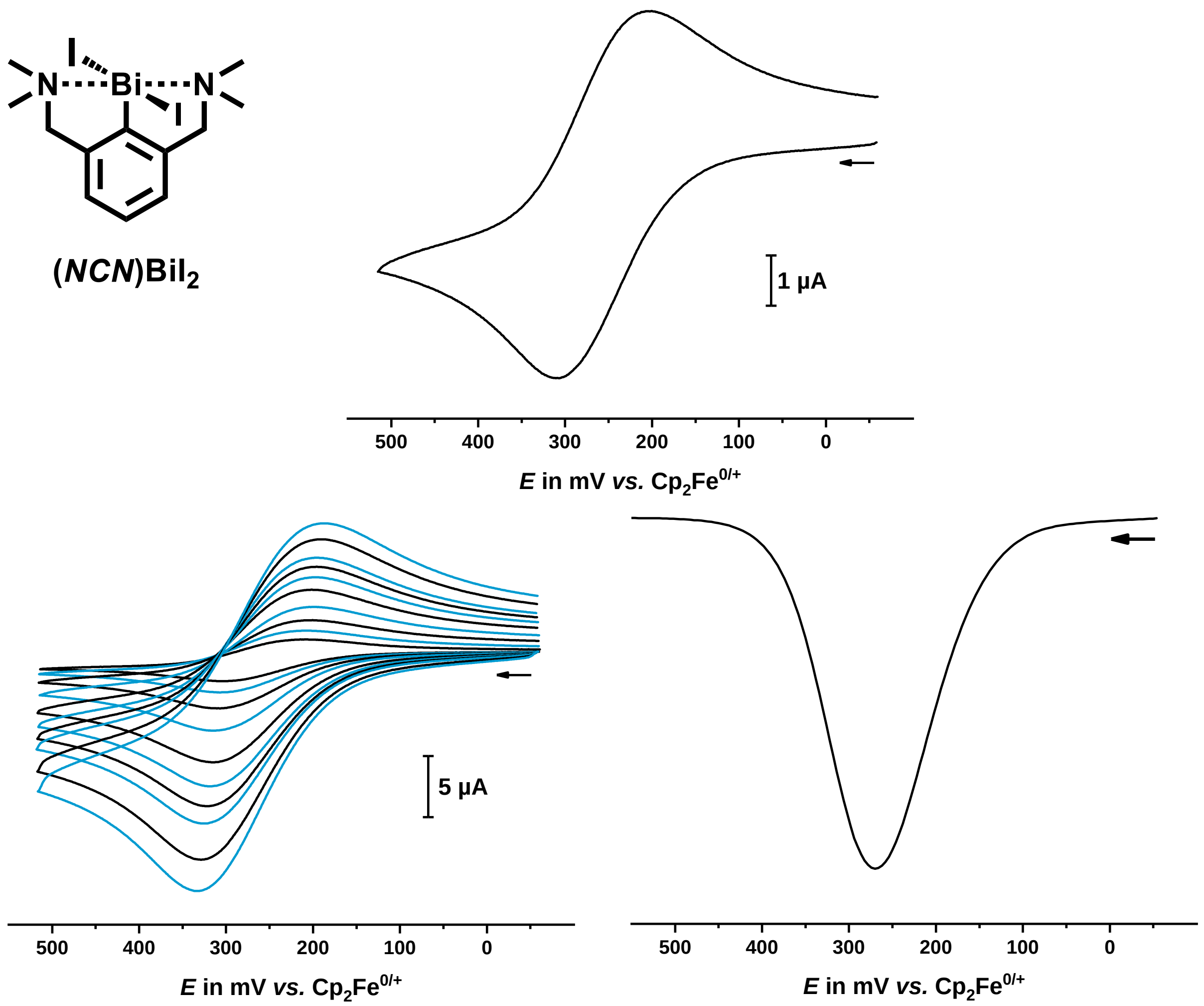

Figure S105. Top and bottom right: Cyclic and square wave voltammograms of **(*NCN*)BiI$_2$** (0.1 M ^{n}Bu$_4$N$^+$ [BAr^{F24}]$^-$/CH$_2$Cl$_2$ at v = 100 mV/s, r.t.); bottom left: cyclic voltammograms of **(*NCN*)BiI$_2$** at v = 25, 50, 100, 200, 400, 600, 800, 1000, 1500, and 2000 mV/s.

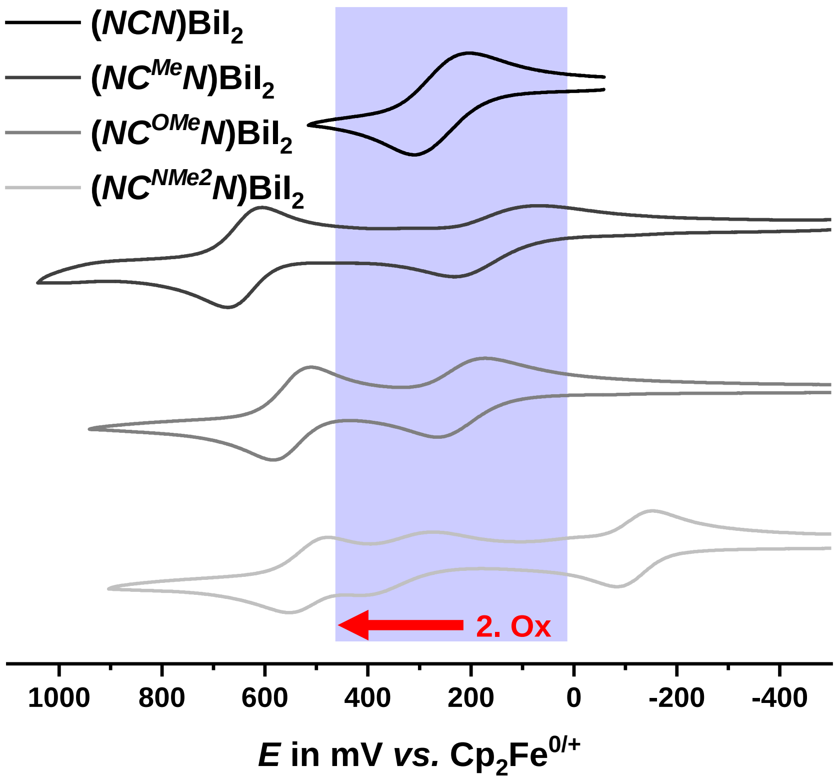

Figure S106. Comparison of the cyclic voltammograms of **(*NCN*)BiI$_2$** and **(*NC^{R}N*)BiI$_2$** (R = Me, OMe, NMe$_2$) (0.1 M ^{n}Bu$_4$N$^+$ [BAr^{F24}]$^-$/CH$_2$Cl$_2$ at v = 100 mV/s, r.t.).

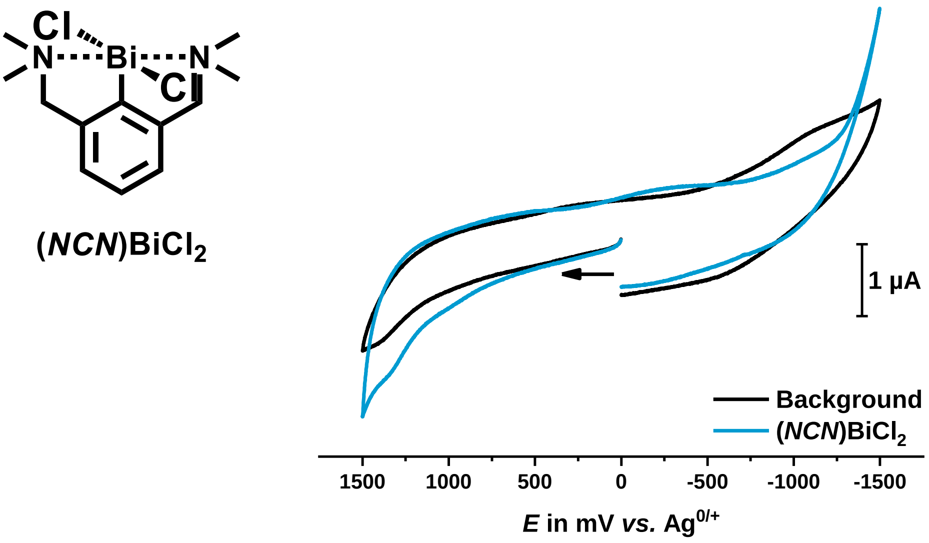

Figure S107. Cyclic voltammograms of **(NCN)BiCl$_2$** and the electrolyte background (0.1 M ^{n}Bu$_4$N$^+$ [BAr^{F24}]$^-$/CH$_2$Cl$_2$ at v = 100 mV/s, r.t.).

EPR Spectroscopy

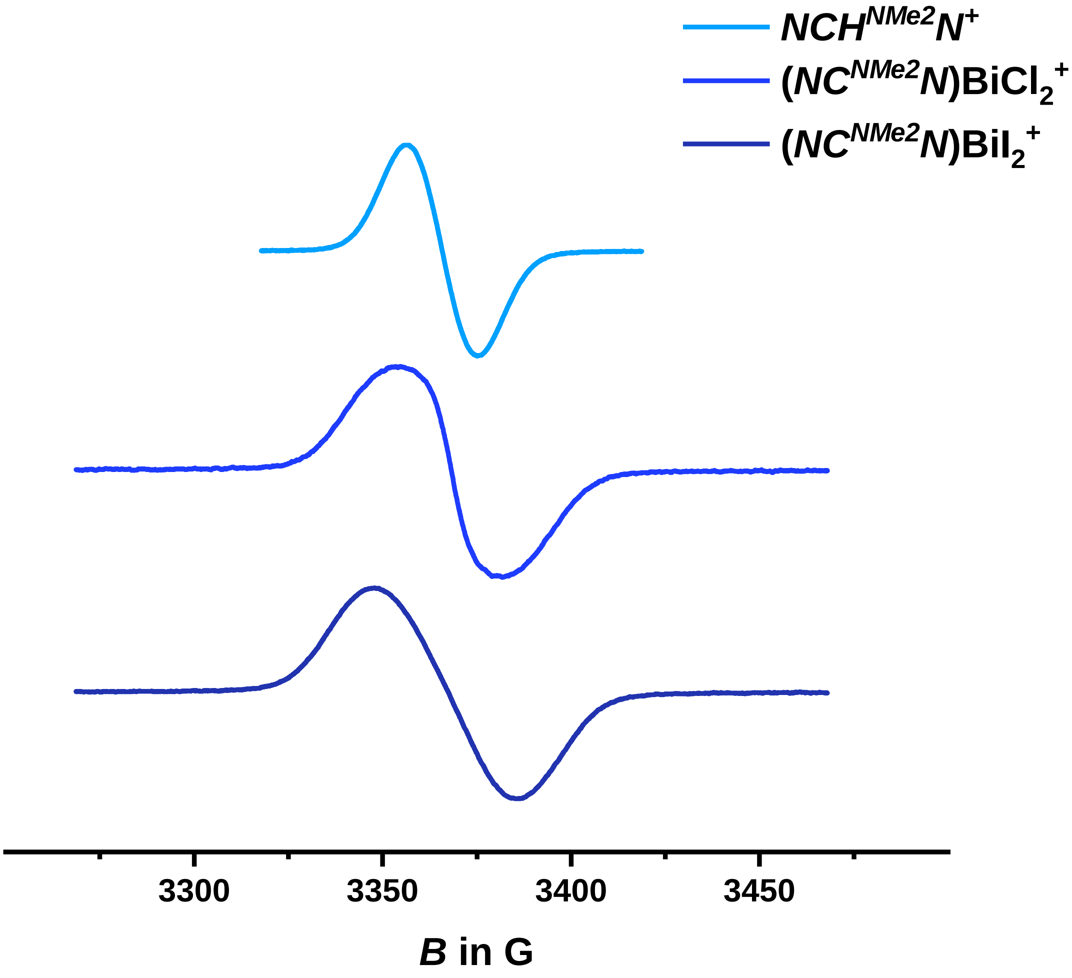

Figure S108. Experimental solution EPR spectra of [***NCH***$^{\textit{NMe2}}$***N***]$^+$, [(***NCN***$^{\textit{NMe2}}$)**BiCl$_2$**]$^+$, and [(***NCN***$^{\textit{NMe2}}$)**BiI$_2$**]$^+$ in CH_2Cl_2 at r.t.

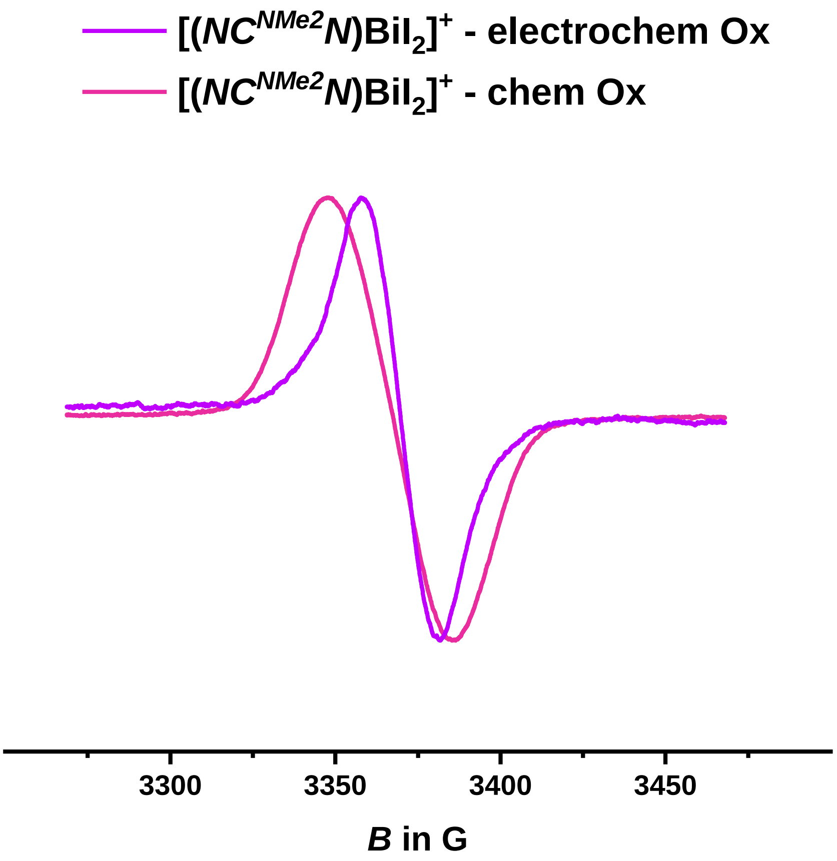

Figure S109. Comparison of EPR spectra of chemically (NO^+ $[SbF_6]^-$) oxidized **[(*NC*^{NMe2}*N*)BiI$_2$]$^+$** chemically oxidized with electrochemically oxidized **[(*NC*^{NMe2}*N*)BiI$_2$]$^+$**.
